# Synthesis and Activity of 2-Acyl-cyclohexane-1,3-dione Congeners Derived from *Peperomia* Natural Products against the Plant p-Hydroxyphenylpyruvate Dioxygenase Herbicidal Molecular Target Site

**DOI:** 10.3390/plants11172269

**Published:** 2022-08-31

**Authors:** Joey K. Ooka, Mauro V. Correia, Marcus T. Scotti, Harold H. Fokoue, Lydia F. Yamaguchi, Massuo J. Kato, Franck E. Dayan, Daniel K. Owens

**Affiliations:** 1 Department of Molecular Biosciences and Bioengineering, University of Hawaii-Manoa, Honolulu, HI 96822, USA; 2 Institute of Chemistry, University of Brasilia, Brasilia 70910-900, DF, Brazil; 3 Department of Chemistry, Federal University of Paraiba, Brasilia 58051-900, PB, Brazil; 4 Laboratório de Avaliação e Síntese de Substâncias Bioativas (LASSBio®), Instituto de Ciências Biomédicas, Centro das Ciências da Saúde, Universidade Federal do Rio de Janeiro, Rio de Janeiro 21941-901, RJ, Brazil; 5 Institute of Chemistry, University of São Paulo, São Paulo 03001-000, SP, Brazil; 6 Agricultural Biology, Colorado State University, Fort Collins, CO 80526, USA

**Keywords:** natural products, triketones, p-hydroxyphenylpyruvate dioxygenase, herbicide mode of action, phytotoxins, quantitative structure-activity relationships, synthesis, essential

## Abstract

Plastoquinone is a key electron carrier in photosynthesis and an essential cofactor for the biosynthesis of carotenoids. p-Hydroxyphenylpyruvate dioxygenase (HPPD) is a vital enzymatic step in plastoquinone biosynthesis that is the target of triketone herbicides, such as those derived from the pharmacophore backbone of the natural product leptospermone. In this work, the inhibitory activity of a series of 2-acyl-cyclohexane-1,3-diones congeners derived from *Peperomia* natural products was tested on plant HPPD. The most active compound was a 2-acyl-cyclohexane-1,3-dione with a C_11_ alkyl side chain (**5d**; I_50_app: 0.18 ± 0.02 μM) that was slightly more potent than the commercial triketone herbicide sulcotrione (I_50_app: 0.25 ± 0.02 μM). QSAR analysis and docking studies were performed to further characterize the key structural features imparting activity. A 1,3-dione feature was required for inhibition of HPPD. Molecules with a side chain of 11 carbons were found to be optimal for inhibition, while the presence of a double bond, hydroxy, or methyl beyond the required structural features on the cyclohexane ring generally decreased HPPD inhibiting activity.

## 1. Introduction

The mechanism of action of triketone herbicides is through inhibition of the enzyme, p-hydroxyphenylpyruvate dioxygenase (HPPD) (Group 27) [1,2,3,4,5,6,7]. HPPD catalyzes the oxidation of 4-hydroxyphenylpyruvate (HPP) to homogentisic acid (HGA) which is a key precursor for the production of tocochromanols and prenyl quinones (e.g., plastoquinone). The herbicidal activity of HPPD inhibitors is directly correlated with reductions in the cellular pool of plastoquinone. Plastoquinone is a central intermediate in photosynthetic electron transport and an essential cofactor for phytoene desaturase (PDS) activity. PDS is a well-known critical enzymatic step in the biosynthesis of the photoprotecting carotenoids [8]. Inhibition of PDS leads to the bleaching of newly expanding foliage, which is a characteristic phenotype that has long been associated with plants treated with HPPD inhibitors [3].

**Figure 1:**
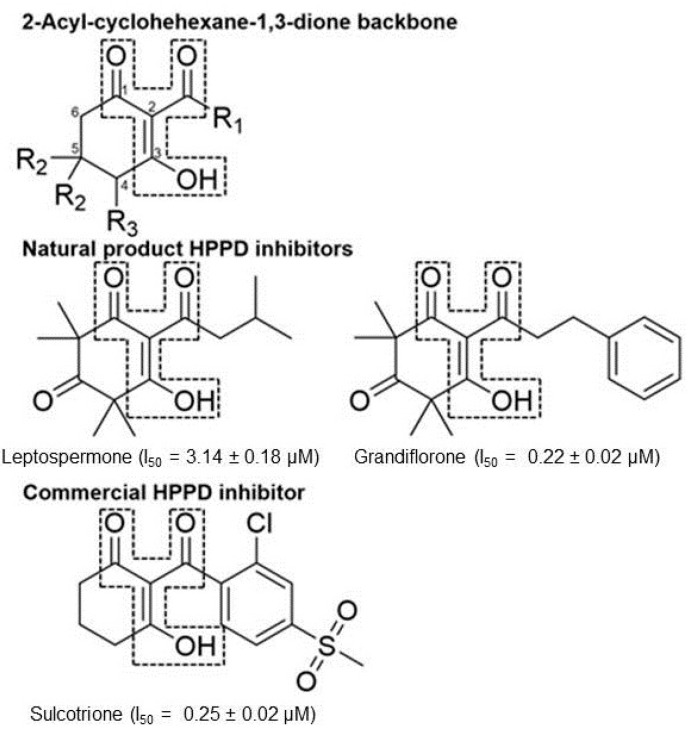
Comparison of the 2-acyl-cyclohexane-1,3-dione backbone to natural product and commercial compounds of known HPPD inhibitory activity. The region within the dashed line represents the triketone moiety containing the 1,3 dione feature required for HPPD inhibitory activity. The ketone group at position 3 undergoes keto-enol isomerization to the form shown at physiological pH. The name of each compound is followed by the *I*_50_app as tested against recombinant *A. thaliana* HPPD.

Commercial triketone herbicides, such as sulcotrione, were originally discovered by derivatization using the natural product, leptospermone (Figure 1) as a base structure [9]. Essential oils from manuka (*Leptopermum scoparium*) are a rich source of natural triketones, including leptospermone and grandiflorone, which have been investigated as sources for the development of natural herbicides [10]. Manuka oil exhibits good pre-emergence control against a variety of weeds by inhibition of HPPD predominantly via the activity of leptospermone, which is the natural triketone found at the highest concentration within the oil [11]. This is unique, as the majority of natural-product herbicides tend to be of the postemergence, contact, burndown type. Leptospermone has several physicochemical features (e.g., logP and pKa values, molecular mass, number of hydrogen donors and acceptors and number of rotatable bonds) that enable ready absorption by the roots and translocation to the foliage, its predominant in planta site of action [12].

*Peperomia* is composed of approximately 1700 species [13] making it the second-largest of the four genera that form the *Piperaceae* family, all of which are known to be rich sources of natural products [14]. *Peperomia* species produce a wide range of secondary metabolites [15,16,17,18,19,20,21,22,23,24,25,26,27,28]. In particular, they are important natural sources of 2-acylcyclohexane-1,3-diones such as alatanone A (1a) and alatanone B (2a) from P. alata and trineurone A (1b), trineurone B (2b), trineurone C (2c), trineurone D (3a) and trineurone E (3b) from P. alata and P. trineura [29]. These polyketides have a number of beneficial roles such as acting as important chemical messengers in insects [30,31,32], pharmacological properties against several cancer cell lines [23,33,34], as well as antifungal and antimicrobial activities [29]. Structurally, 2-acyl-cyclohexane-1,3-diones have features reminiscent of leptospermone and commercial triketone herbicides such as sulcotrione (Figure 1) [2,35]. 

Structure–activity relationships (SAR) have been previously investigated by agrochemical companies utilizing the backbone of leptospermone, which has led to the development and marketing of a number of commercial weed management tools [1,2,3,4,5,36]. Using a similar strategy, we have conducted an extensive structure activity analysis program generating 76 2-acylcyclohexane-1,3-diones and related analogues from *Peperomia* natural product 2-acyl-cyclohexane-1,3-dione base structures which were all tested as inhibitors of HPPD enzymatic activity. The resulting data set was subsequently analyzed using three-dimensional quantitative structure–activity relationship (3D-QSAR) analysis to characterize the key structural features that contribute to inhibition of HPPD activity.

## 2. Results and Discussion

### 2.1. Synthesis of 2-Acyl-cyclohexane-1,3-diones and Analogs

In this study, 76 compounds were synthesized and verified using spectrometric and spectroscopic means (Figures S8–S159, supplemental spectroscopic data). All compounds were synthesized according to literature procedures without major modification (Supplemental Schemes S1–S5). Yields were not optimized, as the primary goal of the work was to obtain pure products in sufficient quantities for HPPD inhibition assays. 

### 2.2. Inhibition of HPPD

**Figure 2:**
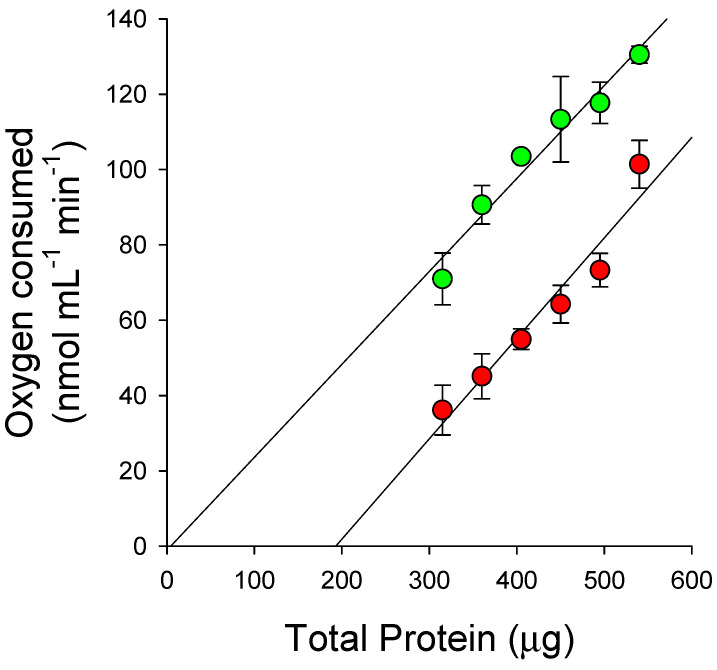
Titration of HPPD activity in TSP extract as measured by oxygen consumption in the presence of 0 (●) and 3.75 μM of **4b** (●). The reaction mixture contained 0.2 M sodium phosphate buffer (pH 7.2), 1.8 mM ascorbate, 0.2 mM HPP, total soluble protein (0.3–0.5 mg) in a total volume of 3 mL and was incubated at 37 °C. Enzyme and inhibitor were incubated together for 3 min prior to the initiation of the enzymatic reaction by the addition of substrate.

Inhibitors of HPPD can interact with the target site in different manners, depending upon their structure. Most triketone-type inhibitors exhibit time-dependent (tight-binding) inhibition [37,38], resulting in the formation of highly stable enzyme-inhibitor complexes with no loss of ferrous iron in the catalytic domain [35]. The tight-binding nature of the interaction causes extremely slow release of the herbicide from the target site (in the range of hours to days), resulting in triketone herbicides mimicking the characteristics of irreversible inhibitors [39,40]. A previous comparison between the binding of natural triketones and quinones to HPPD showed that the triketones interacted as slow tight-binding inhibitors, whereas naphthoquinones and benzoquinones behaved as equilibrium-based reversible inhibitors [41]. Enzyme titration studies with compound **4b** resulted in linear data across the 0 and 3.75 μM inhibitor treatments that were parallel throughout the tested enzyme concentration ranges (Figure 2). This is consistent with the effective concentration of the enzyme having been reduced by the same amount in the presence of 3.75 μM of the inhibitor across the entire experiment, as would be predicted for an irreversible-like model of enzyme inhibition, as has been observed with other triketone compounds. Since the degree of inhibition of HPPD increases over time, the data shown in Table 1, Table 2, Table 3, Table 4 and Table 5 are recorded as relative I_50_app values obtained after 15 min of incubation, as has been performed in previous studies [33]. Due to the irreversible-like nature of inhibitor binding, longer incubation periods would be predicted to shift dose–response curves (Figures S1–S4) to the left, resulting in lower predicted I_50_app values.

### 2.3. Herbicidal Activity of Tested Compounds

**Figure 3:**
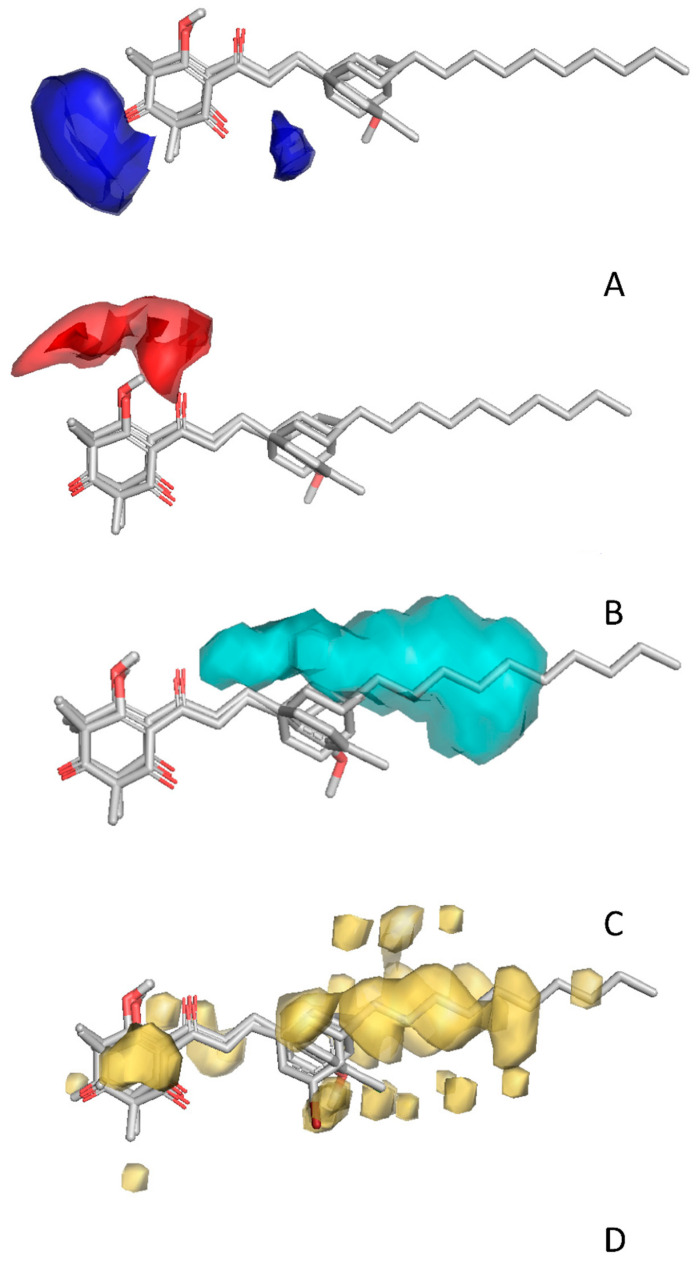
Structural features contributing positively to the activity of 2-acyl-cyclohexane-1,3-diones on HPPD. (**A**) Blue fields highlight hydrogen donor regions that promote activity. (**B**) Red fields highlight hydrogen acceptor regions that enhance activity. (**C**) Cyan fields identify the regions where increase in steric bulk increases activity. (**D**) Yellow fields delineate regions where hydrophobic interaction contributes to activity.

Overall, there was a wide range of activity among the 76 synthesized compounds in this study, including structures with apparent I_50_app in the nanomolar range (e.g., **5d**, **16b**, **18a**, etc.), many structures with activity in the mid-to-low micromolar range (e.g., **1b**, **8a**, **8b** and many others) and others with little to no activity (e.g., **1a**, **1d**, etc.) (Table 1, Table 2, Table 3, Table 4 and Table 5). Most of the inactive compounds did not possess a 1,3-dione pharmacophore capable of interacting with Fe ^2+^ at the HPPD active site, which is a minimal structural requirement for HPPD inhibitors (Table 5) [2,35,42]. The important contribution of the 1,3-dione toward activity was further suggested by 3D-QSAR analysis, as indicated by the blue, red and yellow fields that show interactions with the hydrogen-bonding acceptor, hydrogen-bonding donor and lipophilic fragments of the ligand, respectively, in Figure 3.

Of the 76 compounds screened, 25 (33%) had I_50_app values suggesting activity greater than that of leptospermone, the natural product that is typically identified at the highest concentrations in allelopathic plant tissues (Table 1, Table 2, Table 3, Table 4 and Table 5). The compound **5d** had greater inhibitory activity, while **16b** had a similar high activity level to the commercially produced sulcotrione and the most active identified natural product to date, grandiflorone. The most active compounds were either 2-acyl-cyclohexane-1,3-diones with simple aliphatic side chains of 9 or 11 carbons (**4b**, **4d**, **4e** and **5b**, **5d**, **5f** in Table 1) or were analogues of the grandiflorone backbone possessing a phenyl side chain (**16b**). The benefit of aliphatic side chains is consistent with previous reports [10,43] and was highlighted by positive steric (cyan) and hydrophobic (yellow) contributions in Figure 3. However, excessively large aliphatic chains, compounds possessing more than 11 carbons (**6a** and **6b** in Table 1) hinder activity, being much less active than those with 9 and 11 carbons, potentially due to steric hindrances [43].

**Figure 4:**
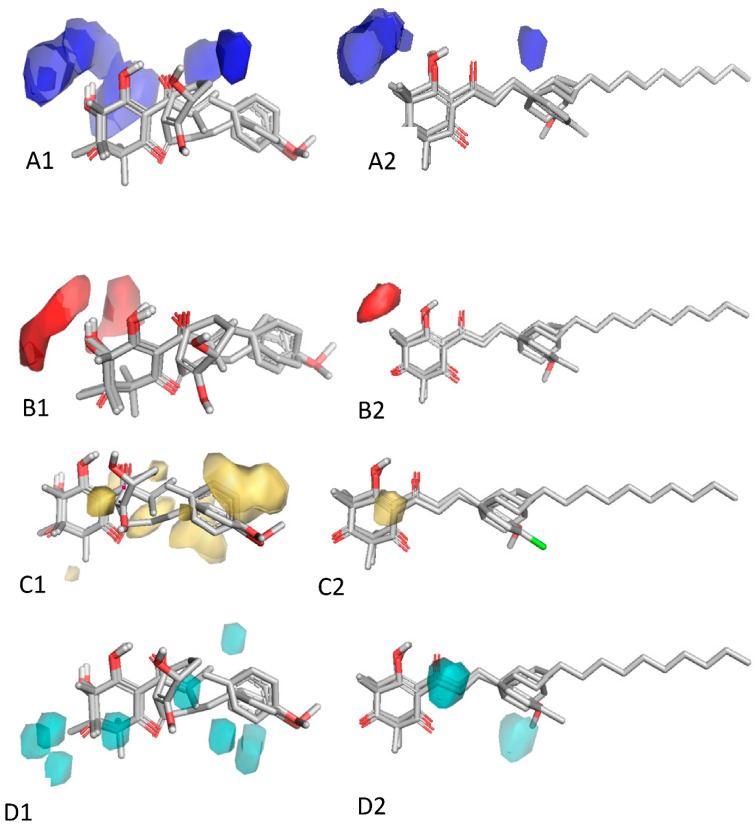
Structural features contributing negatively to the activity of 2-acyl-cyclohexane-1,3-diones on HPPD. (**A1**,**A2**) Blue fields highlight regions where the presence of hydrogen donors reduce activity. (**B1**,**B2**) Red fields highlight hydrogen acceptor regions that negatively affect activity. (**C1**,**C2**) Yellow fields identify hydrophobic regions near to the triketone ring that negatively affect activity. (**D1**,**D2**) Cyan fields delineate regions where steric interactions reduce activity.

In compounds with aromatic side chains, there was a trend for molecules with phenyl groups (Table 2) to be consistently more active than those with phenylene rings (Table 3). A comparison of the function of 14 compounds (**8b**, **8e**, **9b**, **10b**, **10d**, **11b**, **11e**, **12b**, **12d**, **14d**, **15b**, **15d**, **16b** and **16d**) in Table 2 with matching analogues (**8a**, **8d**, **9a**, **10a**, **10c**, **11a**, **11d**, **12a**, **12c**, **14c**, **17a**, **17c**, **19a** and **19b**) in Table 3 showed that on average, structures with a phenyl side chains were 8 times more active than those with the phenylene (Figure 3C,D). The same trend was observed with compounds in Table 4. The negative contribution of the double bond may be due to restriction of its rotation, which prevents a better fit in the binding pocket, although this was not discernable from the 3D-QSAR maps (Figure 3 and Figure 4).

The addition of dimethyl groups in the cyclohexane-1,3-dione ring adversely affected activity. Pairwise comparison of the methylated structures to their unmethylated counterparts in Table 1, Table 2, Table 3 and Table 4 revealed that methyl groups reduced activity 4.5 times (with the exception of **15b** and **15d**). We have previously reported that the volume of the binding domain surrounding the ring is restricted [43], which may account for the negative contribution of a methyl group in that position (Figure 4). Similarly, molecules with a hydroxy group on the cyclohexane ring (**1h**, **3d** and **4d** in Table 1) were on average 3.7 times less active than those without hydroxy groups (**1f**, **3b** and **4b** in Table 1) (Figure 4).

**Figure 5:**
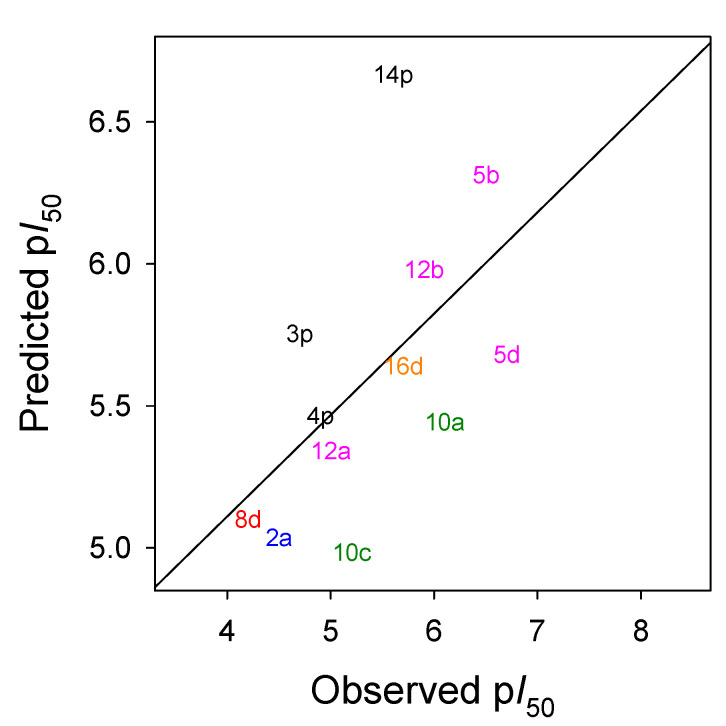
Model predicting activity of the compounds not included in the training set by plotting the relationship between observed and predicted values from the cross-validated leave-one-out partial-least-squares (PLS) analysis. Colors correspond to those used in Table 1, Table 2, Table 3 and Table 4. R ^2^ = 0.96.

Within each series of 2-acylcyclohexane-1,3-diones tested in this study, general structure–activity relationships emerged. The generated model was robust, with R^2^ = 0.96 and Q^2^ = 0.69 (Figure S5–S7). Electronic and steric features that can increase or decrease the activities of 2-acyl-cyclohexane-1,3-diones derivatives that were identified by the model. Figure 5 shows the validation of the test set in which a reasonable linear adjustment and the 3D-QSAR model were able to predict and differentiate the most active compounds among the derivatives. Therefore, in addition to synthesizing and characterizing a broad range of novel triketone compounds, we further identified characteristics of the ligands that contribute to inhibitory function against HPPD, which were incorporated into a model that can be used to predict activity and target the future synthesis of new triketone-based herbicides.

### 2.4. In Silico Analysis of Compound Interactions with HPPD

**Figure 6:**
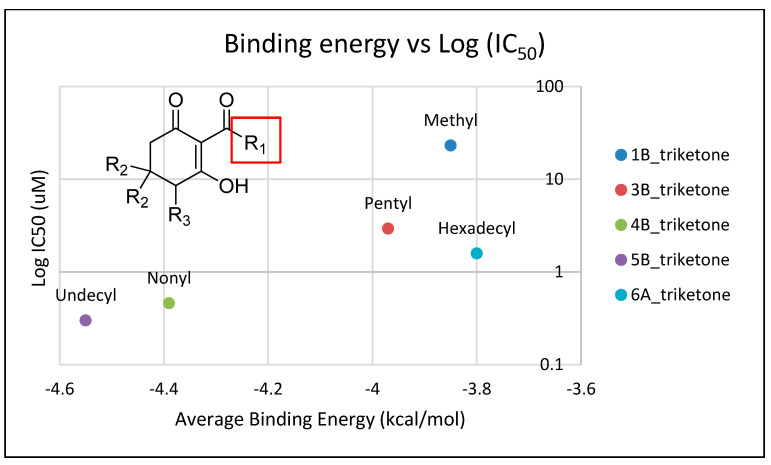
The relationship of binding energy and the log*I_50_* of a selection of 2-acyl-cyclohexane-1,3-diones with simple aliphatic side chains (R_1_ subgroup; indicated by a red square).

In silico docking of structures in the binding pocket of Arabidopsis HPPD led to the identification of key structural features that contribute to inhibition of the enzyme. Structures **1b**, **3b**, **4b**, **5b** and **6a** vary only in the length of hydrophobic tails at the position indicated by R_1_ (Figure 6). There is a positive correlation between log*I_50_* and respective average binding energies up to an R_1_ length of 11 carbons (**5b**), with a noted decrease in activity at an R_1_ of 16 carbons (**6a**). The longer than optimal carbon tail of **6a** likely results in it not being able to fit sterically sound into the hydrophobic pocket at the binding site. However, having a tail length of fewer than optimal carbons results in not fully utilizing the space and the stabilizing effect of the same binding domain. A similar trend has been observed in a related series of triketone HPPD inhibitors containing quinazoline-2,4 dione derivatives [44]. Both cases indicate that flexible, nonpolar alkyl tail groups increase HPPD inhibition up to an optimal length resulting from the size of the hydrophobic HPPD binding pocket.

**Figure 7:**
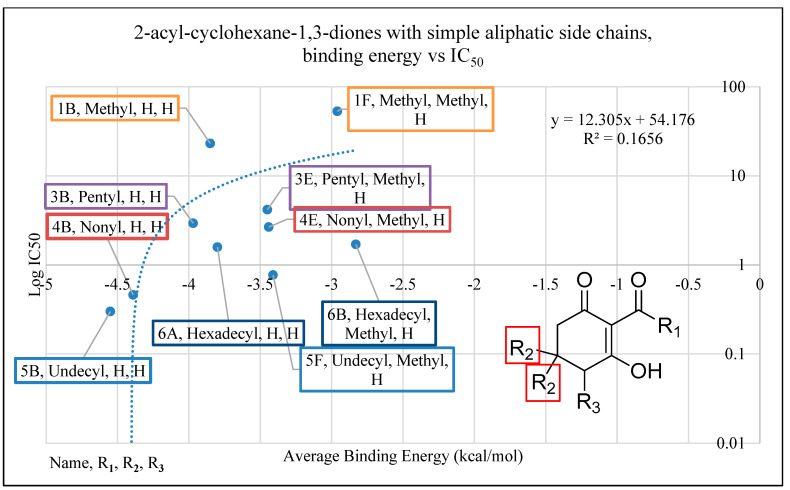
The relationship of binding energy and the log*I_50_* of a selection of 2-acyl-cyclohexane-1,3-diones with simple aliphatic side chains. Molecules are organized according to class of base structure (Grouping), seen on the bottom right. Each data label follows the pattern of “Name, Grouping, R_1_, R_2_, R_3_”, where “R_1_, R_2_ and R_3_” refer to the functional group. Each grouping of similar tail length is shown by a colored box, with the only difference within the color selection being an addition of a head group at the R_2_ location (as indicated by the red square).

**Figure 8:**
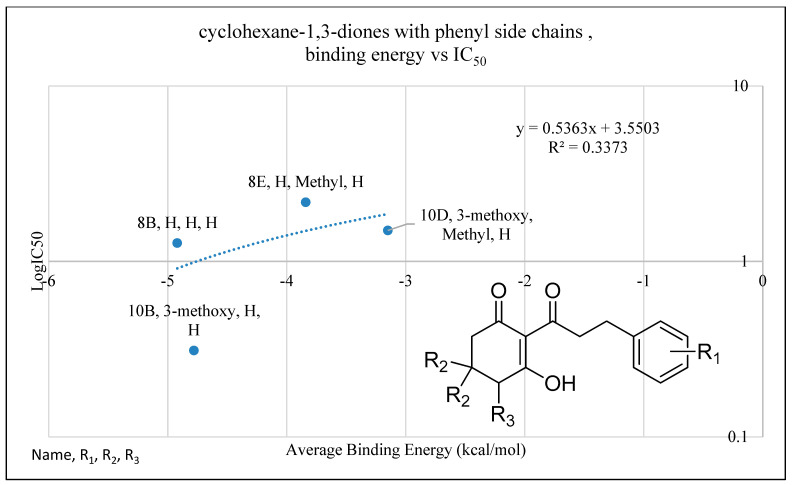
The relationship of binding energy and the log*I_50_* of a selection of cyclohexane-1,3-diones with phenyl side chains. Molecules are organized according to class of base structure (Grouping), seen on the bottom right. Each data label follows the pattern of “Name, Grouping, R_1_, R_2_, R_3_”, where “R_1_, R_2_ and R_3_” refer to the functional group.

Another trend indicated by docking studies is that the addition of methyl moieties to the base ring structure at the positions indicated by R_2_ negatively impacts I_50_app values. This is apparent in comparisons of **1f**, **3e**, **4e**, **5f** and **6b** to their related carbon length counterparts **1b**, **3b**, **4b**, **5b** and **6a** (Figure 7). This trend is further observed when comparing cyclohexane-1,3-diones with phenylene side chains (**8a** vs. **8d**, **10a** vs. **10c**), cyclohexane-1,3-diones with phenyl side chains (**8b** vs. **8e**, **10b** vs. **10d**) and in cyclohexane-1,3-diones with other side chains (**13a** vs. **13c**) (Figure 8). However, an interesting comparison comes into play when factoring in a polar tail group (3-methoxy addition to R_1_) in the phenylene side chains (**8A** vs. **10A**), the phenyl side chains (**8B** vs. **10B**) and the other side chain (benzodioxole group) (**13A** vs. **13C**). The 3-methoxy group is an electron-donating group via resonance, known as the mesomeric effect, with the excess electron lone pairs donated to the ring, increasing reactivity [45]. This can then allow electrons to be shared in conjunction with the tautomerization of the triketone structure [9], as we see a greater decrease in I_50_app in the phenylene sidechain as compared to the phenyl sidechain. Both the 3-methoxy group and the benzodioxole group have oxygens attached to a benzene ring that would allow them to assist in forming pie bonds, with benzodioxole having an anomeric effect due to its carbon and oxygen confirmation causing ring pucker [46]. This would potentially allow for the large bulky groups to be stabilized with conserved amino acid residues Phe360 and Phe403 via *π*-*π* stacking [44]. This addition of a charged functionality increased the polarity of the dione group through the mesomeric effect and tautomerization of the triketone and led to the larger I_50_app value difference observed between the phenylene side chain, which has an extra moveable double bond, and the phenyl side chain, which does not have electron flexibility. The addition of a methyl group to the triketone head group (R_2_) with the methoxy group on R_1_ resulted in the I_50_app returning close to original values (without the methyl head on R_2_ or the methoxy on R_1_), following the trend established previously that adding a methyl group to the base ring structure results in a decreased binding energy and I_50_app_._

## 3. Conclusions

The most effective inhibitor of HPPD has an R1 with an undecyl 11-C chain, which is the optimal number of carbons to fully utilize the binding pocket. This was indicated by binding energy, IC50 values and QSAR analysis. This class, along with cyclohexane-1,3-diones with phenyl side chains, phenylene side chains, or other side chains additionally showed a trend in which the addition of a R2 methyl group had a more positive binding energy and a less-efficient IC50 value, possibly due to stearic hindrance. As such, this study has synthesized and characterized a broad range of novel triketone compounds, where the identified characteristics of the ligands that contribute to inhibitory function against HPPD were incorporated into a protein–ligand docking model that can be used to predict HPPD inhibitory activity and allow the targeted synthesis of new triketone-based herbicides.

## 4. Materials and Methods

### 4.1. General Experimental Conditions

Reactions were carried out under an atmosphere of nitrogen in oven-dried glassware with magnetic stirring and dry solvents under anhydrous conditions unless otherwise indicated. Dichloromethane was purified by passage through a bed of activated alumina. All other reagents and solvents were purchased from Sigma-Aldrich and used without further purification. Reactions were controlled by analytical thin-layer chromatography using Merck precoated silica gel plates with F254 indicator and visualization by UV light (254 nm). Yields refer to chromatographically and spectroscopically pure compounds, unless otherwise indicated. Purification by column chromatography was carried out using Merck silica gel Si 60 (0.040–0.063). ^1^H NMR and ^13^C NMR were recorded on a Bruker DPX 300 (300 MHz ^1^H NMR, 75 MHz ^13^C NMR), or a Bruker DPX 500 (500 MHz ^1^H NMR, 125 MHz ^13^C NMR) instrument (Figures S8–S159). Chemical shift values (δ) are reported in ppm (residual chloroform δ = 7.26 ppm for ^1^H, residual chloroform δ = 77.16 ppm for ^13^C). Proton spectra are reported according to δ (multiplicity, coupling constant *J*, number of protons, assignment). Multiplicities are indicated by s (singlet), d (doublet), dd (double doublets), ddd (double double doublets), t (triplet), q (quartet), quint (quintet), sext (sextuplet), m (multiplet) (Supplemental NMR data). The carbon spectra are reported according to δ (assignment). Infrared spectra were recorded with a Perkin Elmer-Frontier FT-IR apparatus. High-resolution mass spectra were recorded with a MicroTOFQ-II (Bruker, Billerica, MA, USA) apparatus equipped with a positive ESI source (Supplemental Spectroscopic Data).

### 4.2. Synthesis of Saturated 2-Acyl-cyclohexane-1,3-diones

The acid derivative (2.00 mmol), dicyclohexylcarbodiimide (2.40 mmol), triethylamine (2.40 mmol) and 4-dimethylaminopyridine (0.20 mmol) were added successively to a solution of 1,3-cyclohexanedione or 5,5-dimethyl-1,3-cyclohexanedione derivatives (2.00 mmol) in dichloromethane (40 mL) and the reaction mixture was stirred 24 h [47]. After 24 h, the mixture was diluted with dichloromethane and paper-filtered, and 20 mL of 1 M HCl was added to the filtrate. The aqueous phase was extracted by ether (3 × 20 mL). The organic extract was washed with brine, dried over sodium sulfate and concentrated. The residue was purified by flash chromatography (7:3 hexanes/ethyl acetate) or by recrystallization with methanol.

### 4.3. Synthesis of Unsaturated 2-Acyl-cyclohexane-1,3-diones (Supplemental Schemes S2 and S3)

2-Acyl-cyclohexane-1,3-dione (1a, 1d or 1e), 2.0 mmol, was dissolved in 25 mL of toluene, 2.2 mmol of the corresponding aromatic aldehyde and 0.6 mol of secondary amine were added and the mixture was heated for 12 h under reflux in a flask equipped with a Dean–Stark trap [48]. After 12 h, the mixture was cooled, diluted with 30 mL of toluene, transferred into a separatory funnel and extracted with 20 mL of 20% HCl (in one portion). The organic solution was dried over anhydrous sodium sulfate, filtered through a 1 cm layer of silica gel and concentrated. The residues were purified by flash chromatography (6:2:2 hexanes/ethyl acetate/dichloromethane).

### 4.4. Hydrogenation of Unsaturated 2-Acyl-cyclohexane-1,3-diones (Supplemental Scheme S4)

Compounds **15a**, **15c**, **16a** and **16c** (0.5 mmol) were dissolved in MeOH (8 mL) and the solutions were shaken in a Parr apparatus under a hydrogen atmosphere (4 atm) for 4 h. The reaction mixtures were filtered with a 0.45 µm PTFE membrane filter and the filtrates were concentrated to give the products (**15b**, **15d**, **16b** and **16d**) without further purification.

### 4.5. Synthesis of Hydroxylated 2-Acyl-Cyclohexane-1,3-diones (Supplemental Scheme S5)

#### 4.5.1. Protection Step

Hydroxylamine hydrochloride (8 mmol) and sodium acetate (4.1 mmol) were added to a solution of 2-acyl-1,3-cyclohexane-1,3-diones (4 mmol) in H_2_O (30 mL) and ethanol (60 mL) [43]. The reaction mixture was heated on reflux for 18 h, cooled and the solvent evaporated. The residue was purified by flash chromatography (6:2:2 hexanes/ethyl acetate/dichloromethane) to afford pure isoxazoline derivatives. 

#### 4.5.2. Oxidation Step

KOH (35 mmol) in dry MeOH (20 mL) was stirred until dissolved, cooled to −15 °C and a solution of the isoxazoline derivative (2 mmol) in MeOH (2 mL) was added [49]. After 15 min, PhI(OAc)_2_ (3.5 mmol) was added in one portion. The reaction mixture was stirred at −15 °C for 1 h and at room temperature for 2 h, then concentrated under reduced pressure to give the crude hydroxy-dimethylacetals. Crude products were dissolved in dichloromethane and treated with 10% sulfuric acid (98%) for 10 min. After neutralization with saturated aq. NaHCO_3_ and extraction with ether, the combined organic layers were dried and the solvent was removed under vacuum. Final purification was carried out by circular chromatography using a chromatotron (4:1 hexanes/ethyl acetate) to afford hydroxylated isoxazolines. 

#### 4.5.3. Hydrogenation Step

After three vacuum/H_2_ cycles to remove air from the reaction flask, the stirred mixture of hydroxylated isoxazoline derivative (1 mmol), 10% Pd/C in MeOH (10 mL) was hydrogenated at ambient pressure and room temperature for 4 h. The reaction mixture was filtered using a 0.45 µm membrane.

#### 4.5.4. Imine Hydrolysis Step

The crude product from the preview step, after filtration, was treated with 5 mL of NaOH 1 M [50]. After 4 h at room temperature, the solution was treated with a few drops of concentrated HOAc, extracted with dichloromethane and the combined organic layers were dried via anhydrous sodium sulfate, paper-filtered and evaporated under vacuum. The residue was purified by flash chromatography (6:2:2 hexane/ethyl acetate/dichloromethane).

### 4.6. Recombinant Expression of HPPD and Enzyme Assays

HPPD from *Arabidopsis thaliana* was recombinantly overexpressed in *E. coli* and tested in total soluble protein (TSP) extracts. Enzyme activity was measured as described previously [10,51]. The HPLC system used to quantify enzyme activity was composed of a Waters Corporation system (Milford, MA) including a Model 600E pump, Model 717 autosampler, Millenium 2010 controller and Model 996 photodiode detector equipped with a 7.8 mm × 100 mm X-Terra C18 (5 µm) reversed-phase column. Solvent A was 0.1% (*v/v*) trifluoroacetic acid in ddH_2_O and solvent B was 0.08% (*v/v*) trifluoroacetic acid in 80% (*v/v*) HPLC-grade acetonitrile. The solvent system consisted of a linear gradient at 0% (100% A) to 70% B from 0 to 2 min, 70% to 100% B from 2 to 4 min, 100% B from 4 to 6 min, 100% to 0% B from 6 to 7 min and 0% B from 7 to 8 min. The flow rate was 3 mL m ^−1^ and the injection volume was 100 µL. HGA was detected by UV absorbance at 288 nm [52]. A calibration curve was established by injecting various known concentrations of HGA. 

### 4.7. Dose–Response Curve Analysis

Data from dose–response experiments were analyzed using the dose–response curve module [53] of R version 2.2.1, R Core Team, Cambridge, MA, USA). [54] *I*_50_app means and standard deviations, obtained using the untransformed data, are given in Table 1, Table 2, Table 3 and Table 4. The synthetic HPPD inhibitor sulcotrione was included as a positive control [10].

### 4.8. Characterization of the Mechanism of HPPD Binding

HPPD activity was monitored via oxygen consumption during the course of the reaction by an adapted version of a procedure described previously [55]. In short, a 3 mL reaction contained 0.2 M sodium phosphate buffer (pH 7.2) equilibrated at 37 °C, 1.8 mM ascorbic acid, 250 μM HPP, 3.75 μM inhibitor and varying amounts of recombinant enzyme. For control reactions, an equivalent volume of methanol was substituted in place of the inhibitor. The recombinant enzyme was diluted with extraction buffer (20 mM potassium phosphate (pH 6.8), 1 mM EDTA, 1 mM DTT, 1 mM 6-aminohexanoic (aminocaproic) acid, 1 mM benzamidine) to maintain the overall volume at 70 μL in all assays. Components were combined in the reaction vessel of an Oxygraph Plus (Hansatech, Norfolk, UK) oxygen monitoring system, which was maintained at 37 °C by a circulating water bath and allowed to equilibrate for 1 min. Reactions were initiated by the addition of HPP and allowed to settle for 0.5 min, and dissolved oxygen levels were continuously monitored for a further 1.5 min. Oxygen consumption rates were then determined from the slope of the line between 30 s and 60 s of the resulting graphs using O_2_ View software (version 2.05). All reactions were performed in triplicate.

### 4.9. Molecular Modeling

The physicochemical contribution of a series of cyclohexane-1,3-diones on the activity of HPPD was investigated using a structure–activity approach. The structures of 59 cyclohexane-1,3-dione analogs were drawn using Marvin 14.9.8.0, 2014, ChemAxon (http://www.chemaxon.com (accessed on 14 March 2022). Standardizer, JChem 14.9.8.0, 2014, ChemAxon (http://www.chemaxon.com (accessed on 14 March 2022)), was used to canonize the structures by converting an arbitrarily chosen chemical structure to a unique notation, adding hydrogen atoms and cleaning the molecular graph in three dimensions. The process uses a divide-and-conquer approach, whereby the structure is split into small fragments that are organized into a tree from connectivity information using a proprietary extended version of the Dreiding force field [37]. Conformers generated for the initial structure (represented by the root node in the tree) are subsequently optimized. 

Geometry optimizations and conformational searches were performed using Spartan [56]. The geometry of the chemical structure of the compound was initially optimized with a Merck Molecular Force Field (MMFF) [57] and a new geometric optimization was then performed based on the semi-empirical method, Austin Model 1 (AM1) [58]. A systematic search method was used in which the 10 conformers with the lowest minimum energy were selected from an analyzed 10,000 conformers using AM1 and a Monte Carlo algorithm [59]. Dihedrals were evaluated by rotation in accordance with the default conditions of the program, and again the conformers with the lowest minimum energy were selected and optimized based on a vibrational mode calculation using AM1 [58].

### 4.10. Molecular Docking

Autodock version 4.2.6 was employed for all molecular docking experiments. An Arabidopsis HPPD protein crystal structure (PDB: 1SP9) was converted into a .pdbqt file based upon the crystal structure of the protein, with a 0.9 charge on the FE atom ligand in the active site. Ligands were uploaded into Autodock from Avogadro as mol2 files. Torsion of the ligand and conversion into a .pdbqt file were performed within Autodock. The grid utilized for docking used the covalent map around the coordinates x = 24.063, y = 9.506, z = −19.126 of the HPPD protein with an energy barrier of 1000, half-width to 5 and a gridbox of dimensions x = 75, y = 75, z = 75, spacing = 0.2, centered over the binding domain/active site at coordinates x = 25, y = 5, z = −19. Docking parameters with the HPPD protein and the ligand were the “created ligands” from Avogadro, with default search parameters except for genetic algorithm changed to 25 runs. These docking parameters were utilized to simulate interactions and the resultant binding energies.

### 4.11. 3D-QSAR

FLAP 2.1.0 software [60] was used to perform all the analyses described hereafter. The structures of cyclohexane-1,3-dione analogues and the respective values of p*I*_50_ (−log*I*_50_) were used as input data. Structures were aligned using the option “bondType_atomNum” in order that the maximal subgraph required equivalent bond types and atom numbers to match and that the number of conformers was 50 using the MM3 forcefield [61]. Using descriptors generated by the Molecular Interaction Fields (MIF) with a GRID resolution of 0.75 Å, 3D-QSAR was performed. Probe H (shape probe) and probe N1 (amide nitrogen), which represent hydrogen-bonding donor groups and interact with the hydrogen-bonding acceptors fragments of the ligand, and probe O (carbonyl oxygen), which represents hydrogen-bonding acceptor groups and interact with the hydrogen-bonding donor fragments of the ligand, were used. The data were split into a training set with 57 compounds (82.6% of dataset) and a test set with 12 triketone analogues, which represented 17.4% of the entire dataset. The last set was built by randomly selecting compounds, taking into account the structural diversity and range of p *I*_50_ values of the training set. Finally, the model’s PLS (partial least squares) was generated to 8 latent variables (LV) [62]. The final model and the number of LVs were selected by the highest value of cross-validated (leave-one-out) coefficient of determination. 

## Figures and Tables

**Table 1:**
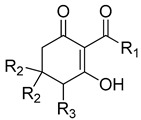
Structures and activities of 19 2-acyl-cyclohexane-1,3-diones with simple aliphatic side chains tested in this study.

Cpd #	R_1_	R_2_	R_3_	mw	*I*_50_ (μM) ^ab^
**1b** ^c^	methyl	H	H	154	23.09 ± 4.34
**1d**	methyl	H	OH	170	>1000
**1f**	methyl	methyl	H	182	53.12 ± 17.09
**1h**	methyl	methyl	OH	198	166.03 ± 19.66
**2a**	propyl	H	H	182	35.12 ± 6.24
**2b**	propyl	methyl	H	210	16.59 ± 4.36
**3b**	pentyl	H	H	210	2.93 ± 0.47
**3d**	pentyl	H	OH	226	15.84 ± 4.9
**3e**	pentyl	methyl	H	238	4.17 ± 0.45
**4b**	nonyl	H	H	266	0.46 ± 0.06
**4d**	nonyl	H	OH	282	1.25 ± 0.40
**4e**	nonyl	methyl	H	294	2.66 ± 0.95
**5b**	undecyl	H	H	294	0.30 ± 0.03
**5d**	undecyl	H	OH	310	0.18 ± 0.02
**5f**	undecyl	methyl	H	322	0.77 ± 0.11
**6a**	hexadecyl	H	H	364	1.58 ± 0.20
**6b**	hexadecyl	methyl	H	392	1.70 ± 0.31
**7a**	4-oxopentyl	H	H	224	9.6 ± 1.4
**7b**	4-oxopentyl	methyl	H	252	17.2 ± 2.6

^a^ The data represent means followed by standard error, n = 3. ^b^ The commercial product sulcotrione has an *I*_50_app: 0.25 ± 0.02 μM. ^c^ The rows are color-coded to match Figure 3.

**Table 2:**
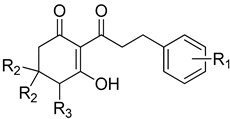
Structures and activities of 17 cyclohexane-1,3-diones with phenyl side chains tested in this study.

Cpd #	R_1_	R_2_	R_3_	mw	*I*_50_ (μM) ^ab^
**8b** ^c^	H	H	H	244	1.27 ± 0.09
**8e**	H	methyl	H	272	2.17 ± 0.37
**9b**	2-methyl	H	H	258	0.97 ± 0.09
**9d**	2-methyl	methyl	H	286	nd ^d^
**10b**	3-methoxy	H	H	274	0.31 ± 0.04
**10d**	3-methoxy	methyl	H	302	1.50 ± 0.17
**11b**	4-methoxy	H	H	274	2.36 ± 0.30
**11c**	4-methoxy	H	OH	290	59.5 ± 11.8
**11e**	4-methoxy	methyl	H	302	12.9 ± 2.18
**12b**	3,4-dimethoxy	H	H	304	1.35 ± 0.18
**12d**	3,4-dimethoxy	methyl	H	332	8.69 ± 1.32
**14b**	3,4,5-trimethoxy	H	H	334	nd
**14d**	3,4,5-trimethoxy	methyl	H	362	2.29 ± 0.25
**15b**	4-dimethylamino	H	H	287	4.47 ± 0.51
**15d**	4-dimethylamino	methyl	H	315	3.46 ± 0.64
**16b**	4-chloro	H	H	278	0.24 ± 0.02
**16d**	4-chloro	methyl	H	307	2.05 ± 0.19

^a^ The data represent means followed by standard error, n = 3. ^b^ The commercial product sulcotrione has an *I*_50_app: 0.25 ± 0.02 μM. ^c^ The rows are color-coded to match Figure 3. ^d^ nd = data could not be obtained because the retention time of the inhibitor overlapped with that of HGA.

**Table 3:**
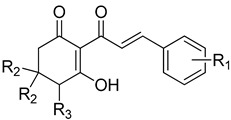
Structures and activities of 24 cyclohexane-1,3-diones with phenylene side chains tested in this study.

Cpd #	R_1_	R_2_	R_3_	mw	*I*_50_ (μM) ^ab^
**8a** ^c^	H	H	H	242	5.43 ± 0.58
**8c**	H	H	OH	258	59.0 ± 3.16
**8d**	H	methyl	H	270	58.6 ± 8.67
**9a**	2-methyl	H	H	256	1.20 ± 0.14
**9c**	2-methyl	methyl	H	284	28.7 ± 8.7
**10a**	3-methoxy	H	H	272	0.89 ± 0.10
**10c**	3-methoxy	methyl	H	300	6.4 ± 1.2
**11a**	4-methoxy	H	H	272	12.3 ± 1.6
**11d**	4-methoxy	methyl	H	300	141.4 ± 27
**12a**	3,4-dimethoxy	H	H	302	9.16 ± 1.21
**12c**	3,4-dimethoxy	methyl	H	330	14.1 ± 2.21
**14a**	3,4,5-trimethoxy	H	H	332	2.06 ± 0.13
**14c**	3,4,5-trimethoxy	methyl	H	360	2.22 ± 0.50
**15a**	4-dimethylamino	H	H	285	nd ^d^
**15c**	4-dimethylamino	methyl	H	313	nd
**16a**	4-chloro	H	H	276	nd
**16c**	4-chloro	methyl	H	305	nd
**17a**	4-bromo	H	H	320	7.55 ± 0.93
**17b**	4-bromo	H	OH	336	28.7 ± 4.82
**17c**	4-bromo	methyl	H	349	8.34 ± 1.57
**18a**	3-bromo	H	H	321	0.82 ± 0.08
**18b**	3-bromo	methyl	H	349	2.42 ± 0.25
**19a**	4-nitro	H	H	287	7.61 ± 0.89
**19b**	4-nitro	methyl	H	315	27.5 ± 2.39

^a^ The data represent means followed by standard error, n = 3. ^b^ The commercial product sulcotrione has an *I*_50_app: 0.25 ± 0.02 μM. ^c^ The rows are color-coded to match Figure 3. ^d^ nd = data could not be obtained because the retention time of the inhibitor overlapped with that of HGA.

**Table 4:** Structures and activities of 4 cyclohexane-1,3-diones with other side chains tested in this study.

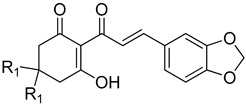			
**Cpd #**	**R_1_**	**mw**	***I*** **_50_** **(μM) ^a,b^**
**13a** ^c^	H	286	9.51 ± 1.84
**13c**	methyl	314	28.7 ± 3.72
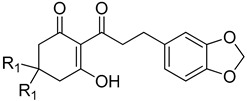			
**Cpd #**	**R_1_**	**mw**	***I*** **_50_** **(μM) ^a,b^**
**13b**	H	288	0.97 ± 0.16
**13d**	methyl	316	3.25 ± 0.60

^a^ The data represent means followed by standard error, n = 3. ^b^ The commercial product sulcotrione has an *I*_50_app: 0.25 ± 0.02 μM. ^c^ The rows are color-coded to match Figure 3.

**Table 5:**
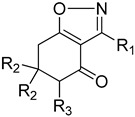
Structures and activities of non-diones with simple aliphatic side chains tested in this study.

Cpd #	R_1_	R_2_	R_3_	mw	*I*_50_ (μM) ^ab^
**1a**	methyl	H	H	151	>100
**1c**	methyl	H	OH	167	>100
**1e**	methyl	methyl	H	179	>100
**1g**	methyl	methyl	OH	195	>100
**3a**	pentyl	H	H	207	>100
**3c**	pentyl	H	OH	223	>100
**4a**	nonyl	H	H	263	>100
**4c**	nonyl	H	OH	279	>100
**5a**	undecyl	H	H	291	>100
**5c**	undecyl	H	OH	307	>100
**5e**	undecyl	methyl	H	319	>100
**5g**	undecyl	methyl	OH	335	>100

^a^ The data represent means, n = 3. ^b^ The commercial product sulcotrione has an *I*_50_app: 0.25 ± 0.02 μM.

## Data Availability

Not applicable.

## Supplementary Materials

The following are available online at https://www.mdpi.com/article/10.3390/plants11172269/s1, Supplemental Scheme S1: Saturated 2-acyl-cyclohexane-1,3-diones; Supplemental Scheme S2 and S3: Unsaturated 2-acyl-cyclohexane-1,3-diones; Supplemental Scheme S4: Hydrogenation of unsaturated 2-acyl-cyclohexane-1,3-diones; Supplemental Scheme S5: Synthesis of hydroxylated 2-acyl-cyclohexane-1,3-diones; Supplemental Figures S8–S159: Spectrometric and Spectropic Data.
