# Supplementary material for: Synthesis and Activity of 2-Acyl-cyclohexane-1,3-dione Congeners Derived from Peperomia Natural Products against the Plant p-Hydroxyphenylpyruvate Dioxygenase Herbicidal Molecular Target Site
Source: Plants (Basel). 2022 Aug 31;11(17):2269. doi: 10.3390/plants11172269 (PMC9459959; doi:10.3390/plants11172269)
Supplement: Supplementary file 1 [file plants-11-02269-s001.zip › plants-1829671-supplementary.pdf]

3D-QSAR Analysis of 2-Acyl-cyclohexane-1,3-diones on Plant *p*-Hydroxyphenylpyruvate Dioxygenase

MAURO V. CORREIA,[1,6] DANIEL K. OWENS,[2] SUSAN B. WATSON,[3] MARCUS T. SCOTTI[4], HAROLD H. FOKOUE,[5,6] LYDIA F. YAMAGUCHI,[6] FRANCK E. DAYAN,[7*] AND MASSUO J. KATO[6*]

[1]*Institute of Chemistry, University of Brasilia, 70704-970, Brasilia, DF, Brazil*

[2]*Molecular Biosciences and Bioengineering, University of Hawaii at Manoa, Honolulu, HI 96822, USA*

[3]*United States Department of Agriculture, Agricultural Research Service, Natural Products Utilization Research Unit, P.O. Box 1848, University, MS 38677, USA*

[4]*Department of Chemistry, Federal University of Paraiba,* 58051-900*, Brazil*

[5]*Laboratório de Avaliação e Síntese de Substâncias Bioativas (LASSBio®), Instituto de Ciências Biomédicas, Centro das Ciências da Saúde, Universidade Federal do Rio de Janeiro. CP 68024, 21944-971, Rio de Janeiro, Brazil*

[6]*Institute of Chemistry, University of São Paulo, 05508-000, São Paulo, SP, Brazil*

[7]*Bioagricultural Sciences and Pest Management, Colorado State University, Fort Collins, CO 80523, USA*

Supplementary material

Synthesis schemes for compounds

Spectroscopic data of compounds

List of Figures

Figure S1. Inhibition of HPPD by compounds from Table 1: **1b** (●), **1f** (■), **1h** (▲), **2a** (●), **2b** (■), **3b** (●), **3d** (■), **3e** (▲), **4b** (●), **4d** (■), **4e** (▲), **5b** (●), **5d** (■), **5f** (▲), **6a** (●), **6b** (■), **7a** (●), and **7b** (■). Each data point represents the mean of three independent experiments ± 1 SD.

Figure S2. Inhibition of HPPD by compounds from Table 2: **8a** (●), **8c** (■), **8d** (▲), **9a** (●), **9c** (■), **10a** (●), **10c** (■), **11a** (●), **11d** (■), **12a** (●), **12c** (■), **14a** (●), **14c** (■), **17a** (●), **17b** (■), **17c** (▲), **18a** (●), **18b** (■), **19a** (●), and **19b** (■).

Figure S3. Inhibition of HPPD by compounds from Table 3: **8b** (●), **8e** (■), **9b** (●), **10b** (●), **10d** (■), **11b** (●), **11c** (■), **11e** (▲), **12b** (●), **12d** (■), **14d** (●), **15b** (●), and **15d** (■), **16b** (●), **16d** (■).

Figure S4. Inhibition of HPPD by compounds from Table 4: **13a** (●), **13c** (■), **13b** (●), **13d** (■).

Figure S5. Plot of R^2 (●) and Q^2 (■) values of the various regressions.

Figure S6. Plot of scores LV1 vs LV2 – The greater and more blue the dots, the more active (higher pIC_{50} values) is the compound.

Figure S7. Plot of loadings LV1 vs LV2.

Figure S8. ^{1}H NMR (500 MHz, $CDCl_3$) spectrum of **1a**.

Figure S9. ^{13}C NMR (125 MHZ, $CDCl_3$) spectrum of **1a**.

Figure S10. ^{1}H NMR (300 MHz, $CDCl_3$) spectrum of **1b**.

Figure S11. ^{13}C NMR (75 MHZ, $CDCl_3$) spectrum of **1b**.

Figure S12. ^{1}H NMR (500 MHz, $CDCl_3$) spectrum of **1c**.

Figure S13. ^{13}C NMR (125 MHZ, $CDCl_3$) spectrum of **1c**.

Figure S14. ^{1}H NMR (500 MHz, $CDCl_3$) spectrum of **1d**.

Figure S15. ^{13}C NMR (125 MHZ, $CDCl_3$) spectrum of **1d**.

Figure S16. ^{1}H NMR (300 MHz, $CDCl_3$) spectrum of **1e**.

Figure S17. ^{13}C NMR (75 MHZ, $CDCl_3$) spectrum of **1e**.

Figure S18. ^{1}H NMR (300 MHz, $CDCl_3$) spectrum of **1f**.

Figure S19. ^{13}C NMR (75 MHZ, $CDCl_3$) spectrum of **1f**.

Figure S20. ^{1}H NMR (300 MHz, $CDCl_3$) spectrum of **1g**.

Figure S21. ^{13}C NMR (75 MHZ, $CDCl_3$) spectrum of **1g**.

Figure S22. ^{1}H NMR (300 MHz, $CDCl_3$) spectrum of **1h**.

Figure S23. ^{13}C NMR (75 MHZ, $CDCl_3$) spectrum of **1h**.

Figure S24. ^{1}H NMR (300 MHz, $CDCl_3$) spectrum of **2a**.

Figure S25. ^{13}C NMR (75 MHZ, $CDCl_3$) spectrum of **2a**.

Figure S26. ^{1}H NMR (300 MHz, $CDCl_3$) spectrum of **2b**.

Figure S27. ^{13}C NMR (75 MHZ, $CDCl_3$) spectrum of **2b**.

Figure S28. ^{1}H NMR (300 MHz, $CDCl_3$) spectrum of **3a**.

Figure S29. ^{13}C NMR (75 MHZ, $CDCl_3$) spectrum of **3a**.

Figure S30. ^{1}H NMR (300 MHz, $CDCl_3$) spectrum of **3b**.

Figure S31. ^{13}C NMR (75 MHZ, $CDCl_3$) spectrum of **3b**.

Figure S32. ^{1}H NMR (300 MHz, $CDCl_3$) spectrum of **3c**.

Figure S33. ^{13}C NMR (75 MHZ, $CDCl_3$) spectrum of **3c**.

Figure S34. ^{1}H NMR (300 MHz, $CDCl_3$) spectrum of **3d**.

Figure S35. ^{13}C NMR (75 MHZ, $CDCl_3$) spectrum of **3d**.

Figure S36. ^{1}H NMR (300 MHz, $CDCl_3$) spectrum of **3e**.

Figure S37. ^{13}C NMR (75 MHZ, $CDCl_3$) spectrum of **3e**.

Figure S38. ^{1}H NMR (300 MHz, $CDCl_3$) spectrum of **4a**.

Figure S39. ^{13}C NMR (75 MHZ, $CDCl_3$) spectrum of **4a**.

Figure S40. ^{1}H NMR (300 MHz, $CDCl_3$) spectrum of **4b**.

Figure S41. ^{13}C NMR (75 MHZ, $CDCl_3$) spectrum of **4b**.

Figure S42. ^{1}H NMR (300 MHz, $CDCl_3$) spectrum of **4c**.

Figure S43. ^{13}C NMR (75 MHZ, $CDCl_3$) spectrum of **4c**.

Figure S44. ^{1}H NMR (300 MHz, $CDCl_3$) spectrum of **4d**.

Figure S45. ^{13}C NMR (75 MHZ, $CDCl_3$) spectrum of **4d**.

Figure S46. ^{1}H NMR (300 MHz, $CDCl_3$) spectrum of **4e**.

Figure S47. ^{13}C NMR (75 MHZ, $CDCl_3$) spectrum of **4e**.

Figure S48. ^{1}H NMR (300 MHz, $CDCl_3$) spectrum of **5a**.

Figure S49. ^{13}C NMR (75 MHZ, $CDCl_3$) spectrum of **5a**.

Figure S50. ^{1}H NMR (300 MHz, $CDCl_3$) spectrum of **5b**.

Figure S51. ^{13}C NMR (75 MHZ, $CDCl_3$) spectrum of **5b**.

Figure S52. ^{1}H NMR (300 MHz, $CDCl_3$) spectrum of **5c**.

Figure S53. ^{13}C NMR (75 MHZ, $CDCl_3$) spectrum of **5c**.

Figure S54. ^{1}H NMR (300 MHz, $CDCl_3$) spectrum of **5d**.

Figure S55. ^{13}C NMR (75 MHZ, $CDCl_3$) spectrum of **5d**.

Figure S56. ^{1}H NMR (300 MHz, $CDCl_3$) spectrum of **5e**.

Figure S57. ^{13}C NMR (75 MHZ, $CDCl_3$) spectrum of **5e**.

Figure S58. ^{1}H NMR (300 MHz, $CDCl_3$) spectrum of **5f**.

Figure S59. ^{13}C NMR (75 MHZ, $CDCl_3$) spectrum of **5f**.

Figure S60. ^{1}H NMR (300 MHz, $CDCl_3$) spectrum of **5g**.

Figure S61. ^{13}C NMR (75 MHZ, $CDCl_3$) spectrum of **5g**.

Figure S62. ^{1}H NMR (300 MHz, $CDCl_3$) spectrum of **6a**.

Figure S63. ^{13}C NMR (75 MHZ, $CDCl_3$) spectrum of **6a**.

Figure S64. ^{1}H NMR (300 MHz, $CDCl_3$) spectrum of **6b**.

Figure S65. ^{13}C NMR (75 MHZ, $CDCl_3$) spectrum of **6b**.

Figure S66. ^{1}H NMR (300 MHz, $CDCl_3$) spectrum of **7a**.

Figure S67. ^{13}C NMR (75 MHZ, $CDCl_3$) spectrum of **7a**.

Figure S68. ^{1}H NMR (300 MHz, $CDCl_3$) spectrum of **7b**.

Figure S69. ^{13}C NMR (75 MHZ, $CDCl_3$) spectrum of **7b**.

Figure S70. ^{1}H NMR (300 MHz, $CDCl_3$) spectrum of **8a**.

Figure S71. ^{13}C NMR (75 MHZ, $CDCl_3$) spectrum of **8a**.

Figure S72. ^{1}H NMR (300 MHz, $CDCl_3$) spectrum of **8b**.

Figure S73. ^{13}C NMR (75 MHZ, $CDCl_3$) spectrum of **8b**.

Figure S74. ^{1}H NMR (300 MHz, $CDCl_3$) spectrum of **8c**.

Figure S75. ^{13}C NMR (75 MHZ, $CDCl_3$) spectrum of **8c**.

Figure S76. ^{1}H NMR (300 MHz, $CDCl_3$) spectrum of **8d**.

Figure S77. ^{13}C NMR (75 MHZ, $CDCl_3$) spectrum of **8d**.

Figure S78. ^{1}H NMR (300 MHz, $CDCl_3$) spectrum of **8e**.

Figure S79. ^{13}C NMR (75 MHZ, $CDCl_3$) spectrum of **8e**.

Figure S80. ^{1}H NMR (300 MHz, $CDCl_3$) spectrum of **9a**.

Figure S81. ^{13}C NMR (75 MHZ, $CDCl_3$) spectrum of **9a**.

Figure S82. ^{1}H NMR (300 MHz, $CDCl_3$) spectrum of **9b**.

Figure S83. ^{13}C NMR (75 MHZ, $CDCl_3$) spectrum of **9b**.

Figure S84. ^{1}H NMR (300 MHz, $CDCl_3$) spectrum of **9c**.

Figure S85. ^{13}C NMR (75 MHZ, $CDCl_3$) spectrum of **9c**.

Figure S86. ^{1}H NMR (300 MHz, $CDCl_3$) spectrum of **9d**.

Figure S87. ^{13}C NMR (75 MHZ, $CDCl_3$) spectrum of **9d**.

Figure S88. ^{1}H NMR (300 MHz, $CDCl_3$) spectrum of **10a**.

Figure S89. ^{13}C NMR (75 MHZ, $CDCl_3$) spectrum of **10a**.

Figure S90. ^{1}H NMR (300 MHz, $CDCl_3$) spectrum of **10b**.

Figure S91. ^{13}C NMR (75 MHZ, $CDCl_3$) spectrum of **10b**.

Figure S92. ^{1}H NMR (300 MHz, $CDCl_3$) spectrum of **10c**.

Figure S93. ^{13}C NMR (75 MHZ, $CDCl_3$) spectrum of **10c**.

Figure S94. ^{1}H NMR (300 MHz, $CDCl_3$) spectrum of **10d**.

Figure S95. ^{13}C NMR (75 MHZ, $CDCl_3$) spectrum of **10d**.

Figure S96. ^{1}H NMR (300 MHz, $CDCl_3$) spectrum of **11a**.

Figure S97. ^{13}C NMR (75 MHZ, $CDCl_3$) spectrum of **11a**.

Figure S98. ^{1}H NMR (300 MHz, $CDCl_3$) spectrum of **11b**.

Figure S99. ^{13}C NMR (75 MHZ, $CDCl_3$) spectrum of **11b**.

Figure S100. ^{1}H NMR (300 MHz, $CDCl_3$) spectrum of **11c**.

Figure S101. ^{13}C NMR (75 MHZ, $CDCl_3$) spectrum of **11c**.

Figure S102. ^{1}H NMR (300 MHz, $CDCl_3$) spectrum of **11d**.

Figure S103. ^{13}C NMR (75 MHZ, $CDCl_3$) spectrum of **11d**.

Figure S104. ^{1}H NMR (300 MHz, $CDCl_3$) spectrum of **11e**.

Figure S105. ^{13}C NMR (75 MHZ, $CDCl_3$) spectrum of **11e**.

Figure S106. ^{1}H NMR (300 MHz, $CDCl_3$) spectrum of **12a**.

Figure S107. ^{13}C NMR (75 MHZ, $CDCl_3$) spectrum of **12a**.

Figure S108. ^{1}H NMR (300 MHz, $CDCl_3$) spectrum of **12b**.

Figure S109. ^{13}C NMR (75 MHZ, $CDCl_3$) spectrum of **12b**.

Figure S110. ^{1}H NMR (300 MHz, $CDCl_3$) spectrum of **12c**.

Figure S111. ^{13}C NMR (75 MHZ, $CDCl_3$) spectrum of **12c**.

Figure S112. ^{1}H NMR (300 MHz, $CDCl_3$) spectrum of **12d**.

Figure S113. ^{13}C NMR (75 MHZ, $CDCl_3$) spectrum of **12d**.

Figure S114. ^{1}H NMR (300 MHz, $CDCl_3$) spectrum of **13a**.

Figure S115. ^{13}C NMR (75 MHZ, $CDCl_3$) spectrum of **13a**.

Figure S116. ^{1}H NMR (300 MHz, $CDCl_3$) spectrum of **13b**.

Figure S117. ^{13}C NMR (75 MHZ, $CDCl_3$) spectrum of **13b**.

Figure S118. ^{1}H NMR (300 MHz, $CDCl_3$) spectrum of **13c**.

Figure S119. ^{13}C NMR (75 MHZ, $CDCl_3$) spectrum of **13c**.

Figure S120. ^{1}H NMR (300 MHz, $CDCl_3$) spectrum of **13d**.

Figure S121. ^{13}C NMR (75 MHZ, $CDCl_3$) spectrum of **13d**.

Figure S122. ^{1}H NMR (300 MHz, $CDCl_3$) spectrum of **14a**.

Figure S123. ^{13}C NMR (75 MHZ, $CDCl_3$) spectrum of **14a**.

Figure S124. ^{1}H NMR (300 MHz, $CDCl_3$) spectrum of **14b**.

Figure S125. ^{13}C NMR (75 MHZ, $CDCl_3$) spectrum of **14b**.

Figure S126. ^{1}H NMR (300 MHz, $CDCl_3$) spectrum of **14c**.

Figure S127. ^{13}C NMR (75 MHZ, $CDCl_3$) spectrum of **14c**.

Figure S128. ^{1}H NMR (500 MHz, $CDCl_3$) spectrum of **14d**.

Figure S129. ^{13}C NMR (75 MHZ, $CDCl_3$) spectrum of **14d**.

Figure S130. ^{1}H NMR (300 MHz, $CDCl_3$) spectrum of **15a**.

Figure S131. ^{13}C NMR (75 MHZ, $CDCl_3$) spectrum of **15a**.

Figure S132. ^{1}H NMR (300 MHz, $CDCl_3$) spectrum of **15b**.

Figure S133. ^{13}C NMR (75 MHZ, $CDCl_3$) spectrum of **15b**.

Figure S134. ^{1}H NMR (300 MHz, $CDCl_3$) spectrum of **15c**.

Figure S135. ^{13}C NMR (75 MHZ, $CDCl_3$) spectrum of **15c**.

Figure S136. ^{1}H NMR (300 MHz, $CDCl_3$) spectrum of **15d**.

Figure S137. ^{13}C NMR (75 MHZ, $CDCl_3$) spectrum of **15d**.

Figure S138. ^{1}H NMR (300 MHz, $CDCl_3$) spectrum of **16a**.

Figure S139. ^{13}C NMR (75 MHZ, $CDCl_3$) spectrum of **16a**.

Figure S140. ^{1}H NMR (300 MHz, $CDCl_3$) spectrum of **16b**.

Figure S141. ^{13}C NMR (75 MHZ, $CDCl_3$) spectrum of **16b**.

Figure S142. ^{1}H NMR (300 MHz, $CDCl_3$) spectrum of **16c**.

Figure S143. ^{13}C NMR (75 MHZ, $CDCl_3$) spectrum of **16c**.

Figure S144. ^{1}H NMR (300 MHz, $CDCl_3$) spectrum of **16d**.

Figure S145. ^{13}C NMR (75 MHZ, $CDCl_3$) spectrum of **16d**.

Figure S146. ^{1}H NMR (300 MHz, $CDCl_3$) spectrum of **17a**.

Figure S147. ^{13}C NMR (75 MHZ, $CDCl_3$) spectrum of **17a**.

Figure S148. ^{1}H NMR (300 MHz, $CDCl_3$) spectrum of **17b**.

Figure S149. ^{13}C NMR (75 MHZ, $CDCl_3$) spectrum of **17b**.

Figure S150. ^{1}H NMR (300 MHz, $CDCl_3$) spectrum of **17c**.

Figure S151. ^{13}C NMR (75 MHZ, $CDCl_3$) spectrum of **17c**.

Figure S152. ^{1}H NMR (300 MHz, $CDCl_3$) spectrum of **18a**.

Figure S153. ^{13}C NMR (75 MHZ, $CDCl_3$) spectrum of **18a**.

Figure S154. ^{1}H NMR (300 MHz, $CDCl_3$) spectrum of **18b**.

Figure S155. ^{13}C NMR (75 MHZ, $CDCl_3$) spectrum of **18b**.

Figure S156. ^{1}H NMR (300 MHz, $CDCl_3$) spectrum of **19a**.

Figure S157. ^{13}C NMR (75 MHZ, $CDCl_3$) spectrum of **19a**.

Figure S158. ^{1}H NMR (300 MHz, $CDCl_3$) spectrum of **19b**.

Figure S159. ^{13}C NMR (75 MHZ, $CDCl_3$) spectrum of **19b**.

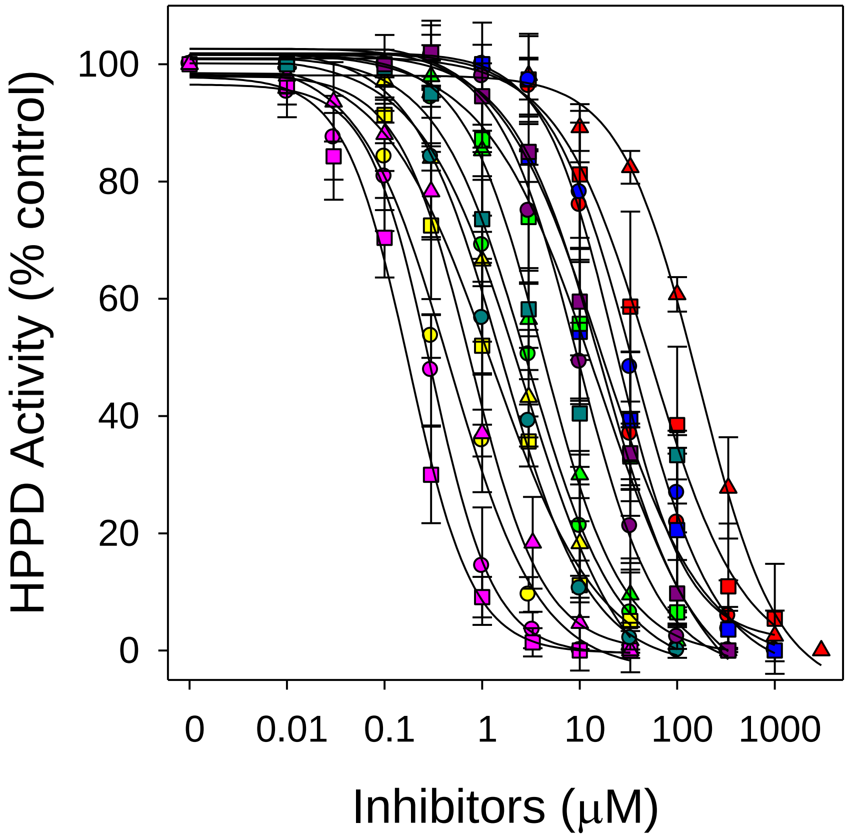

Figure S1. Inhibition of HPPD by compounds from Table 1: **1b** (●), **1f** (■), **1h** (▲), **2a** (●), **2b** (■), **3b** (●), **3d** (■), **3e** (▲), **4b** (●), **4d** (■), **4e** (▲), **5b** (●), **5d** (■), **5f** (▲), **6a** (●), **6b** (■), **7a** (●), and **7b** (■). Each data point represents the mean of three independent experiments ± 1 SD.

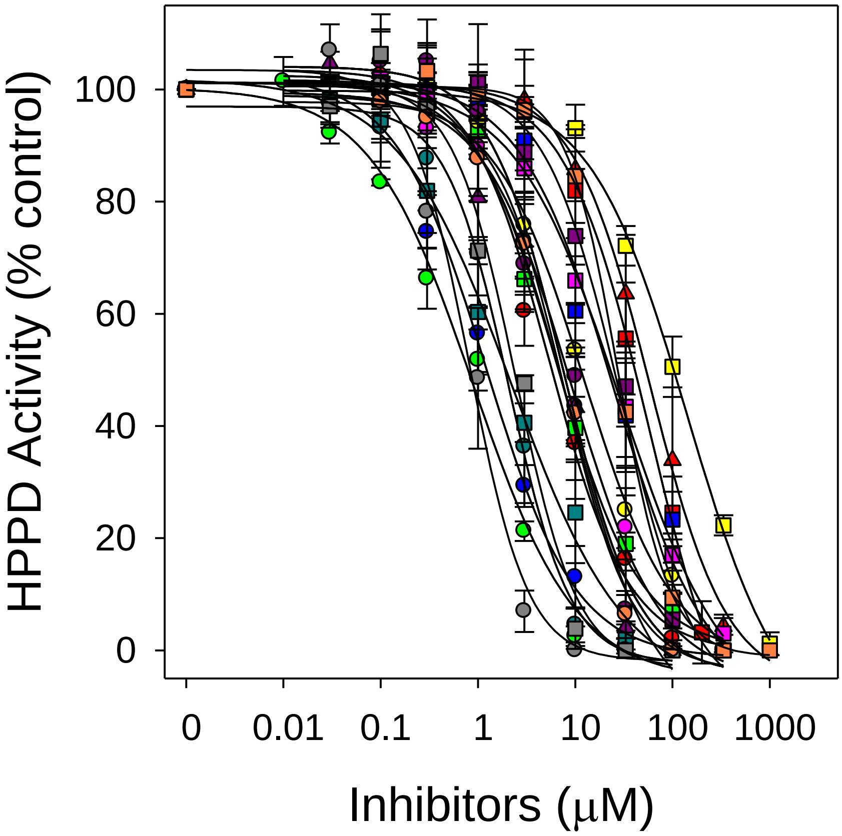

Figure S2. Inhibition of HPPD by compounds from Table 2: **8a** (●), **8c** (■), **8d** (▲), **9a** (●), **9c** (■), **10a** (●), **10c** (■), **11a** (●), **11d** (■), **12a** (●), **12c** (■), **14a** (●), **14c** (■), **17a** (●), **17b** (■), **17c** (▲), **18a** (●), **18b** (■), **19a** (●), and **19b** (■). Each data point represents the mean of three independent experiments ± 1 SD. The curves for compound **15a, 15c, 16a** and **16c** are not shown because their minimal values did not reach less than 30% inhibition.

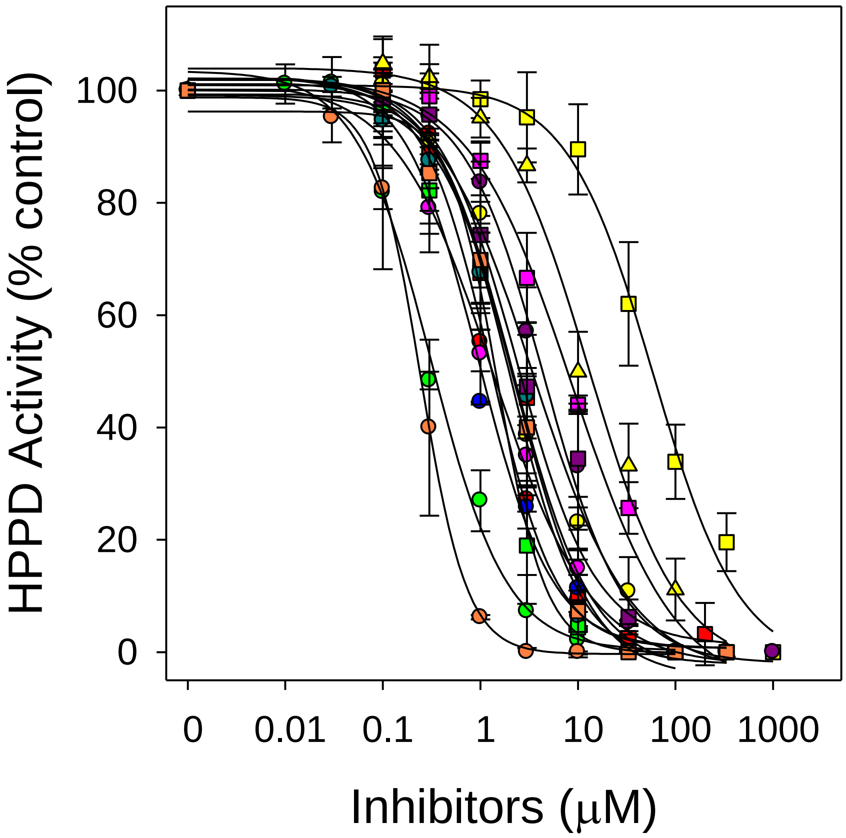

Figure S3. Inhibition of HPPD by compounds from Table 3: **8b** (●), **8e** (■), **9b** (●), **10b** (●), **10d** (■), **11b** (●), **11c** (■), **11e** (▲), **12b** (●), **12d** (■), **14d** (●), **15b** (●), and **15d** (■), **16b** (●), **16d** (■). Each data point represents the mean of three independent experiments ± 1 SD.

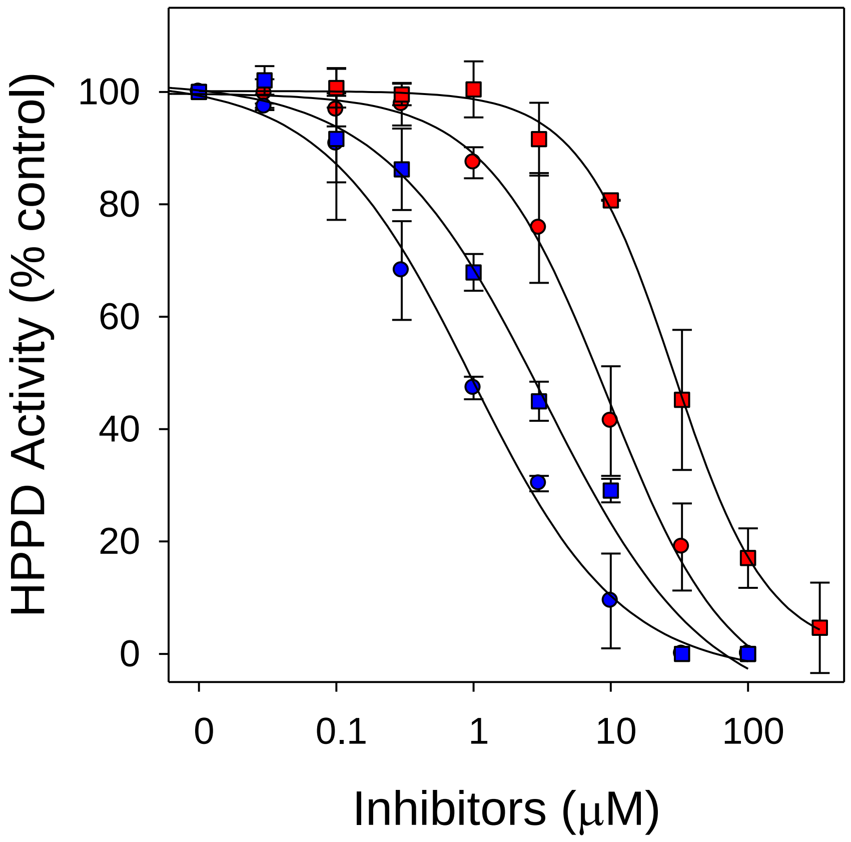

Figure S4. Inhibition of HPPD by compounds from Table 4: **13a** (●), **13c** (■), **13b** (●), **13d** (■). Each data point represents the mean of three independent experiments ± 1 SD.

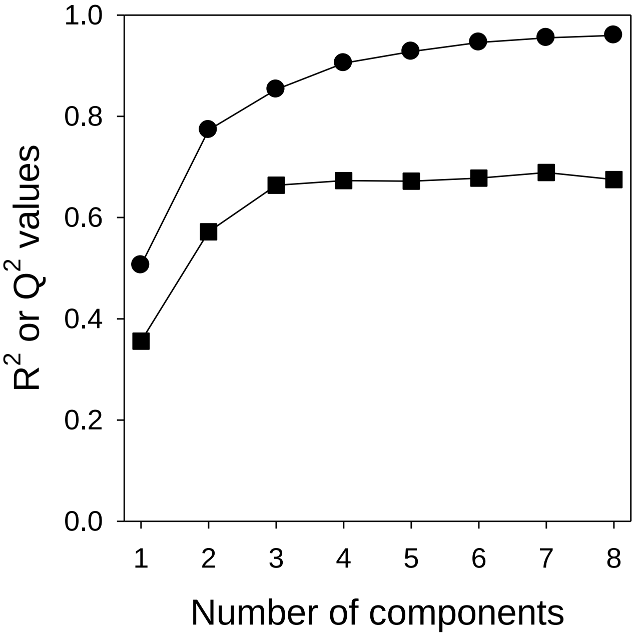

Figure S5. Plot of R^2 (●) and Q^2 (■) values of the various regressions. The selected 3LV were R^2 = 0.96 and Q^2 = 0.69

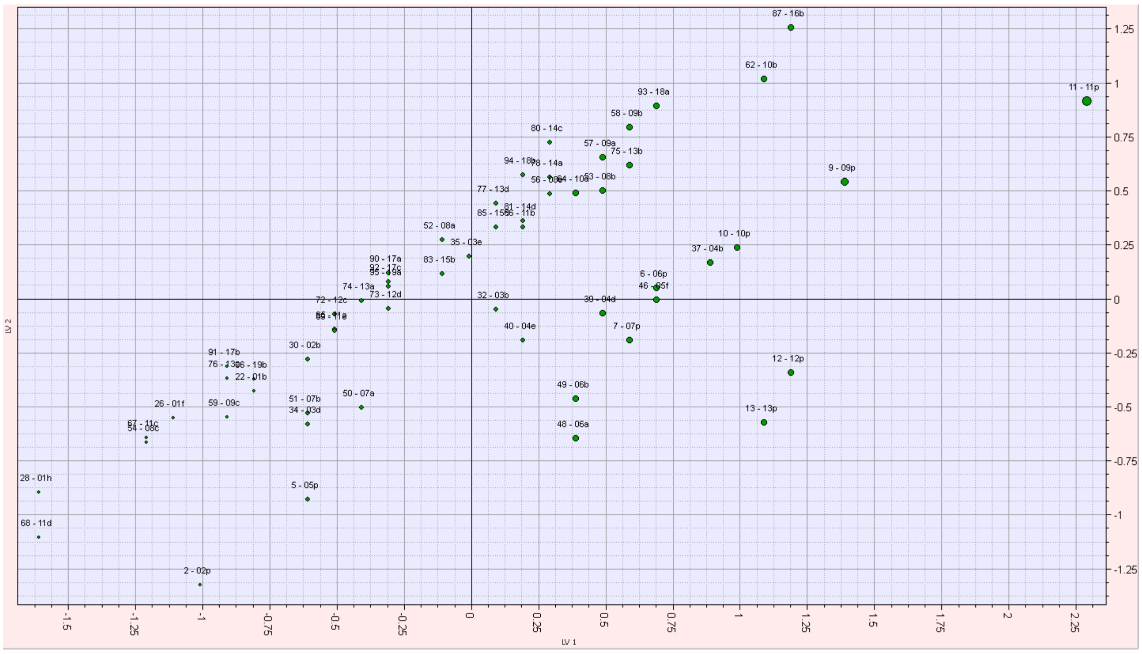

Figure S6. Plot of scores LV1 vs LV2 – The greater and more blue the dots, the more active (higher pIC_{50} values) is the compound.

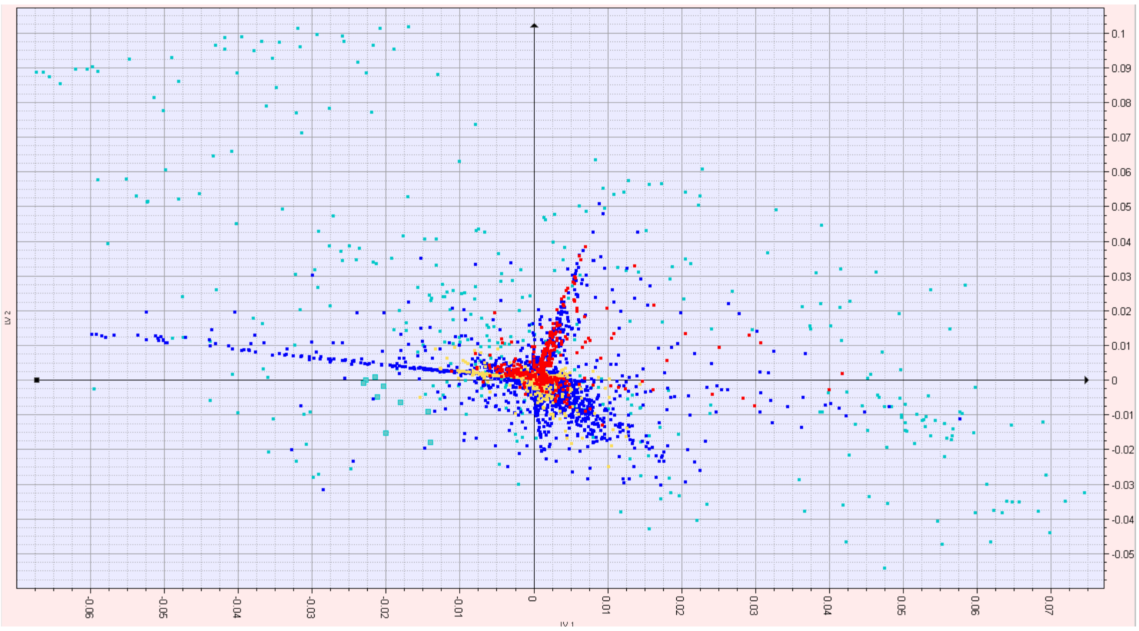

Figure S7. Plot of loadings LV1 vs LV2 – Blue are the variables generated by the amide nitrogen probe (hydrogen bond donor), red are the variables generated by the carbonyl oxygen probe (hydrogen bond acceptor), yellow are variables generated by the hydrophobic probe and cyan are variables generated by hydrogen probe, which describes the shape of the interaction.

Supplemental NMR data.

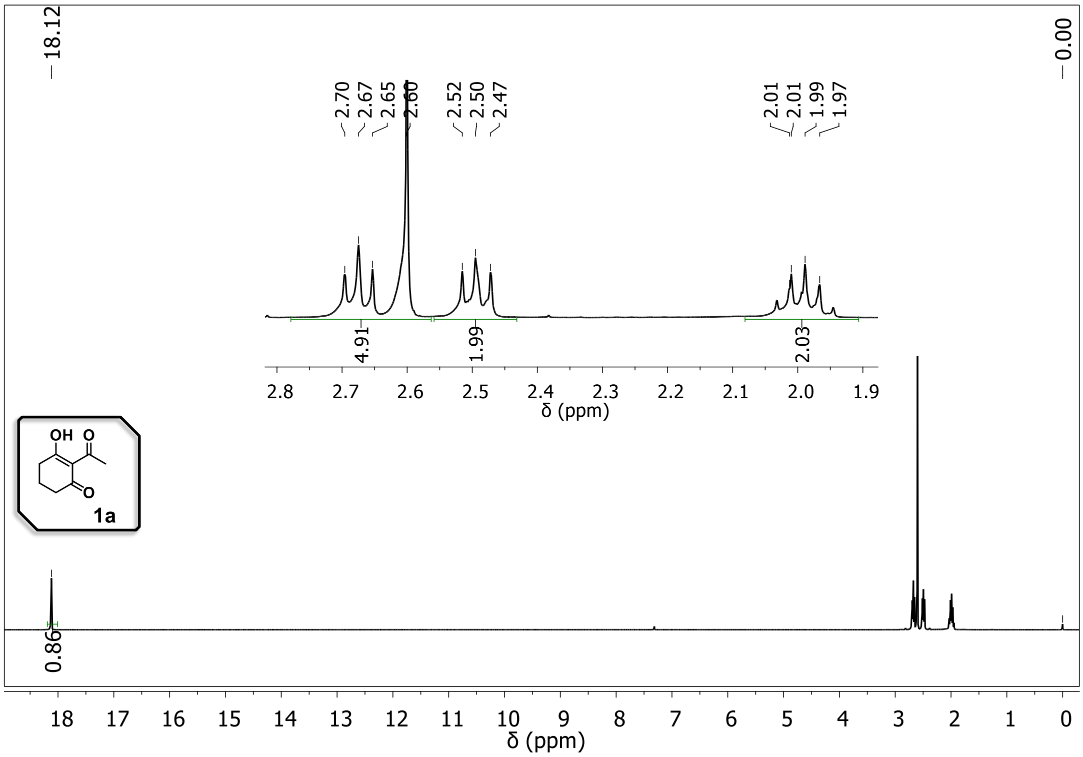

Figure S8. ^{1}H NMR (500 MHz, $CDCl_3$) spectrum of **1a**.

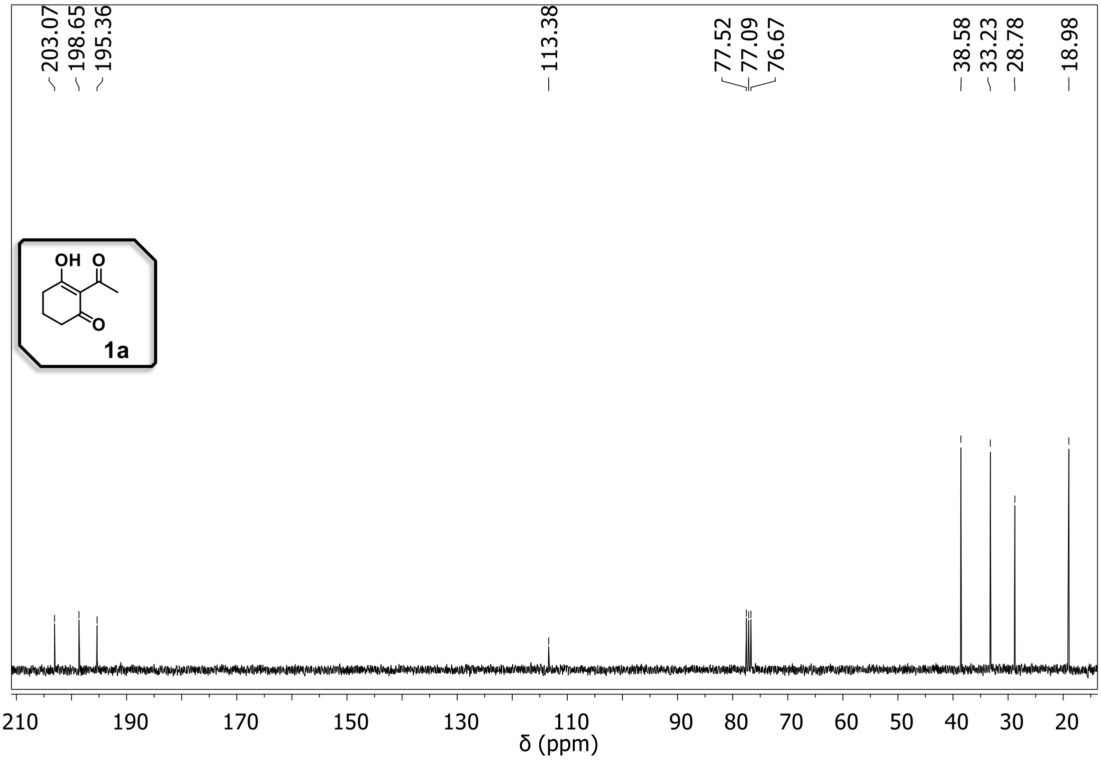

Figure S9. ^{13}C NMR (125 MHZ, $CDCl_3$) spectrum of **1a**.

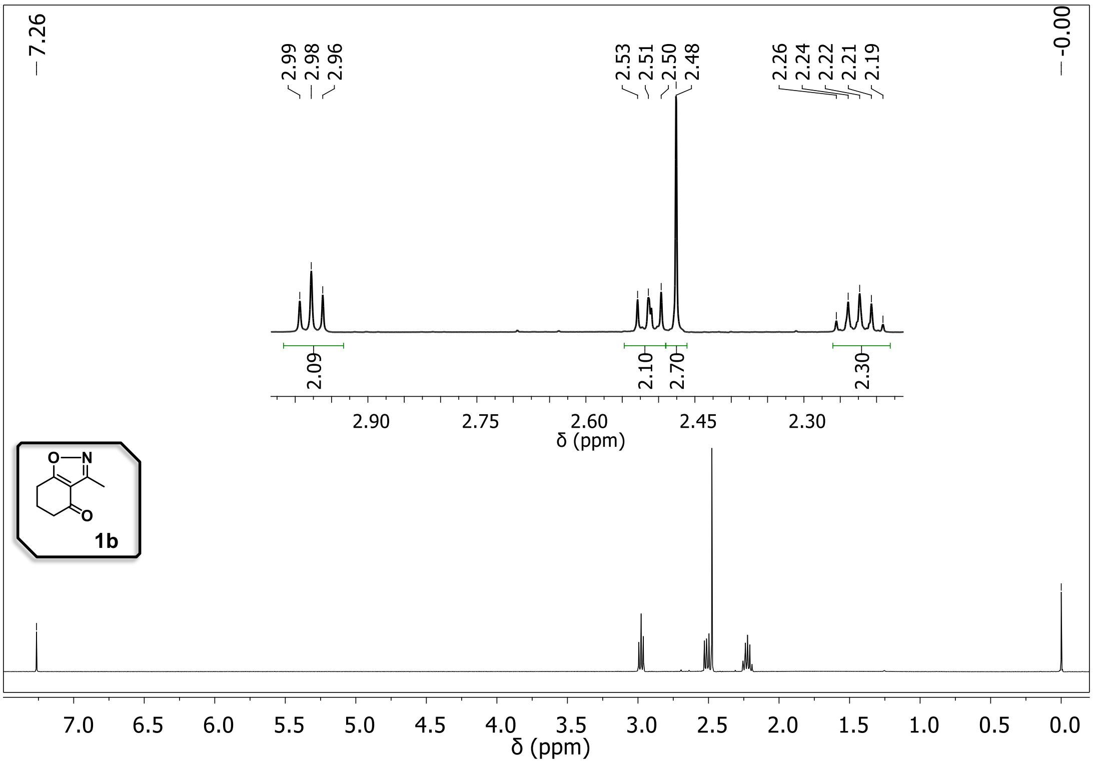

Figure S10. ^{1}H NMR (300 MHz, $CDCl_3$) spectrum of **1b**.

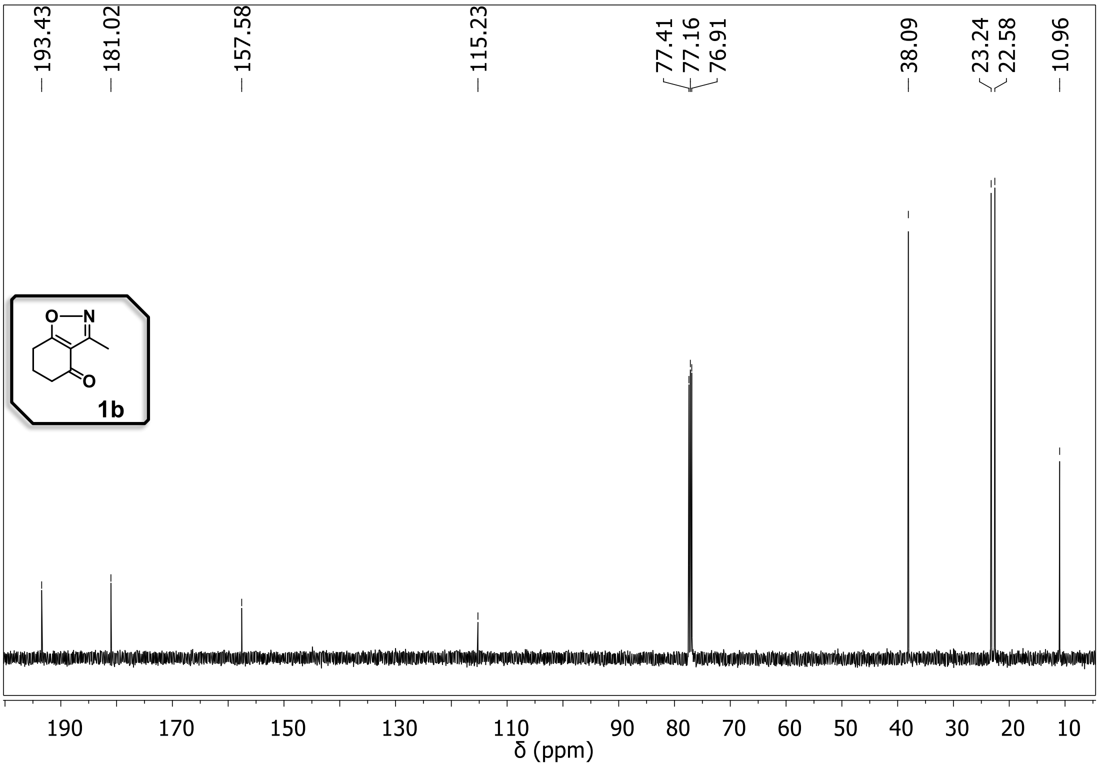

Figure S11. ^{13}C NMR (75 MHZ, $CDCl_3$) spectrum of **1b**.

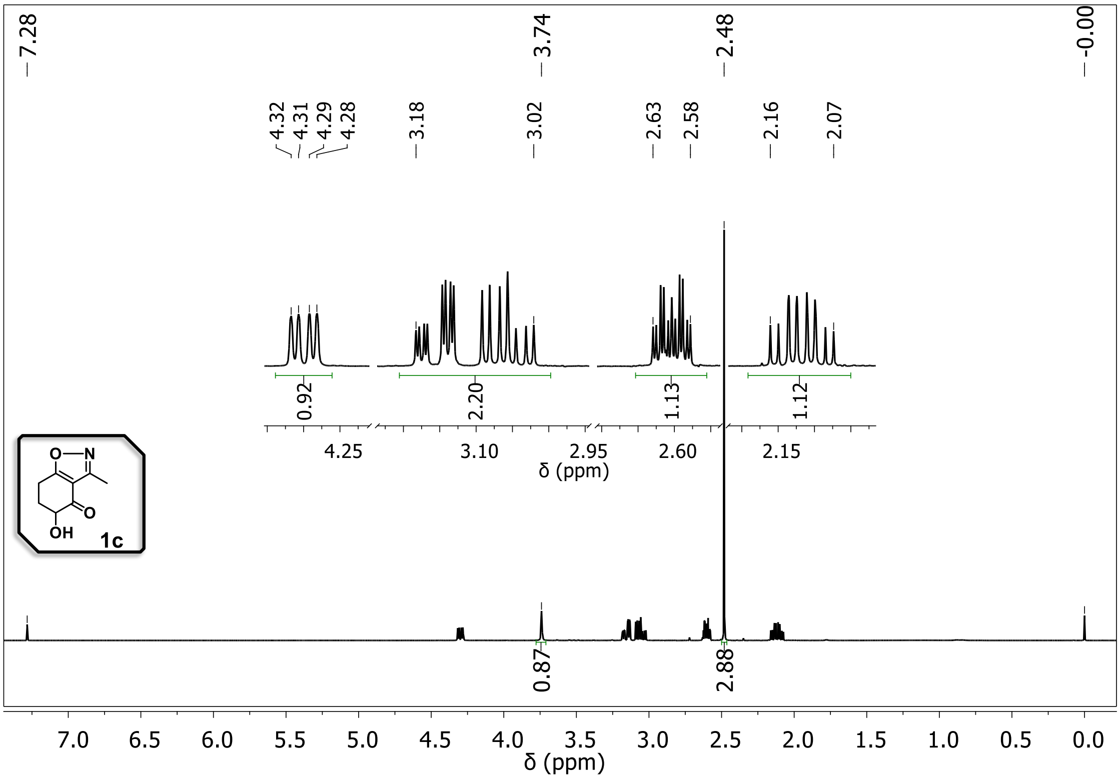

Figure S12. ^{1}H NMR (500 MHz, $CDCl_3$) spectrum of **1c**.

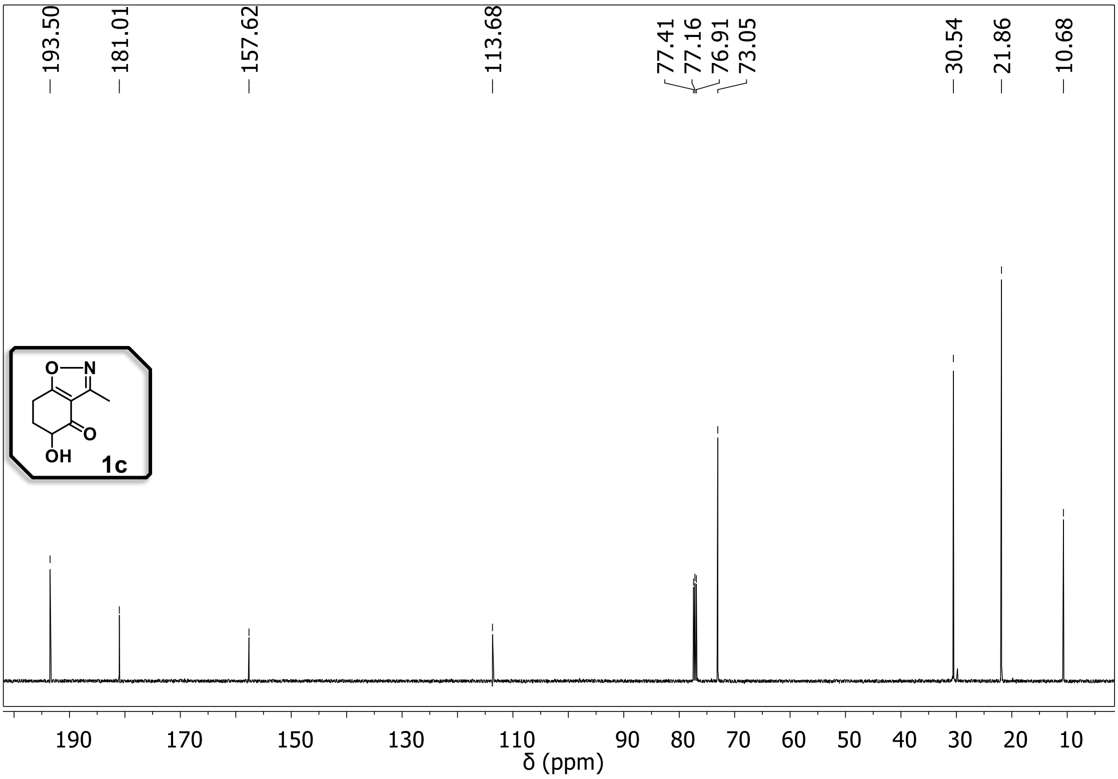

Figure S13. ^{13}C NMR (125 MHZ, $CDCl_3$) spectrum of **1c**.

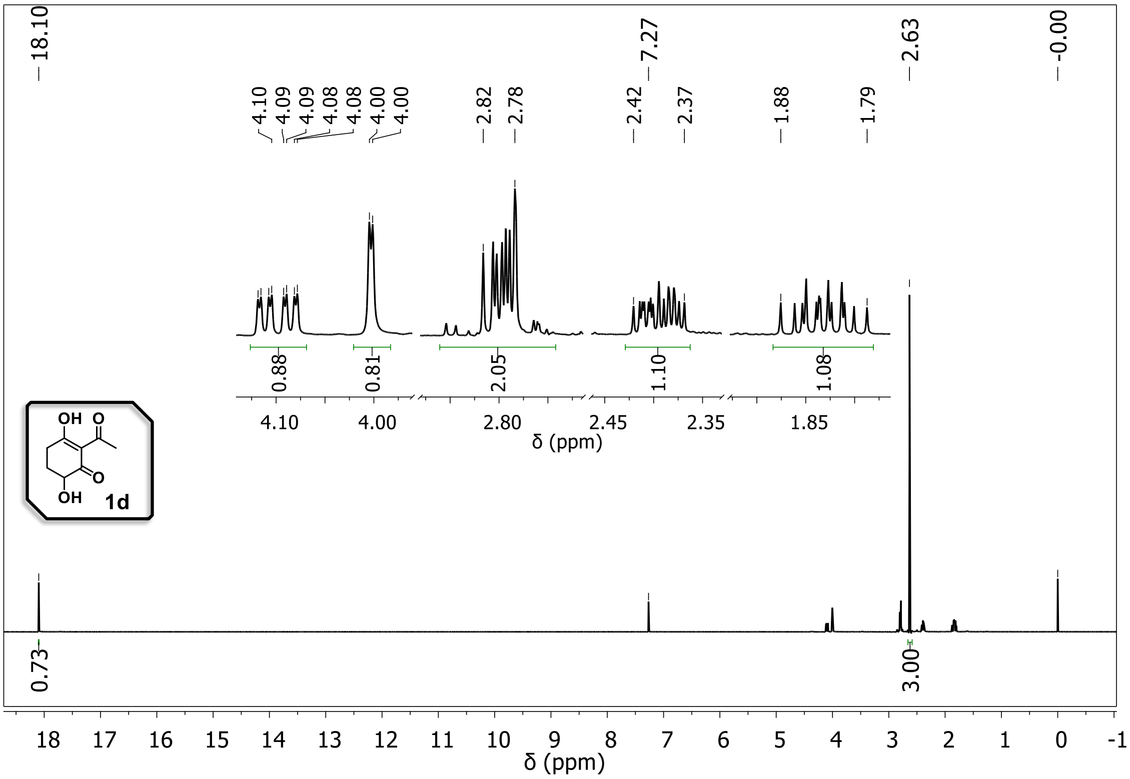

Figure S14. ^{1}H NMR (500 MHz, $CDCl_3$) spectrum of **1d**.

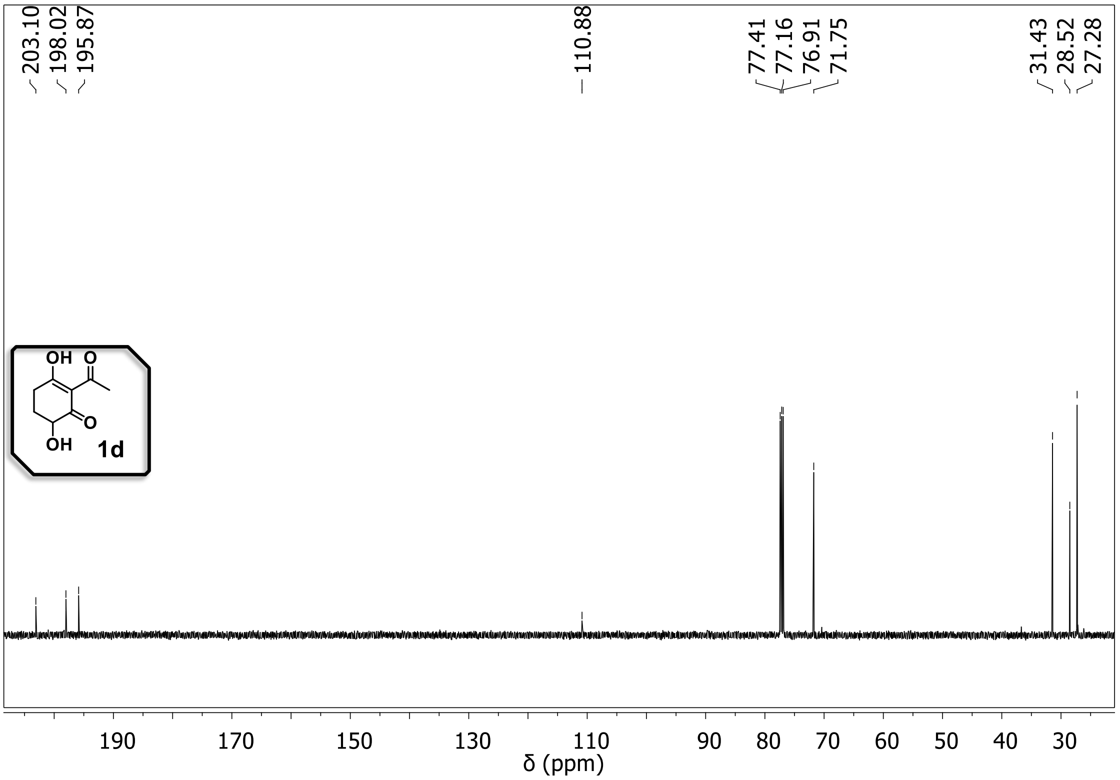

Figure S15. ^{13}C NMR (125 MHZ, $CDCl_3$) spectrum of **1d**.

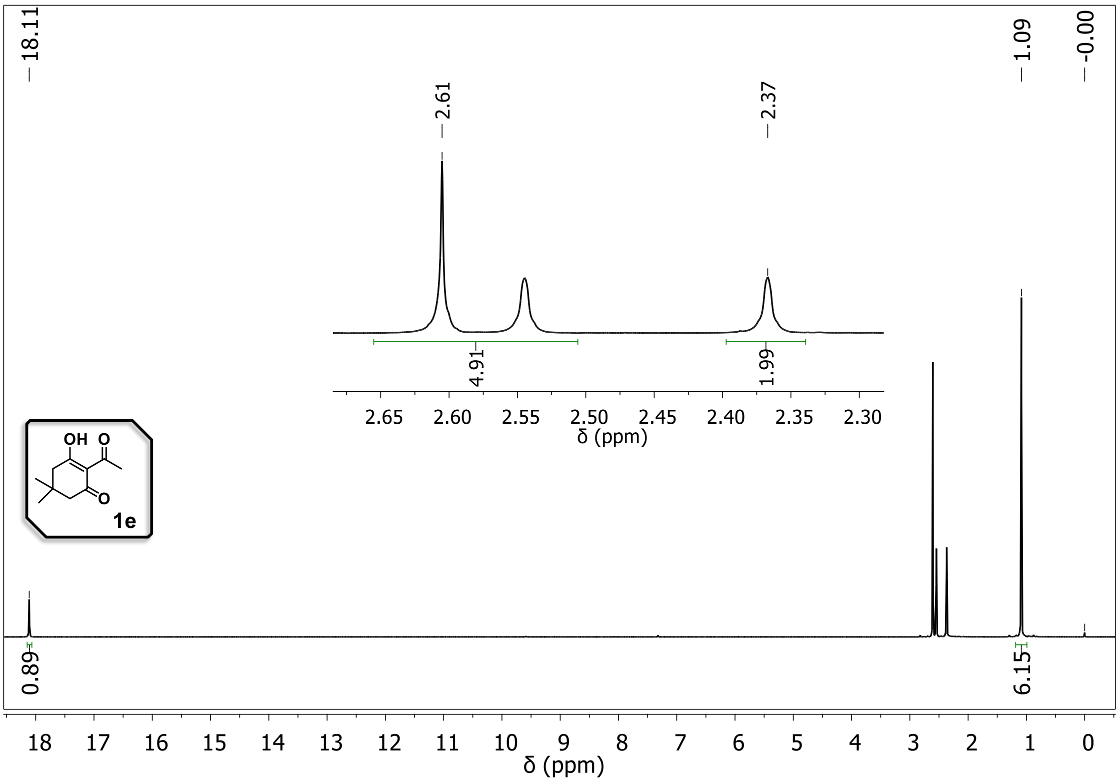

Figure S16. ^{1}H NMR (300 MHz, $CDCl_3$) spectrum of **1e**.

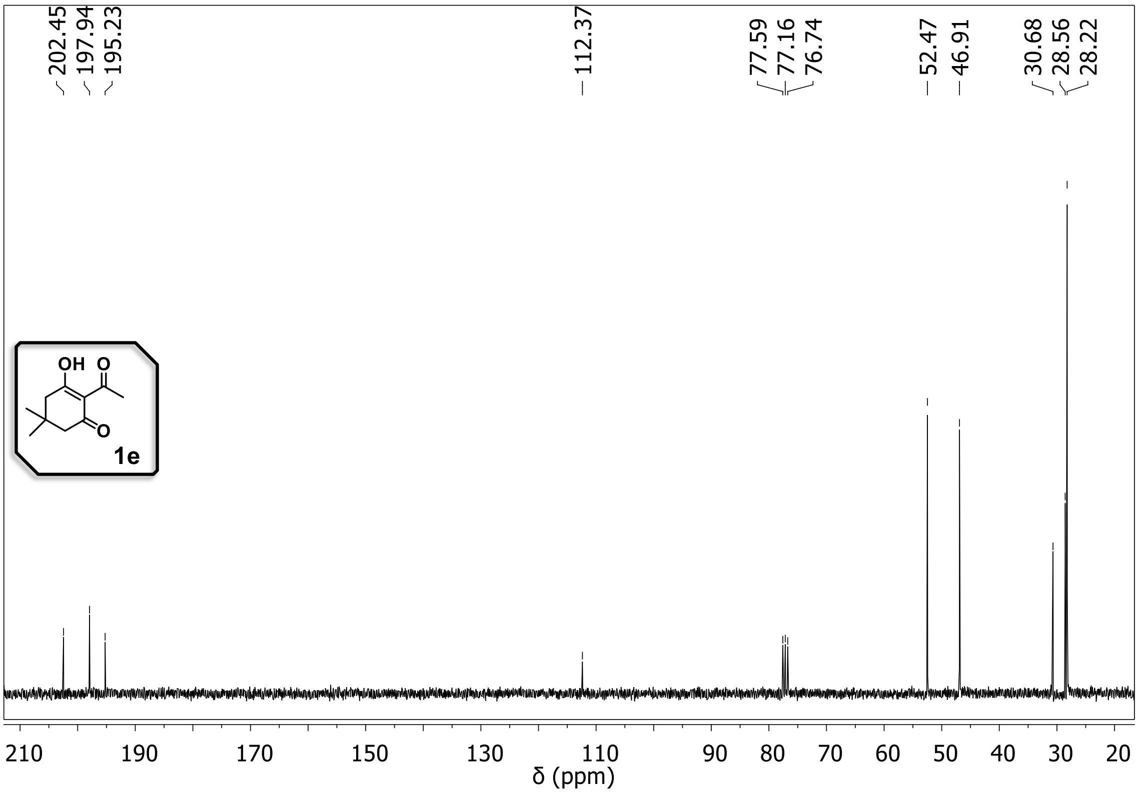

Figure S17. ^{13}C NMR (75 MHZ, $CDCl_3$) spectrum of **1e**.

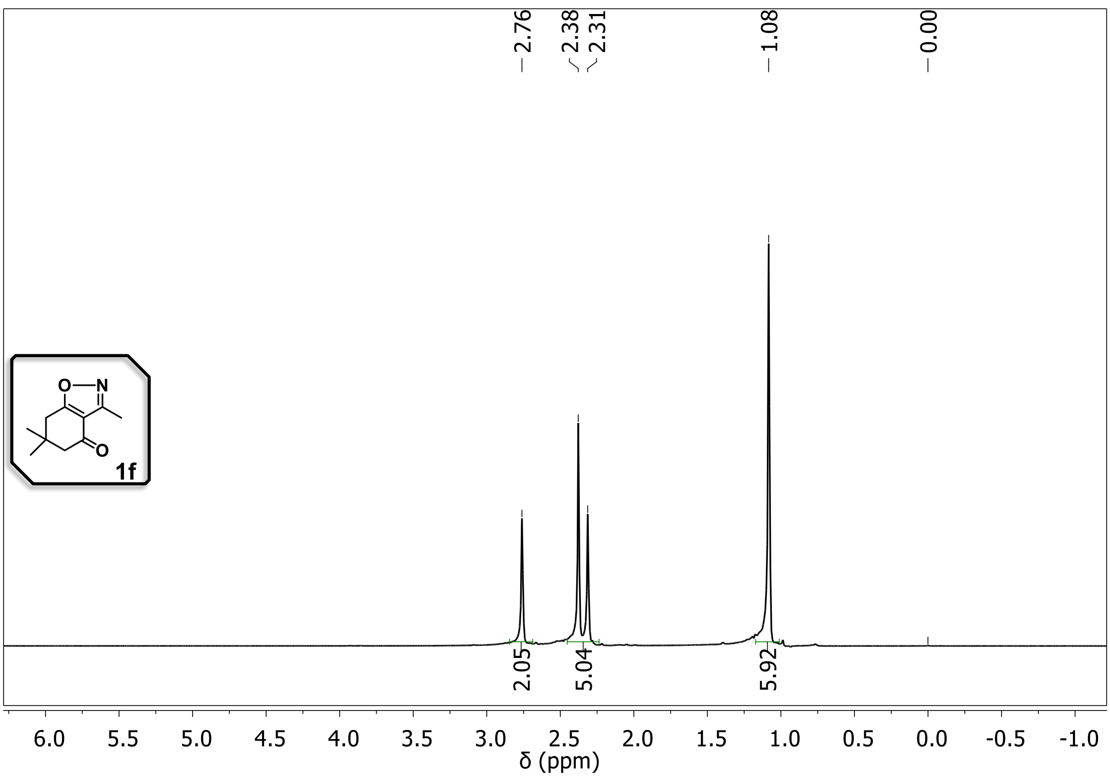

Figure S18. ^{1}H NMR (300 MHz, $CDCl_3$) spectrum of **1f**.

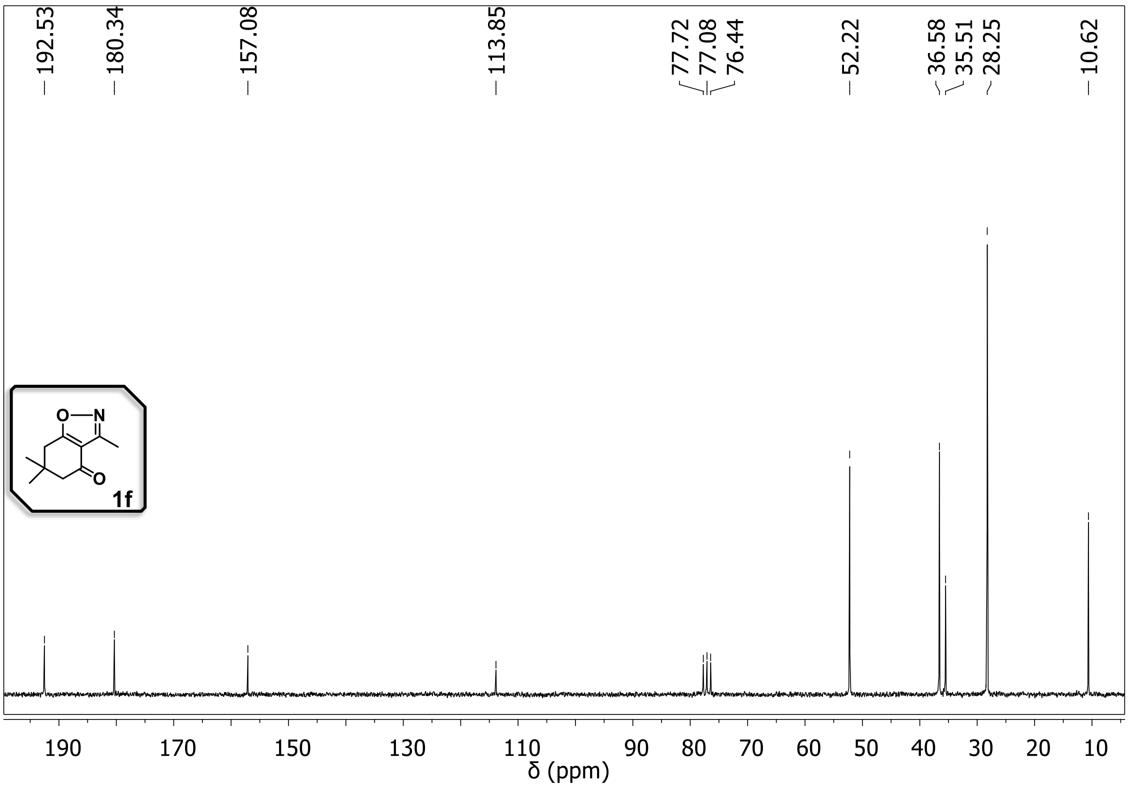

Figure S19. ^{13}C NMR (75 MHZ, $CDCl_3$) spectrum of **1f**.

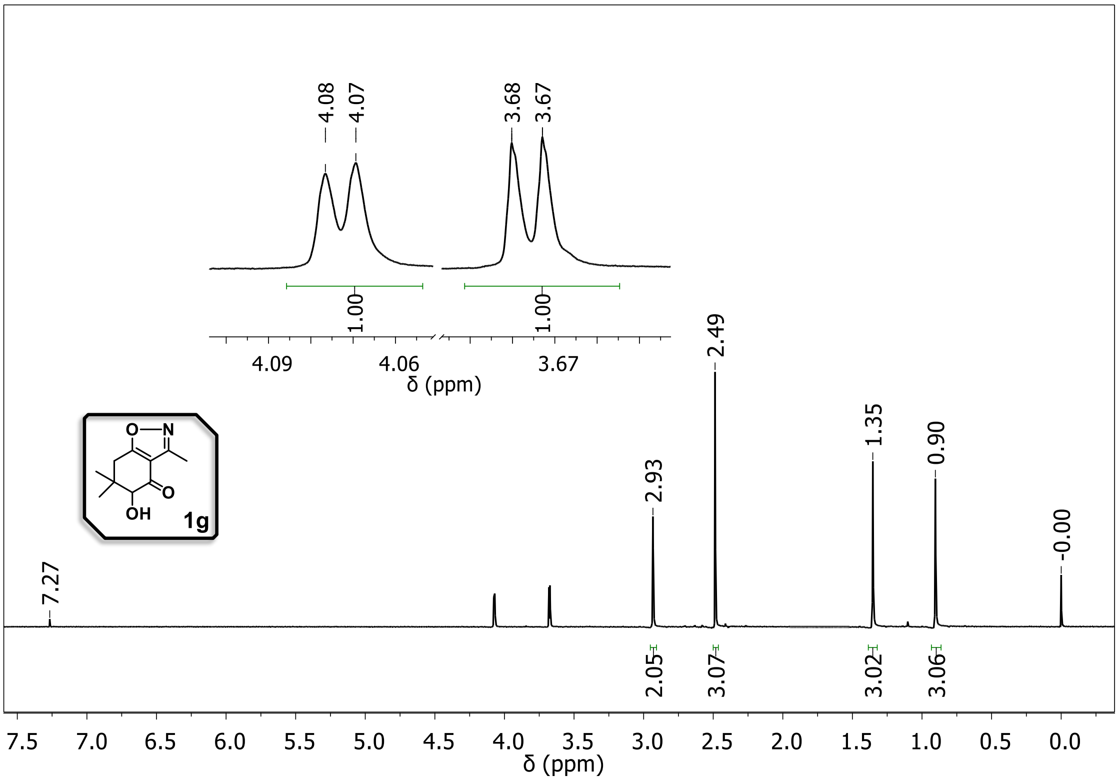

Figure S20. ^{1}H NMR (300 MHz, $CDCl_3$) spectrum of **1g**.

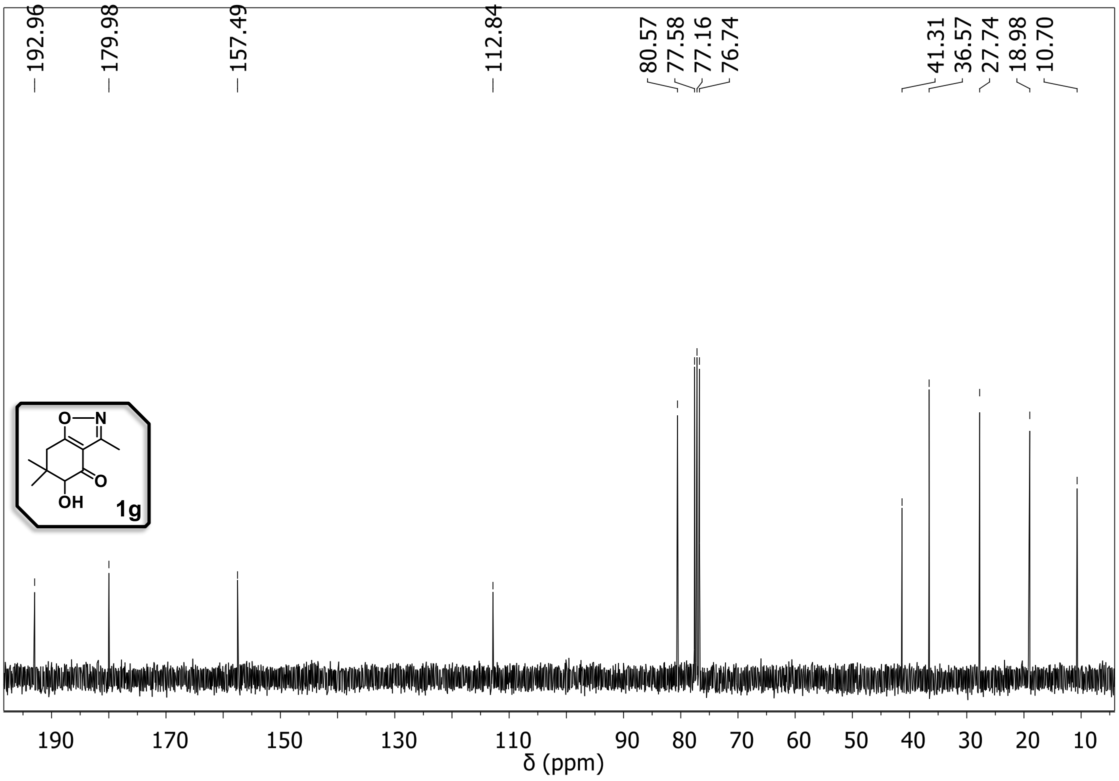

Figure S21. ^{13}C NMR (75 MHZ, $CDCl_3$) spectrum of **1g**.

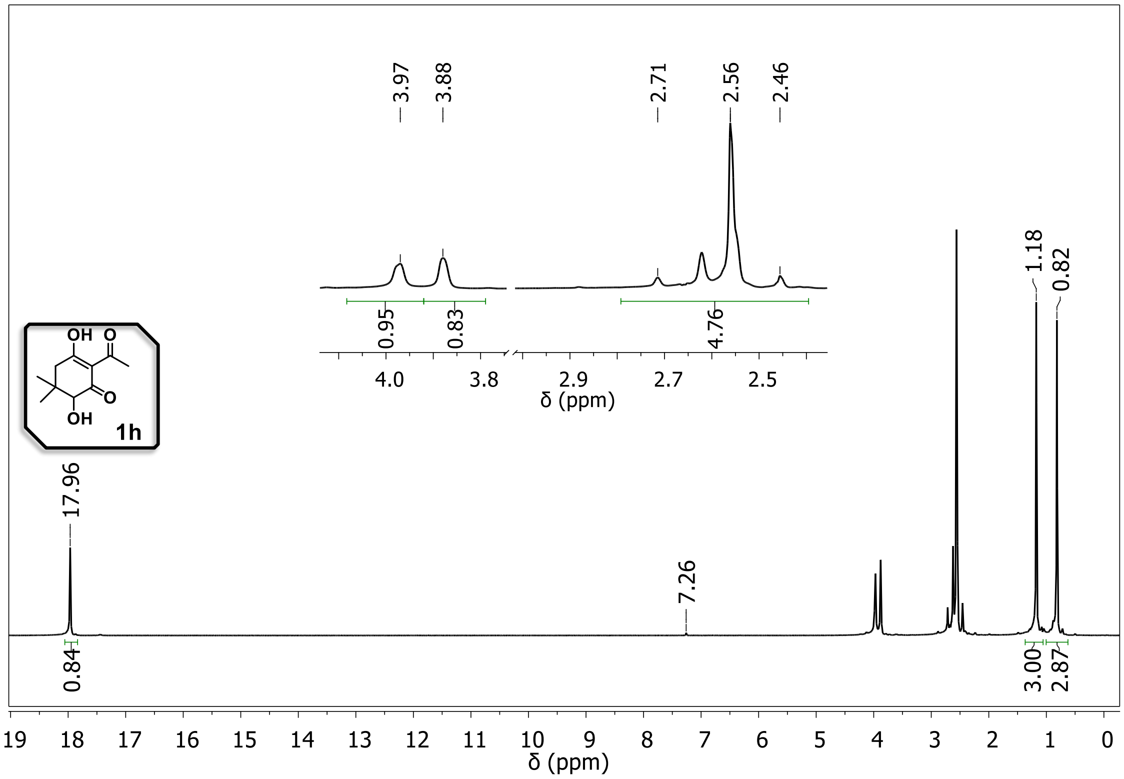

Figure S22. ^{1}H NMR (300 MHz, $CDCl_3$) spectrum of **1h**.

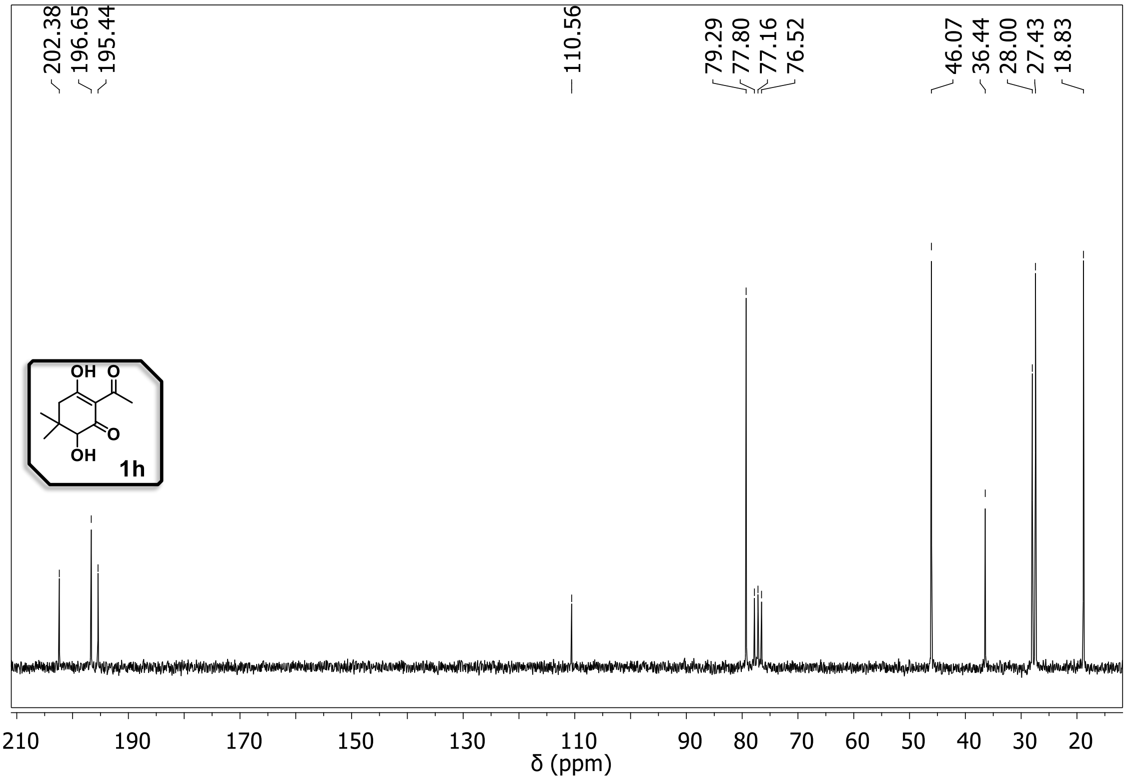

Figure S23. ^{13}C NMR (75 MHZ, $CDCl_3$) spectrum of **1h**.

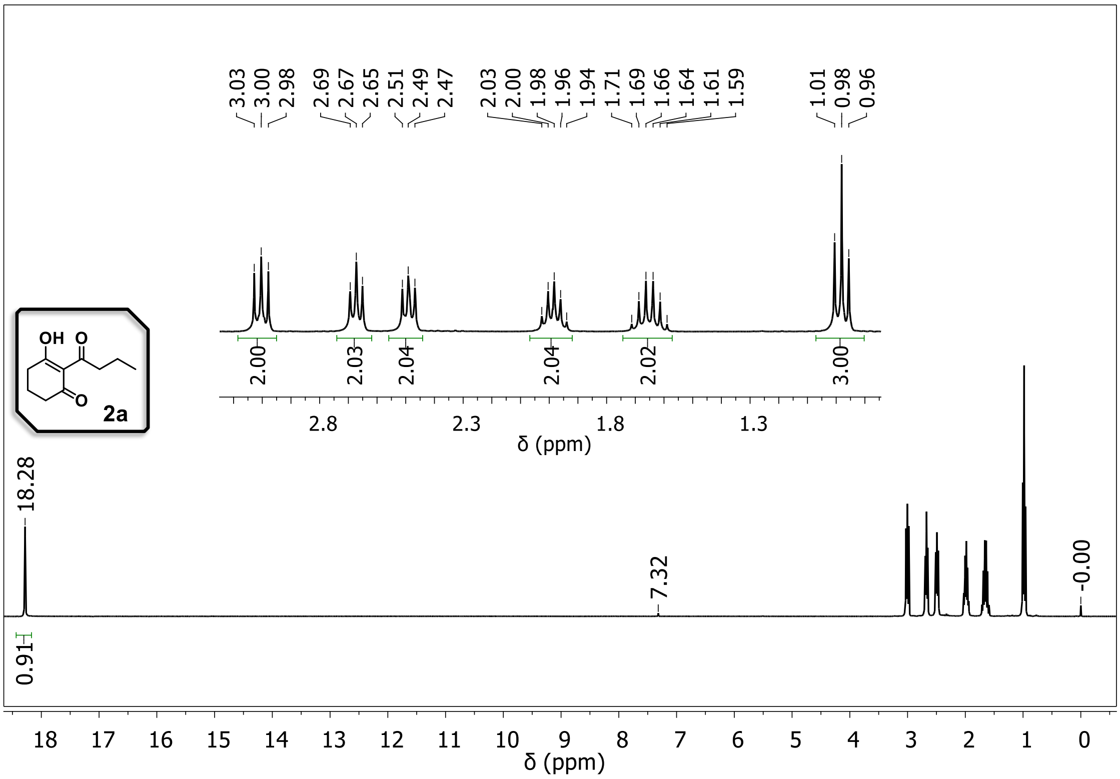

Figure S24. ^{1}H NMR (300 MHz, $CDCl_3$) spectrum of **2a**.

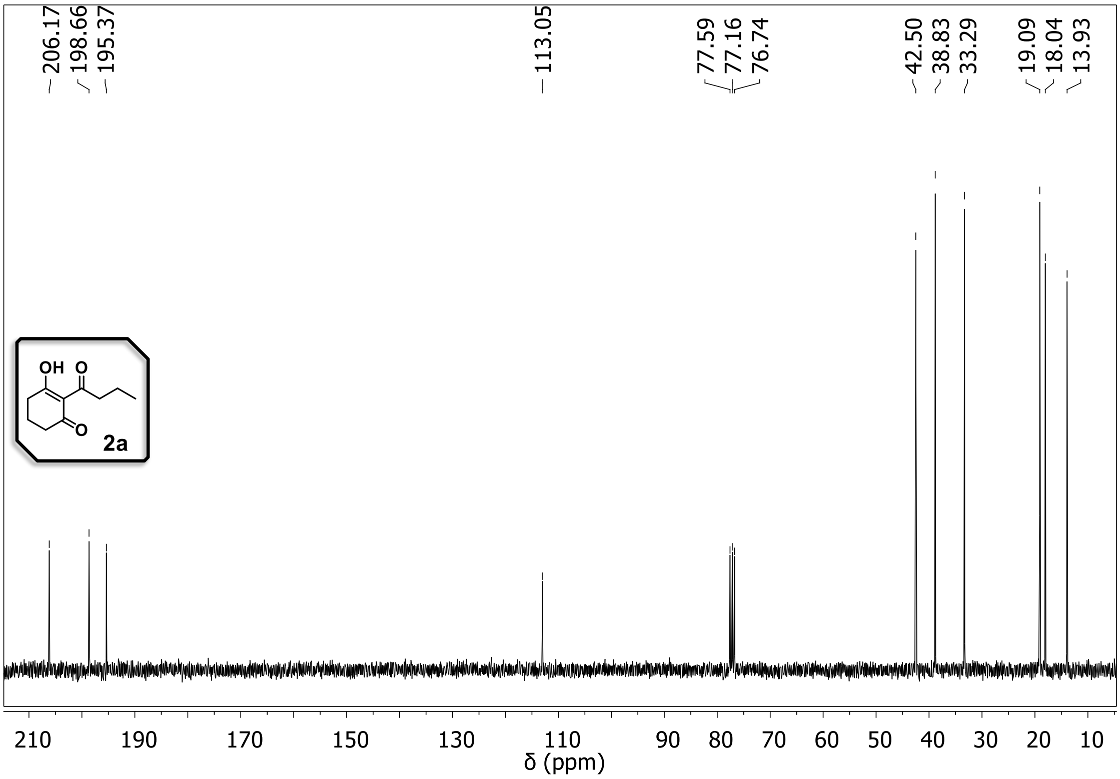

Figure S25. ^{13}C NMR (75 MHZ, $CDCl_3$) spectrum of **2a**.

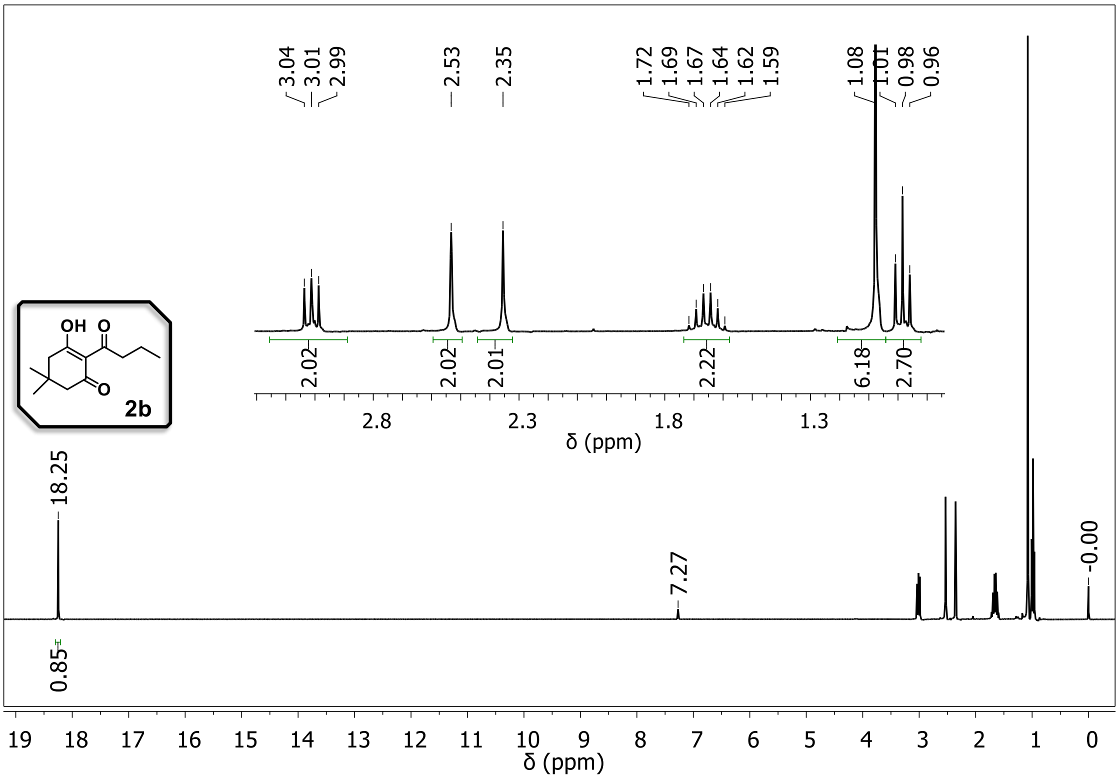

Figure S26. ^{1}H NMR (300 MHz, $CDCl_3$) spectrum of **2b**.

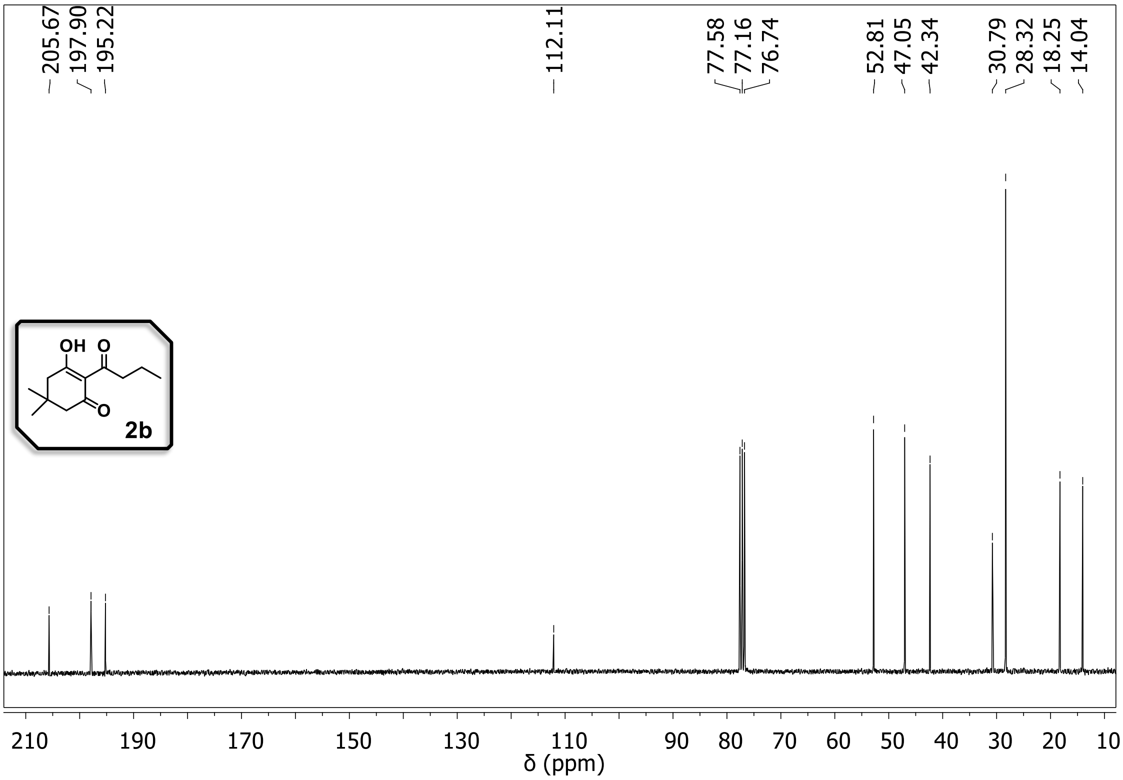

Figure S27. ^{13}C NMR (75 MHZ, $CDCl_3$) spectrum of **2b**.

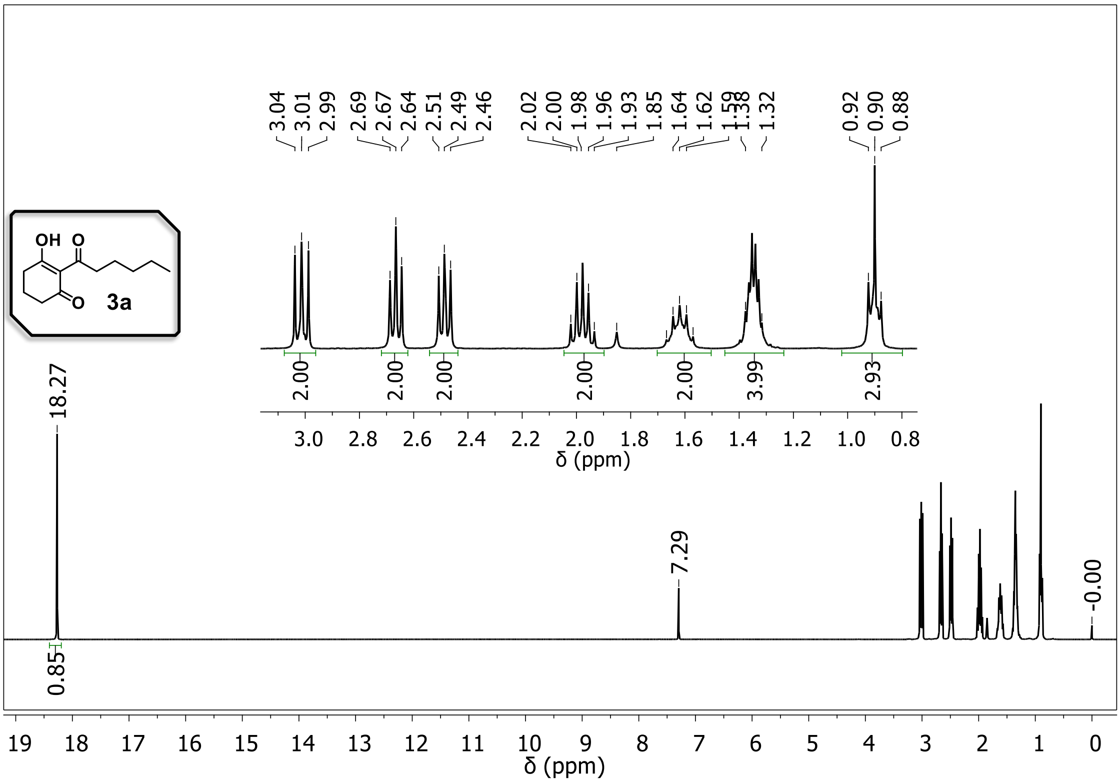

Figure S28. ^{1}H NMR (300 MHz, $CDCl_3$) spectrum of **3a**.

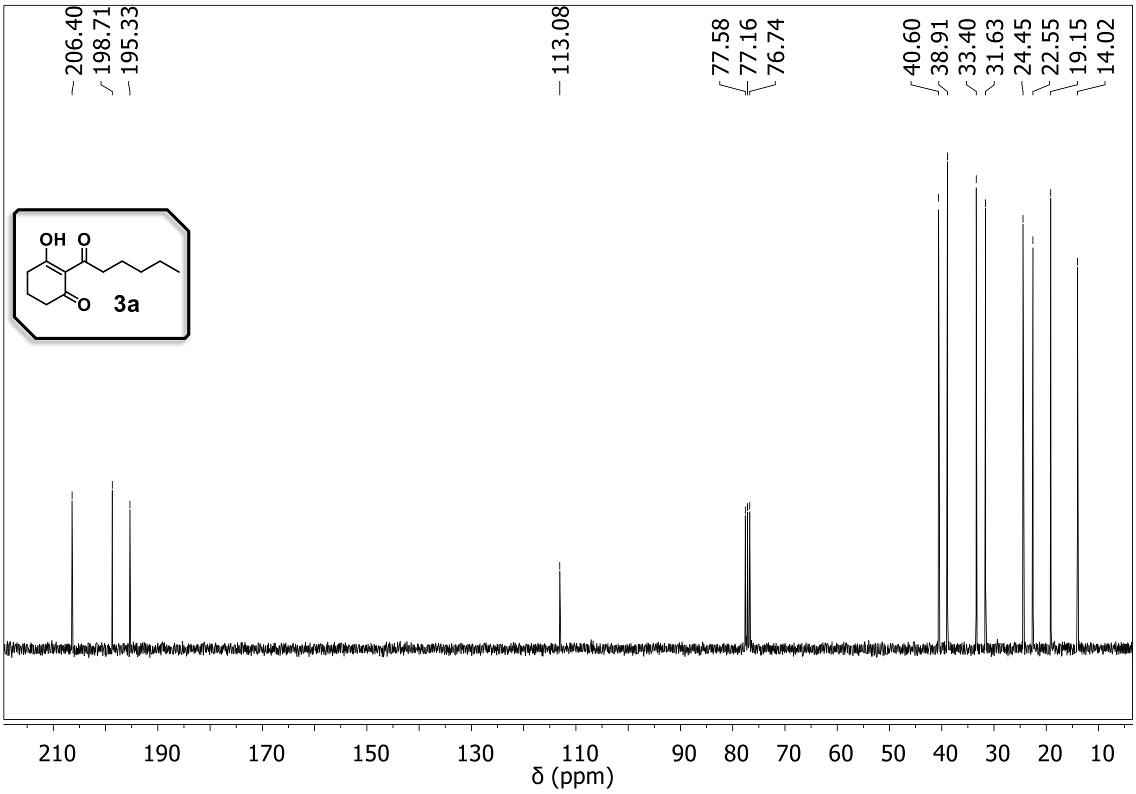

Figure S29. ^{13}C NMR (75 MHZ, $CDCl_3$) spectrum of **3a**.

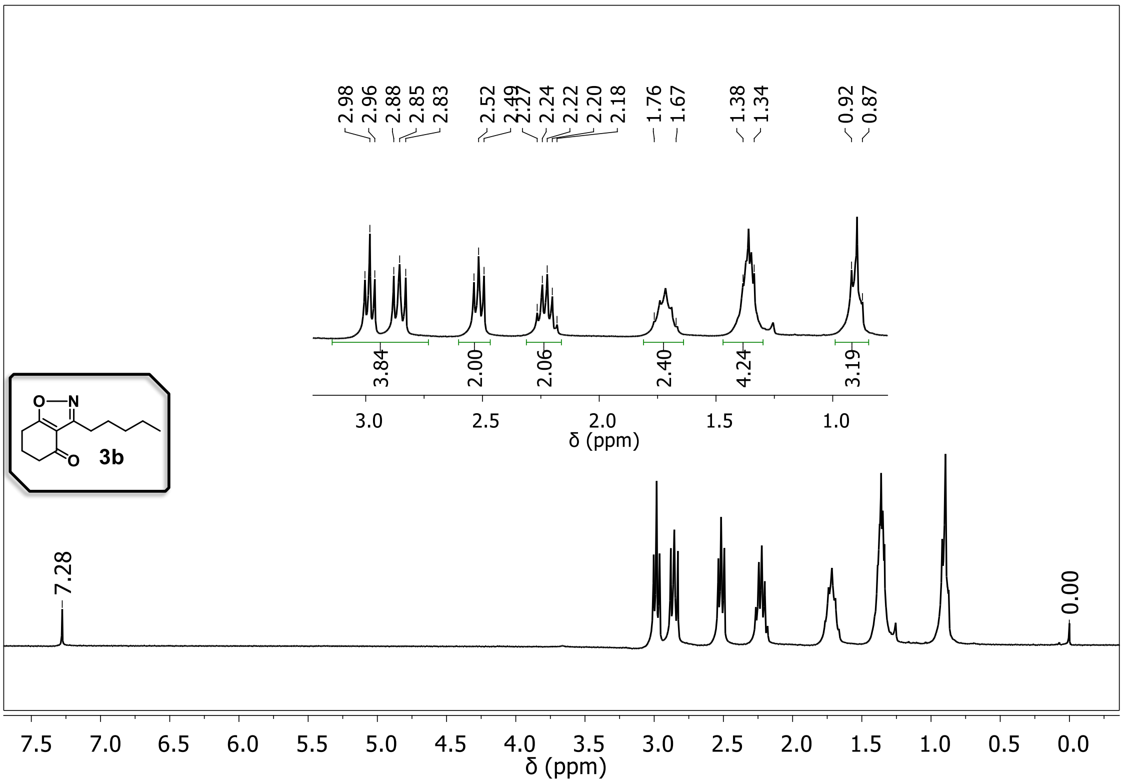

Figure S30. ^{1}H NMR (300 MHz, $CDCl_3$) spectrum of **3b**.

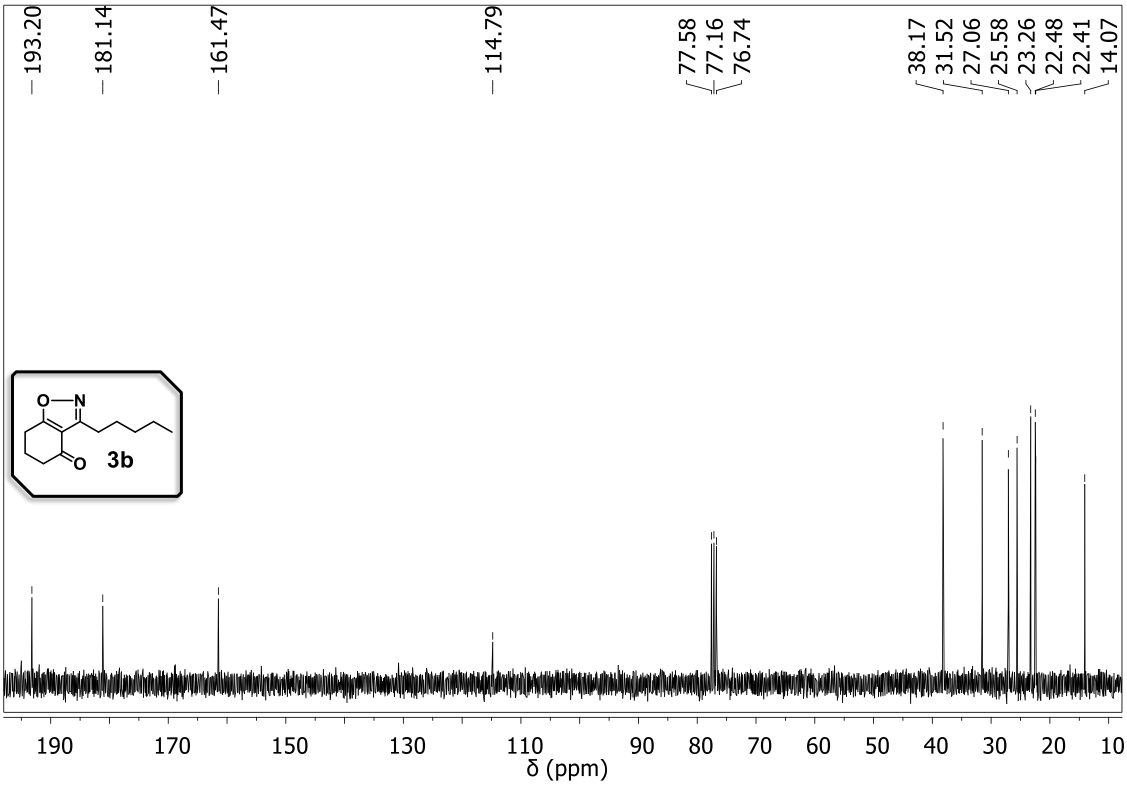

Figure S31. ^{13}C NMR (75 MHZ, $CDCl_3$) spectrum of **3b**.

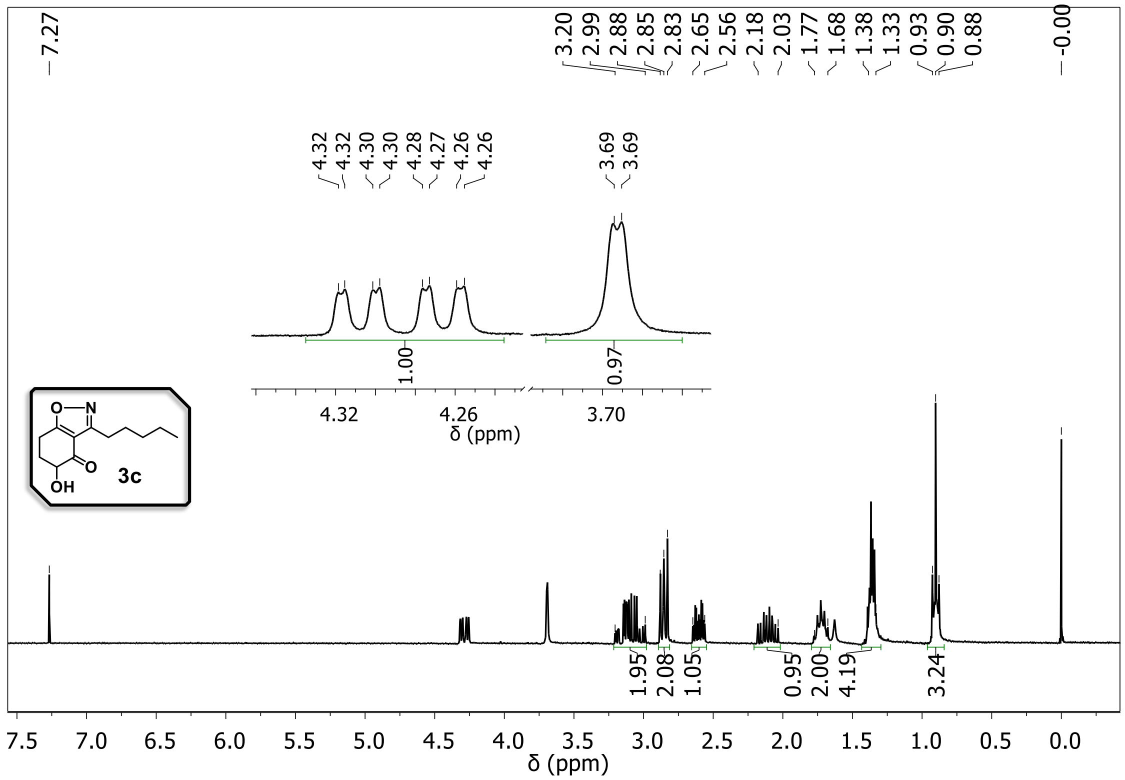

Figure S32. ^{1}H NMR (300 MHz, $CDCl_3$) spectrum of **3c**.

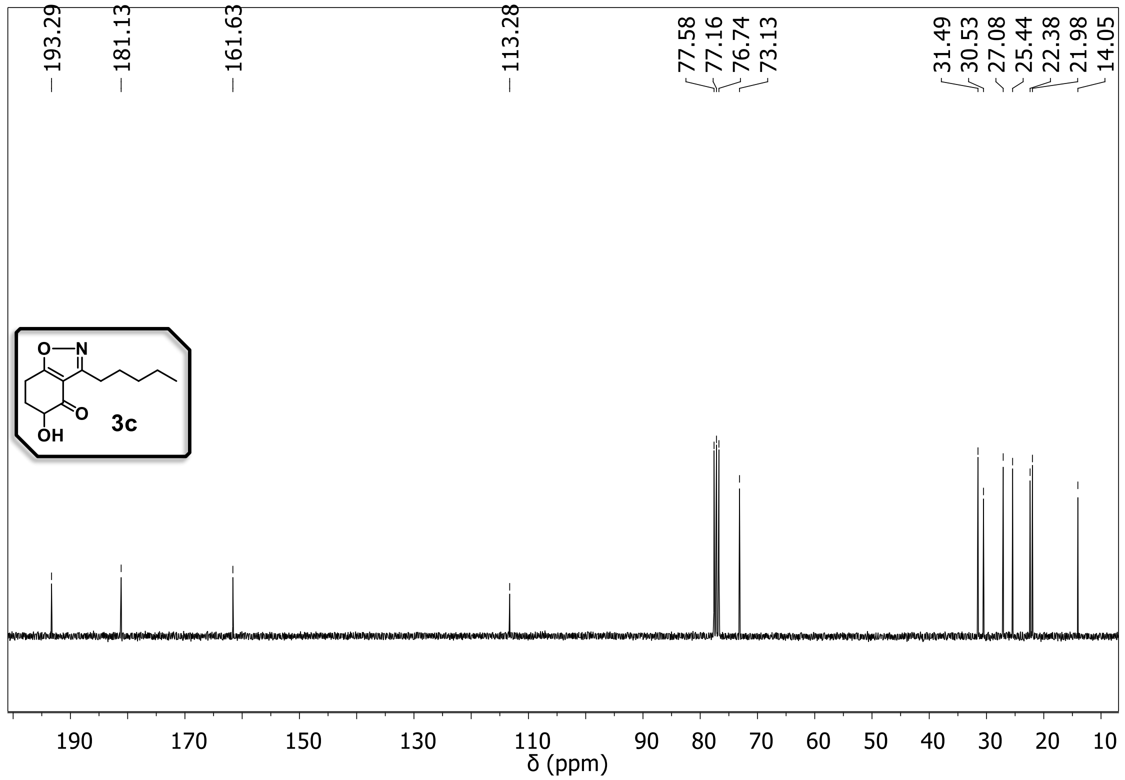

Figure S33. ^{13}C NMR (75 MHZ, $CDCl_3$) spectrum of **3c**.

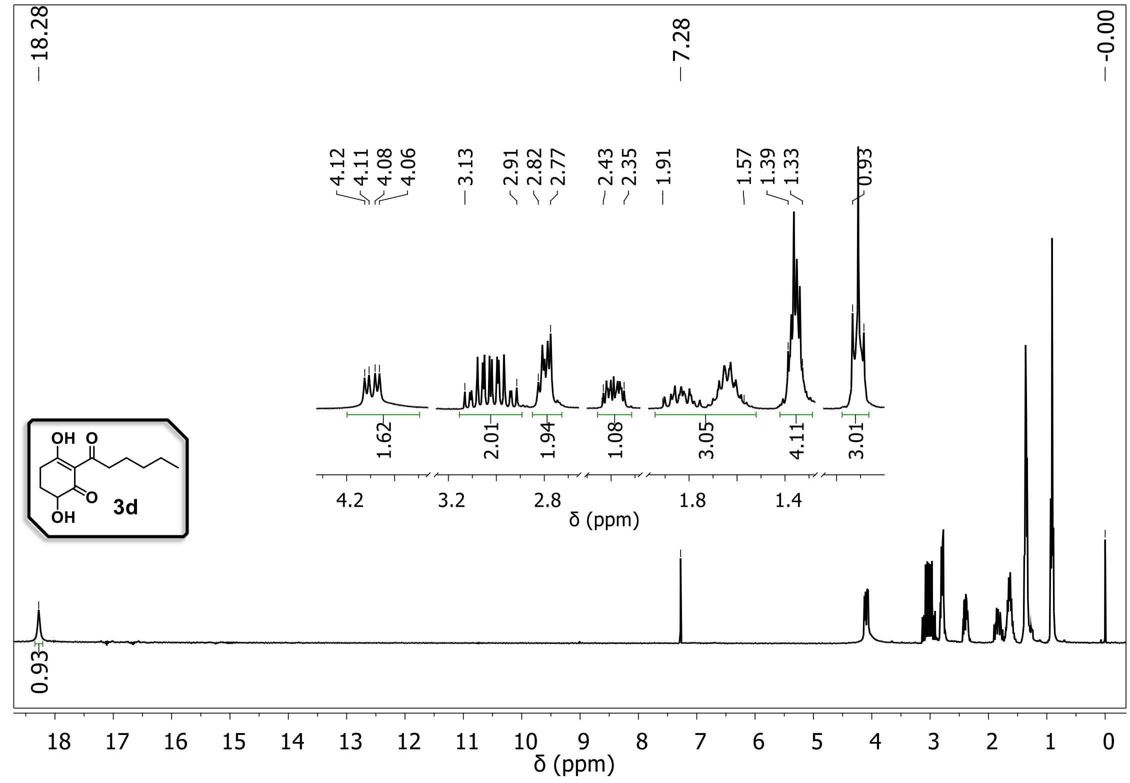

Figure S34. ^{1}H NMR (300 MHz, CDCl$_3$) spectrum of **3d**.

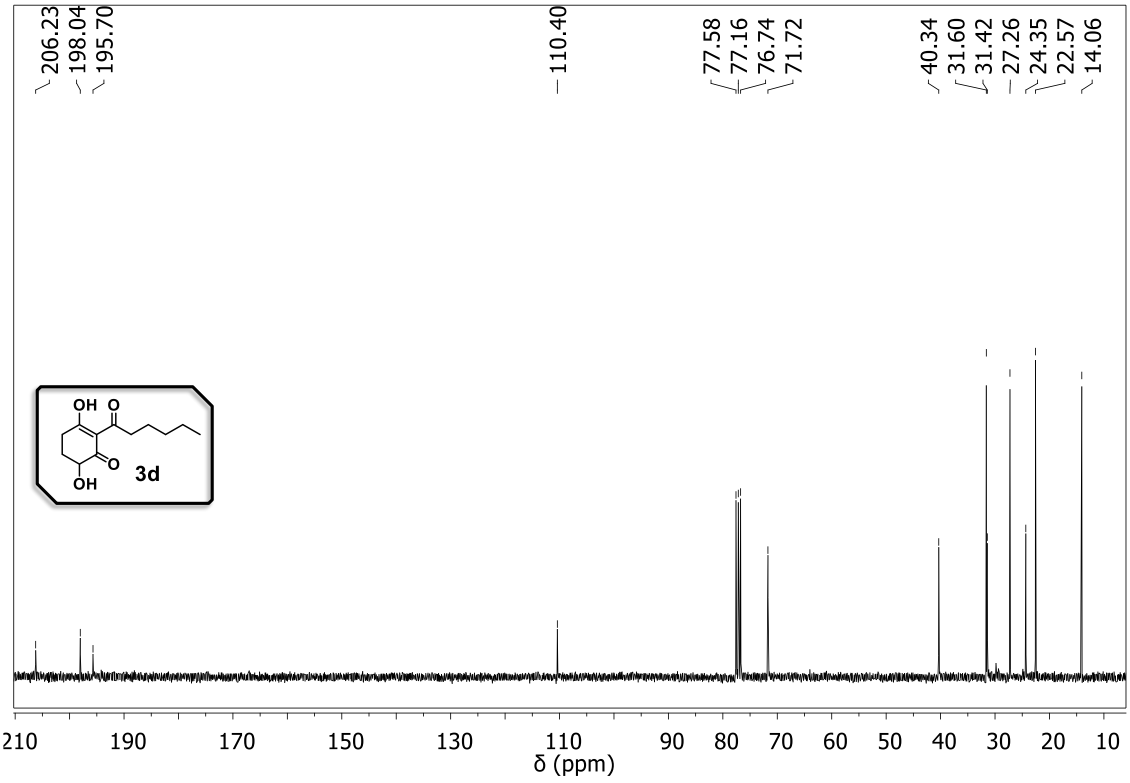

Figure S35. ^{13}C NMR (75 MHZ, CDCl$_3$) spectrum of **3d**.

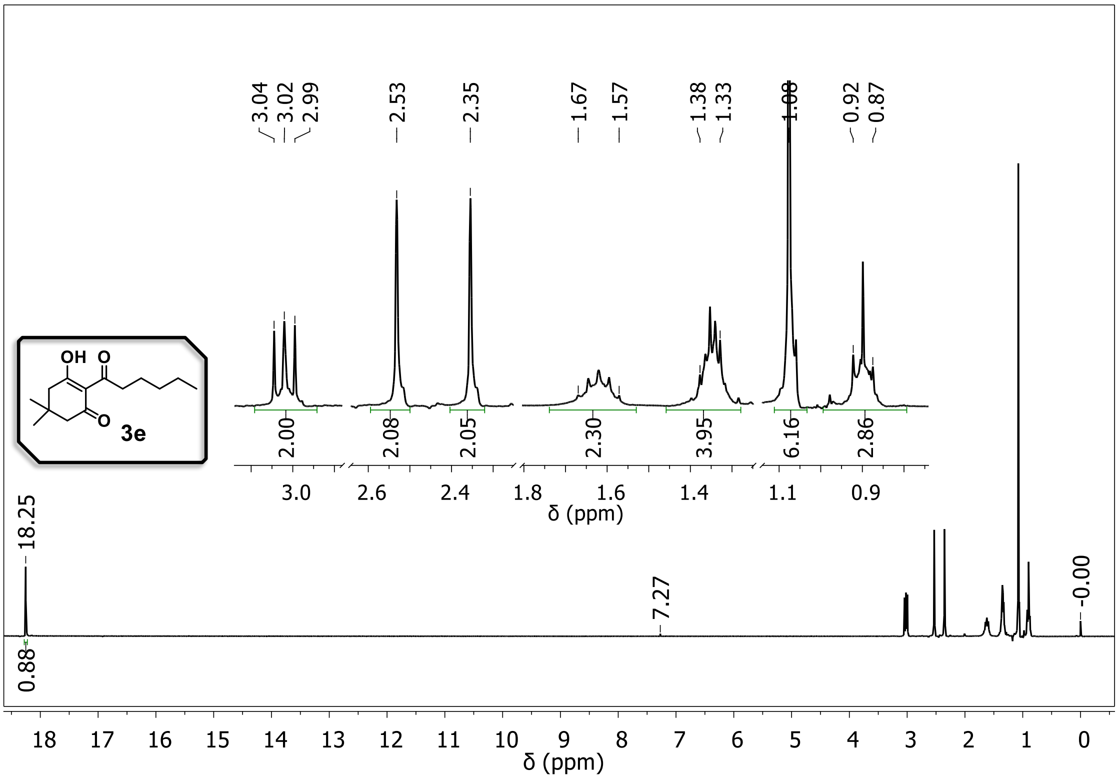

Figure S36. ^{1}H NMR (300 MHz, $CDCl_3$) spectrum of **3e**.

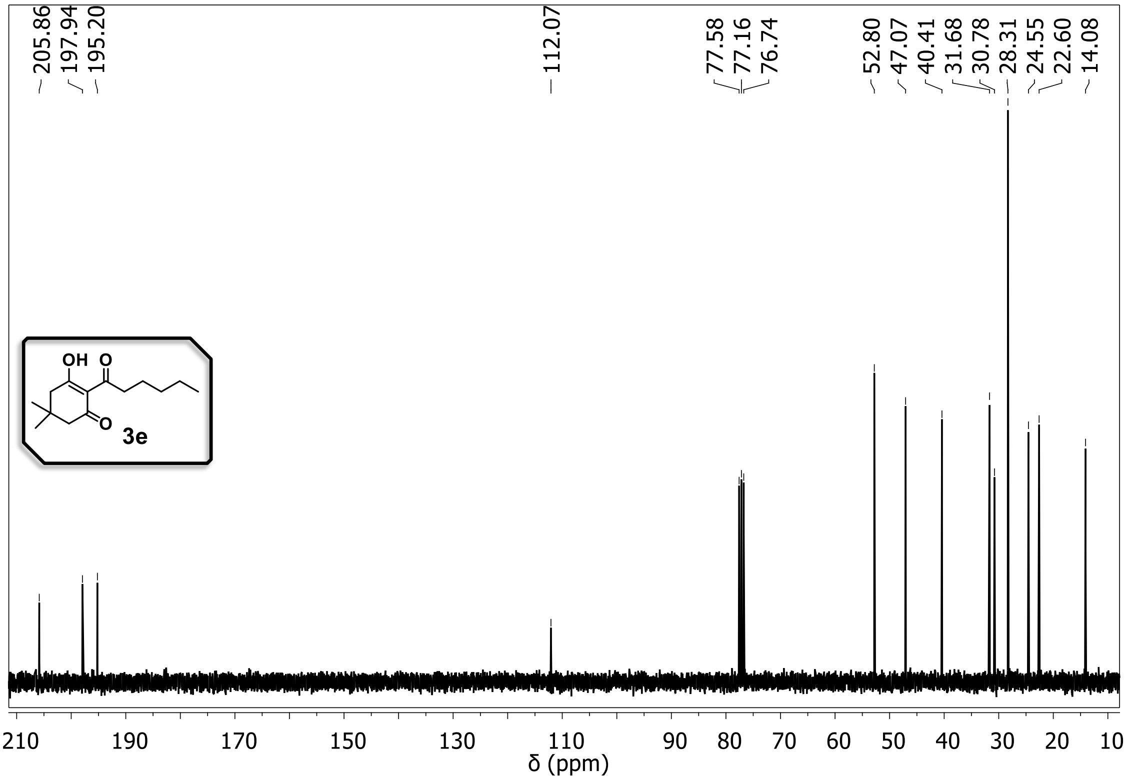

Figure S37. ^{13}C NMR (75 MHZ, $CDCl_3$) spectrum of **3e**.

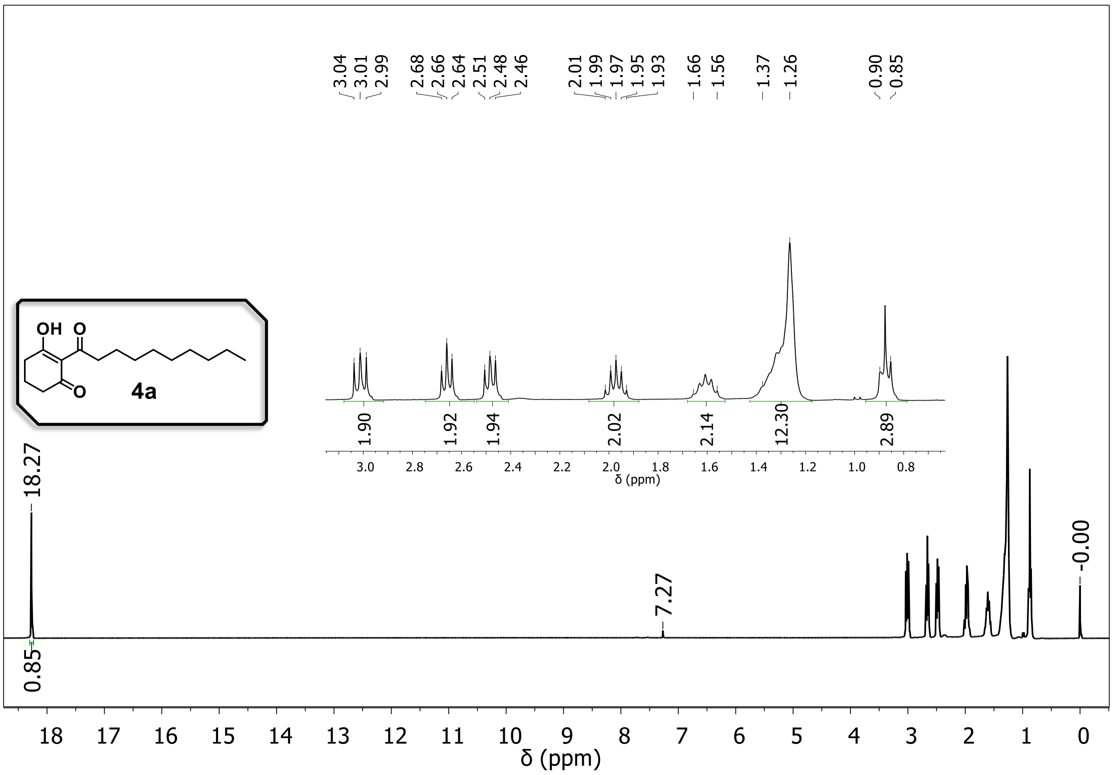

Figure S38. ^{1}H NMR (300 MHz, $CDCl_3$) spectrum of **4a**.

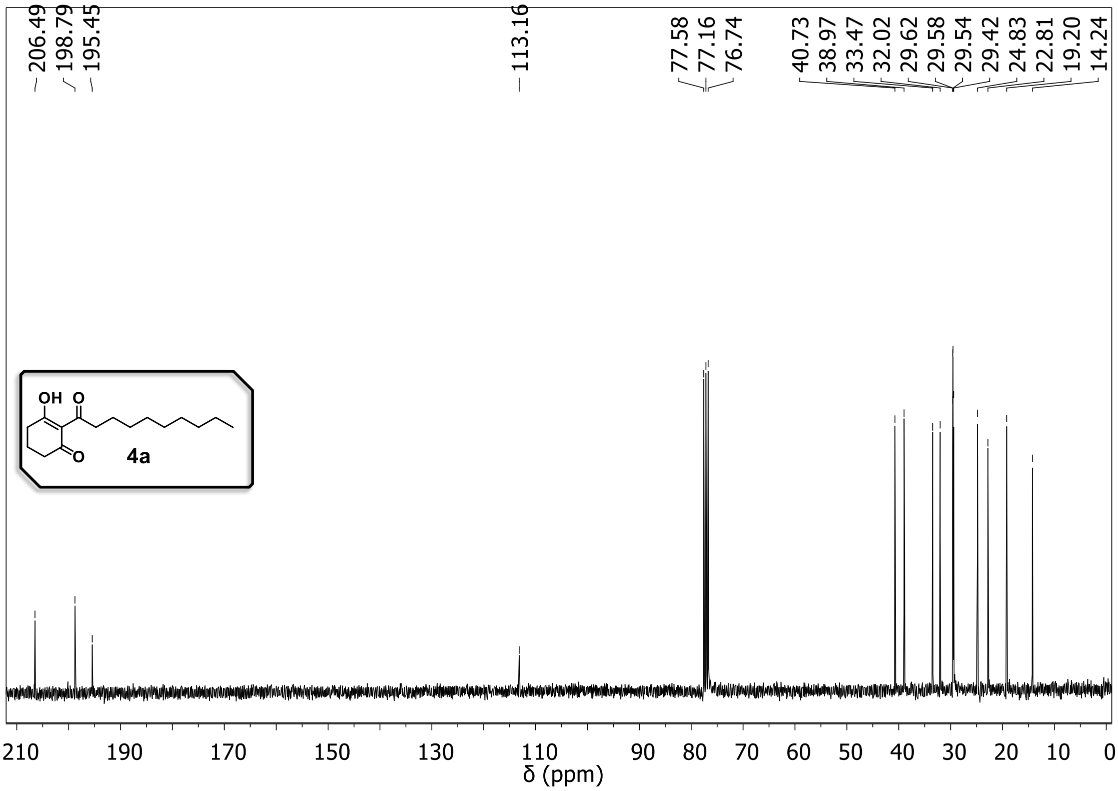

Figure S39. ^{13}C NMR (75 MHZ, $CDCl_3$) spectrum of **4a**.

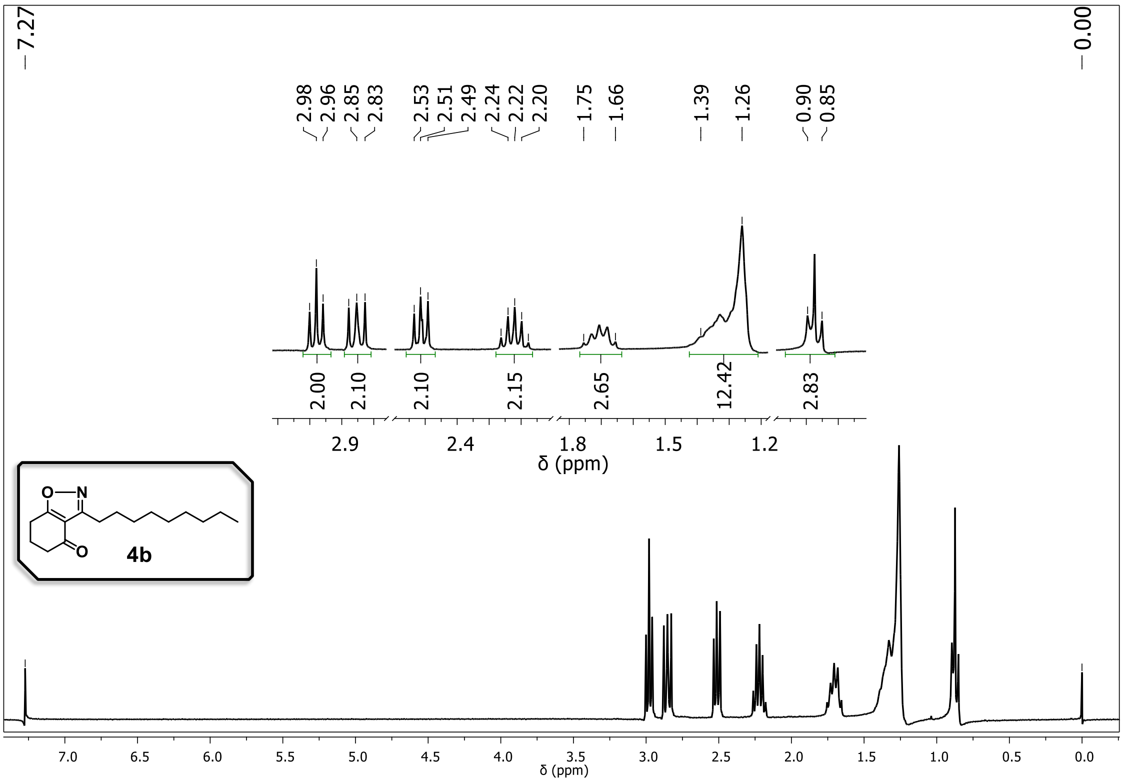

Figure S40. ^{1}H NMR (300 MHz, $CDCl_3$) spectrum of **4b**.

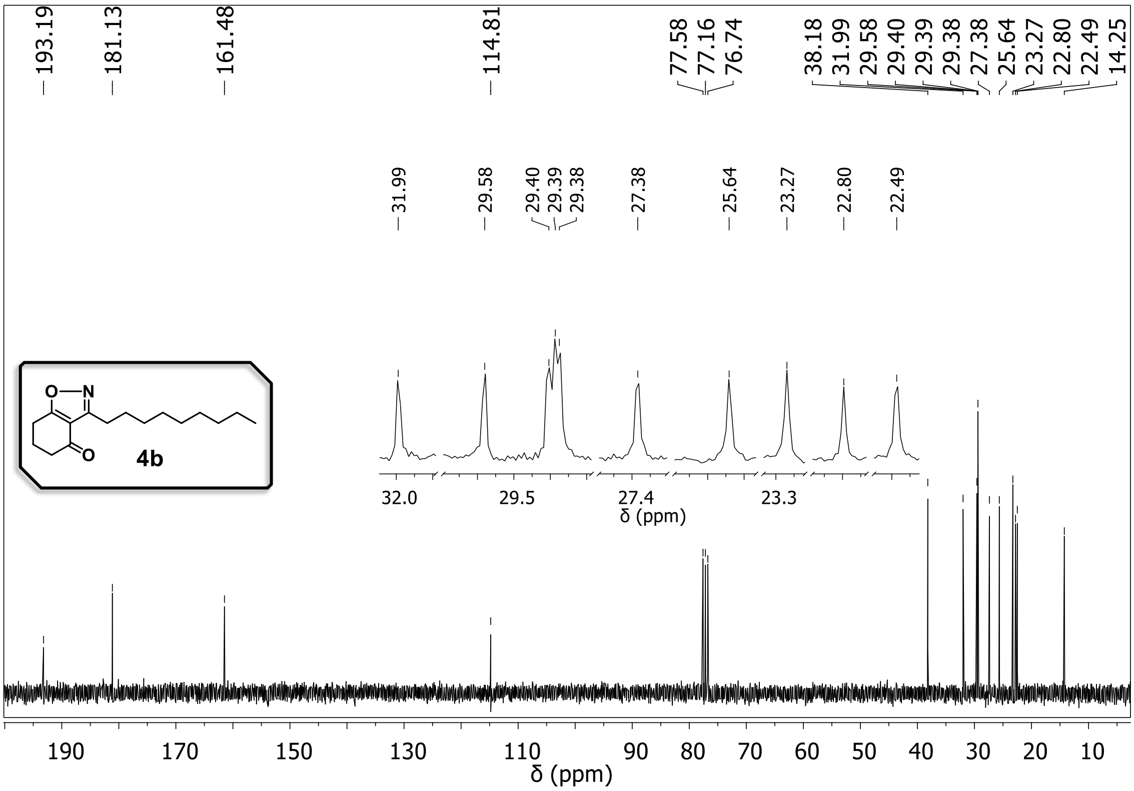

Figure S41. ^{13}C NMR (75 MHZ, $CDCl_3$) spectrum of **4b**.

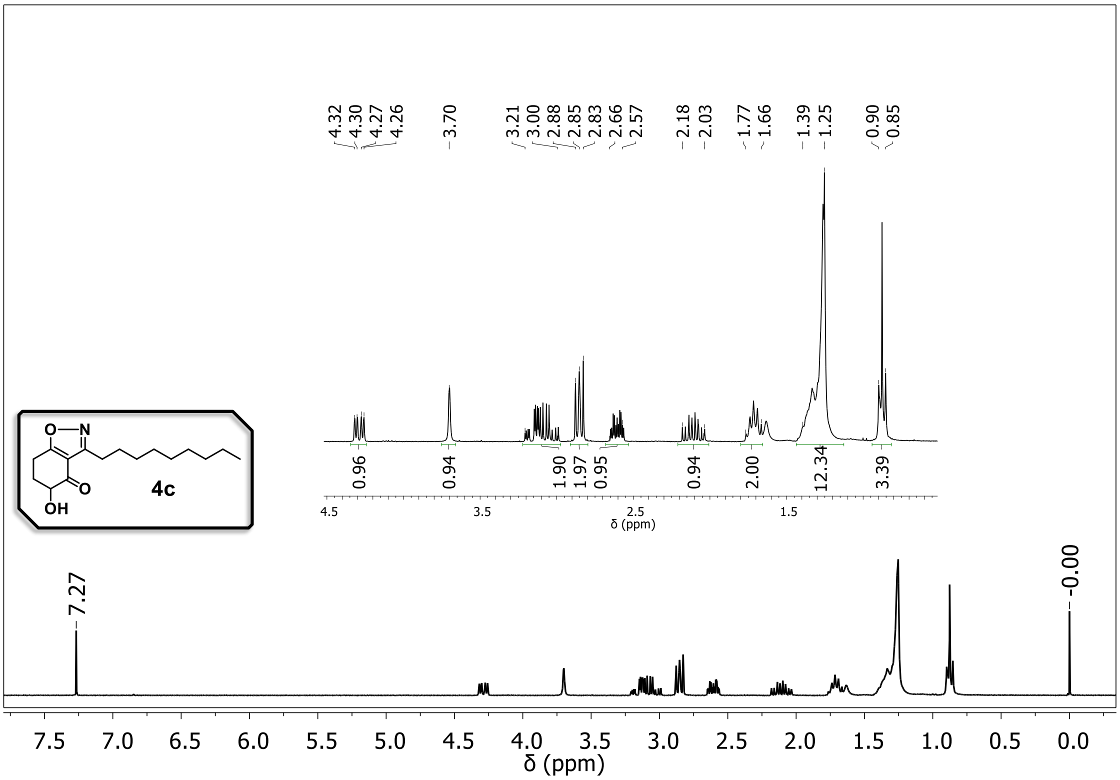

Figure S42. ^{1}H NMR (300 MHz, $CDCl_3$) spectrum of **4c**.

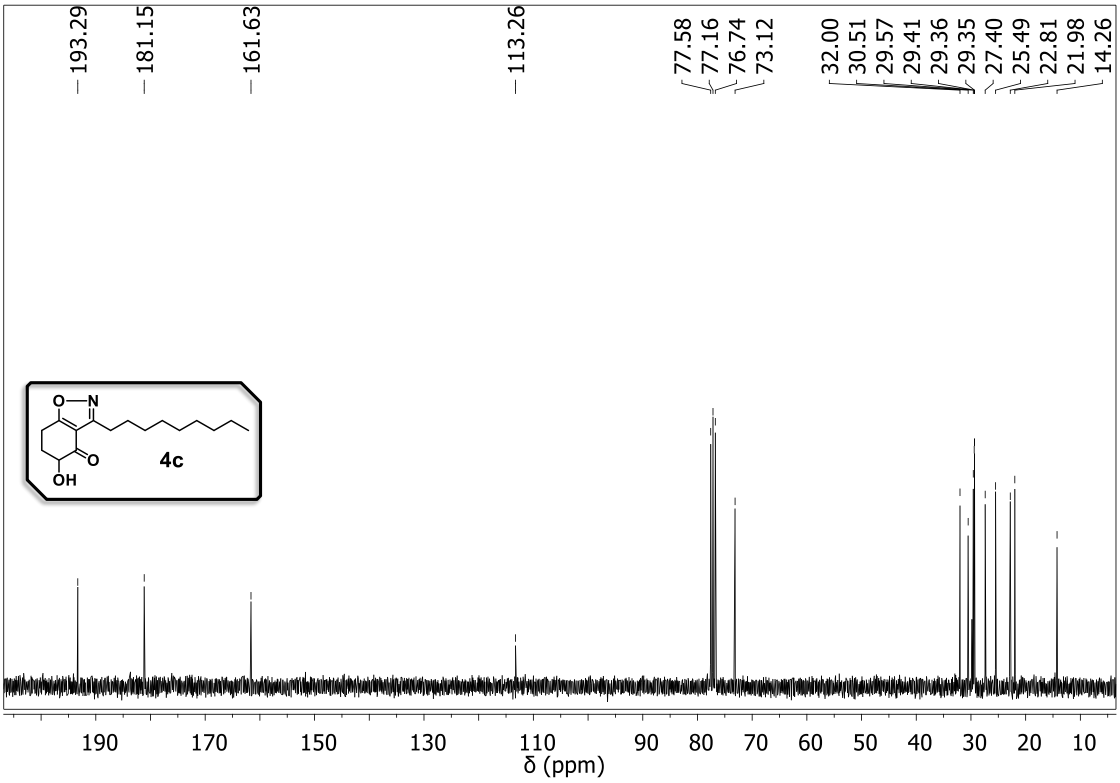

Figure S43. ^{13}C NMR (75 MHZ, $CDCl_3$) (300 MHz, $CDCl_3$) spectrum of **4c**.

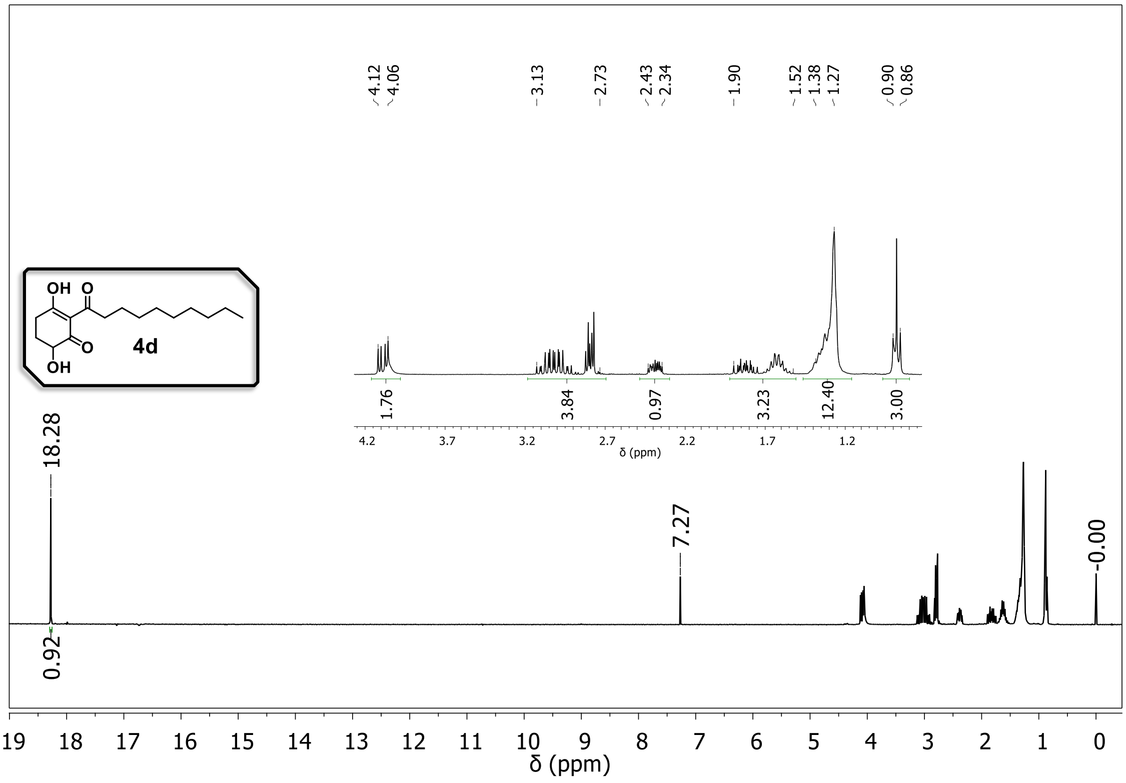

Figure S44. ^{1}H NMR (300 MHz, $CDCl_3$) spectrum of **4d**.

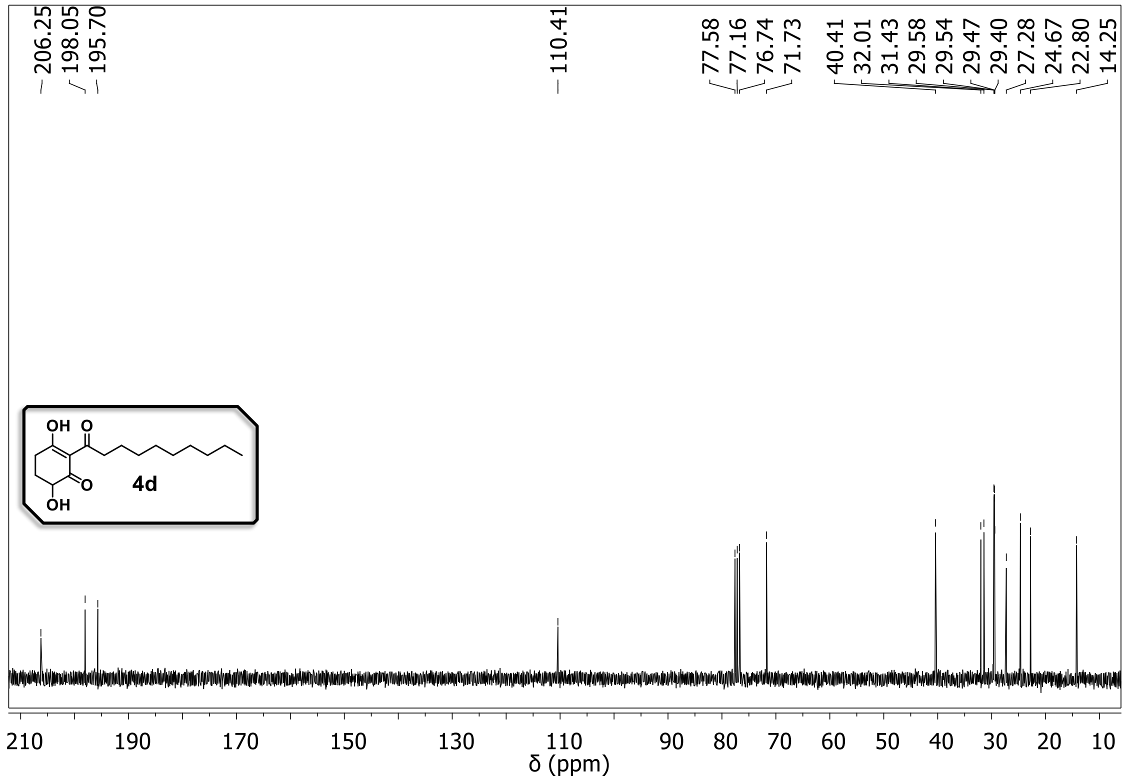

Figure S45. ^{13}C NMR (75 MHZ, $CDCl_3$) spectrum of **4d**.

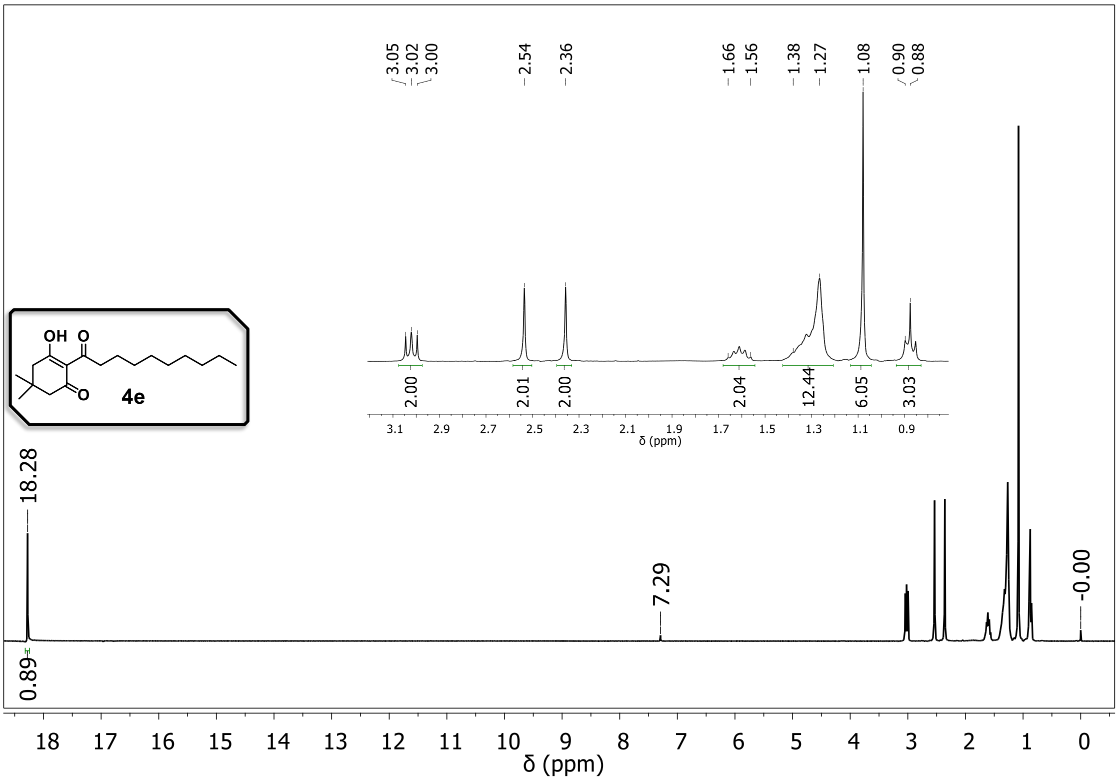

Figure S46. ^{1}H NMR (300 MHz, $CDCl_3$) spectrum of **4e**.

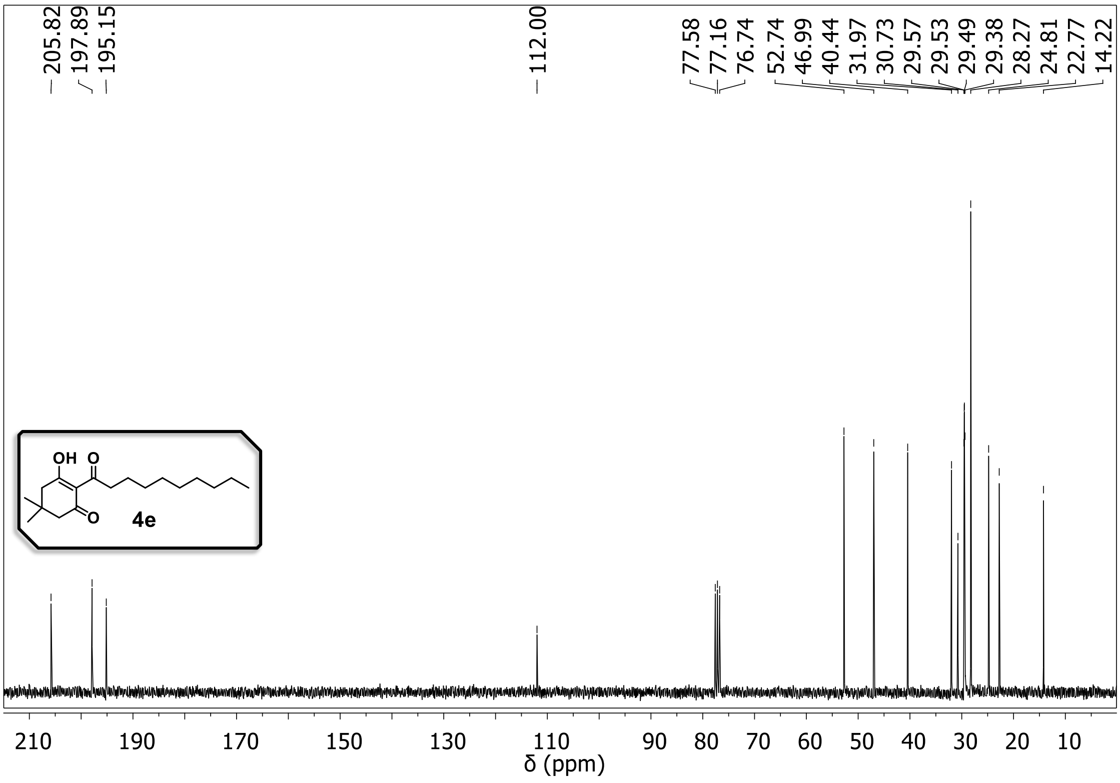

Figure S47. ^{13}C NMR (75 MHZ, $CDCl_3$) spectrum of **4e**.

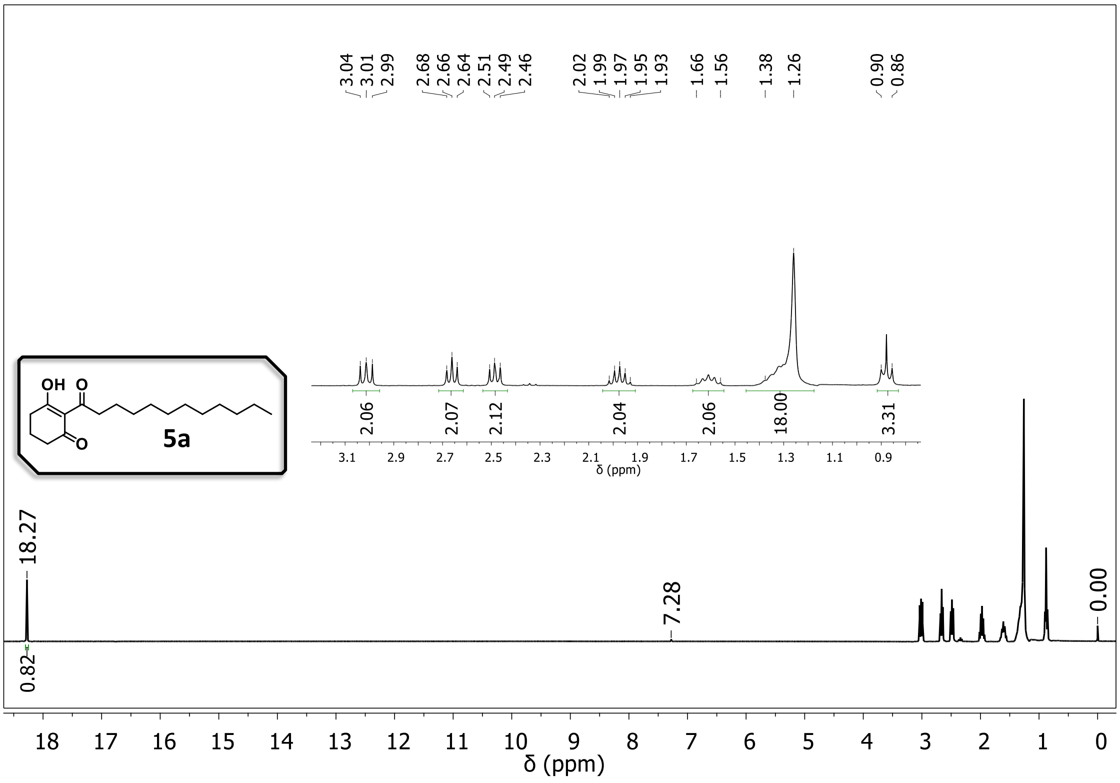

Figure S48. ^{1}H NMR (300 MHz, $CDCl_3$) spectrum of **5a**.

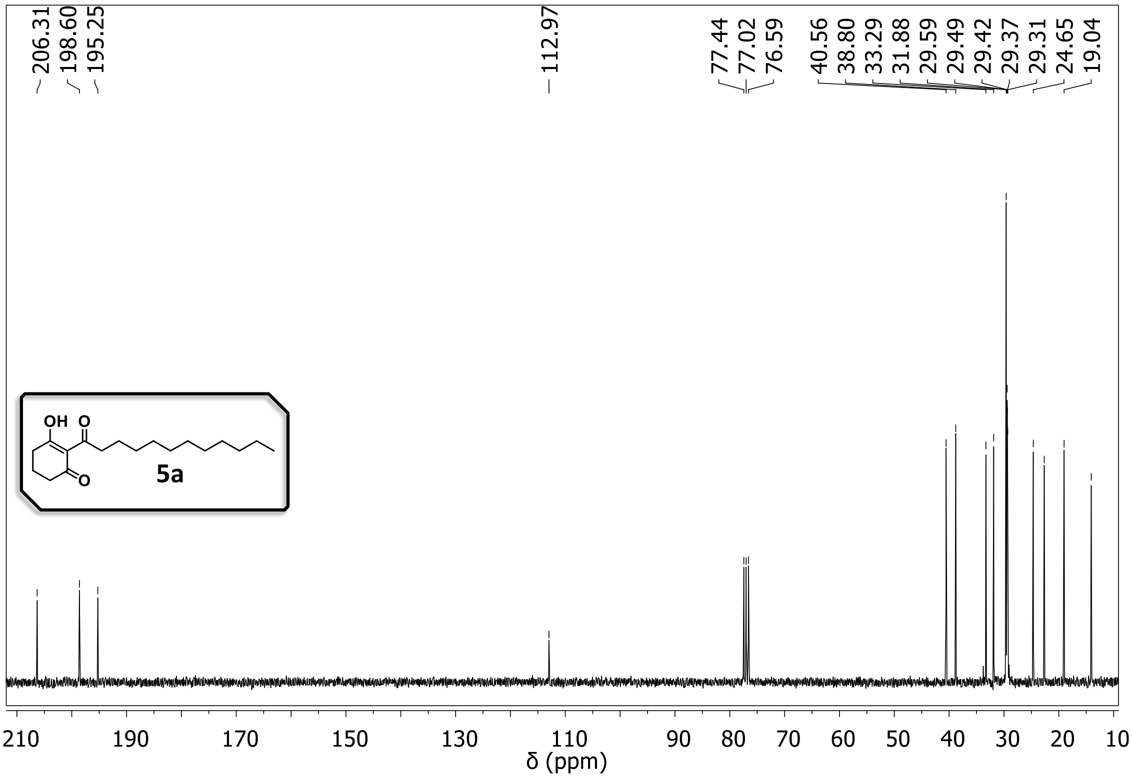

Figure S49. ^{13}C NMR (75 MHZ, $CDCl_3$) spectrum of **5a**.

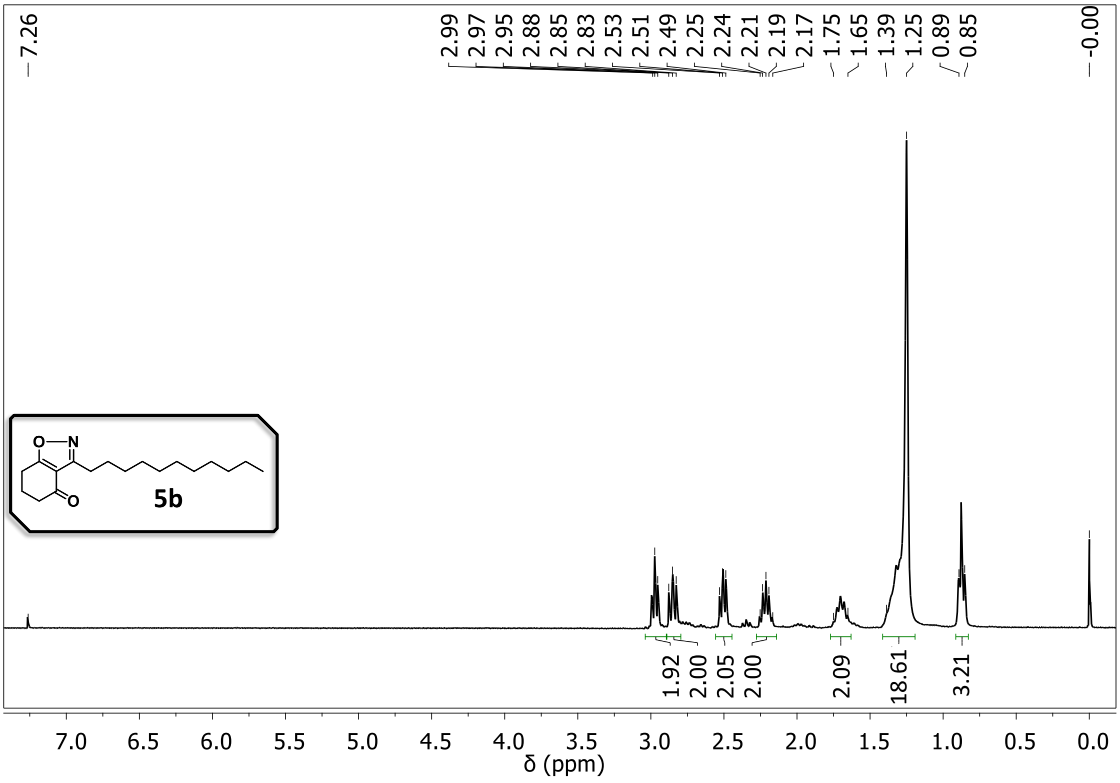

Figure S50. ^{1}H NMR (300 MHz, $CDCl_3$) spectrum of **5b**.

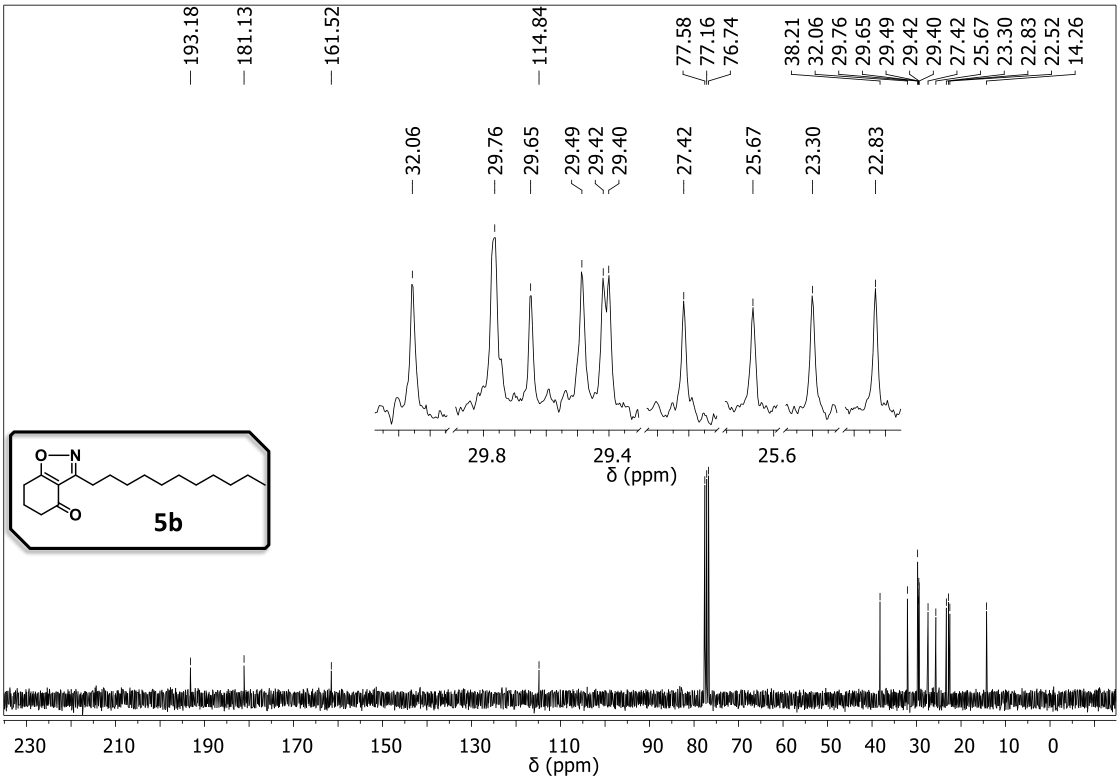

Figure S51. ^{13}C NMR (75 MHZ, $CDCl_3$) spectrum of **5b**.

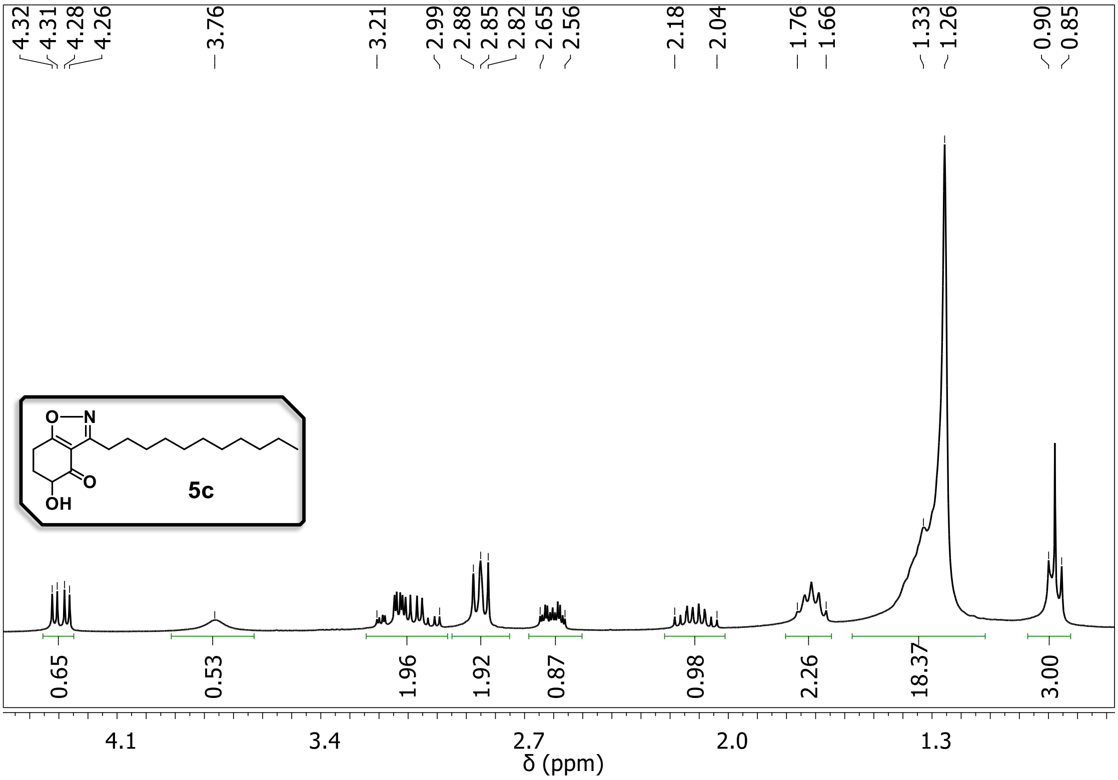

Figure S52. ^{1}H NMR (300 MHz, $CDCl_3$) spectrum of **5c**.

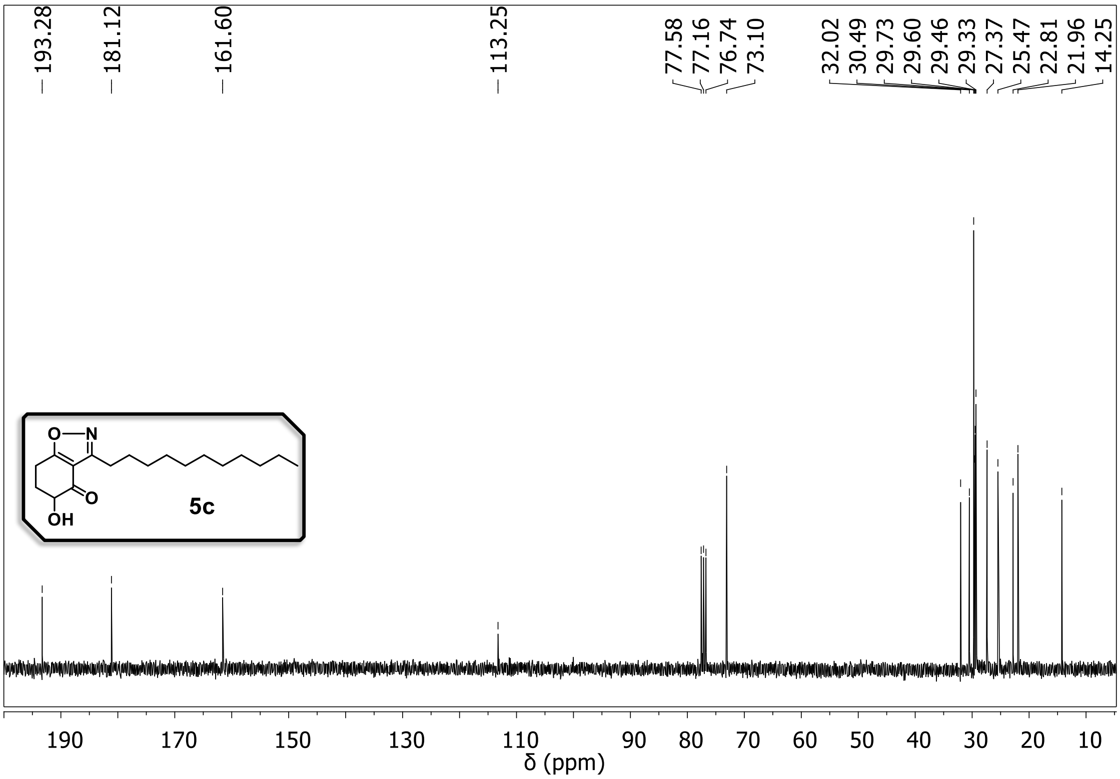

Figure S53. ^{13}C NMR (75 MHZ, $CDCl_3$) spectrum of **5c**.

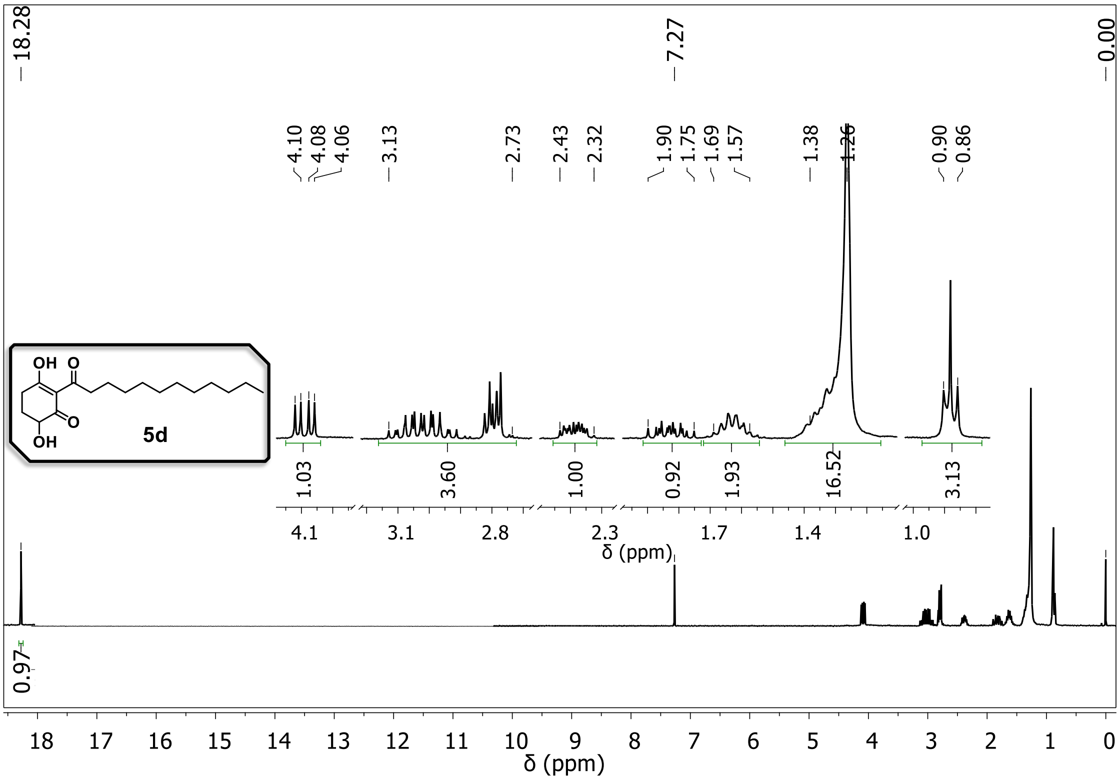

Figure S54. ^{1}H NMR (300 MHz, $CDCl_3$) spectrum of **5d**.

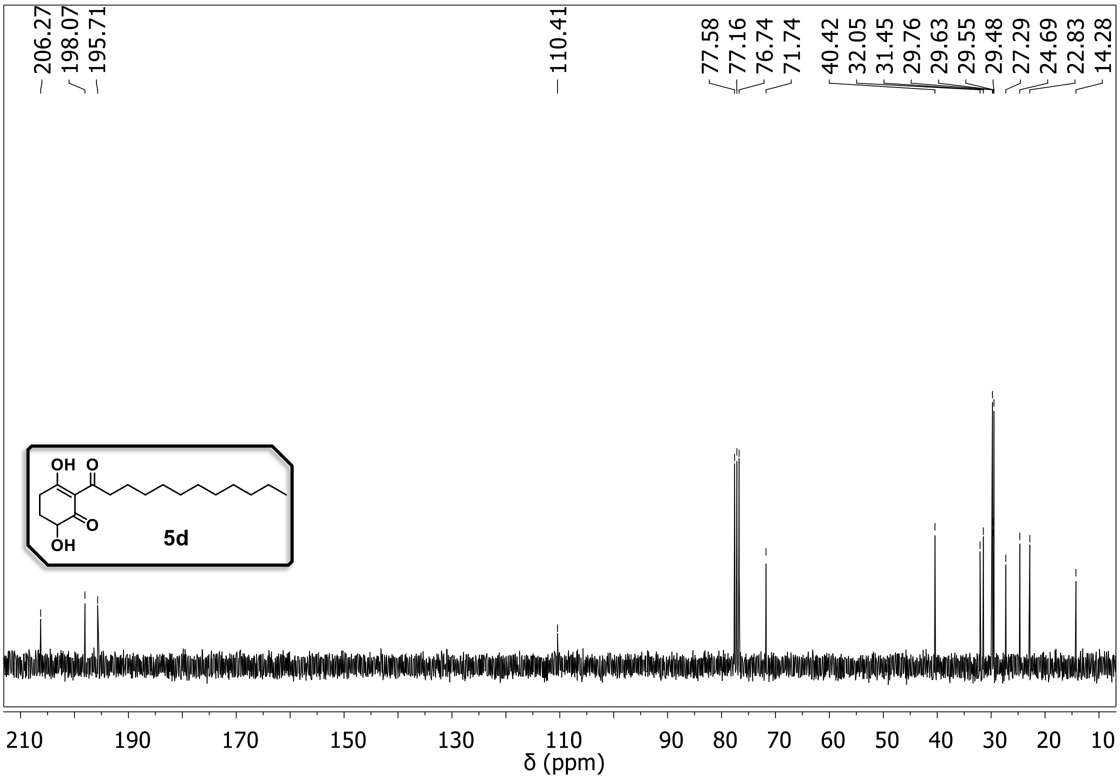

Figure S55. ^{13}C NMR (75 MHZ, $CDCl_3$) spectrum of **5d**.

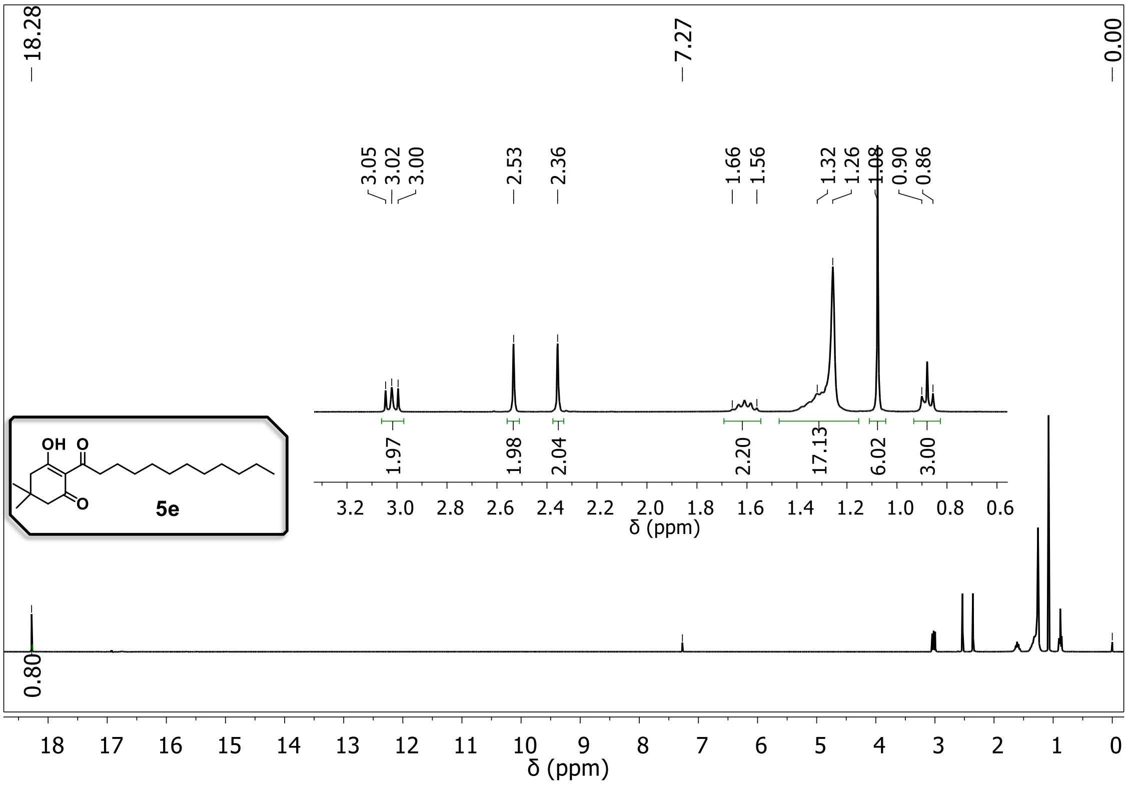

Figure S56. ^{1}H NMR (300 MHz, $CDCl_3$) spectrum of **5e**.

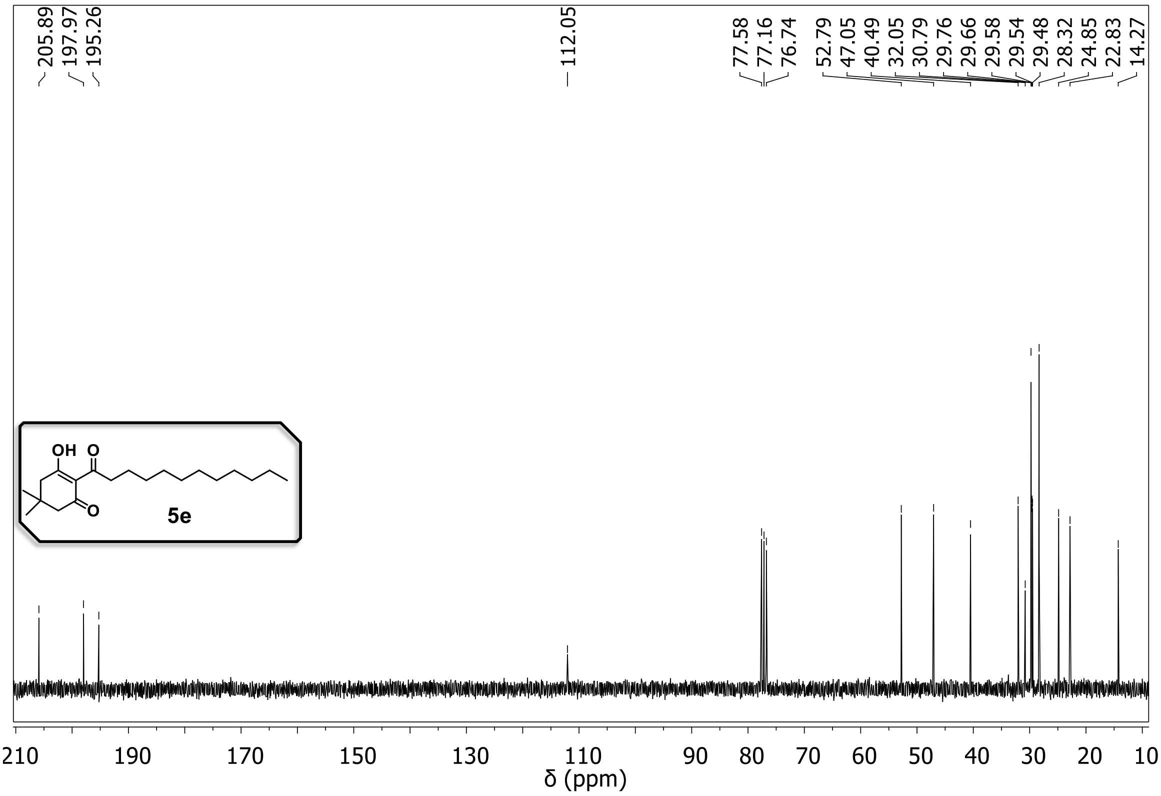

Figure S57. ^{13}C NMR (75 MHZ, $CDCl_3$) spectrum of **5e**.

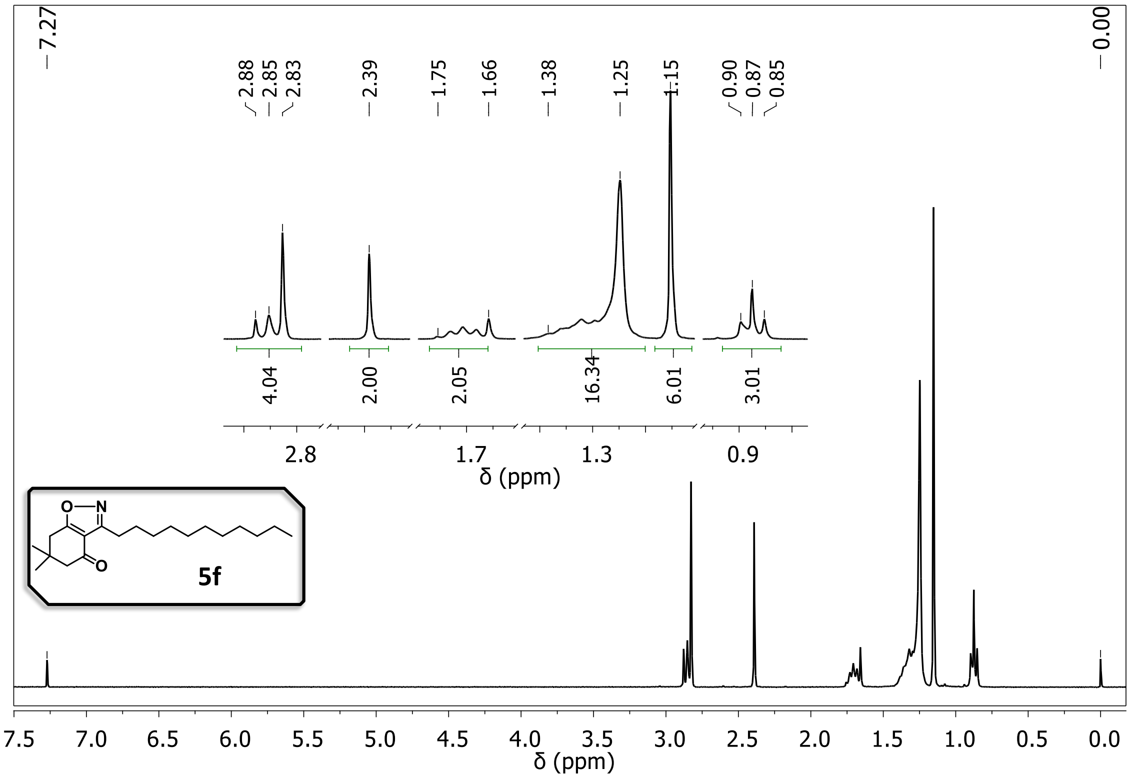

Figure S58. ^{1}H NMR (300 MHz, $CDCl_3$) spectrum of **5f**.

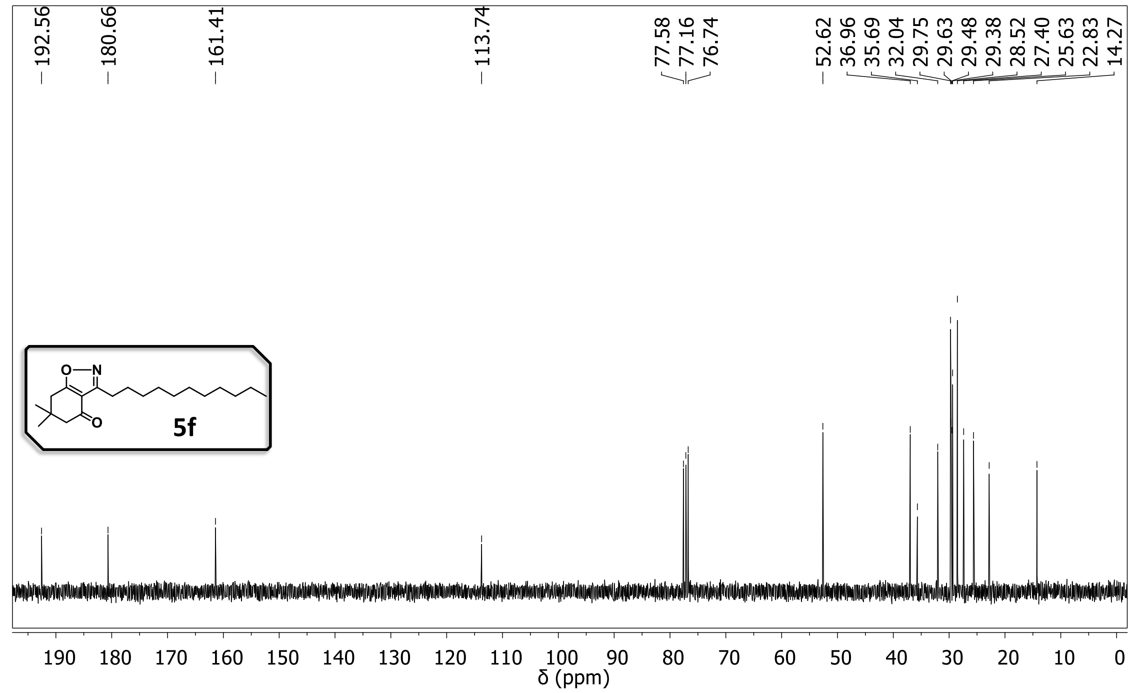

Figure S59. ^{13}C NMR (75 MHZ, $CDCl_3$) spectrum of **5f**.

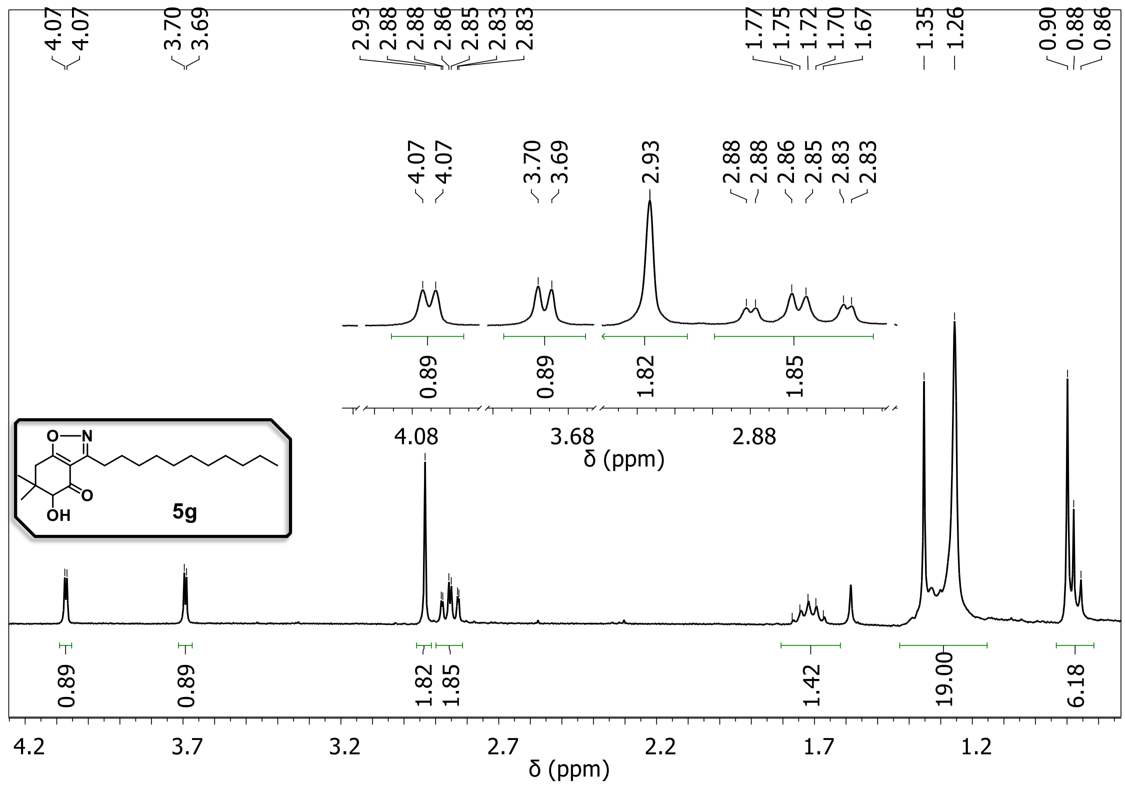

Figure S60. ^{1}H NMR (300 MHz, CDCl$_3$) spectrum of **5g**.

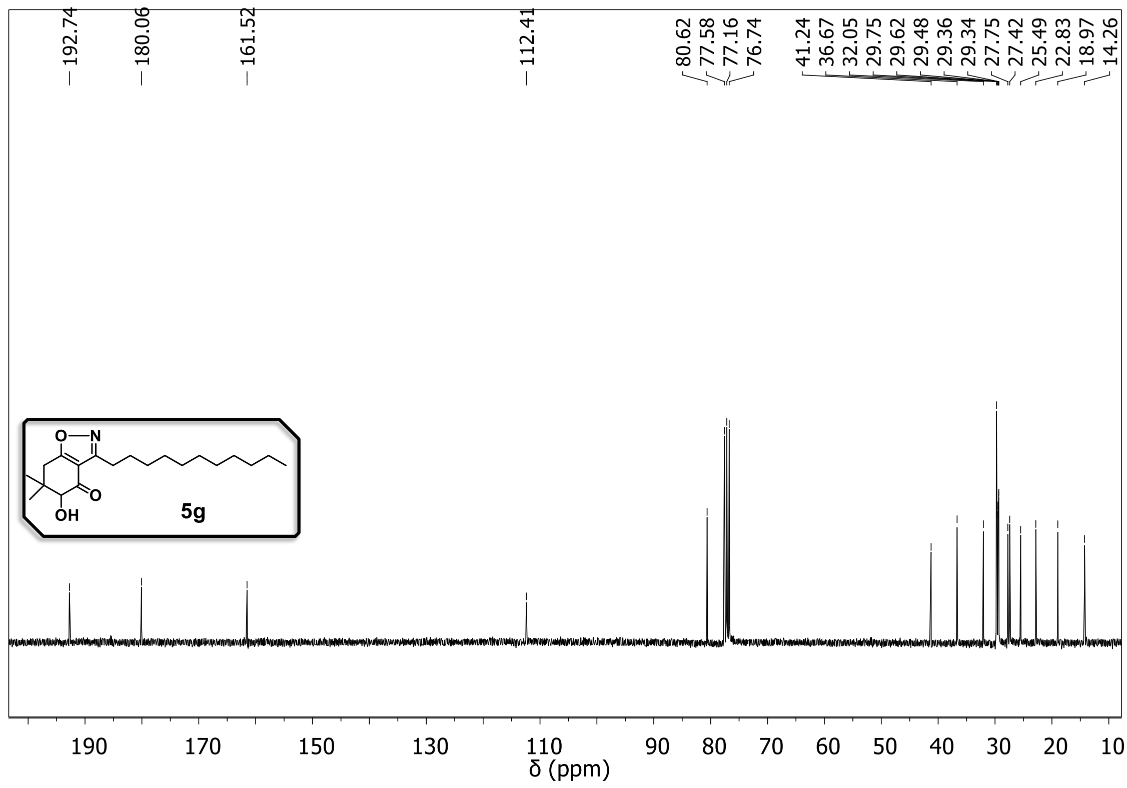

Figure S61. ^{13}C NMR (75 MHZ, CDCl$_3$) spectrum of **5g**.

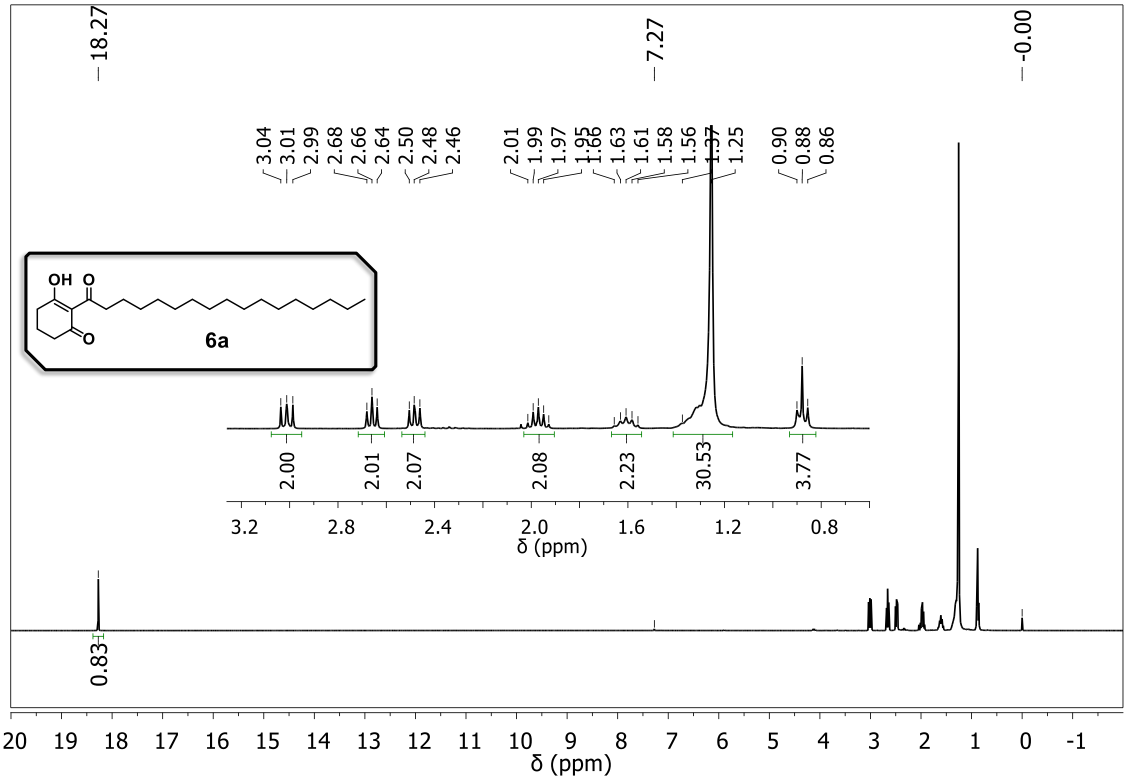

Figure S62. ^{1}H NMR (300 MHz, $CDCl_3$) spectrum of **6a**.

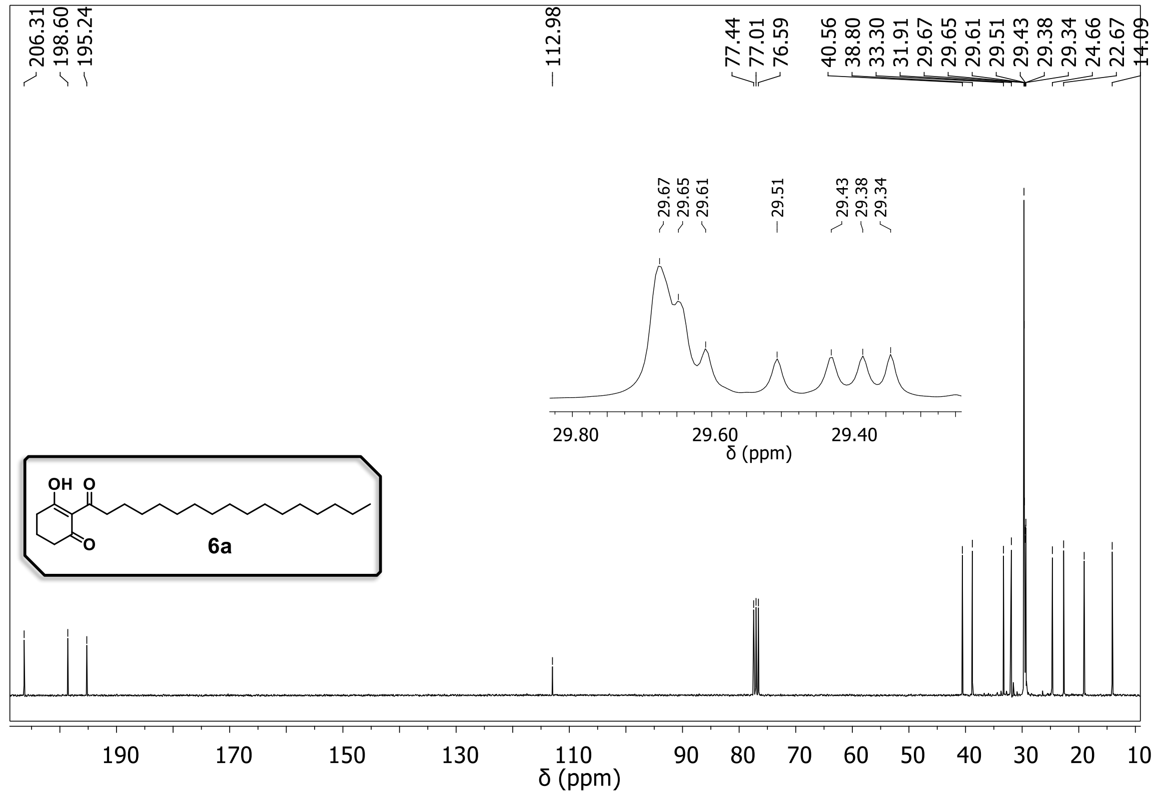

Figure S63. ^{13}C NMR (75 MHZ, $CDCl_3$) spectrum of **6a**.

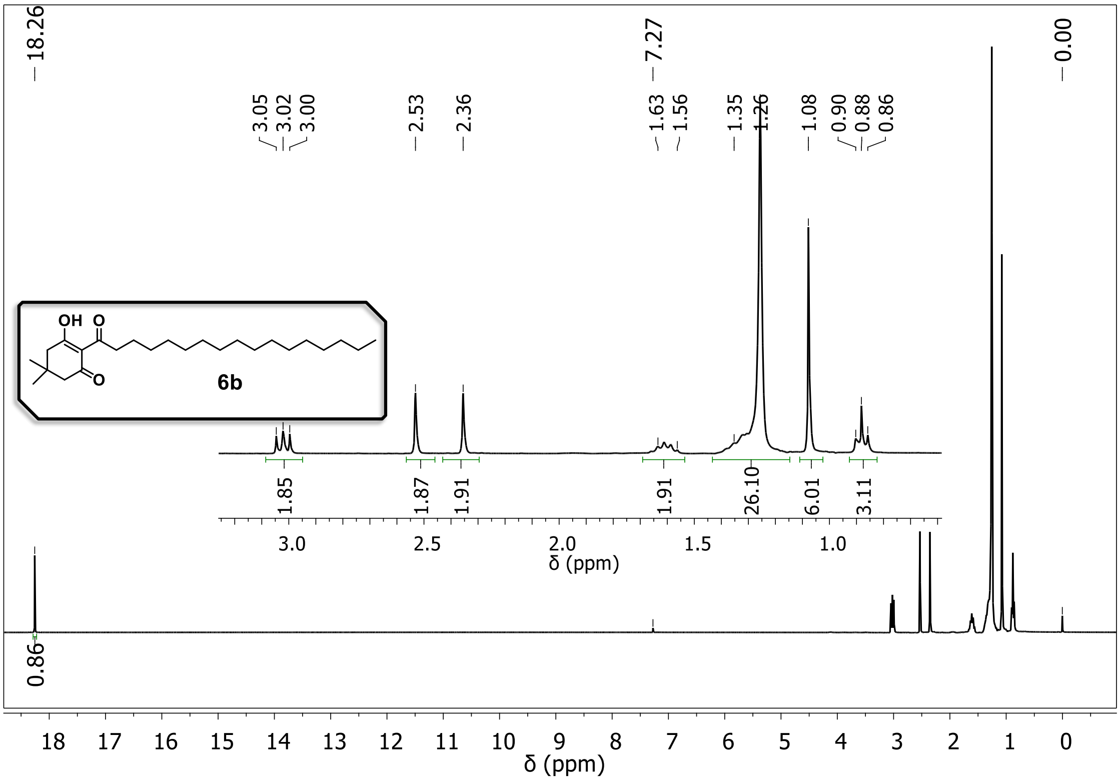

Figure S64. ^{1}H NMR (300 MHz, $CDCl_3$) spectrum of **6b**.

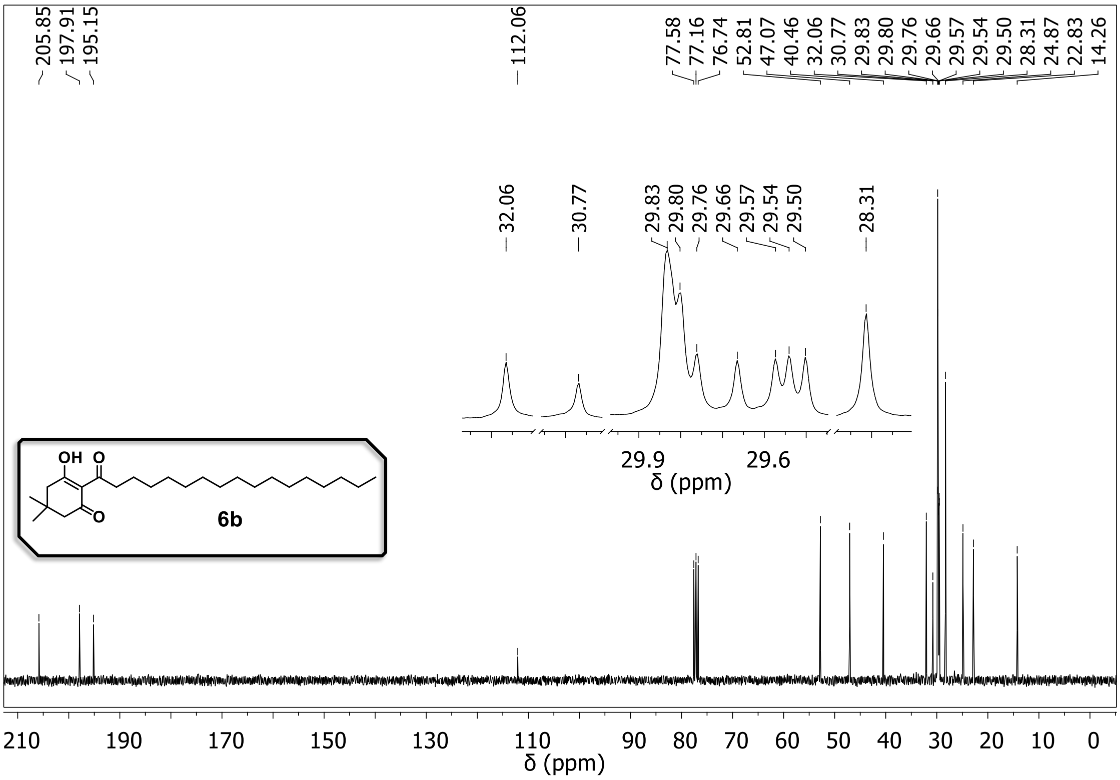

Figure S65. ^{13}C NMR (75 MHZ, $CDCl_3$) spectrum of **6b**.

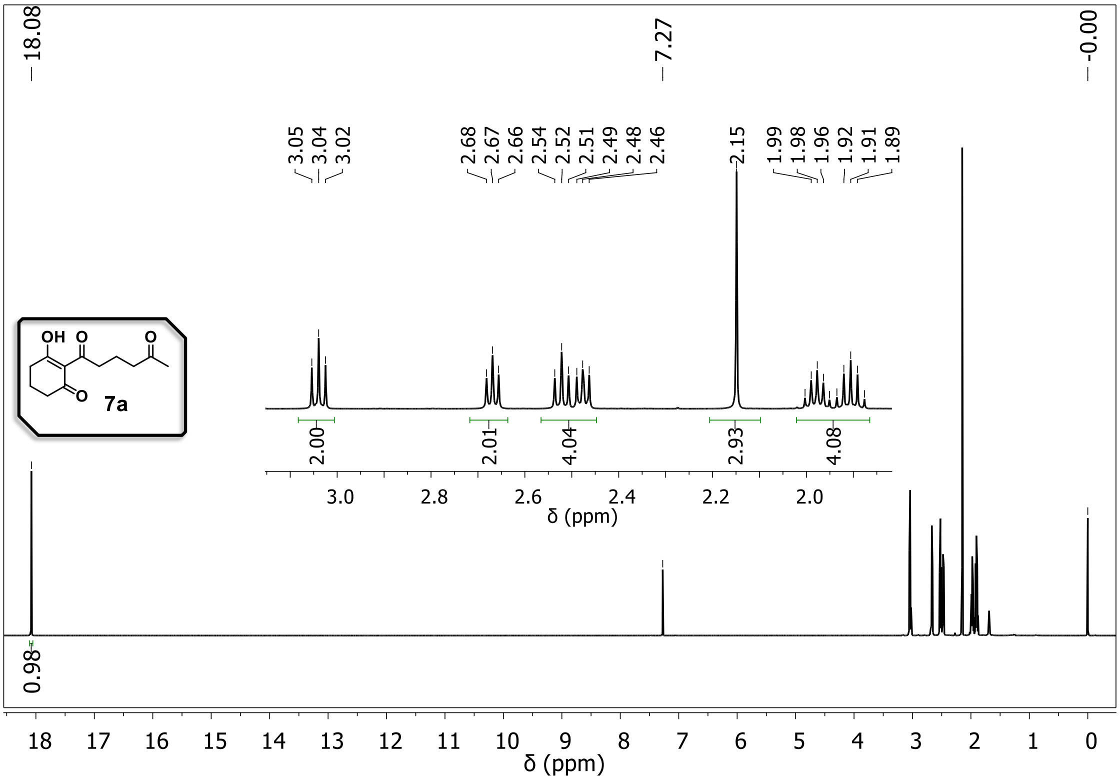

Figure S66. ^{1}H NMR (300 MHz, $CDCl_3$) spectrum of **7a**.

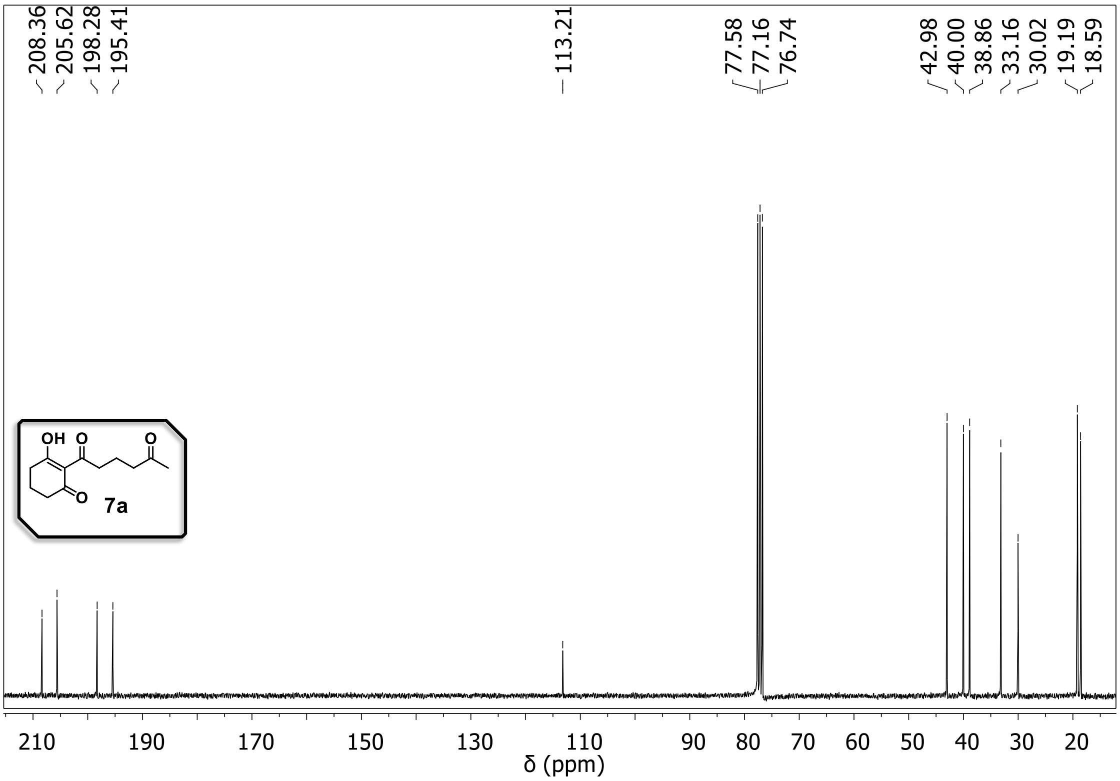

Figure S67. ^{13}C NMR (75 MHZ, $CDCl_3$) spectrum of **7a**.

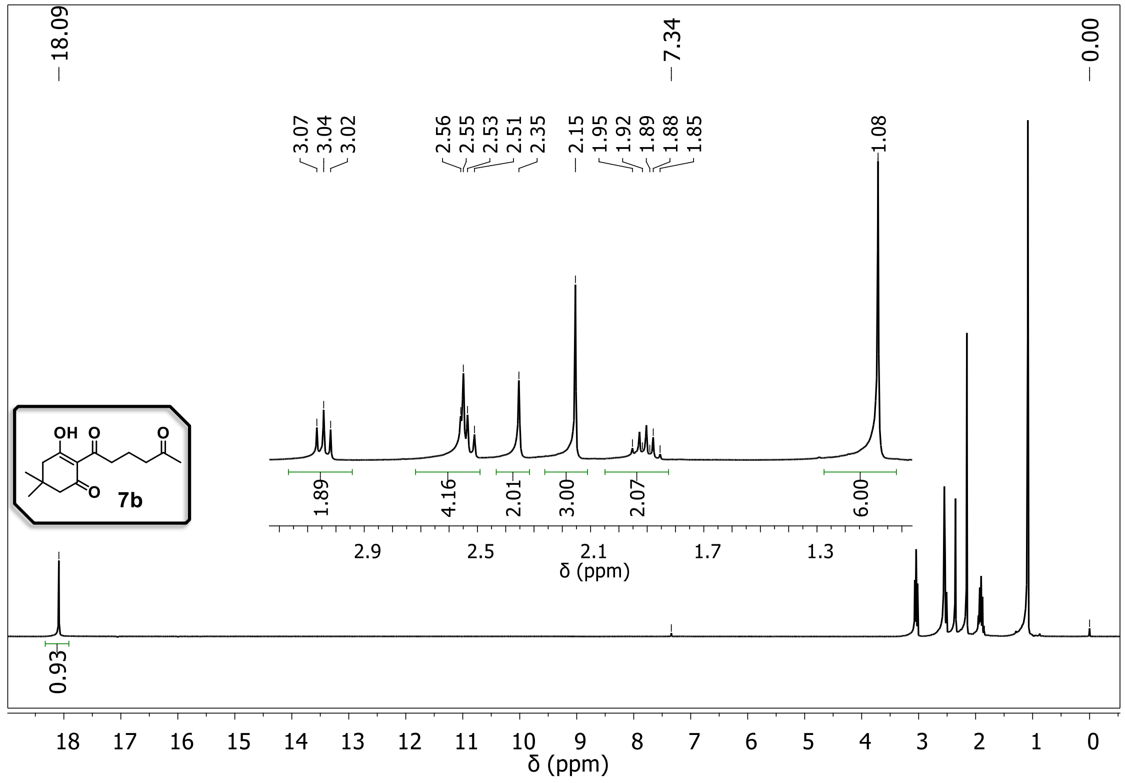

Figure S68. ^{1}H NMR (300 MHz, $CDCl_3$) spectrum of **7b**.

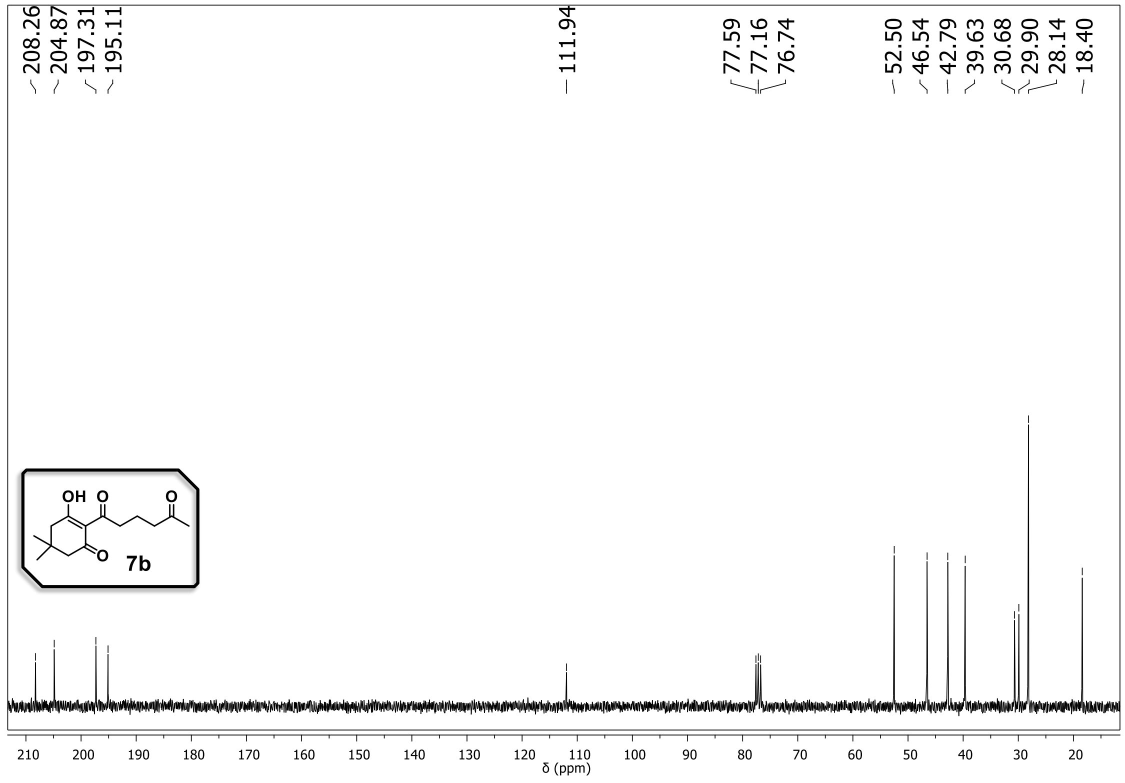

Figure S69. ^{13}C NMR (75 MHZ, $CDCl_3$) spectrum of **7b**.

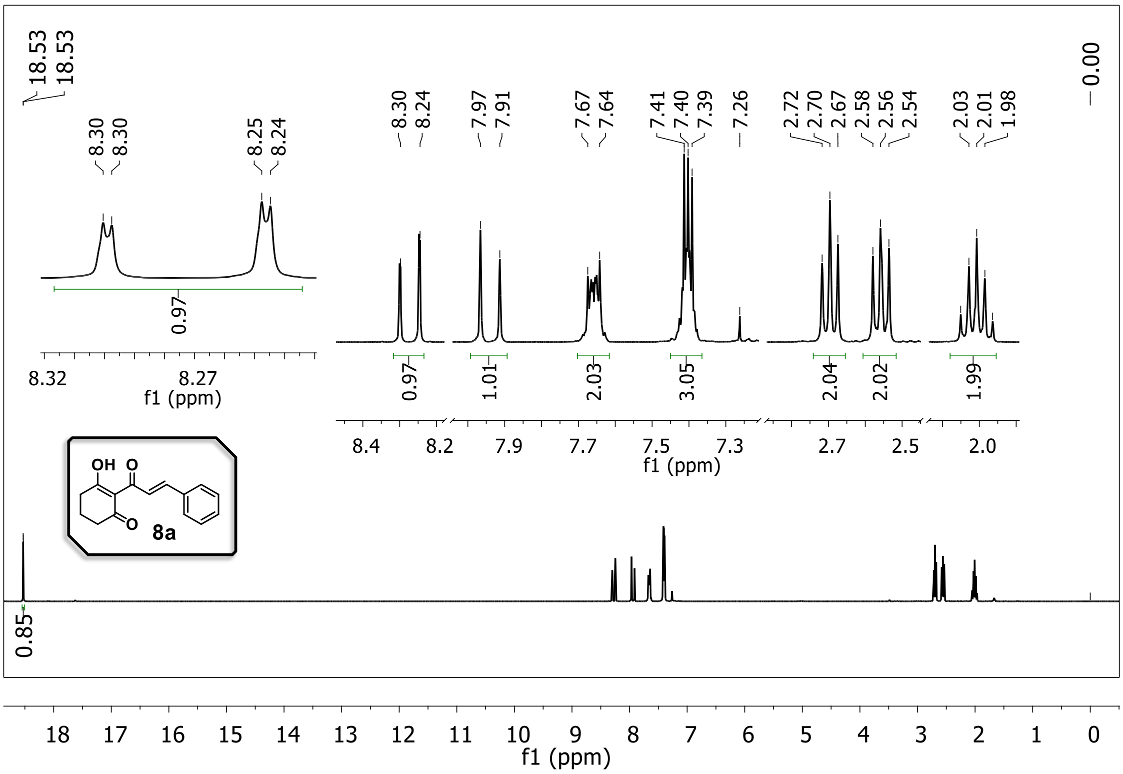

Figure S70. ^{1}H NMR (300 MHz, $CDCl_3$) spectrum of **8a**.

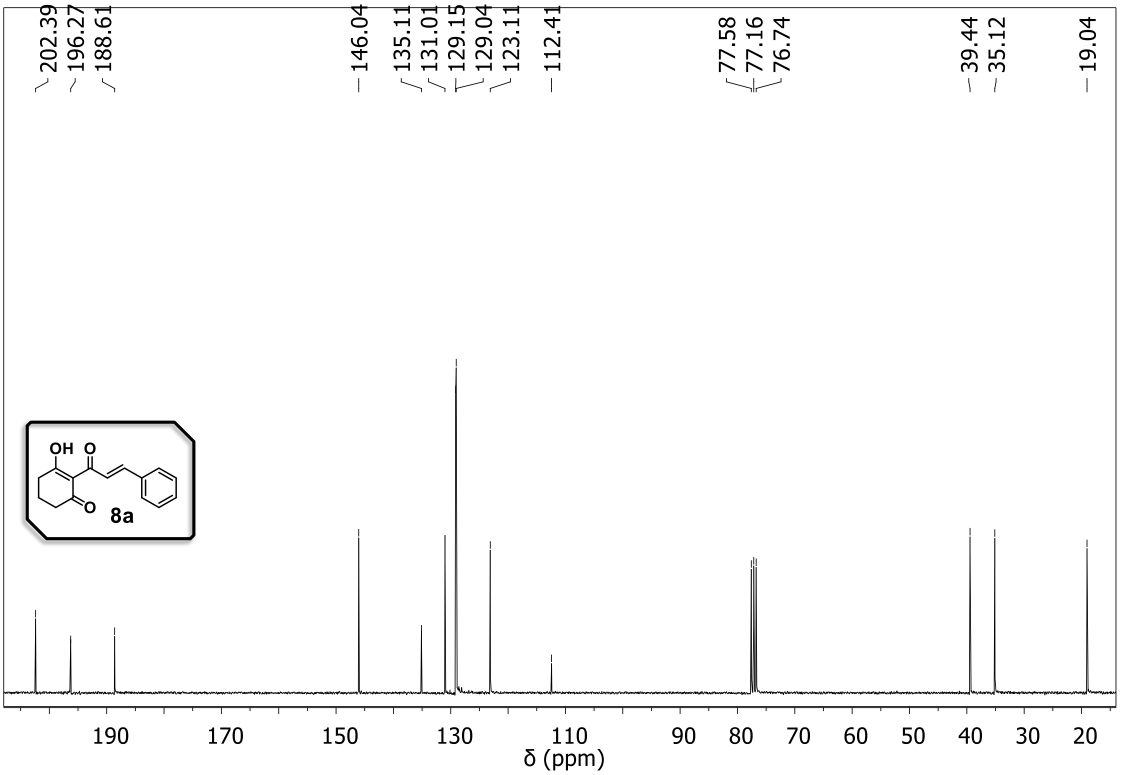

Figure S71. ^{13}C NMR (75 MHZ, $CDCl_3$) spectrum of **8a**.

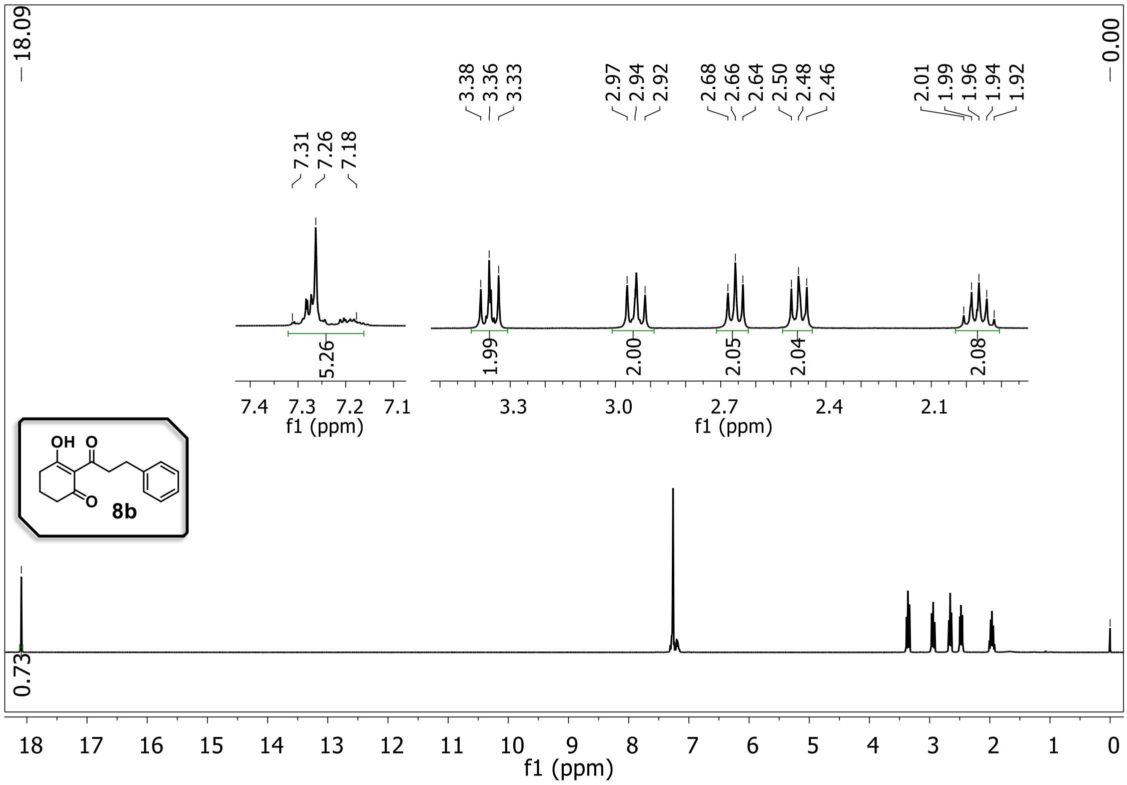

Figure S72. ^{1}H NMR (300 MHz, $CDCl_3$) spectrum of **8b**.

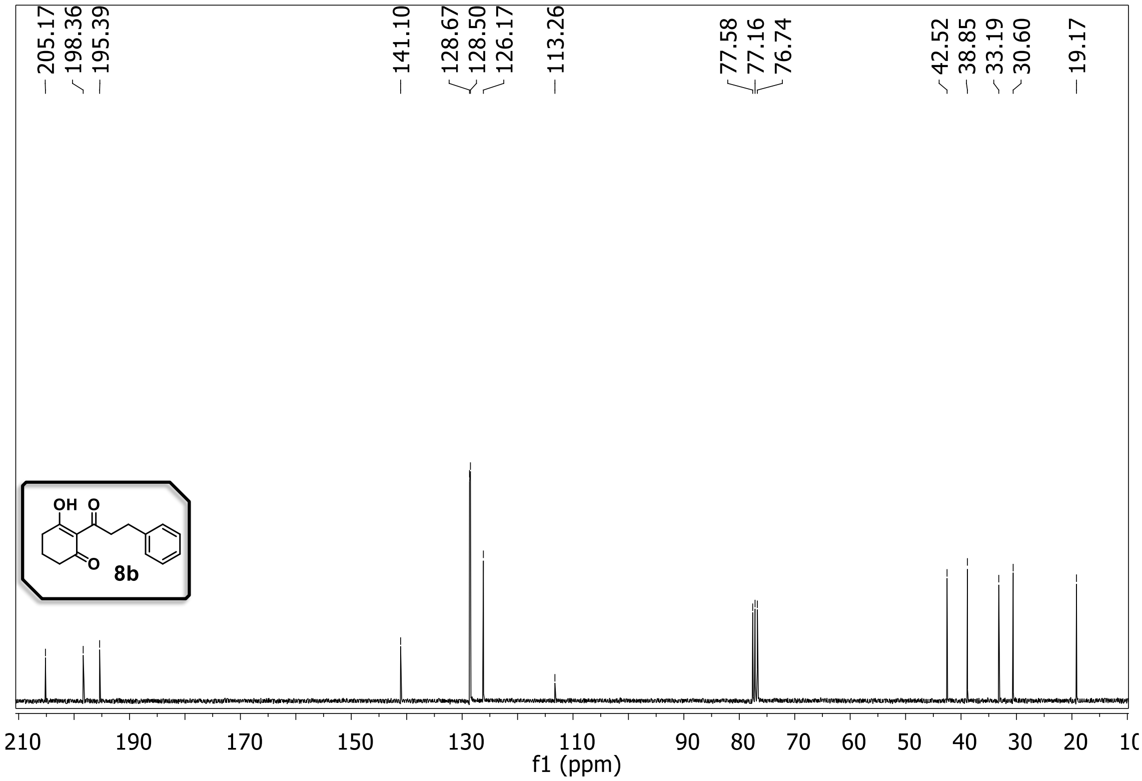

Figure S73. ^{13}C NMR (75 MHZ, $CDCl_3$) spectrum of **8b**.

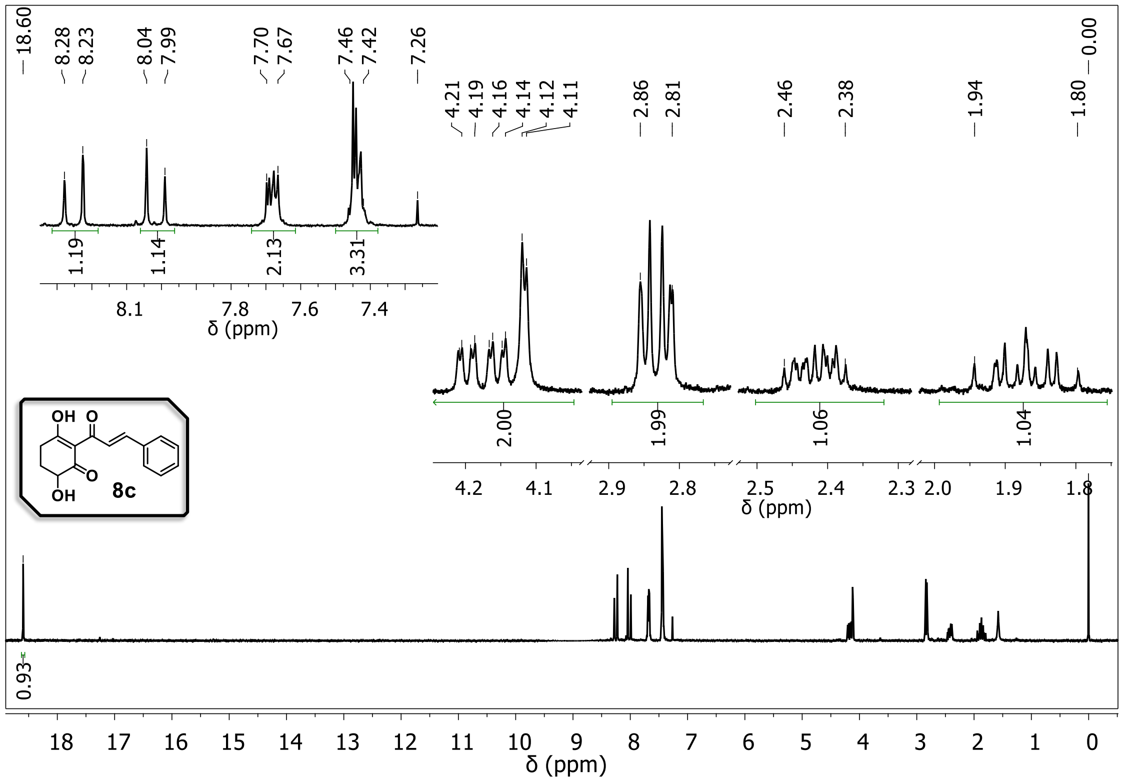

Figure S74. ^{1}H NMR (300 MHz, $CDCl_3$) spectrum of **8c**.

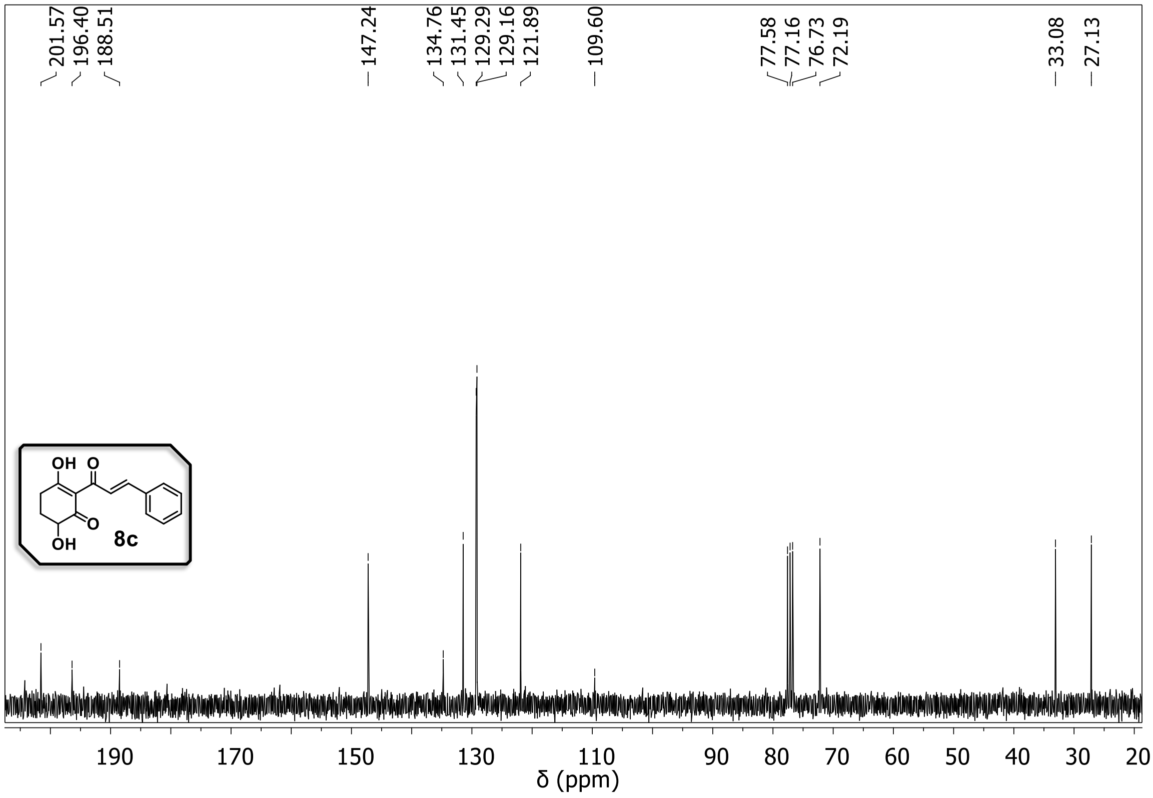

Figure S75. ^{13}C NMR (75 MHZ, $CDCl_3$) spectrum of **8c**.

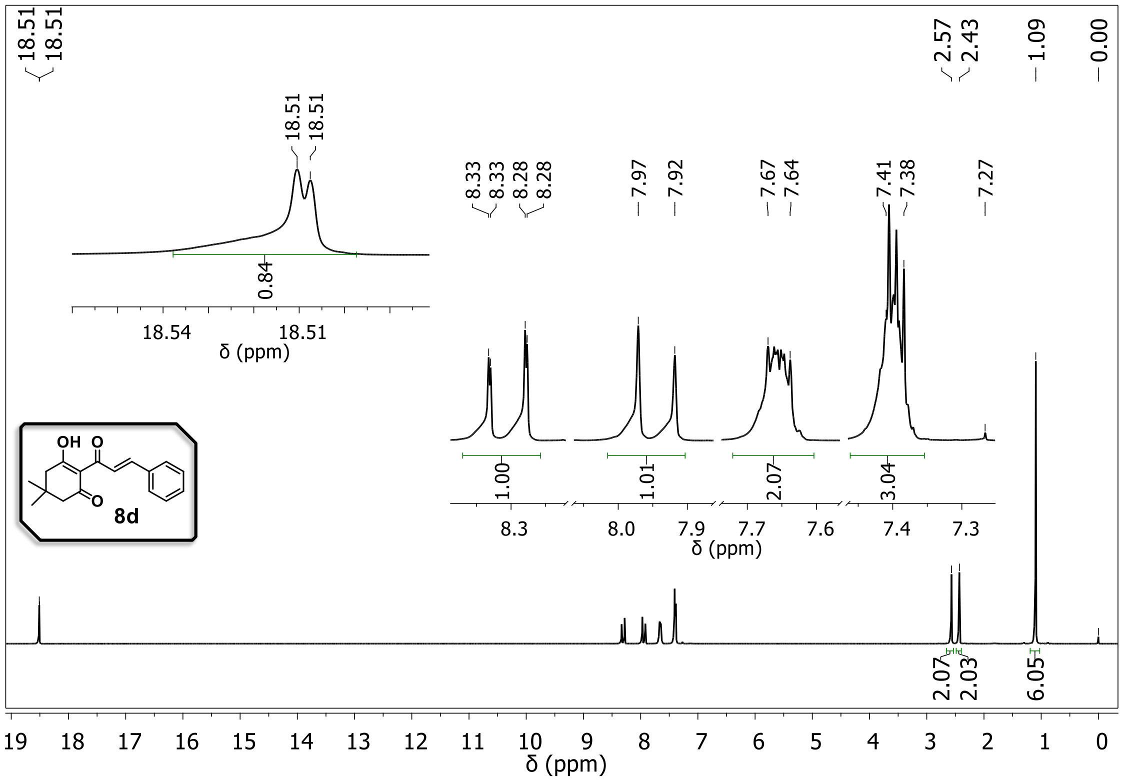

Figure S76. ^{1}H NMR (300 MHz, $CDCl_3$) spectrum of **8d**.

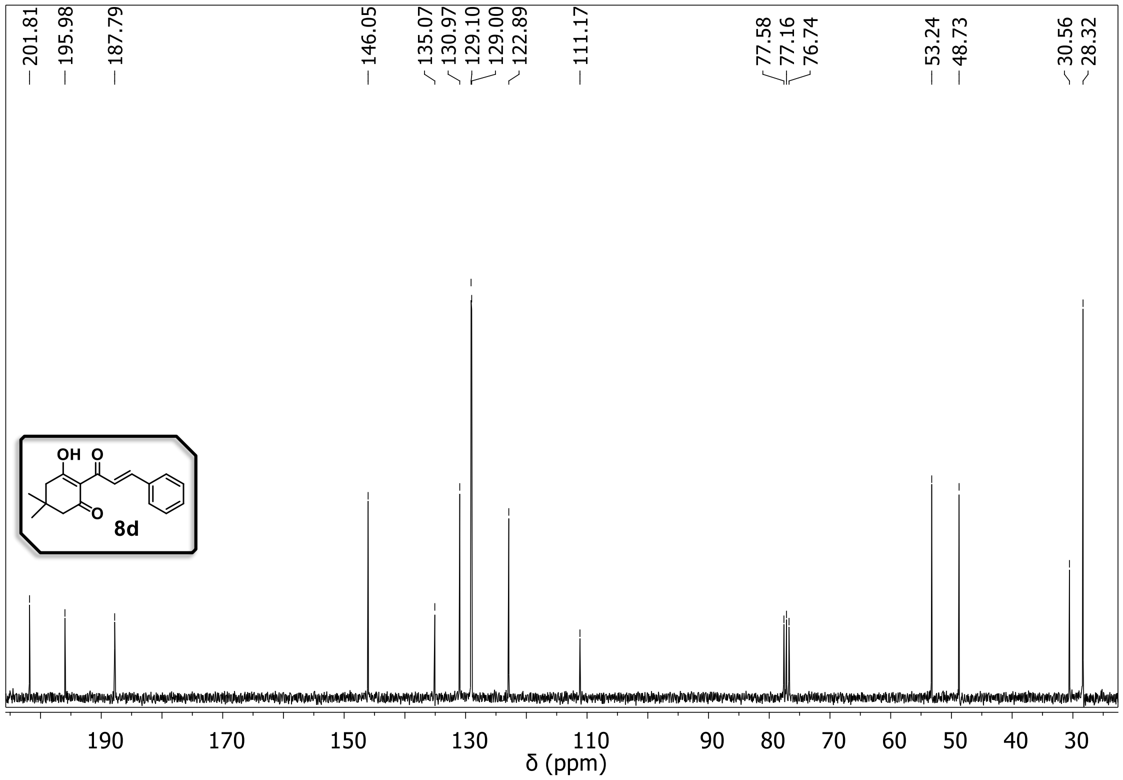

Figure S77. ^{13}C NMR (75 MHZ, $CDCl_3$) spectrum of **8d**.

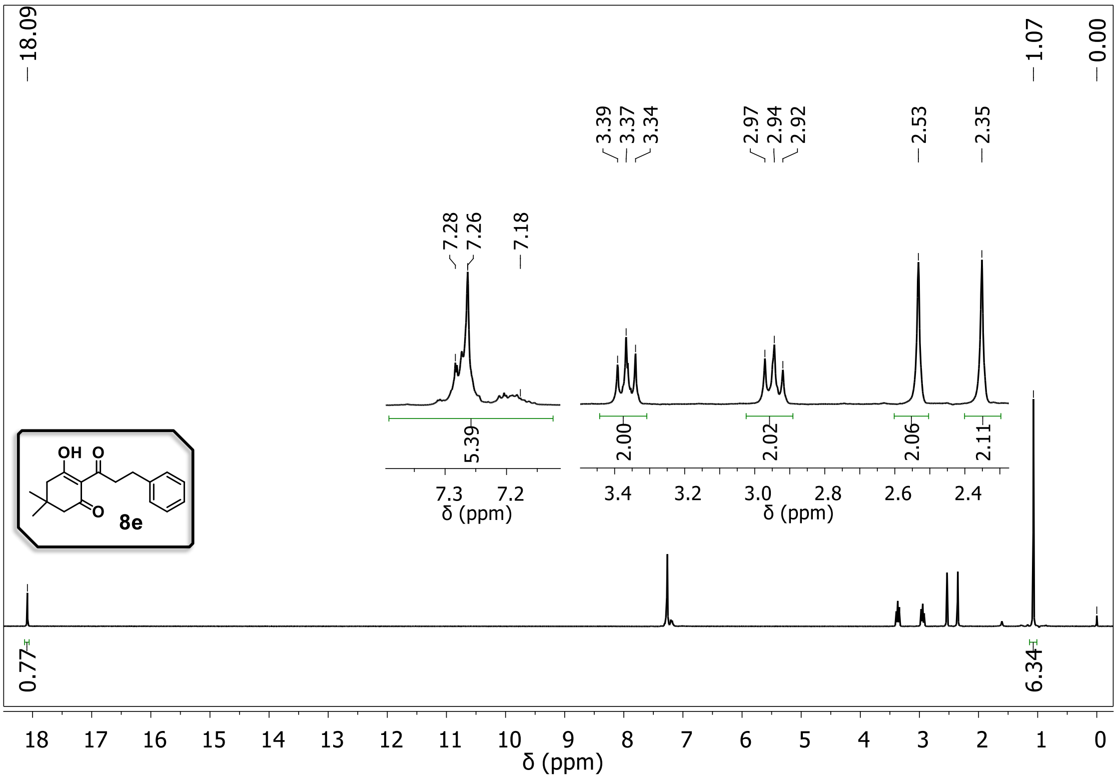

Figure S78. ^{1}H NMR (300 MHz, $CDCl_3$) spectrum of **8e**.

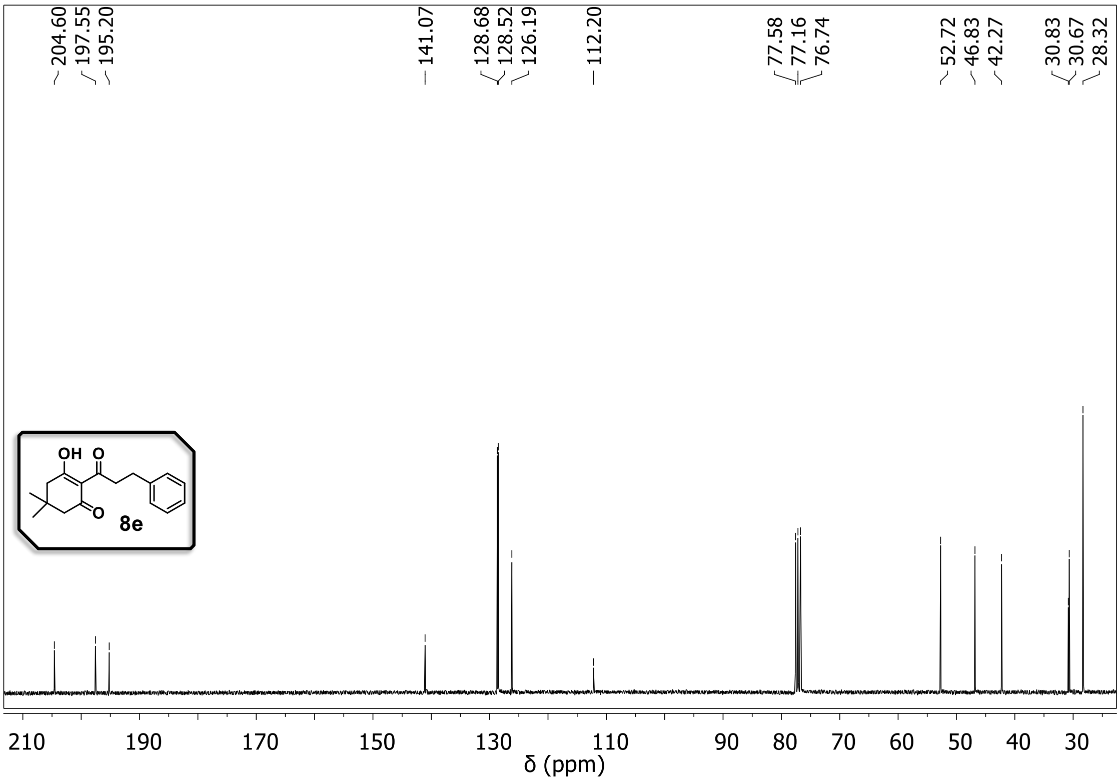

Figure S79. ^{13}C NMR (75 MHZ, $CDCl_3$) spectrum of **8e**.

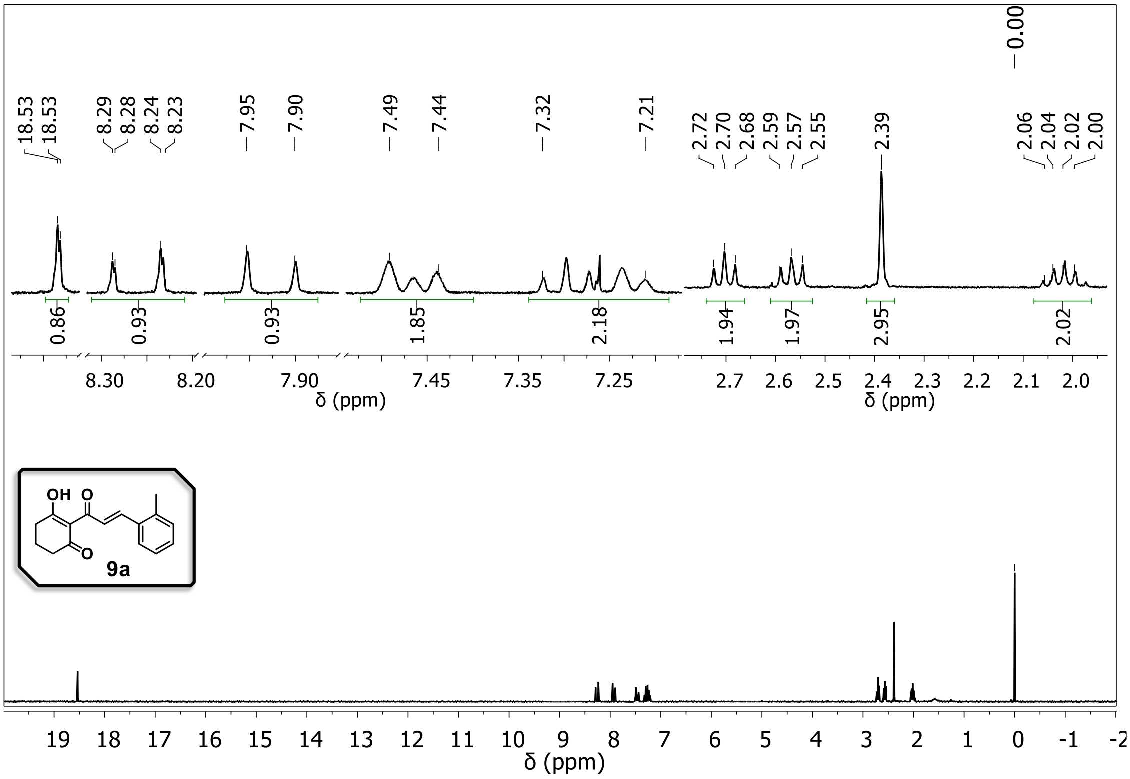

Figure S80. ^{1}H NMR (300 MHz, $CDCl_3$) spectrum of **9a**.

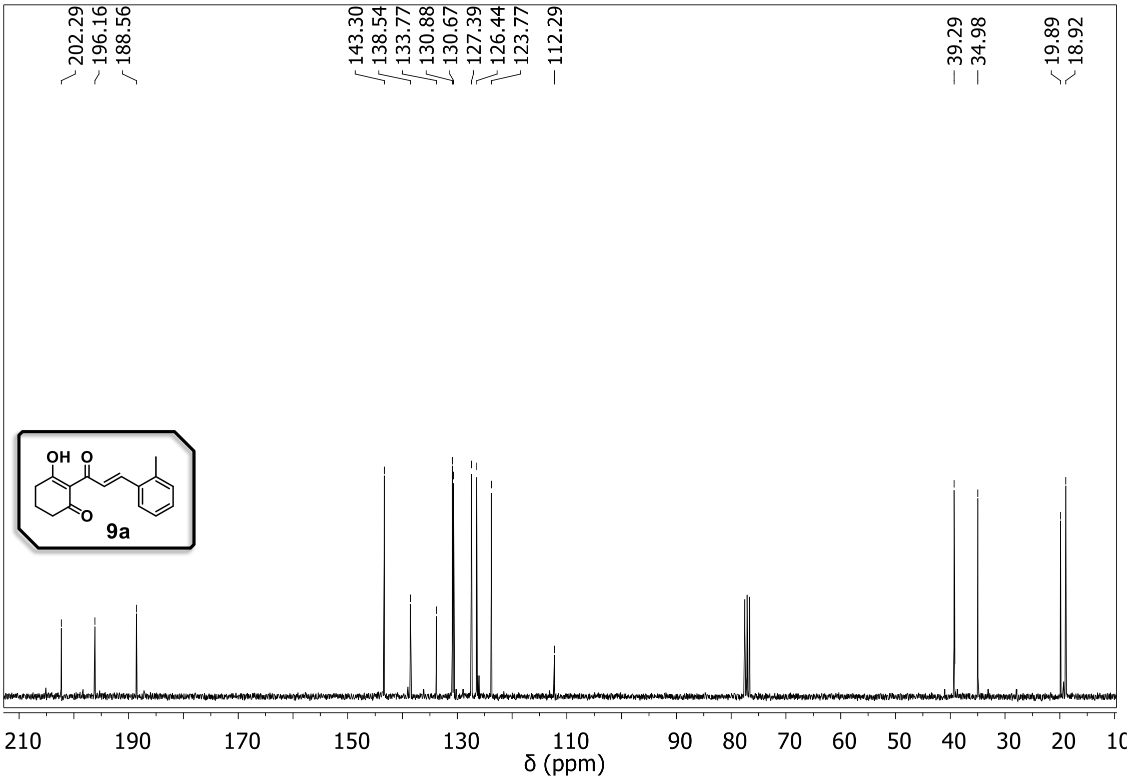

Figure S81. ^{13}C NMR (75 MHZ, $CDCl_3$) spectrum of **9a**.

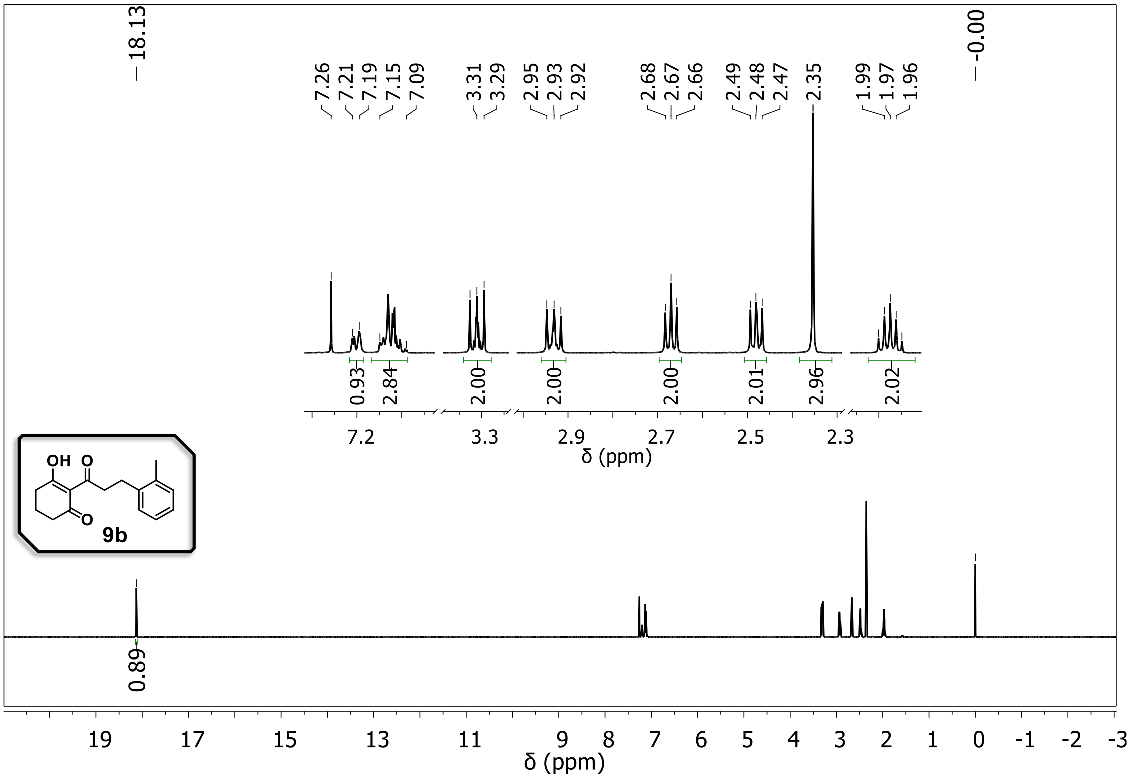

Figure S82. ^{1}H NMR (300 MHz, $CDCl_3$) spectrum of **9b**.

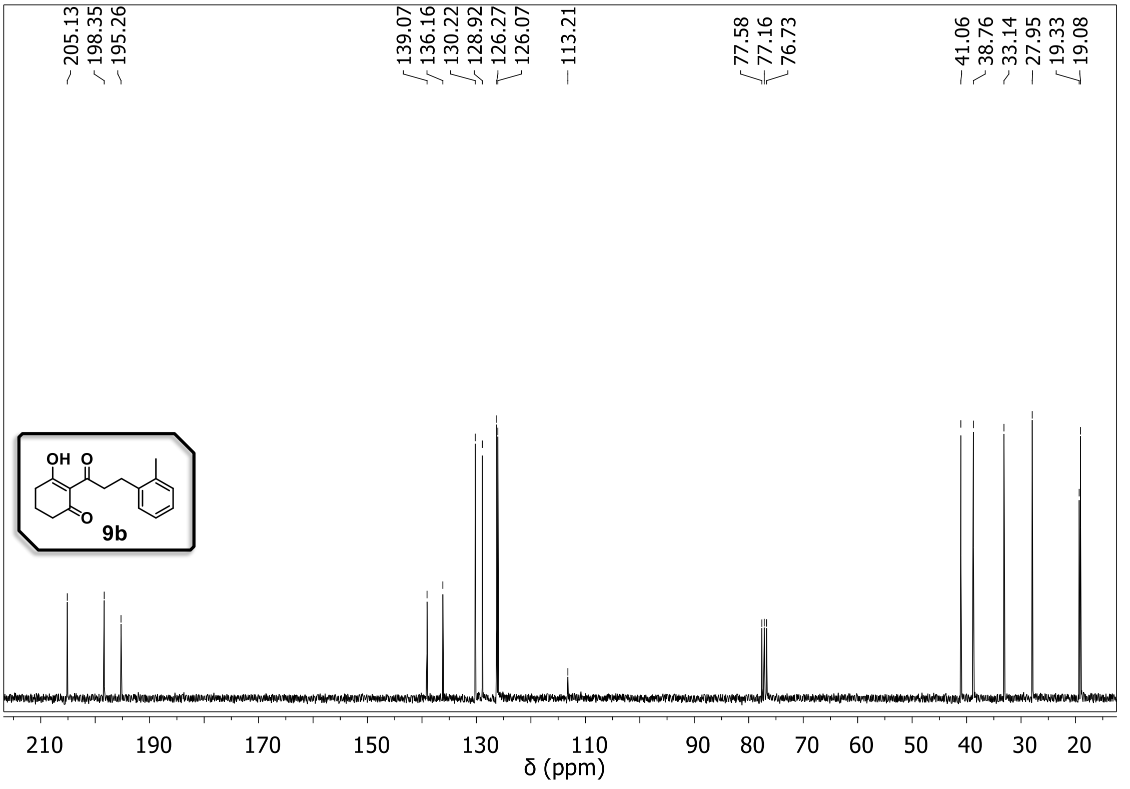

Figure S83. ^{13}C NMR (75 MHZ, $CDCl_3$) spectrum of **9b**.

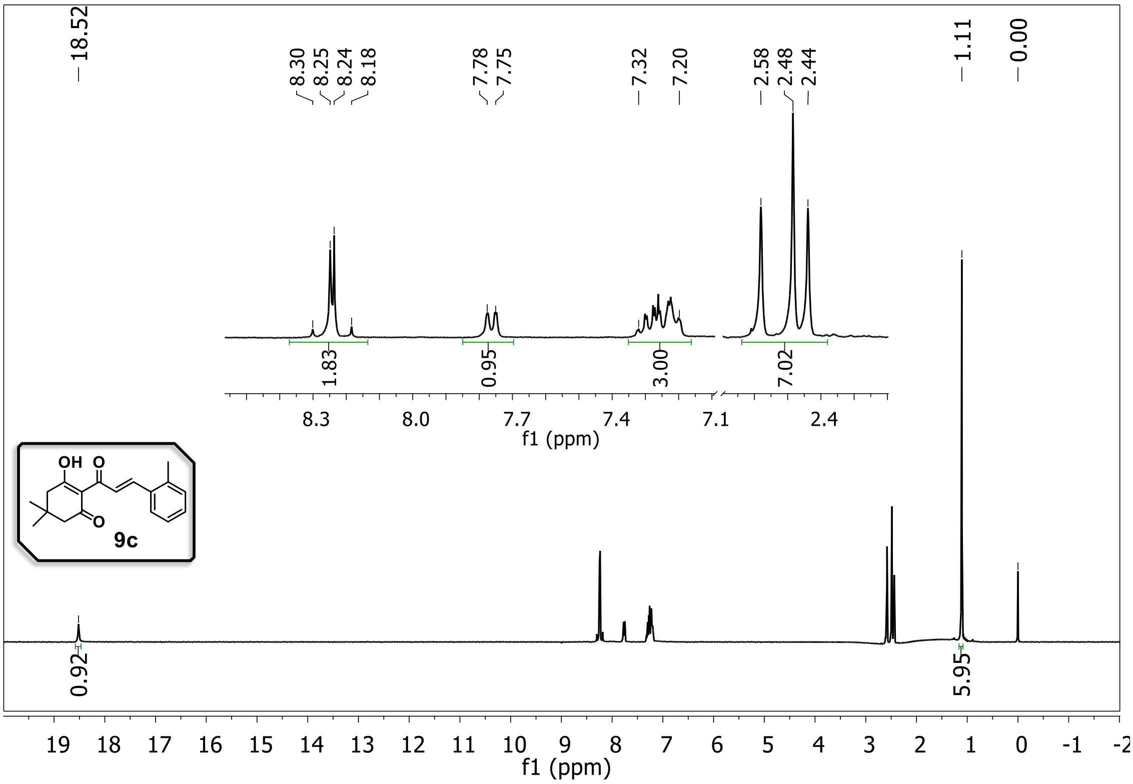

Figure S84. ^{1}H NMR (300 MHz, $CDCl_3$) spectrum of **9c**.

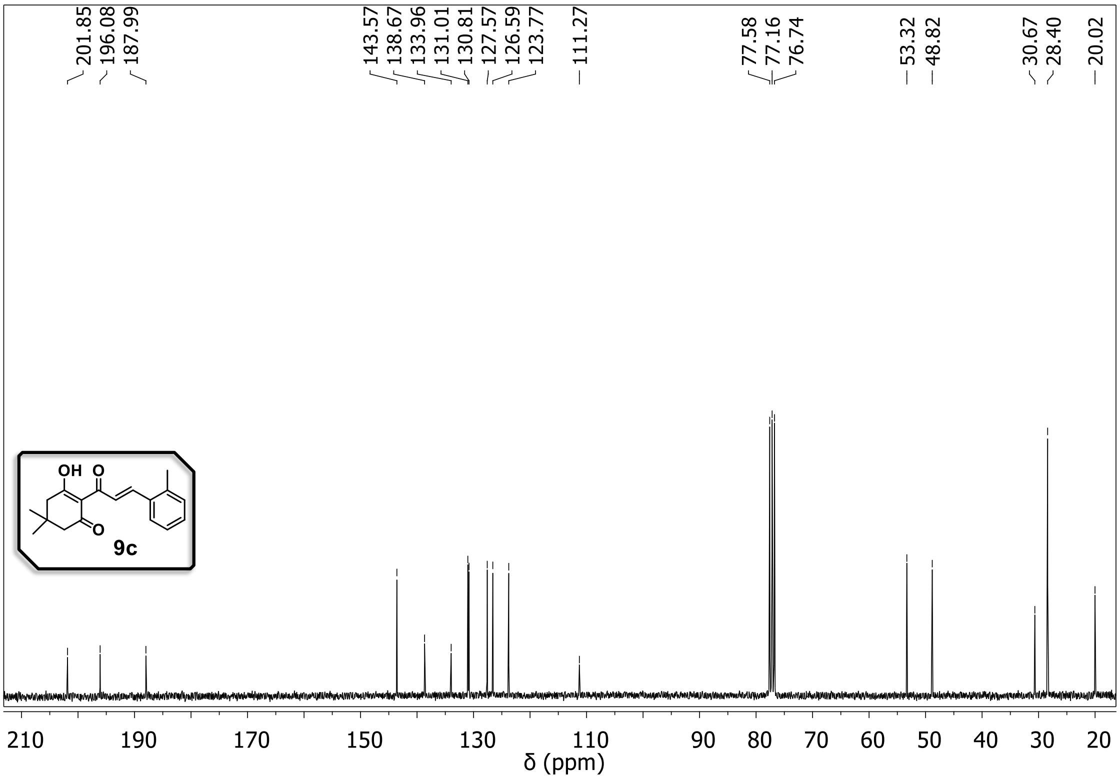

Figure S85. ^{13}C NMR (75 MHZ, $CDCl_3$) spectrum of **9c**.

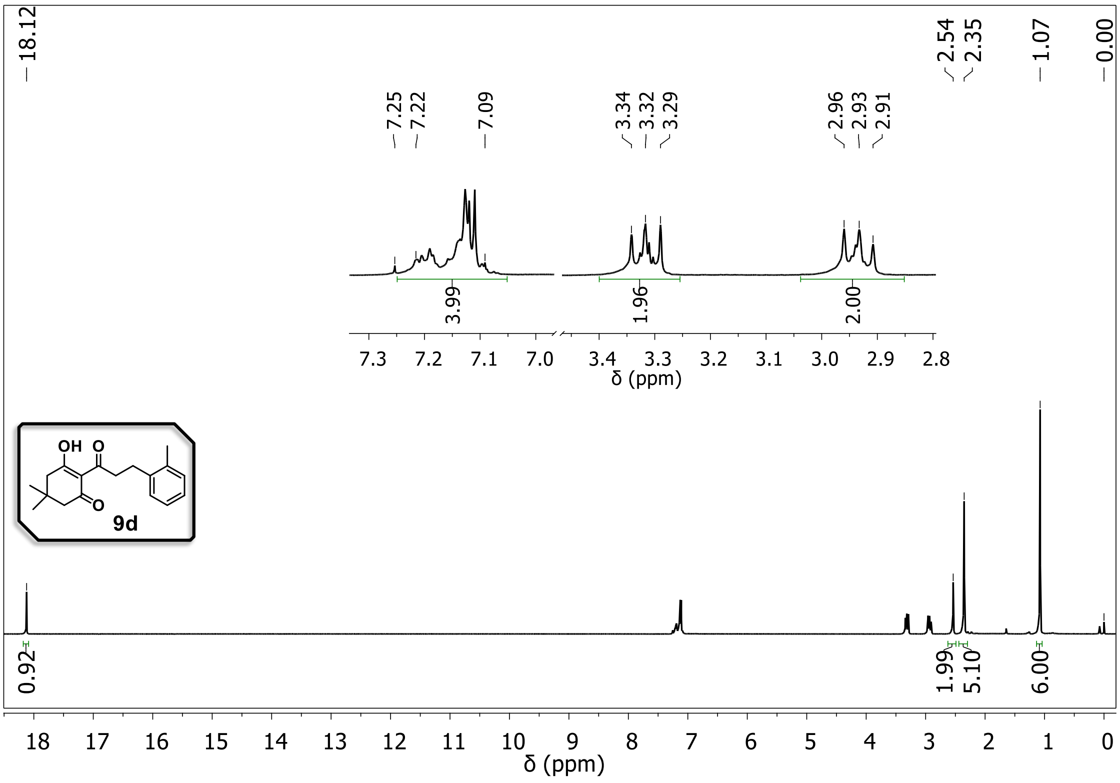

Figure S86. ^{1}H NMR (300 MHz, $CDCl_3$) spectrum of **9d**.

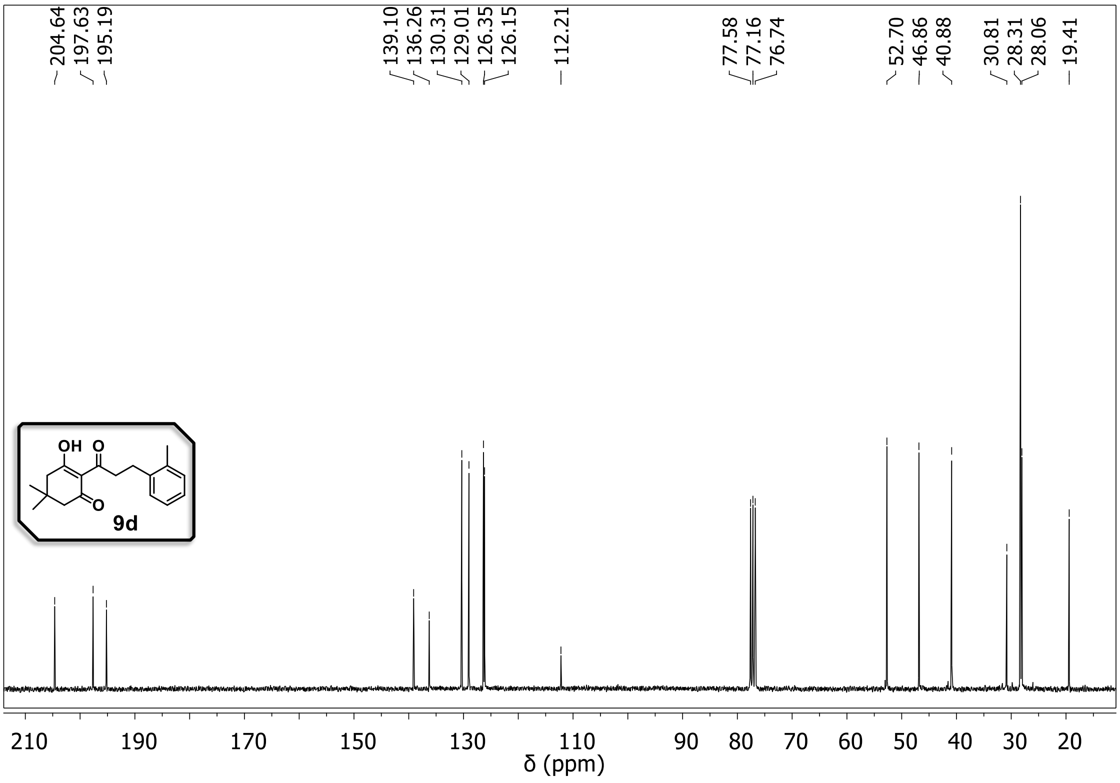

Figure S87. ^{13}C NMR (75 MHZ, $CDCl_3$) spectrum of **9d**.

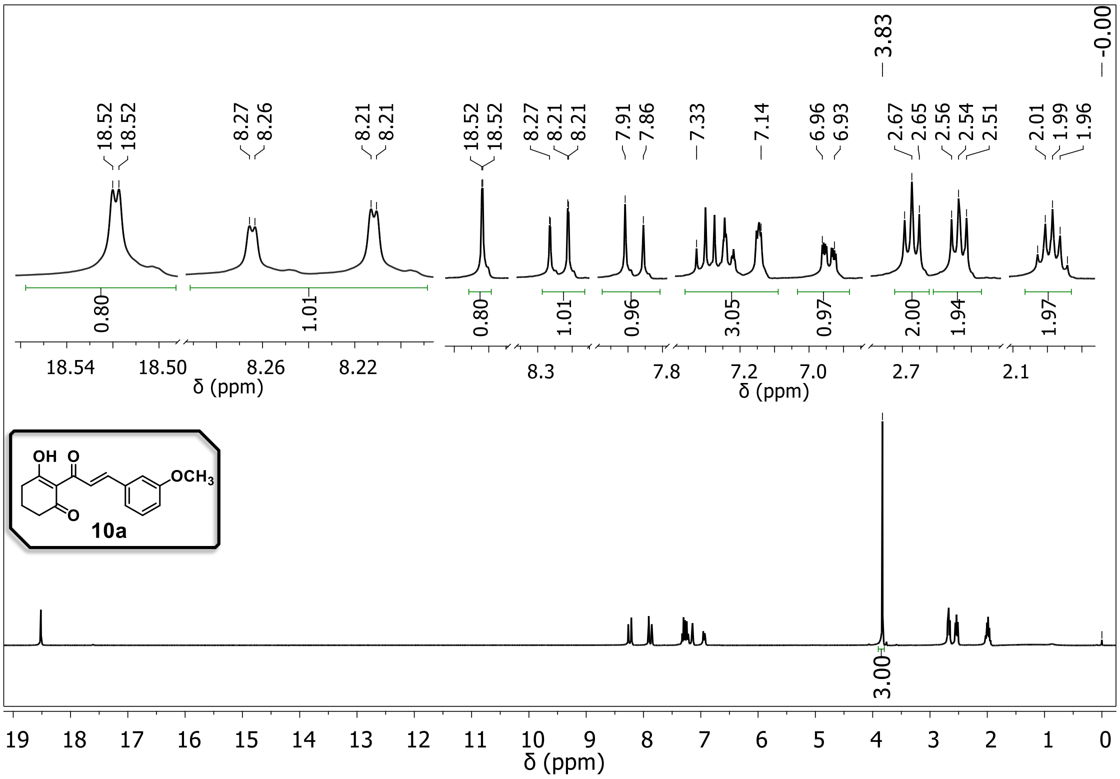

Figure S88. ^{1}H NMR (300 MHz, $CDCl_3$) spectrum of **10a**.

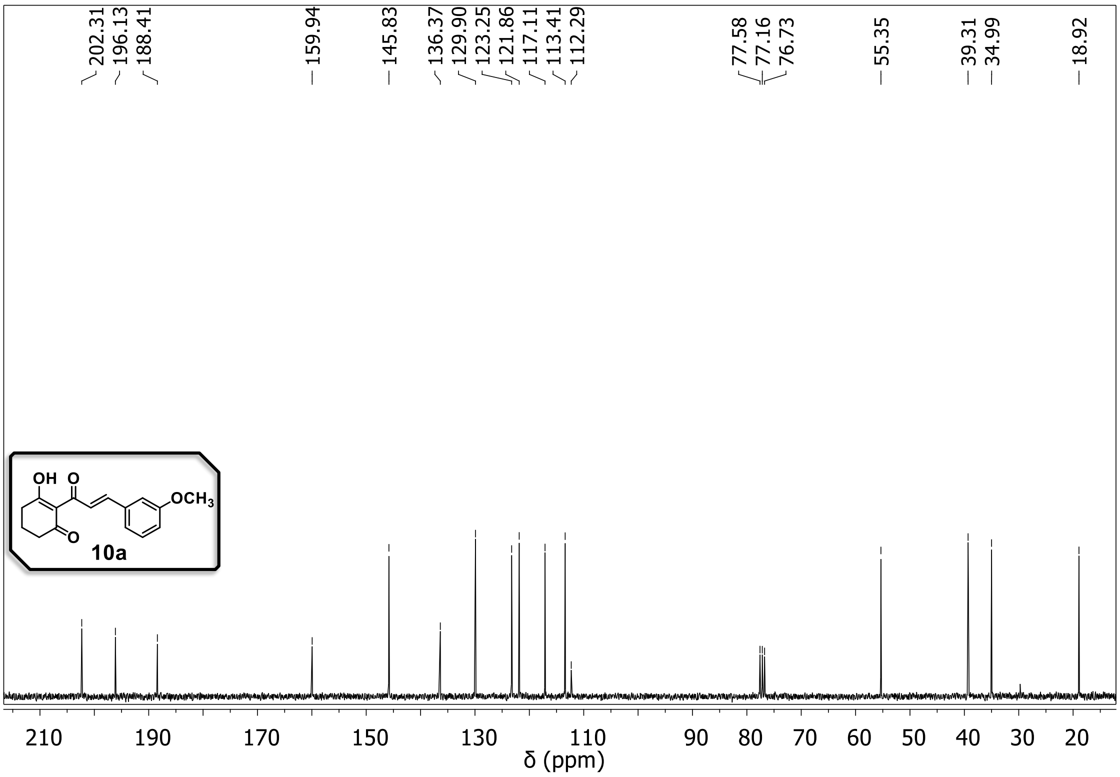

Figure S89. ^{13}C NMR (75 MHZ, $CDCl_3$) spectrum of **10a**.

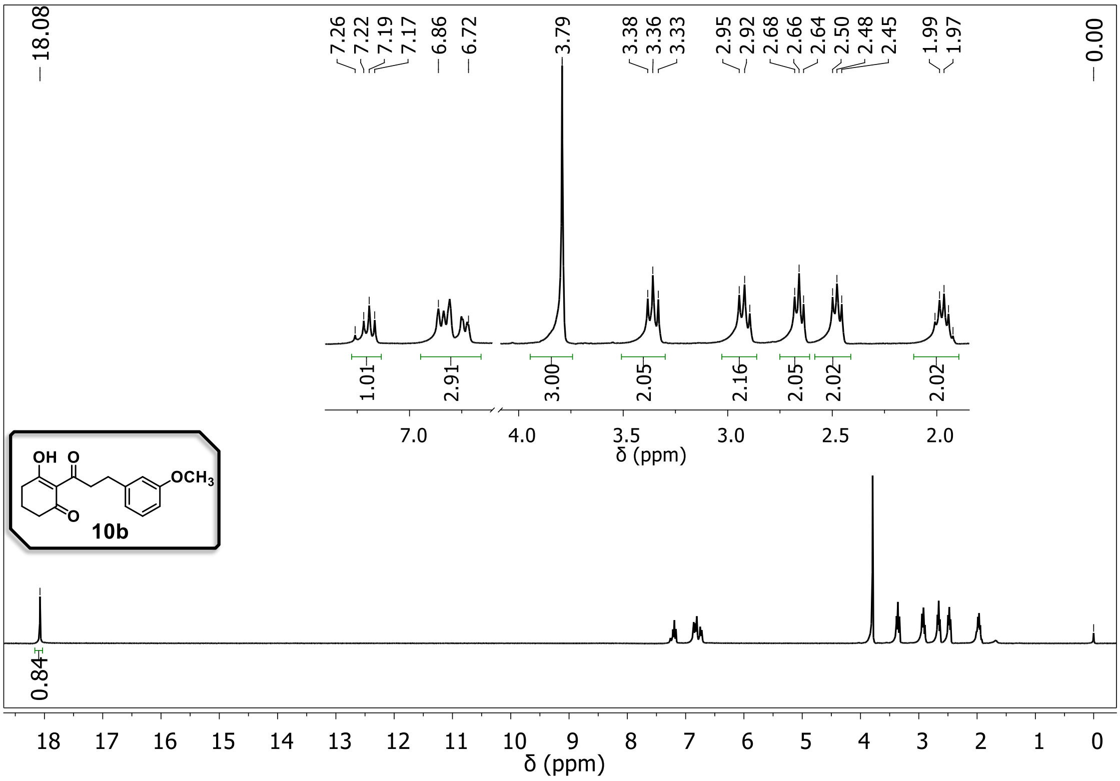

Figure S90. ^{1}H NMR (300 MHz, $CDCl_3$) spectrum of **10b**.

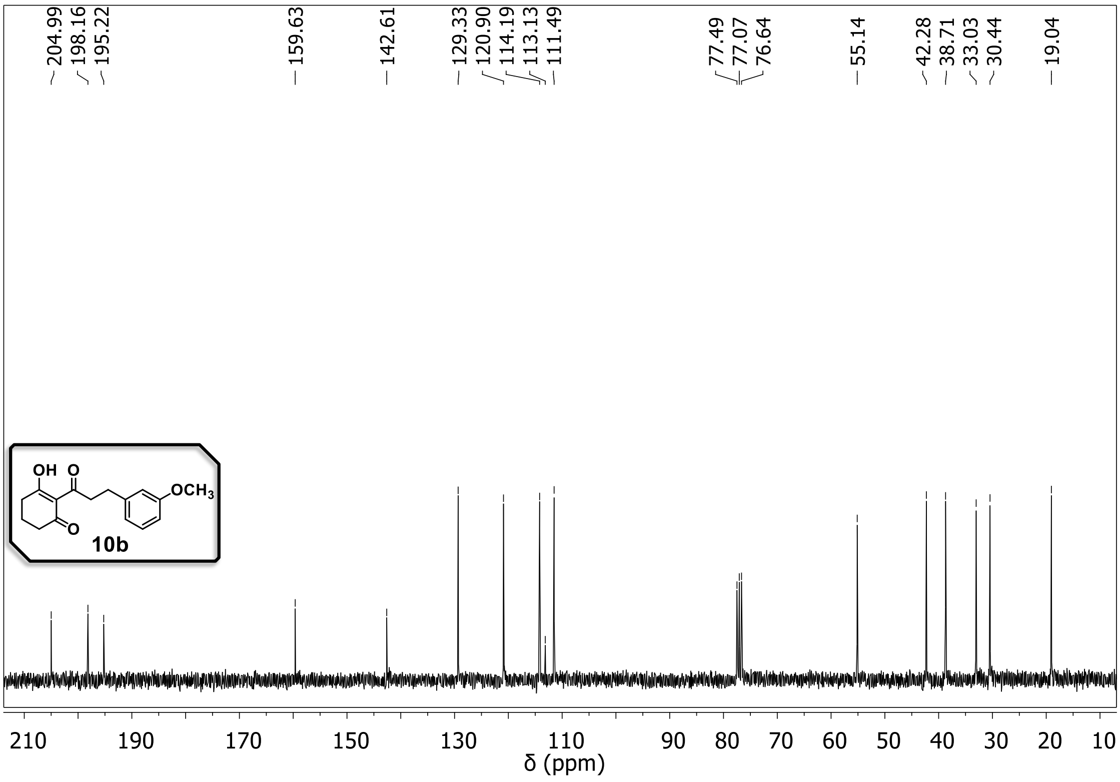

Figure S91. ^{13}C NMR (75 MHZ, $CDCl_3$) spectrum of **10b**.

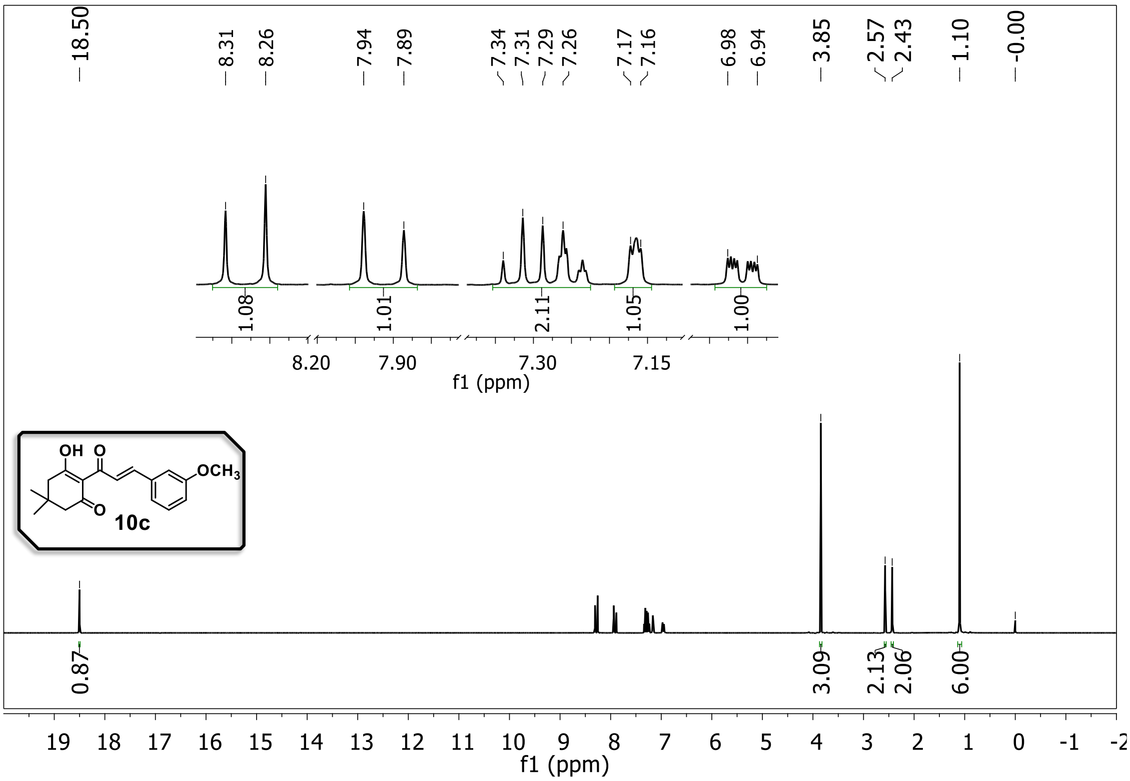

Figure S92. ^{1}H NMR (300 MHz, $CDCl_3$) spectrum of **10c**.

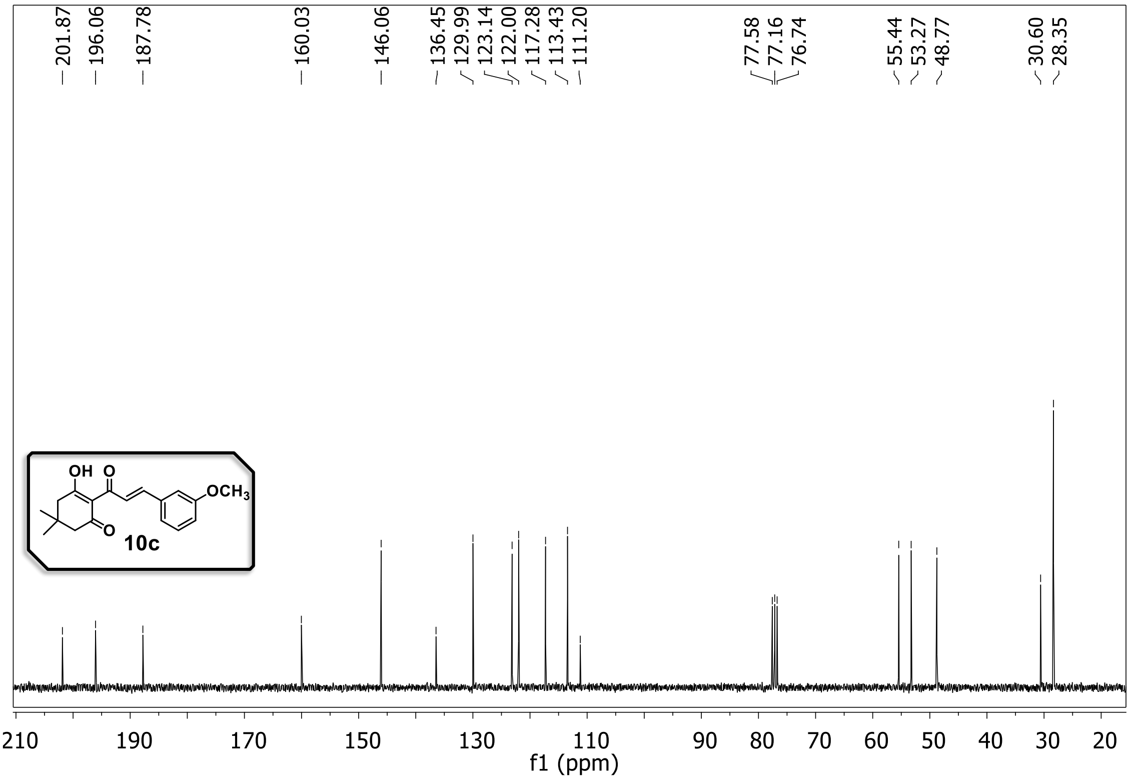

Figure S93. ^{13}C NMR (75 MHZ, $CDCl_3$) spectrum of **10c**.

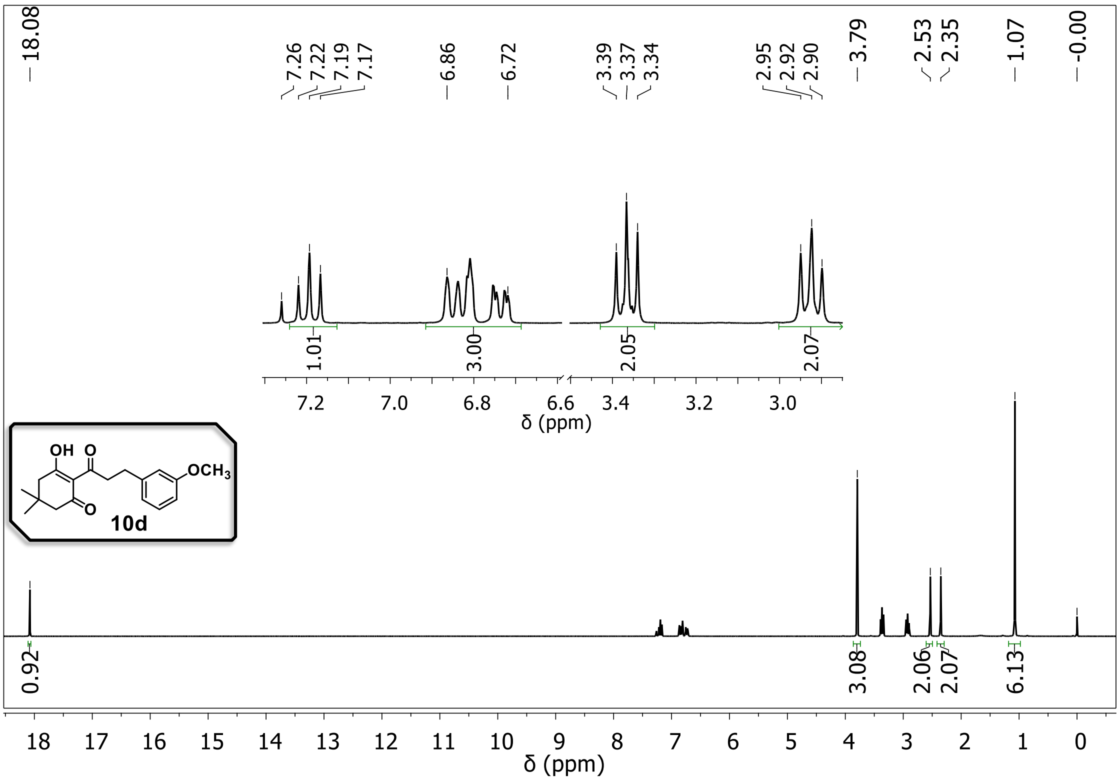

Figure S94. ^{1}H NMR (300 MHz, $CDCl_3$) spectrum of **10d**.

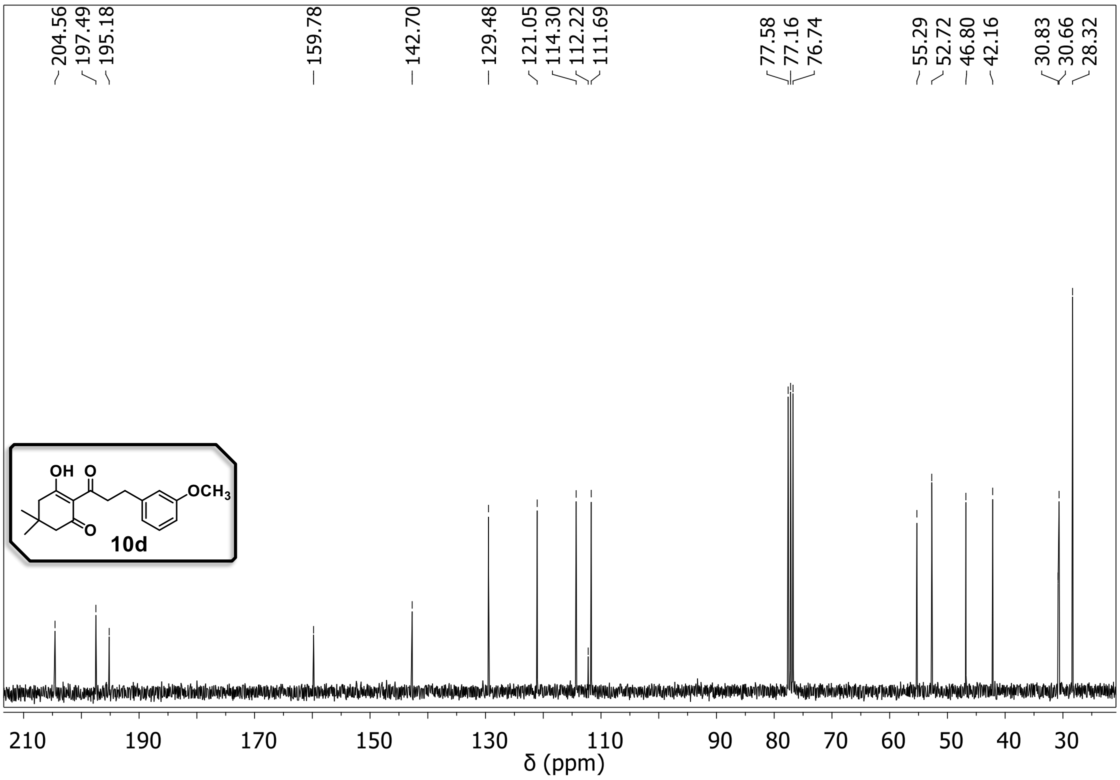

Figure S95. ^{13}C NMR (75 MHZ, $CDCl_3$) spectrum of **10d**.

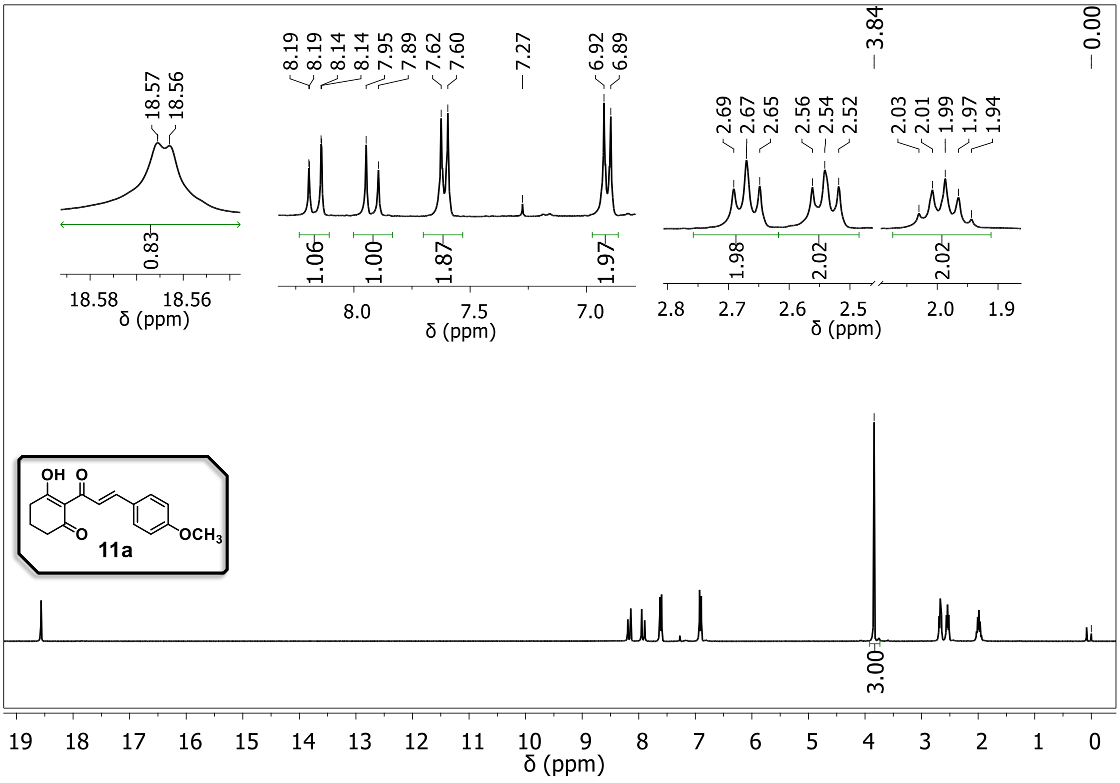

Figure S96. ^{1}H NMR (300 MHz, $CDCl_3$) spectrum of **11a**.

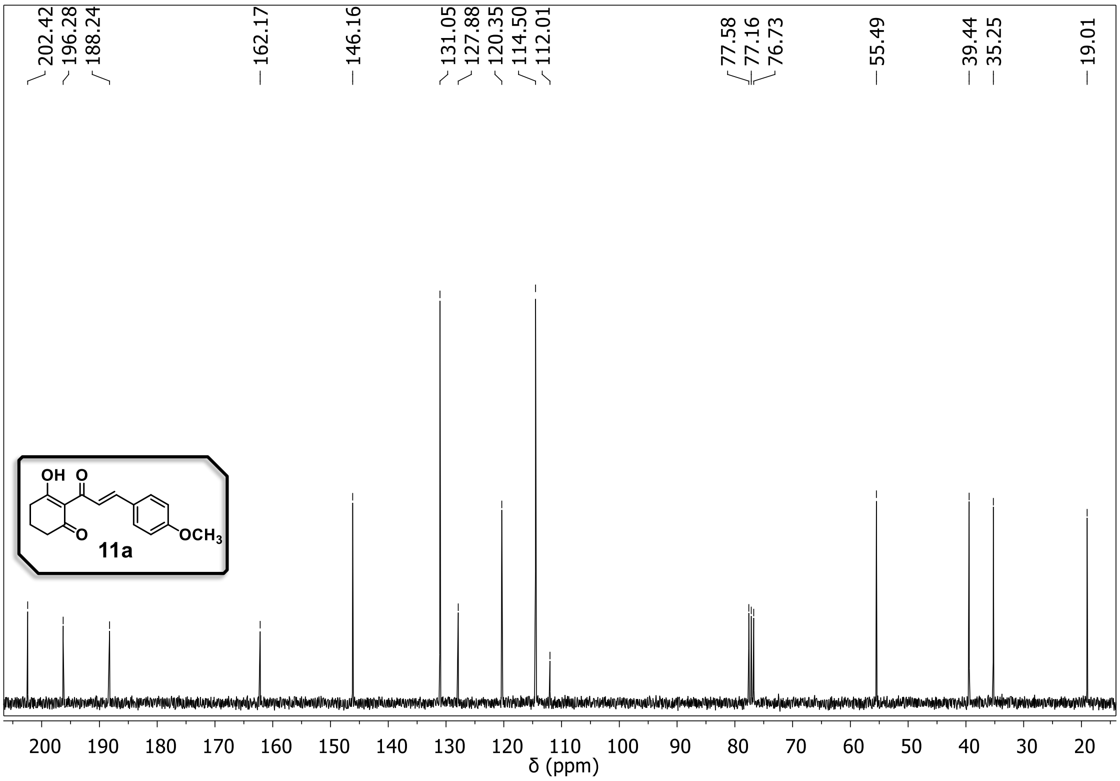

Figure S97. ^{13}C NMR (75 MHZ, $CDCl_3$) spectrum of **11a**.

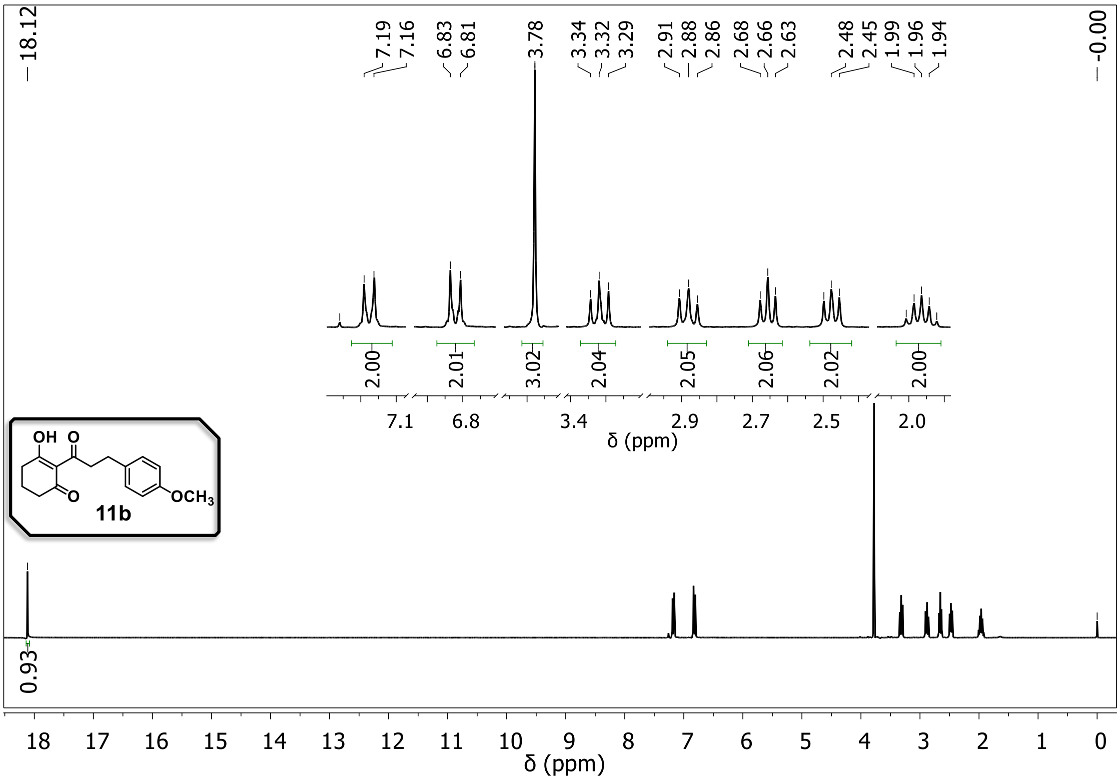

Figure S98. ^{1}H NMR (300 MHz, $CDCl_3$) spectrum of **11b**.

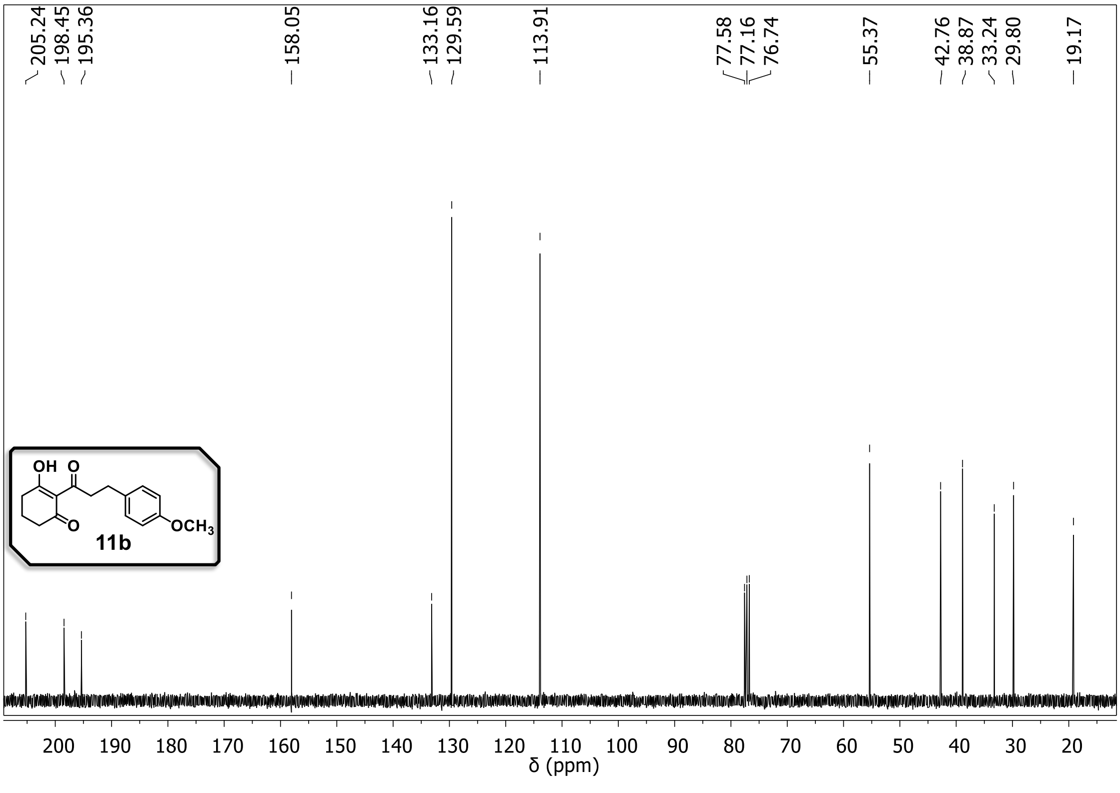

Figure S99. ^{13}C NMR (75 MHZ, $CDCl_3$) spectrum of **11b**.

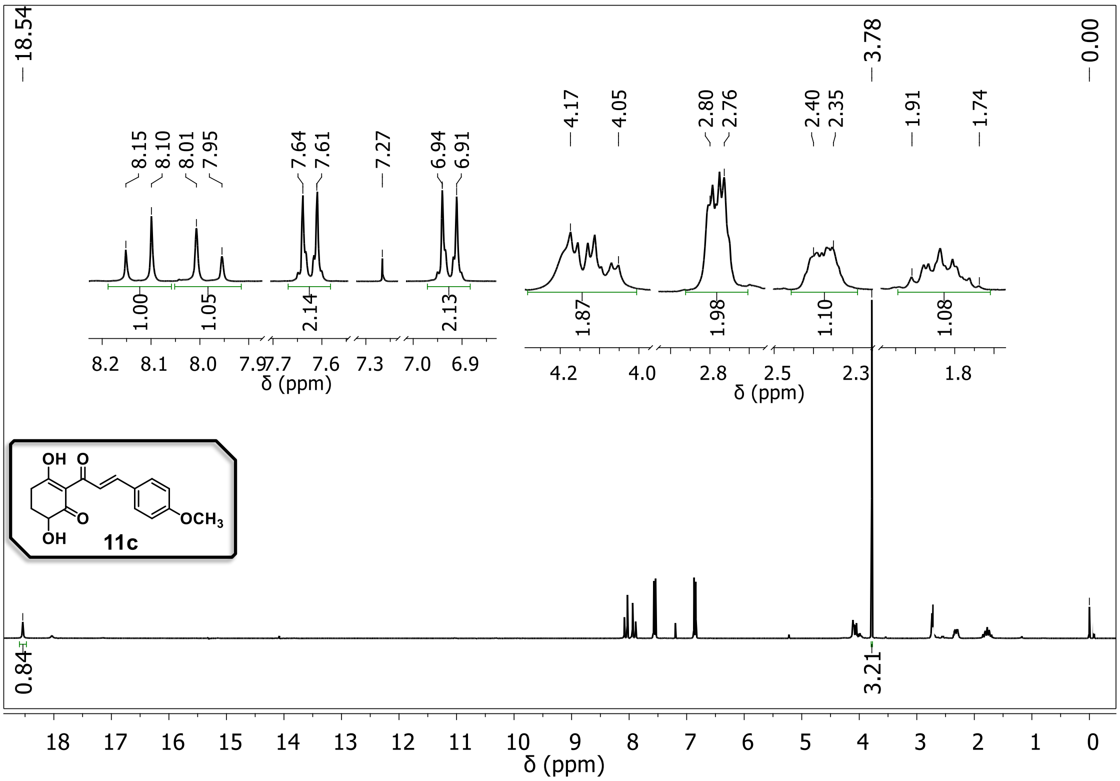

Figure S100. ^{1}H NMR (300 MHz, $CDCl_3$) spectrum of **11c**.

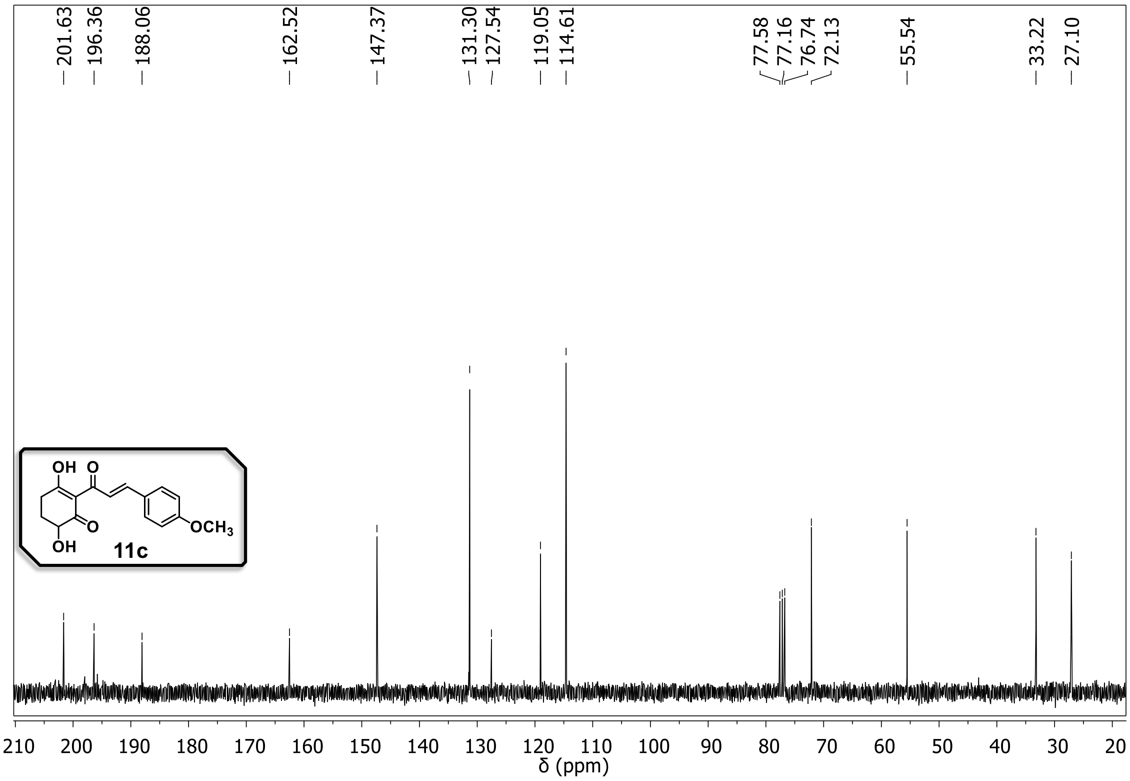

Figure S101. ^{13}C NMR (75 MHZ, $CDCl_3$) spectrum of **11c**.

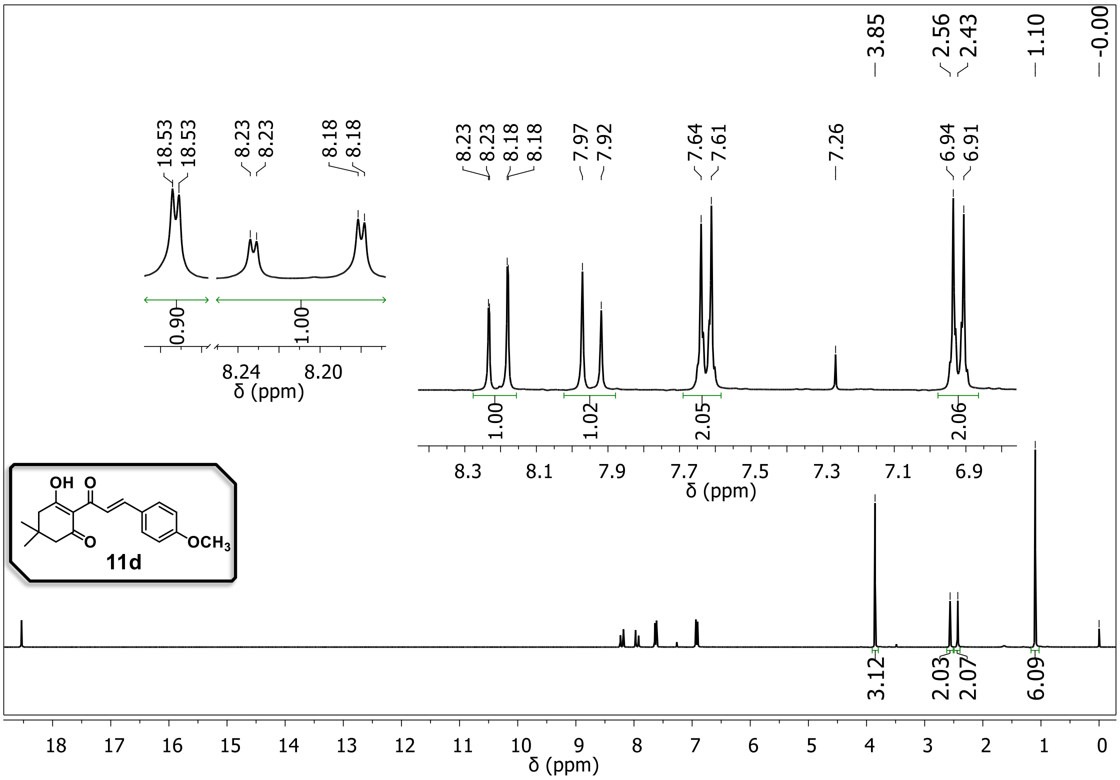

Figure S102. ^{1}H NMR (300 MHz, $CDCl_3$) spectrum of **11d**.

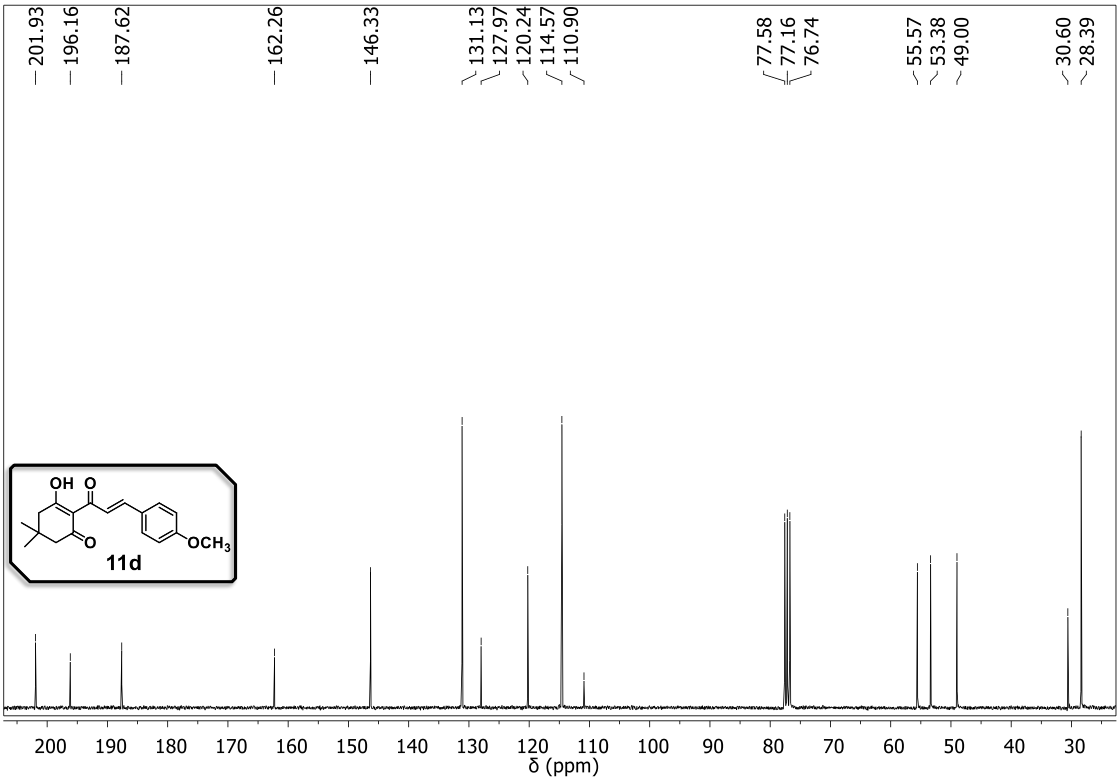

Figure S103. ^{13}C NMR (75 MHZ, $CDCl_3$) spectrum of **11d**.

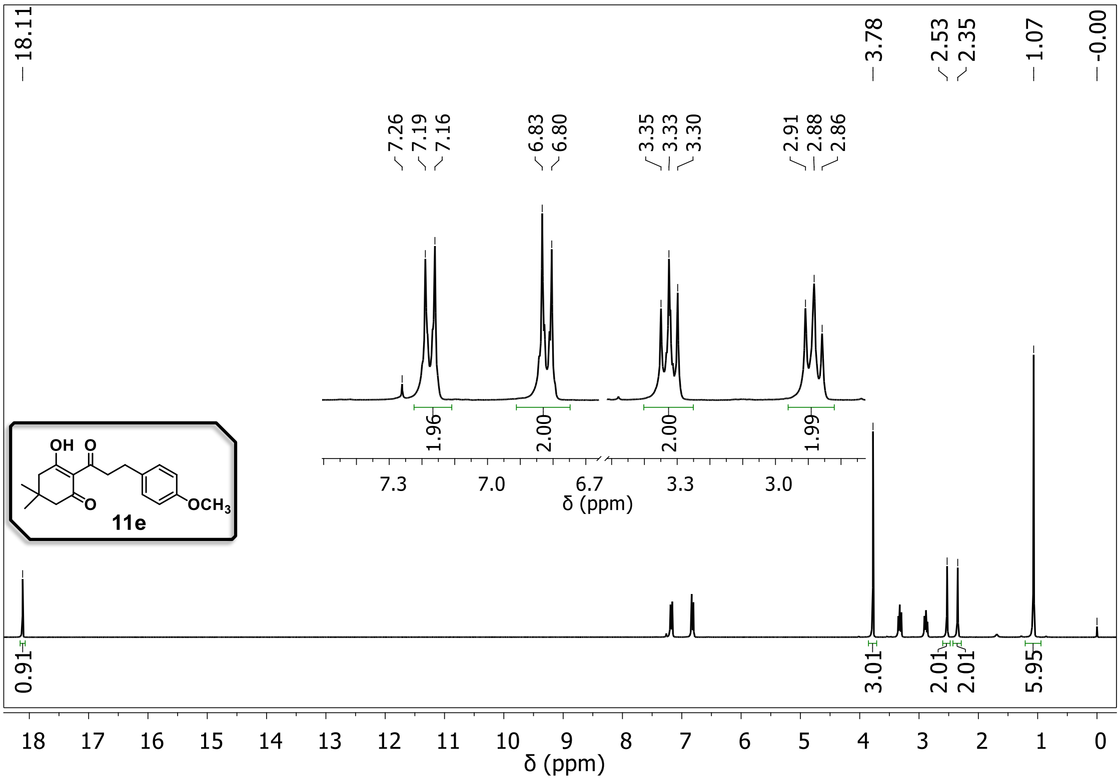

Figure S104. ^{1}H NMR (300 MHz, $CDCl_3$) spectrum of **11e**.

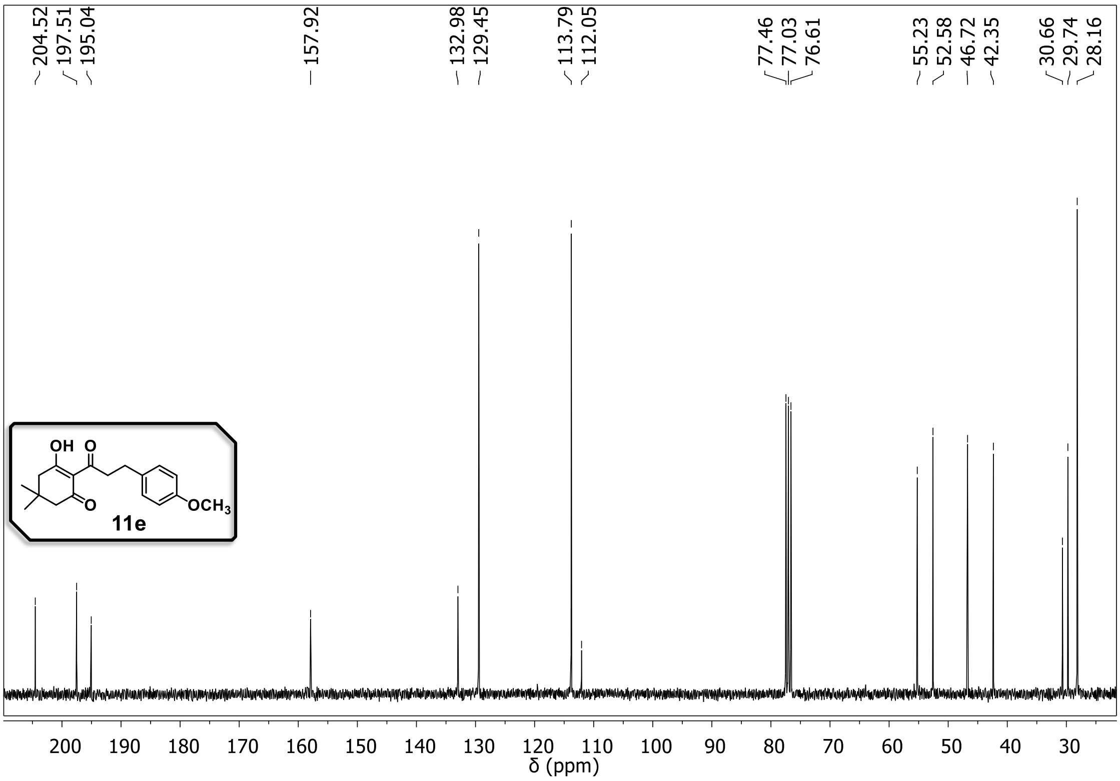

Figure S105. ^{13}C NMR (75 MHZ, $CDCl_3$) spectrum of **11e**.

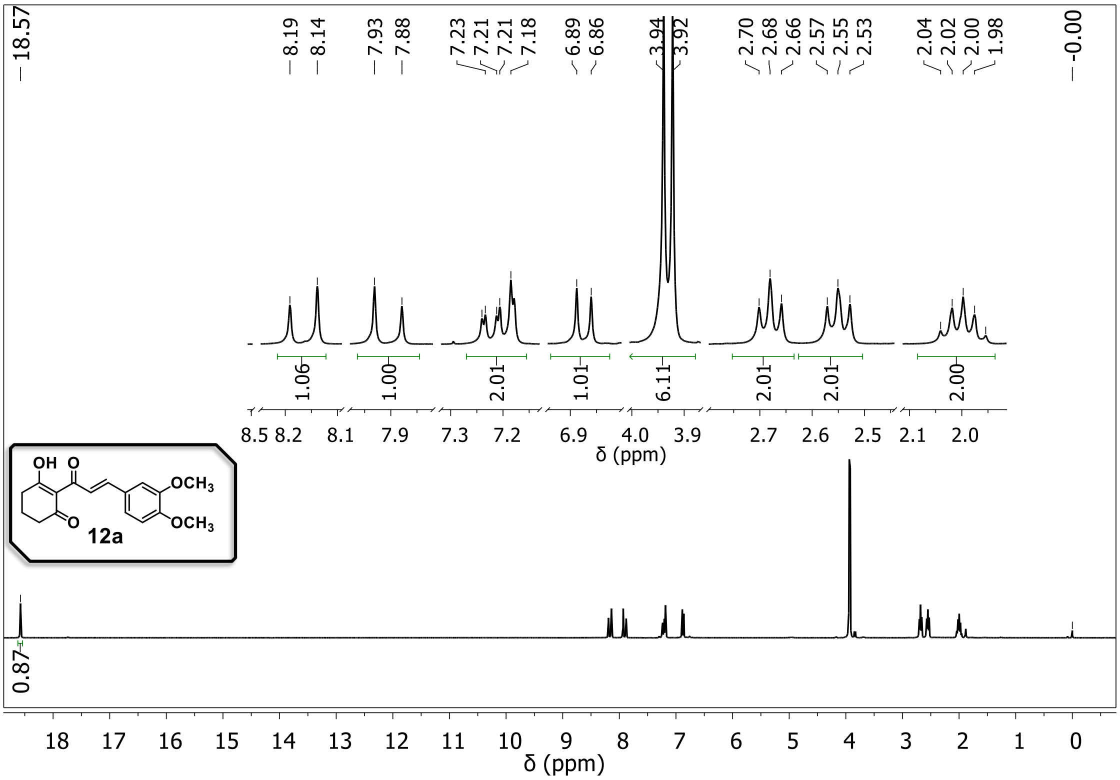

Figure S106. ^{1}H NMR (300 MHz, $CDCl_3$) spectrum of **12a**.

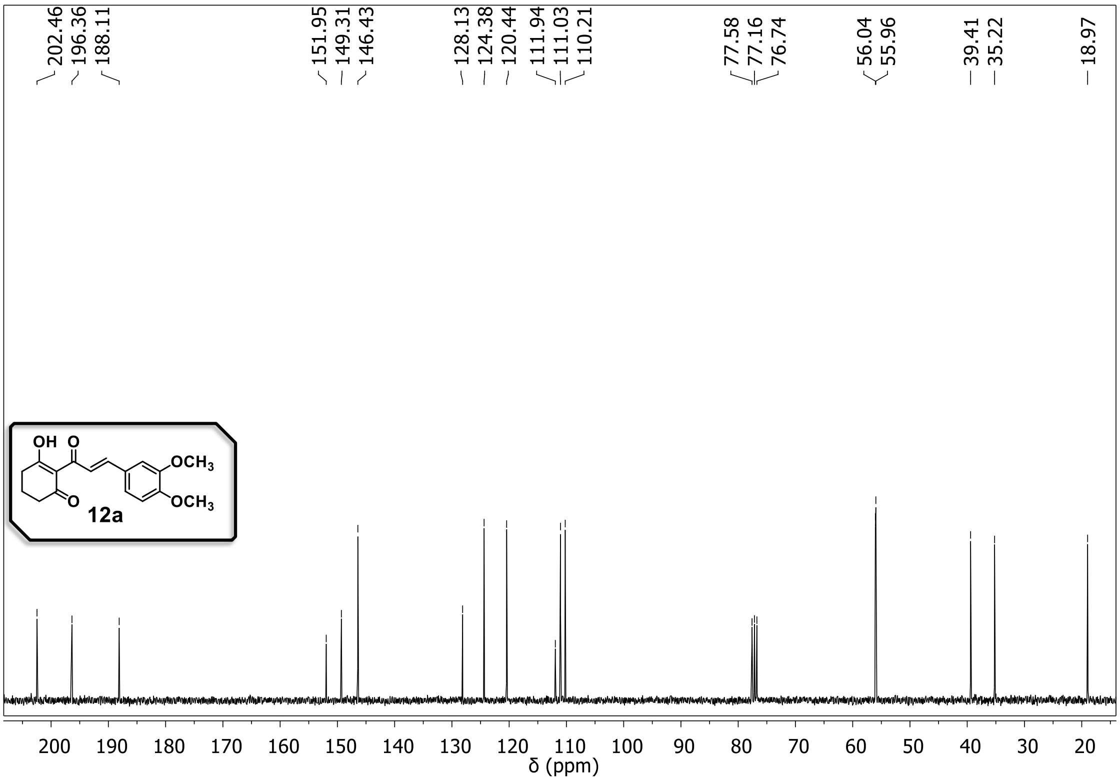

Figure S107. ^{13}C NMR (75 MHZ, $CDCl_3$) spectrum of **12a**.

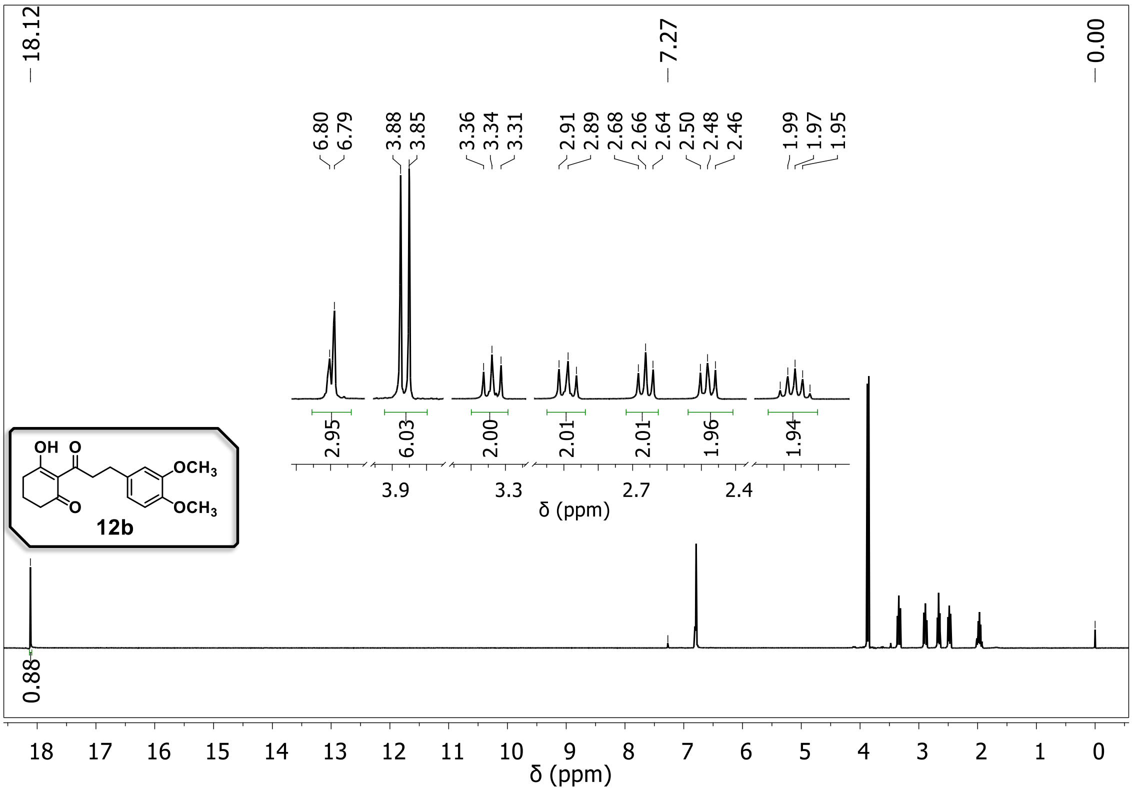

Figure S108. ^{1}H NMR (300 MHz, $CDCl_3$) spectrum of **12b**.

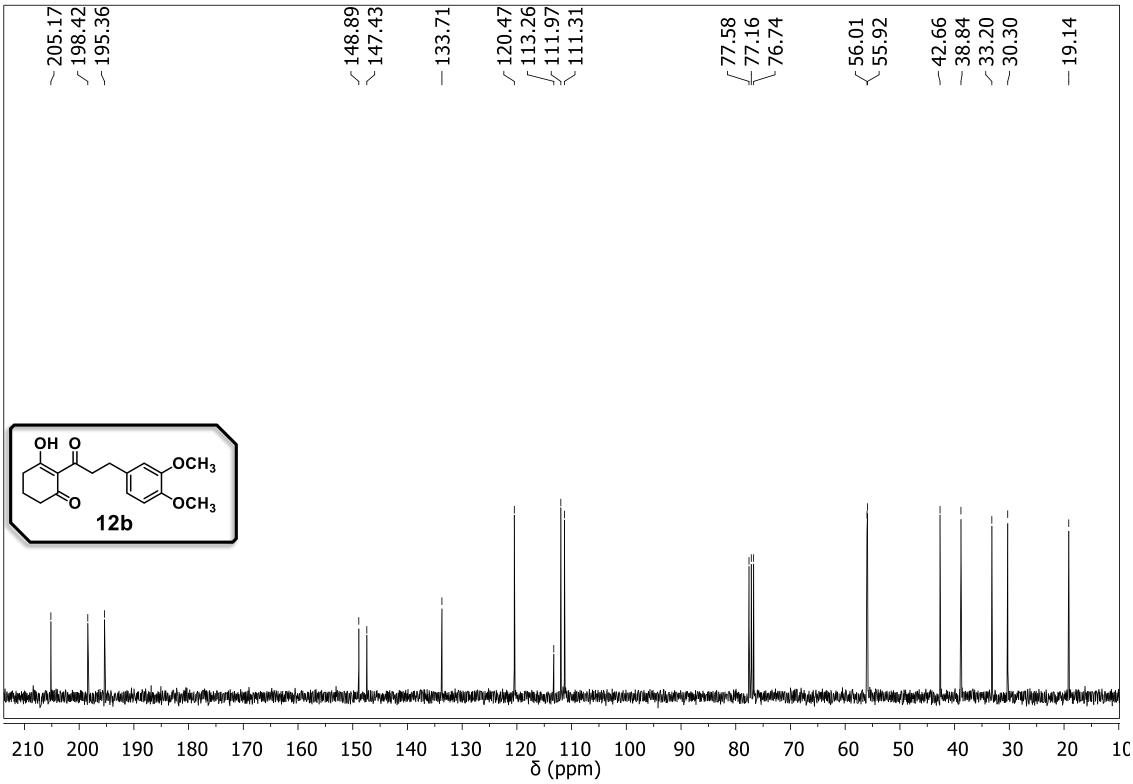

Figure S109. ^{13}C NMR (75 MHZ, $CDCl_3$) spectrum of **12b**.

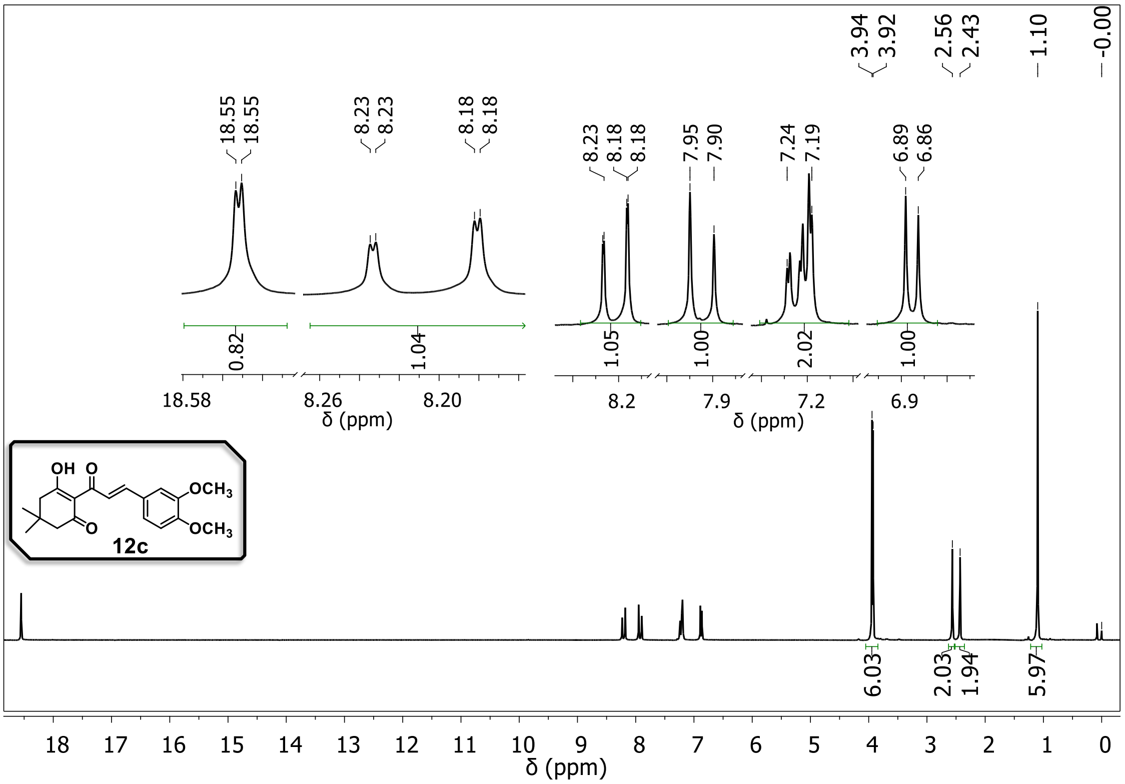

Figure S110. ^{1}H NMR (300 MHz, $CDCl_3$) spectrum of **12c**.

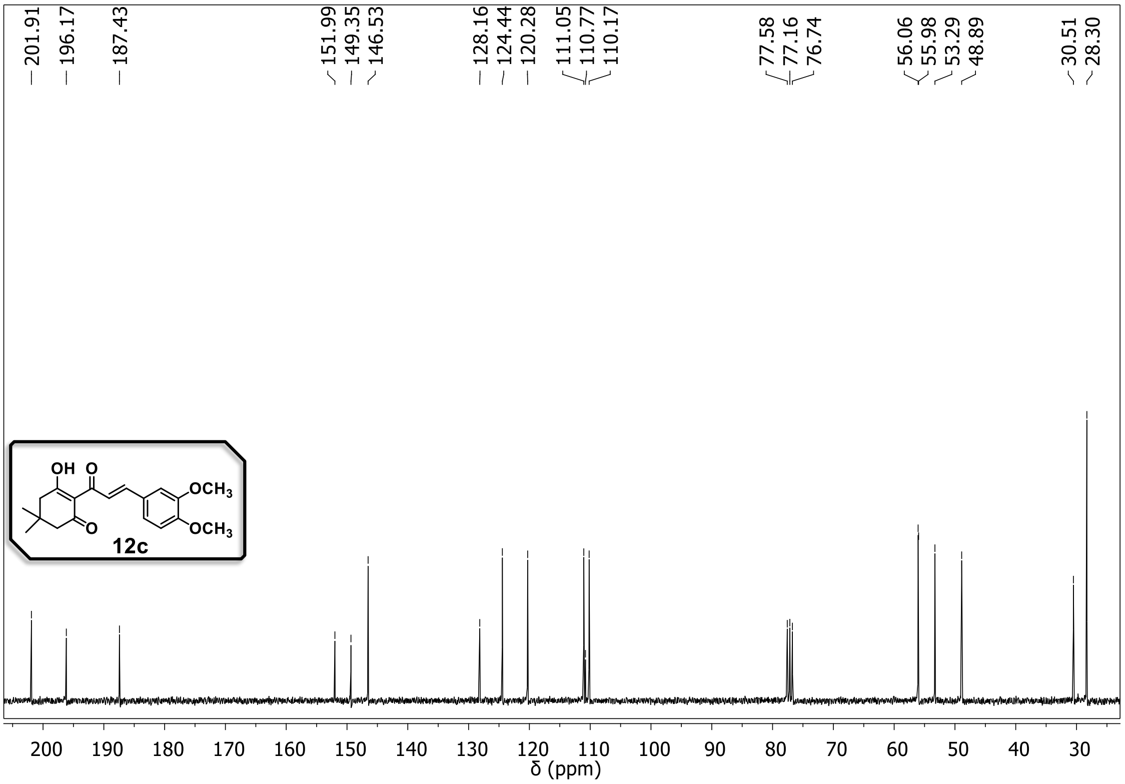

Figure S111. ^{13}C NMR (75 MHZ, $CDCl_3$) spectrum of **12c**.

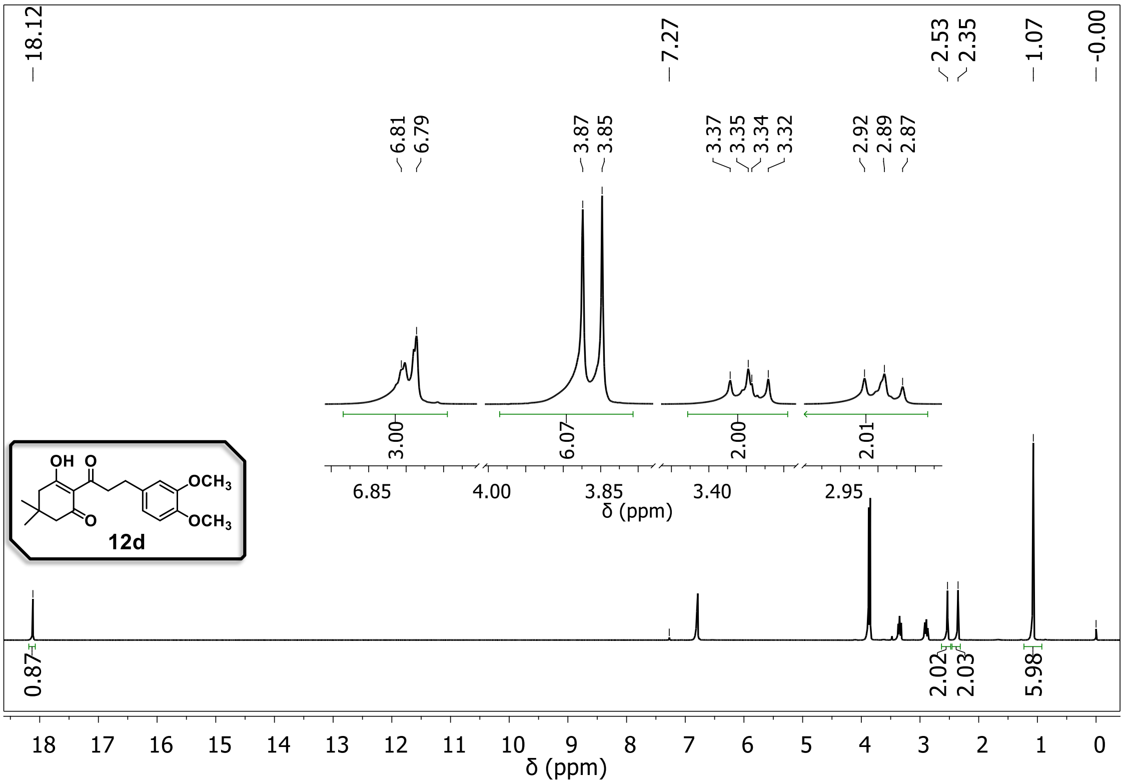

Figure S112. ^{1}H NMR (300 MHz, $CDCl_3$) spectrum of **12d**.

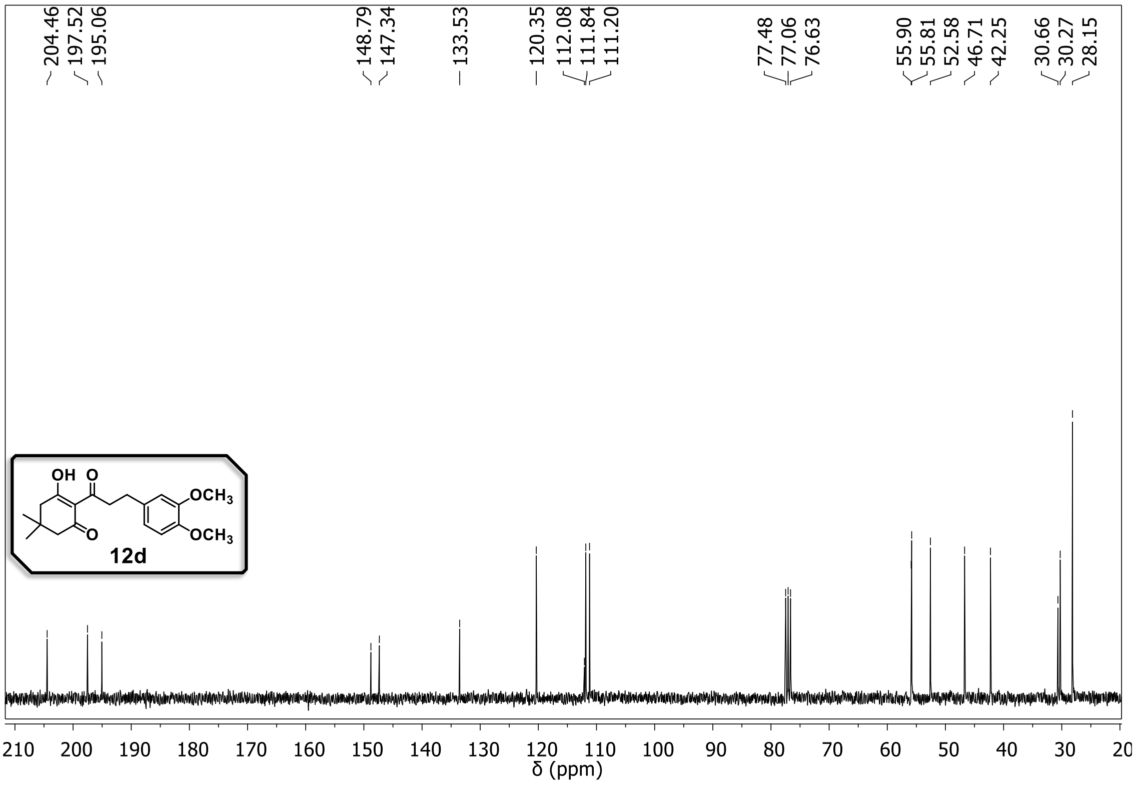

Figure S113. ^{13}C NMR (75 MHZ, $CDCl_3$) spectrum of **12d**.

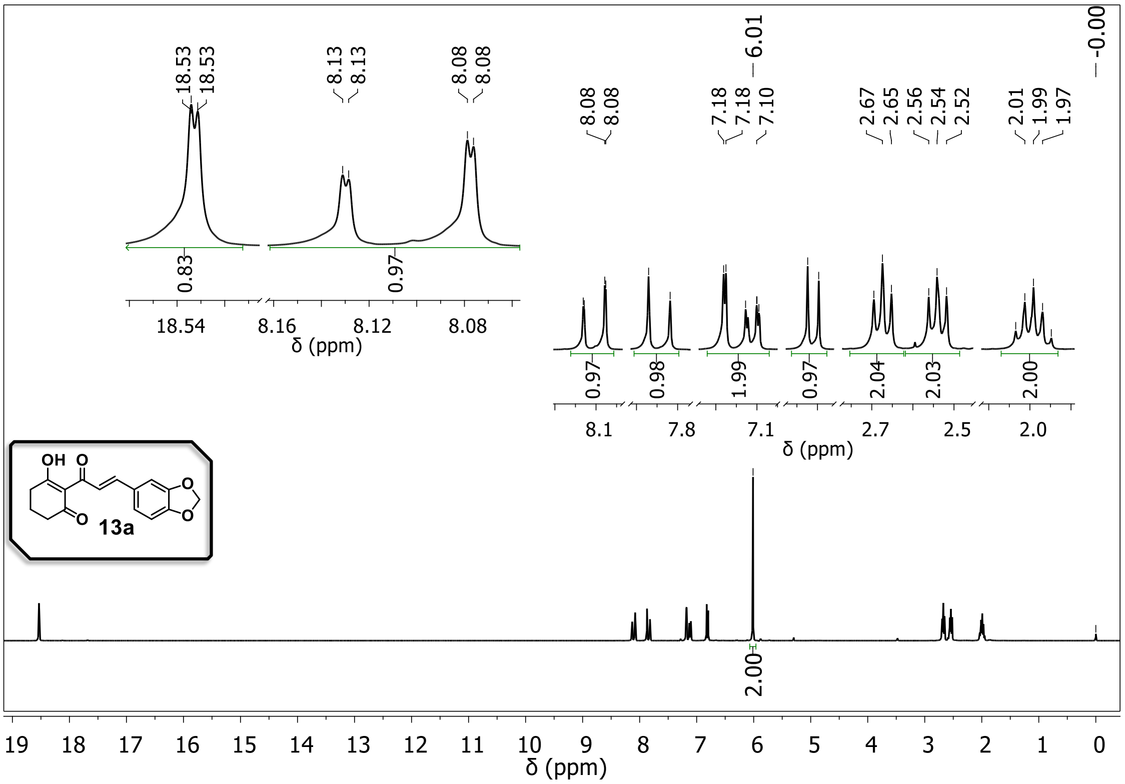

Figure S114. ^{1}H NMR (300 MHz, $CDCl_3$) spectrum of **13a**.

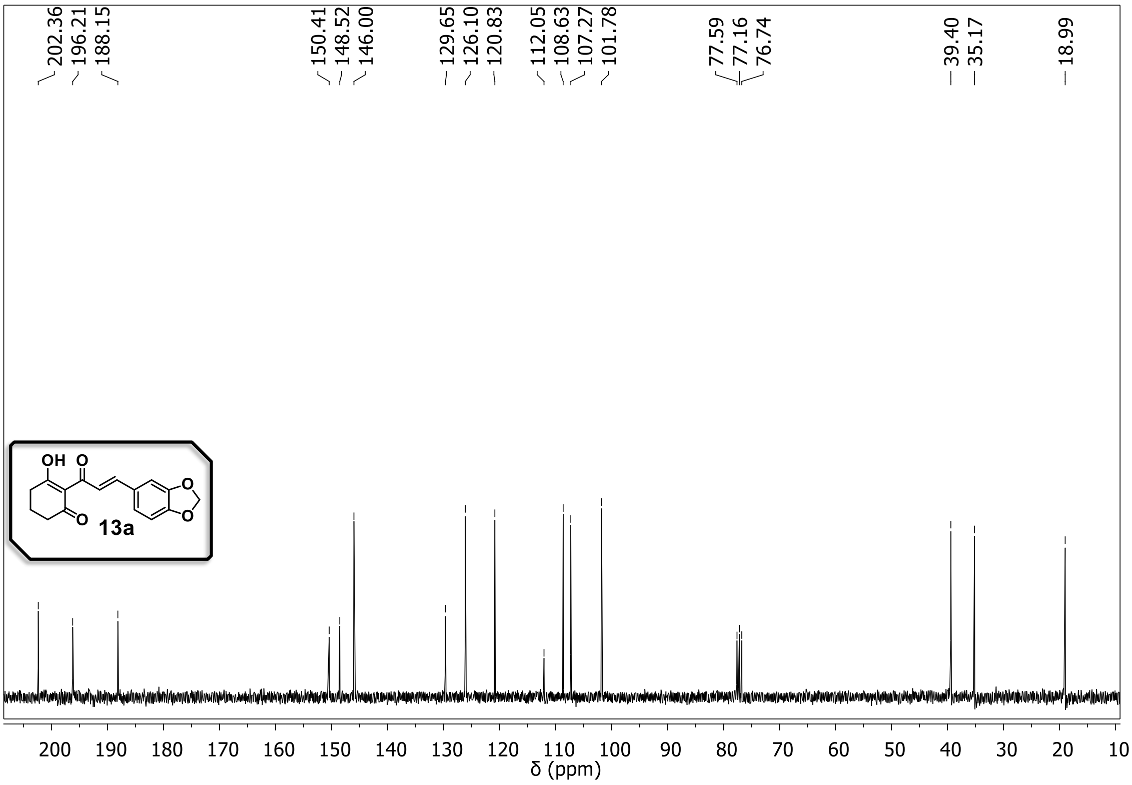

Figure S115. ^{13}C NMR (75 MHZ, $CDCl_3$) spectrum of **13a**.

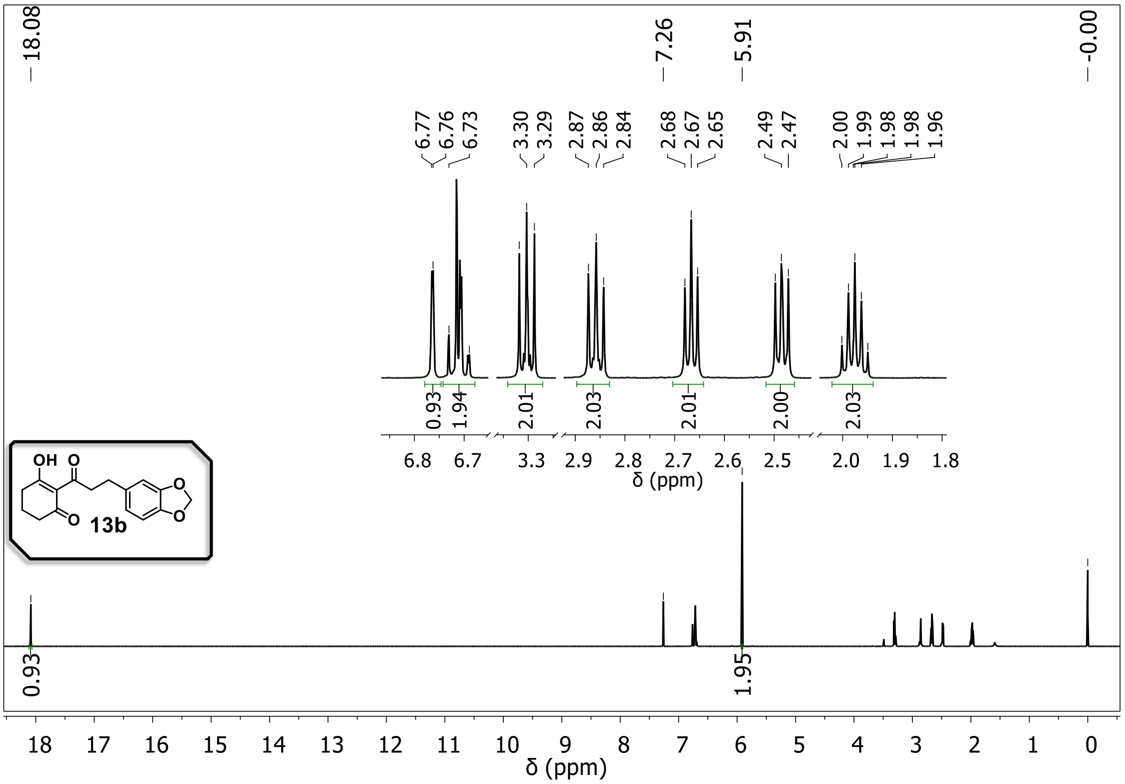

Figure S116. ^{1}H NMR (300 MHz, $CDCl_3$) spectrum of **13b**.

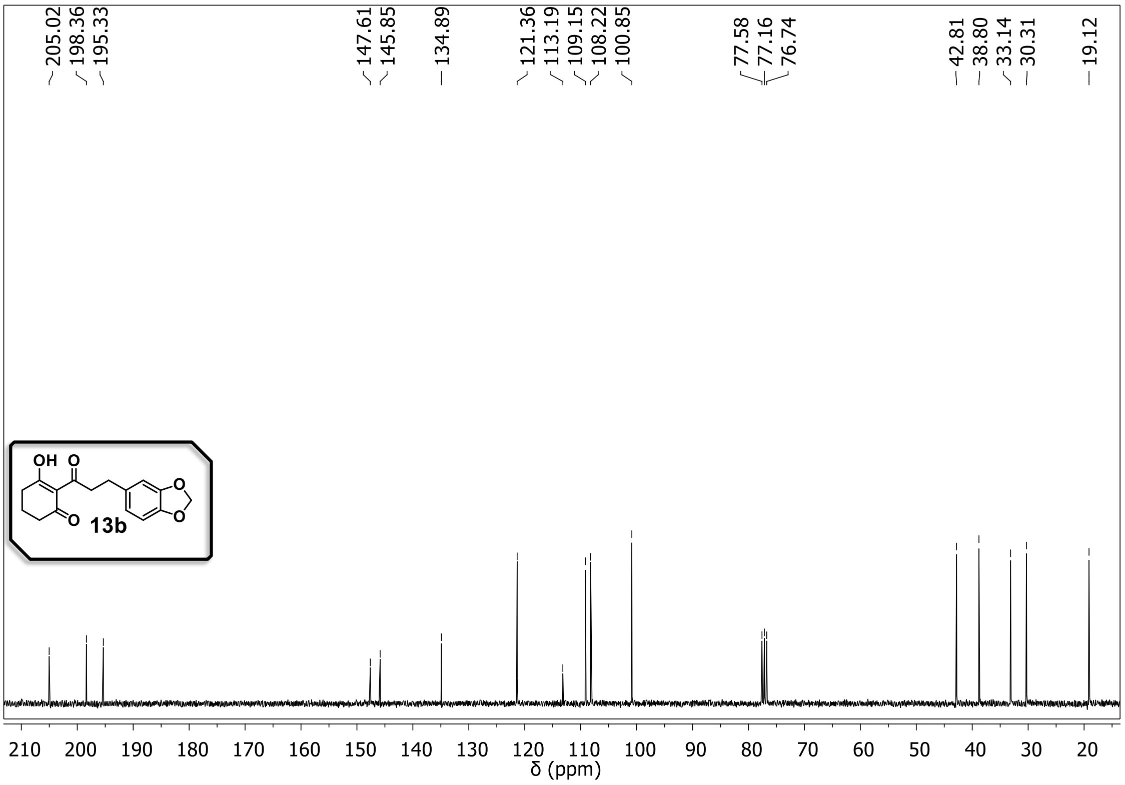

Figure S117. ^{13}C NMR (75 MHZ, $CDCl_3$) spectrum of **13b**.

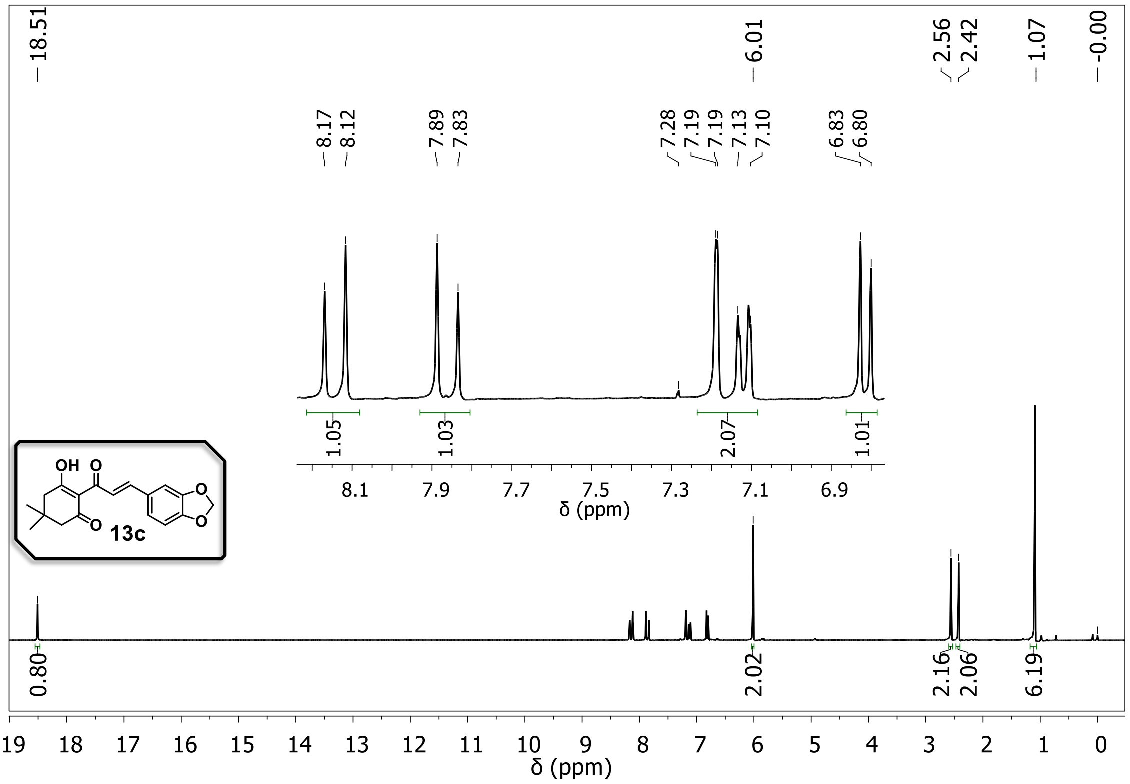

Figure S118. ^{1}H NMR (300 MHz, $CDCl_3$) spectrum of **13c**.

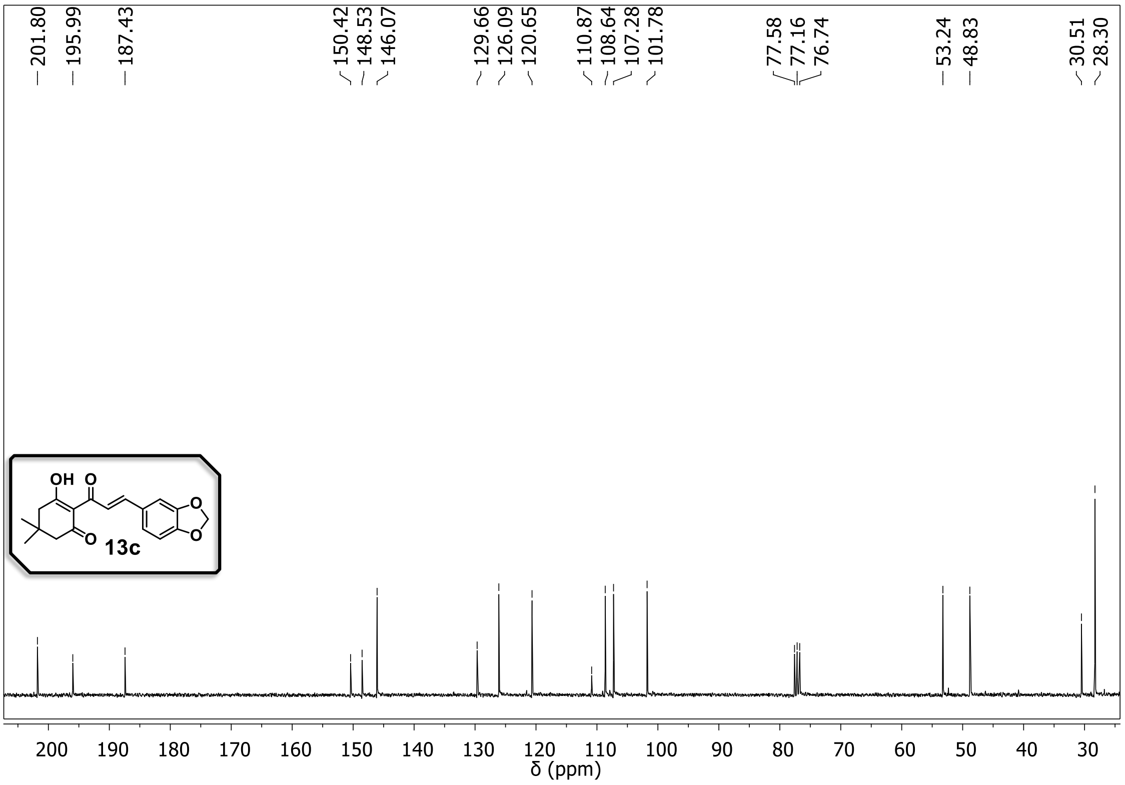

Figure S119. ^{13}C NMR (75 MHZ, $CDCl_3$) spectrum of **13c**.

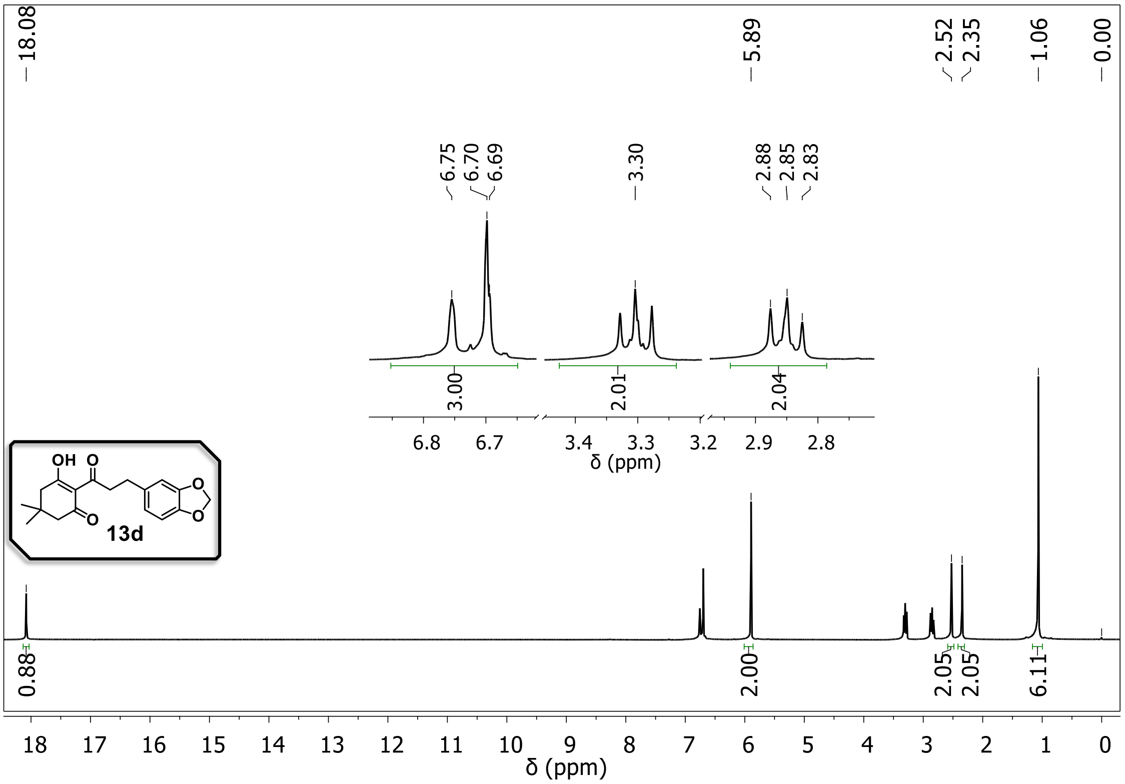

Figure S120. ^{1}H NMR (300 MHz, $CDCl_3$) spectrum of **13d**.

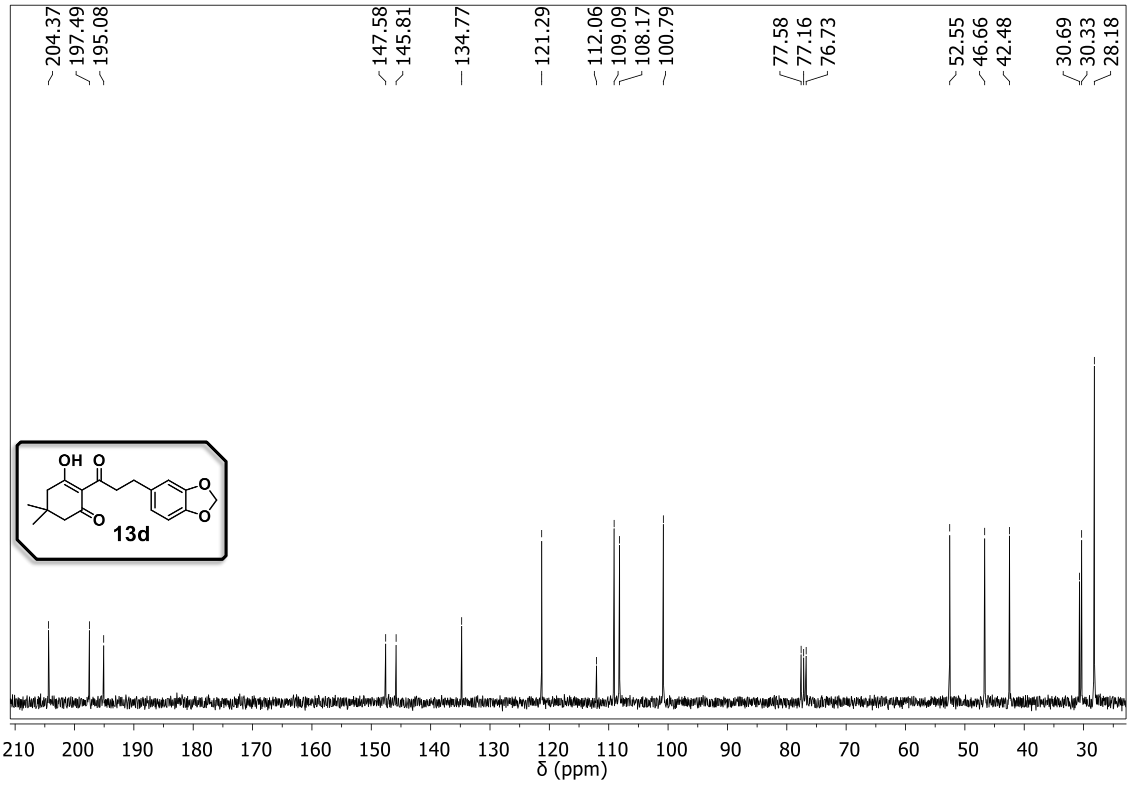

Figure S121. ^{13}C NMR (75 MHZ, $CDCl_3$) spectrum of **13d**.

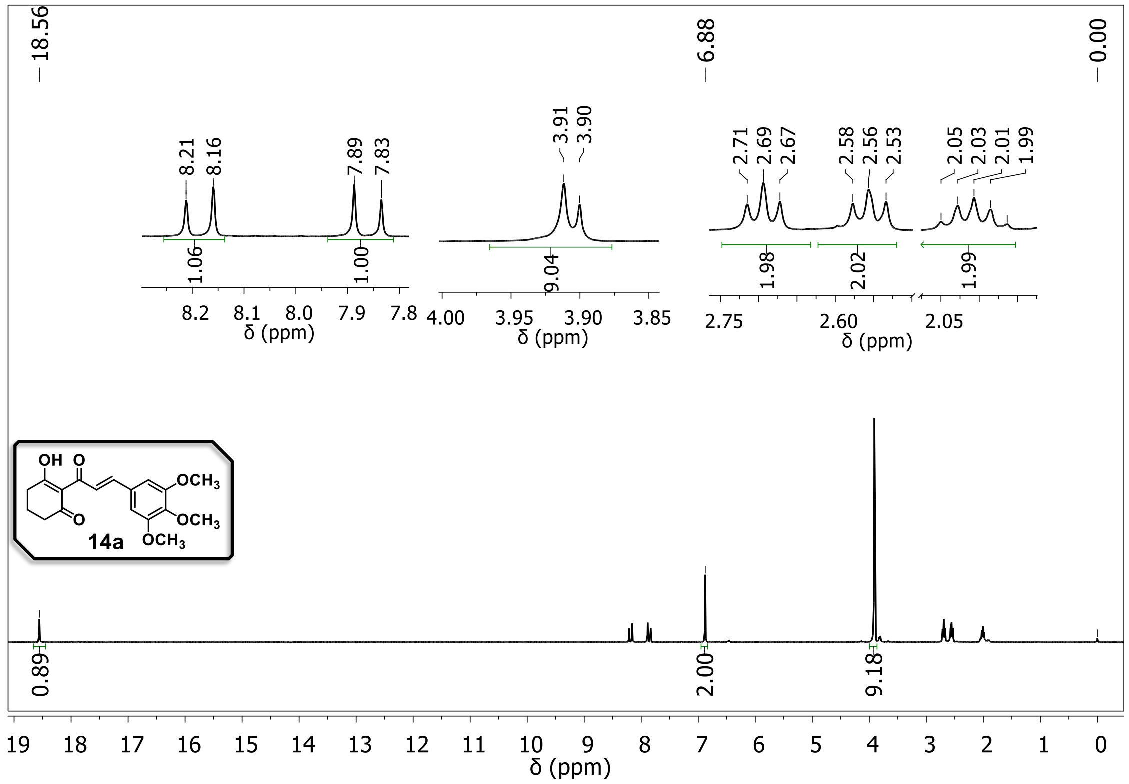

Figure S122. ^{1}H NMR (300 MHz, $CDCl_3$) spectrum of **14a**.

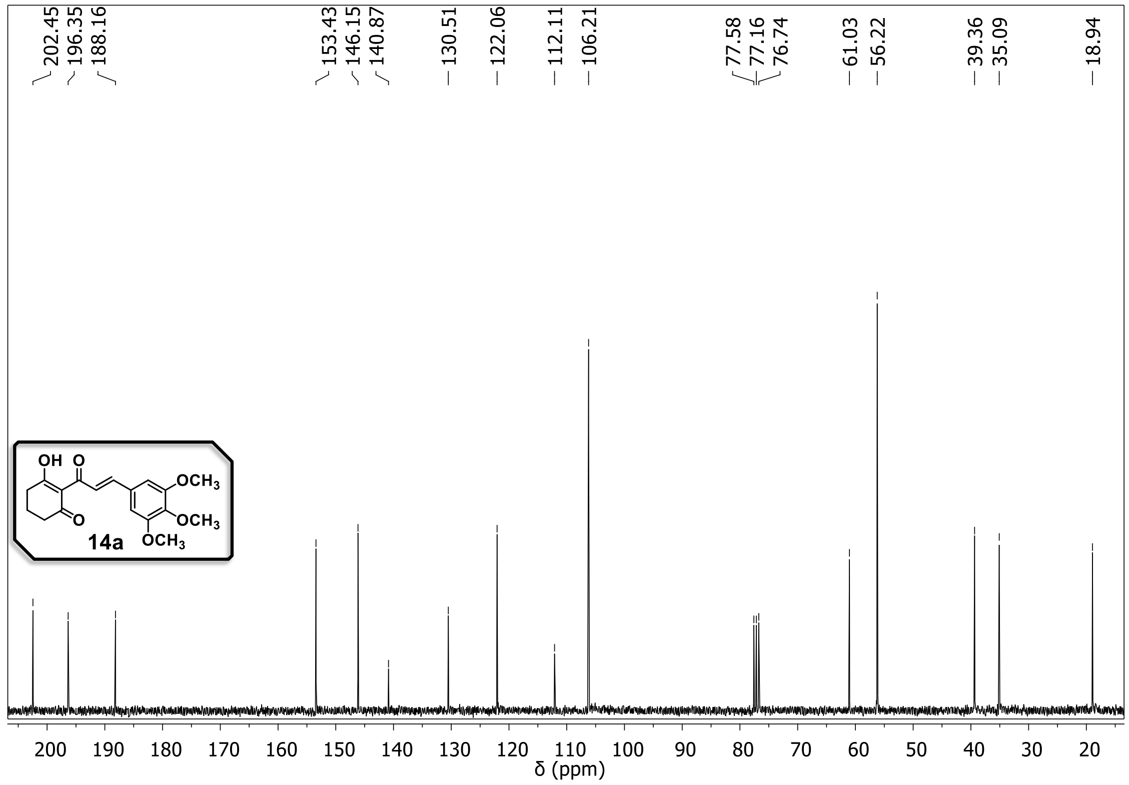

Figure S123. ^{13}C NMR (75 MHZ, $CDCl_3$) spectrum of **14a**.

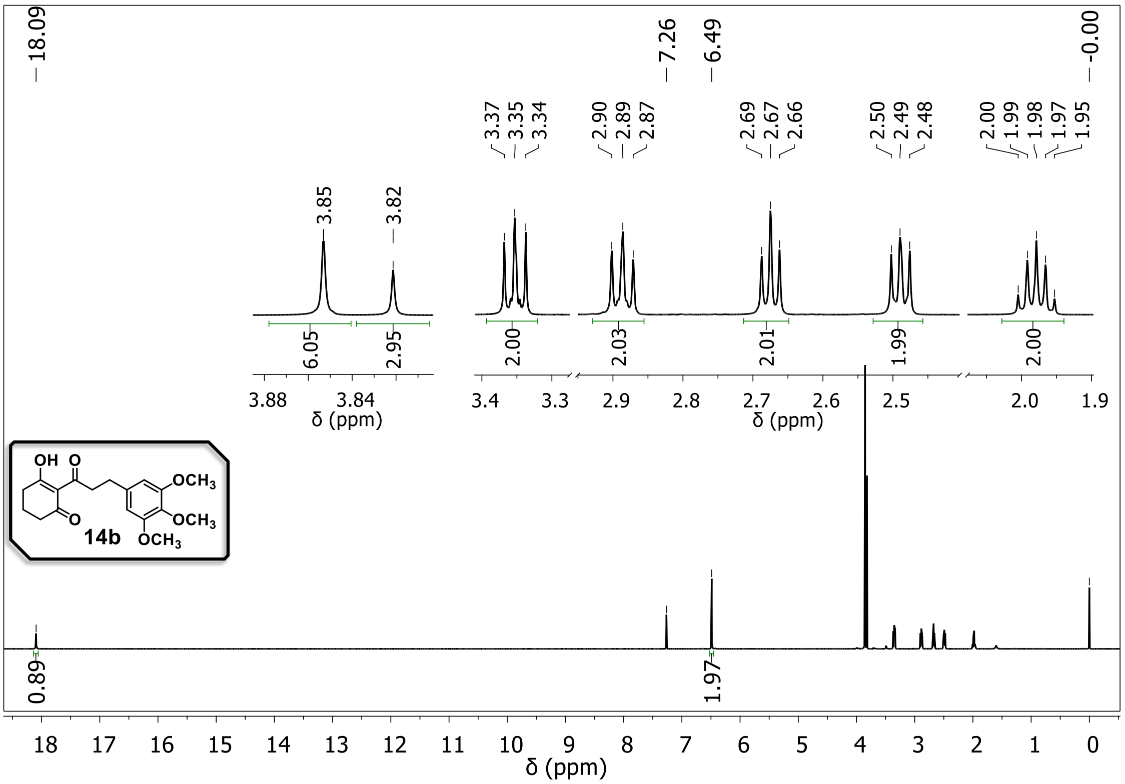

Figure S124. ^{1}H NMR(300 MHz, $CDCl_3$) spectrum of **14b**.

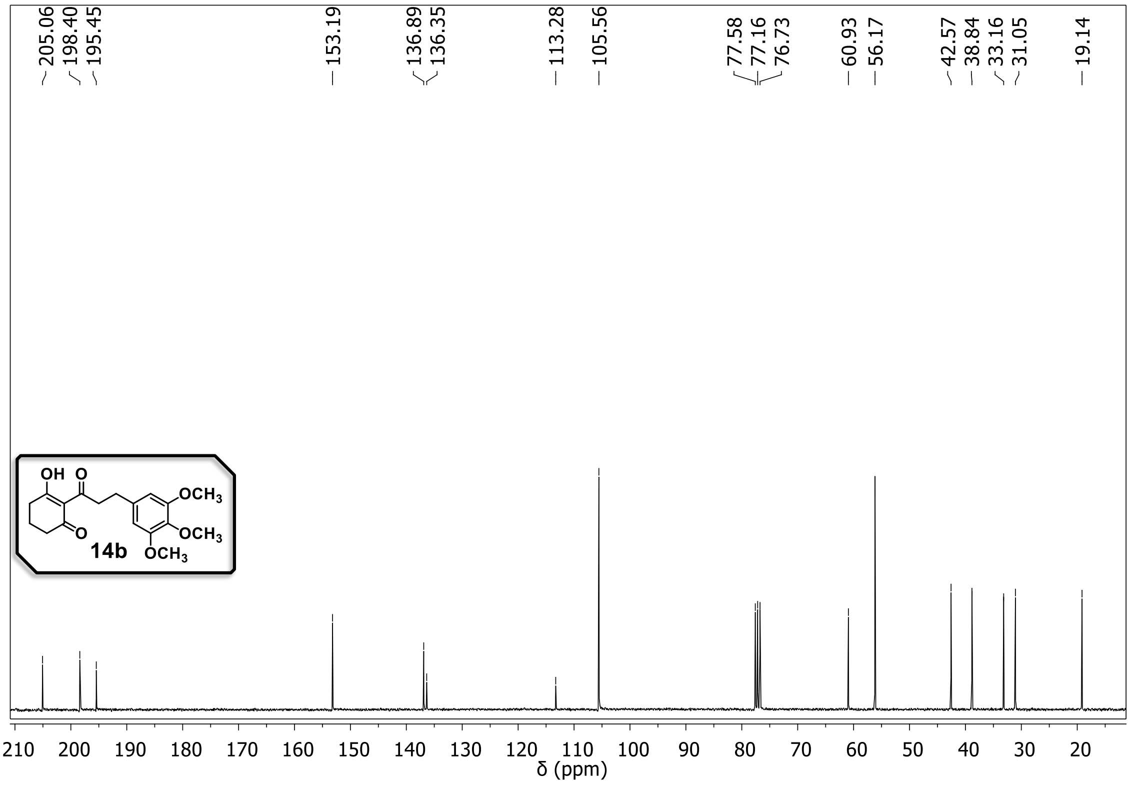

Figure S125. ^{13}C NMR (75 MHZ, $CDCl_3$) spectrum of **14b**.

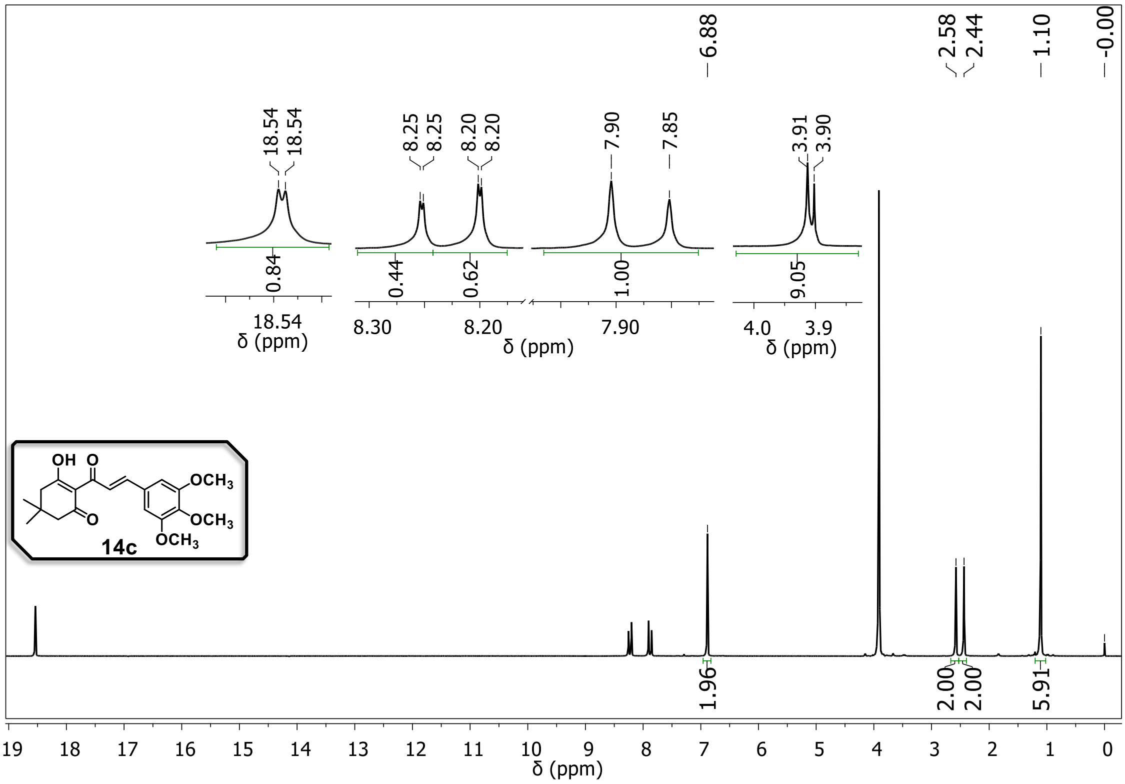

Figure S126. ^{1}H NMR (300 MHz, $CDCl_3$) spectrum of **14c**.

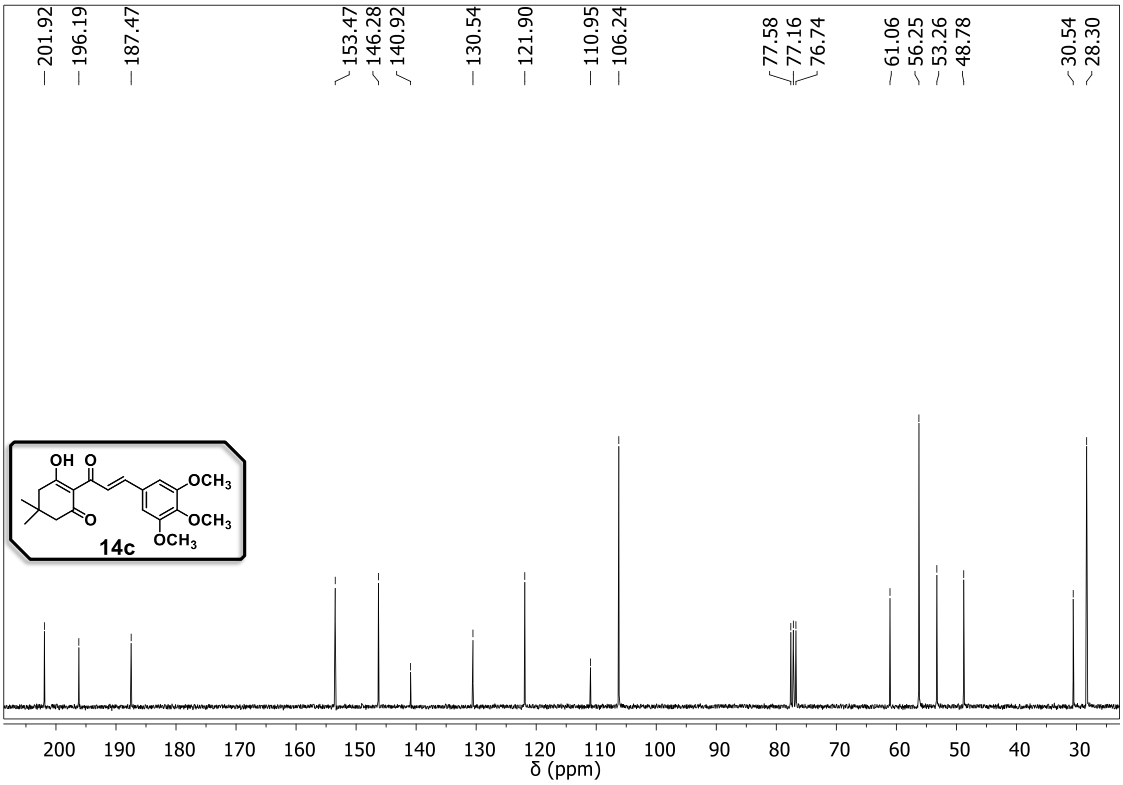

Figure S127. ^{13}C NMR (75 MHZ, $CDCl_3$) spectrum of **14c**.

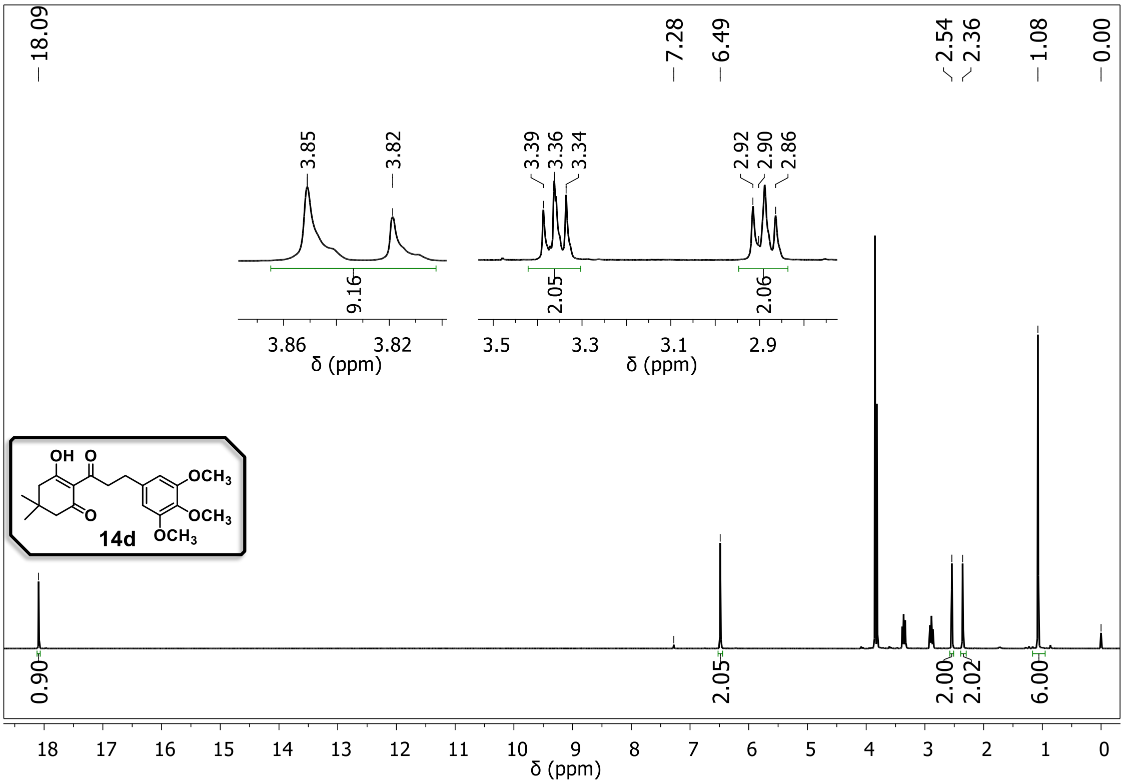

Figure S128. ^{1}H NMR (500 MHz, $CDCl_3$) spectrum of **14d**.

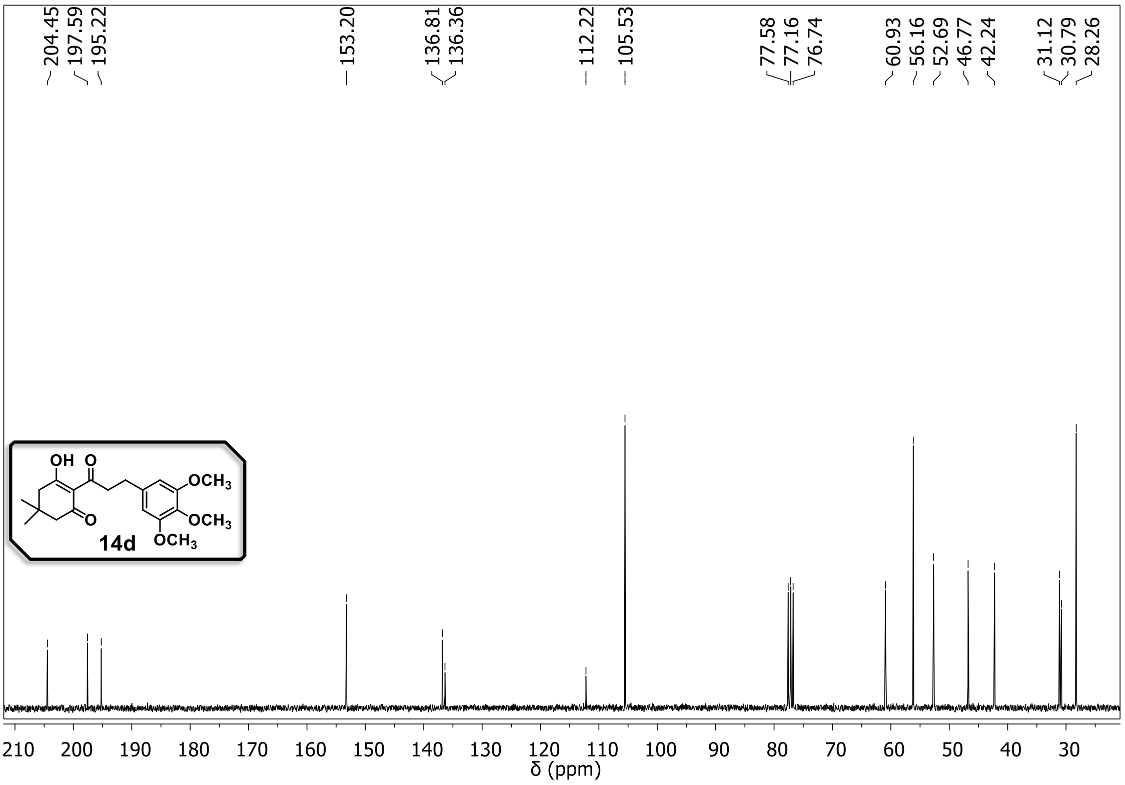

Figure S129. ^{13}C NMR (75 MHZ, $CDCl_3$) spectrum of **14d**.

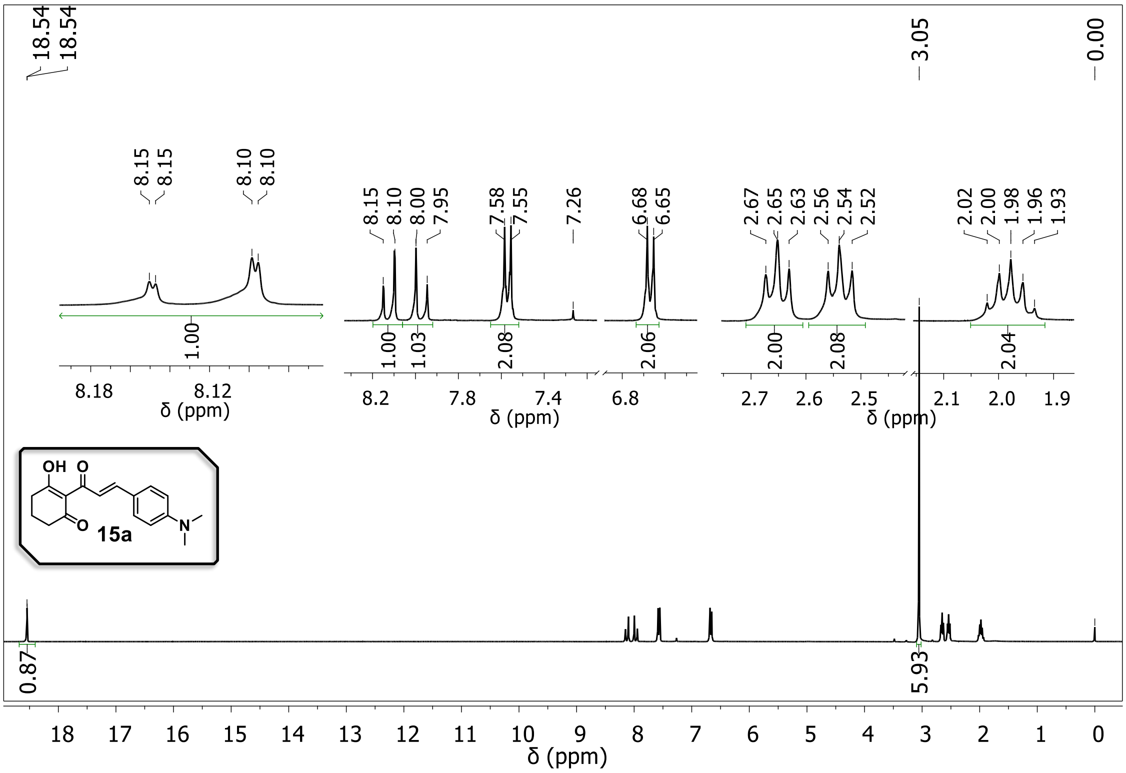

Figure S130. ^{1}H NMR (300 MHz, $CDCl_3$) spectrum of **15a**.

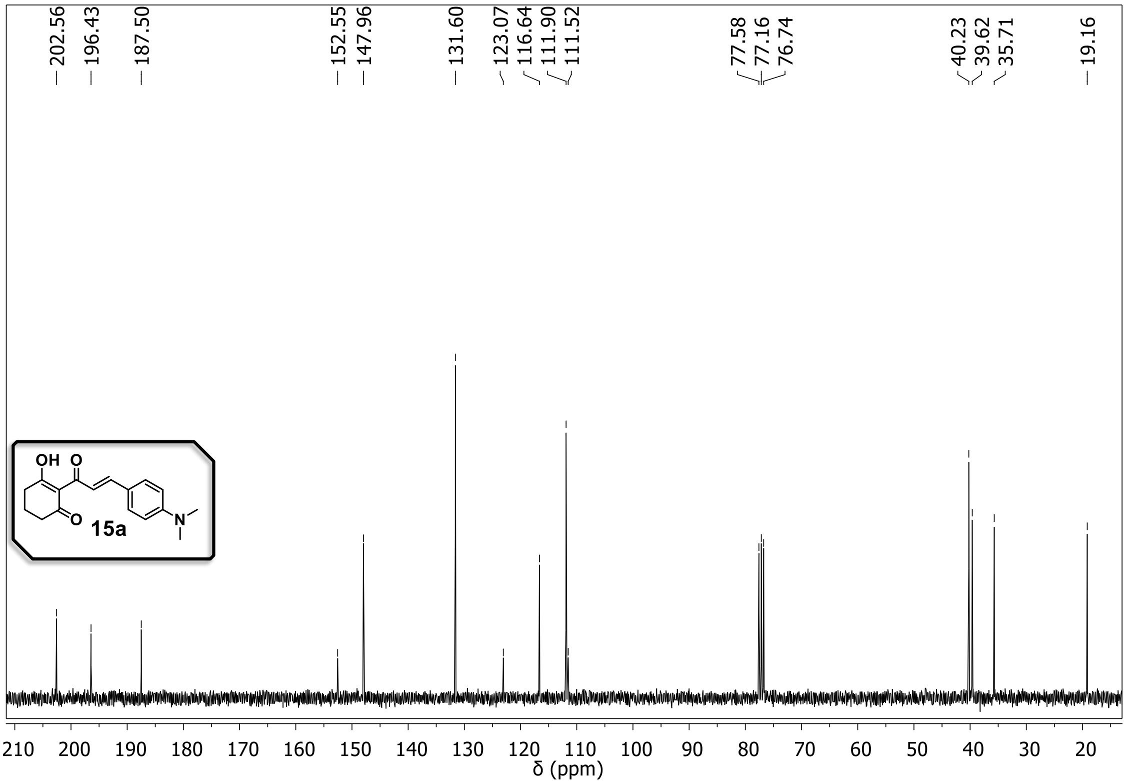

Figure S131. ^{13}C NMR (75 MHZ, $CDCl_3$) spectrum of **15a**.

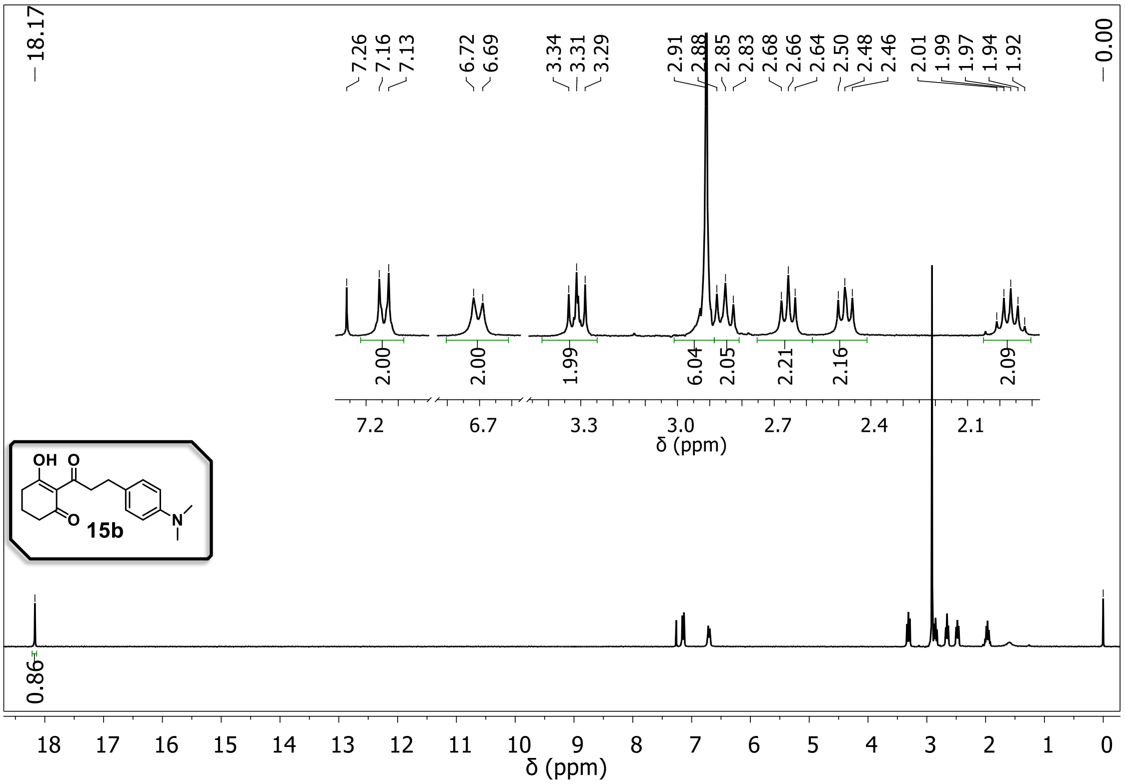

Figure S132. ^{1}H NMR (300 MHz, $CDCl_3$) spectrum of **15b**.

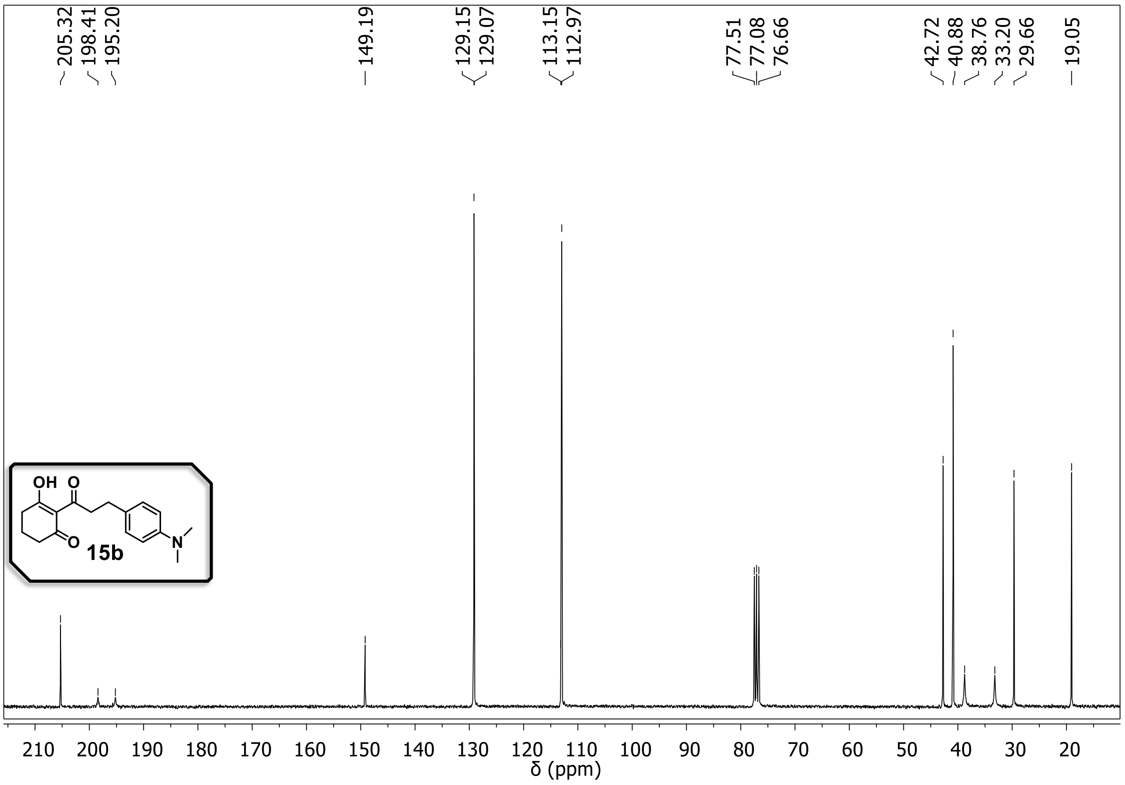

Figure S133. ^{13}C NMR (75 MHZ, $CDCl_3$) spectrum of **15b**.

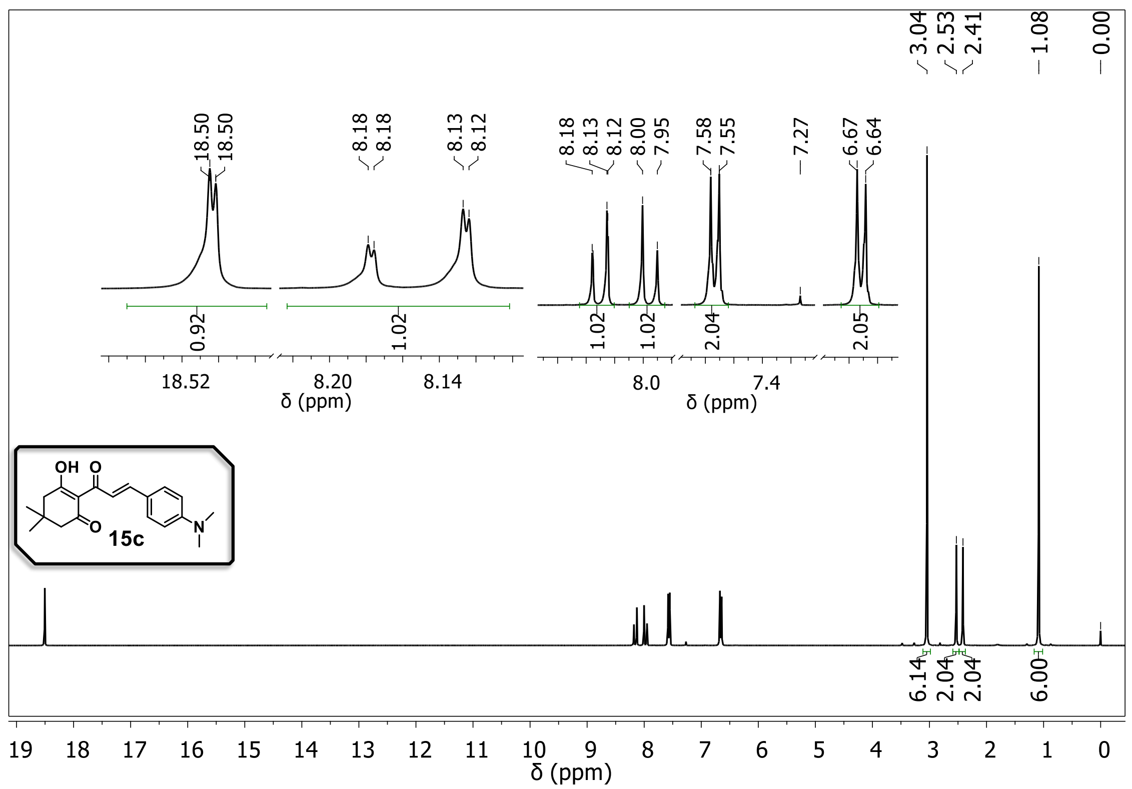

Figure S134. ^{1}H NMR (300 MHz, $CDCl_3$) spectrum of **15c**.

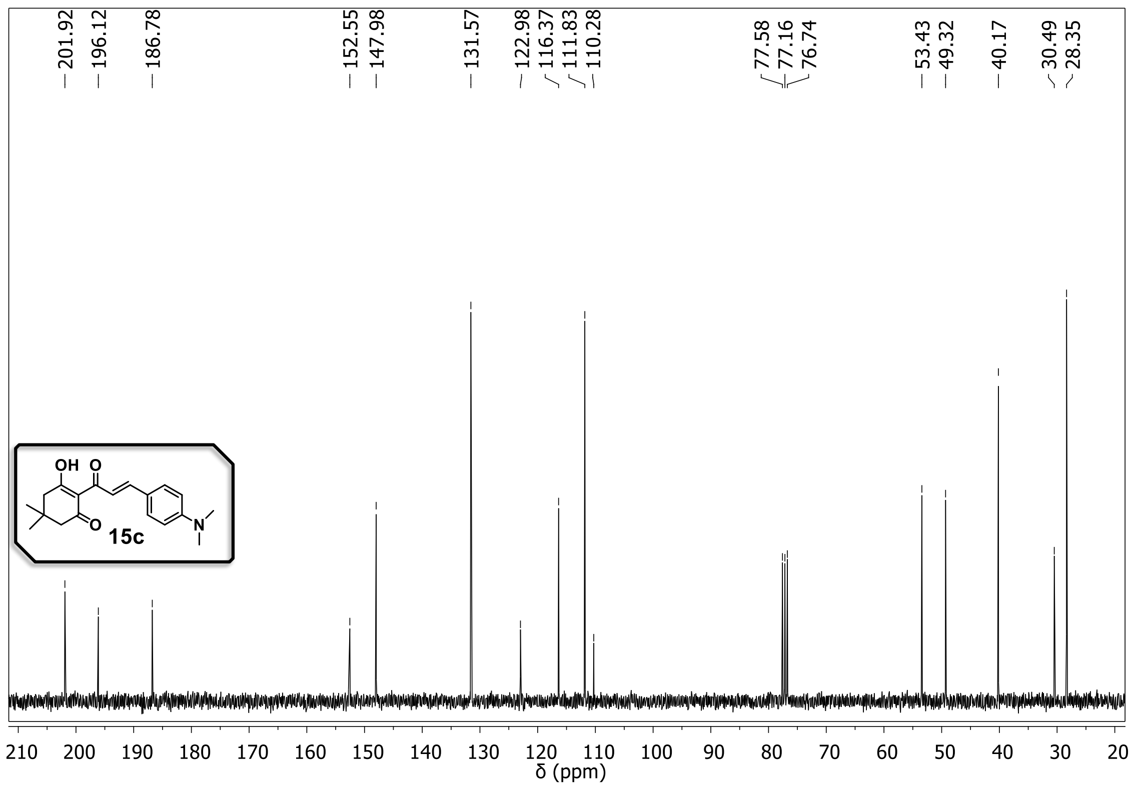

Figure S135. ^{13}C NMR (75 MHZ, $CDCl_3$) spectrum of **15c**.

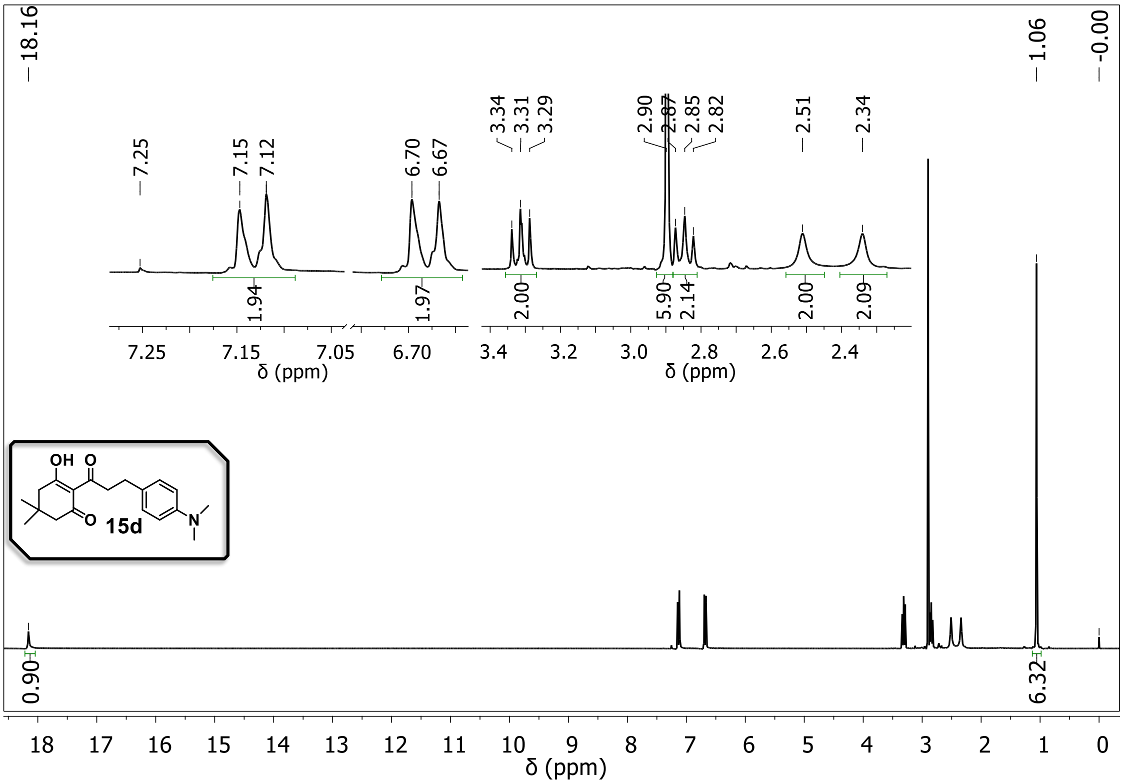

Figure S136. ^{1}H NMR (300 MHz, $CDCl_3$) spectrum of **15d**.

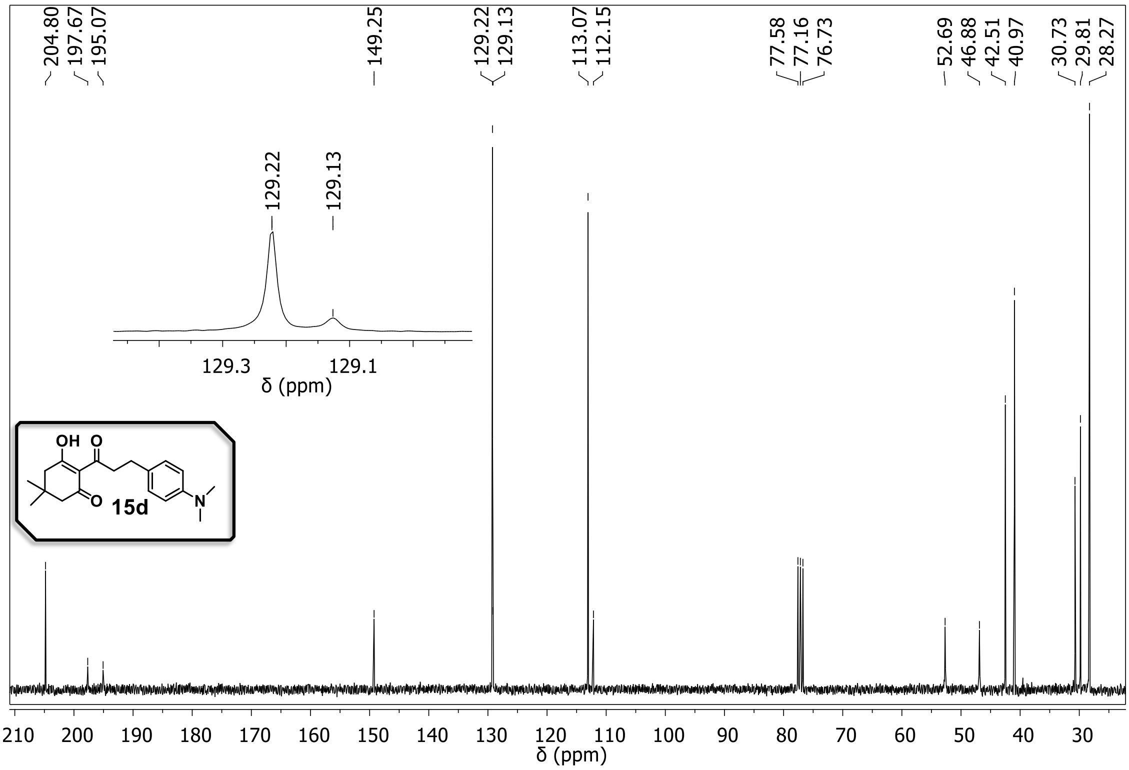

Figure S137. ^{13}C NMR (75 MHZ, $CDCl_3$) spectrum of **15d**.

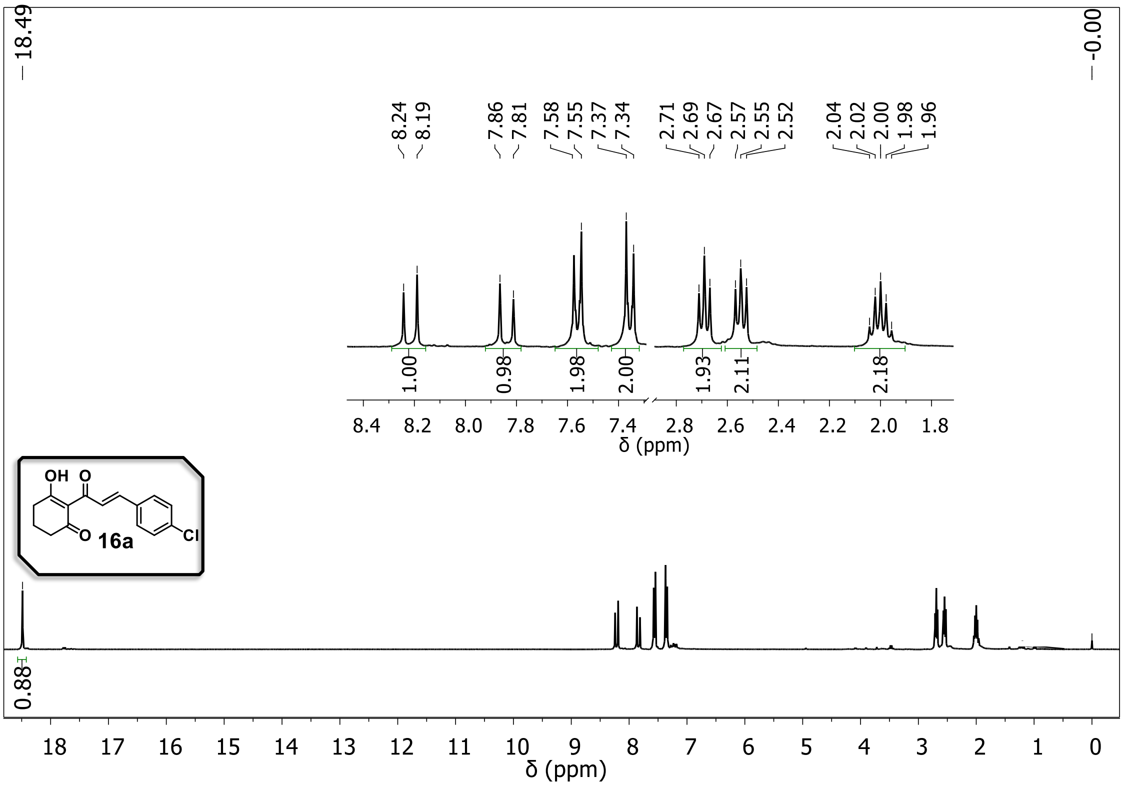

Figure S138. ^{1}H NMR (300 MHz, $CDCl_3$) spectrum of **16a**.

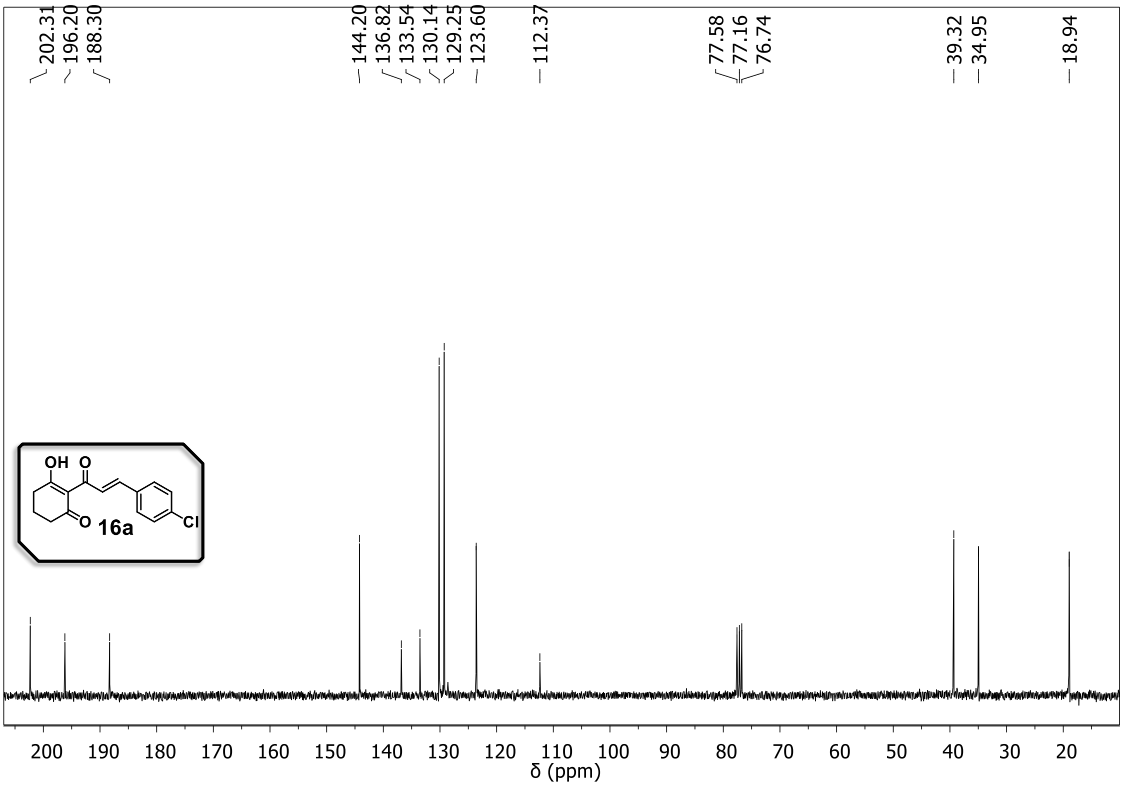

Figure S139. ^{13}C NMR (75 MHZ, $CDCl_3$) spectrum of **16a**.

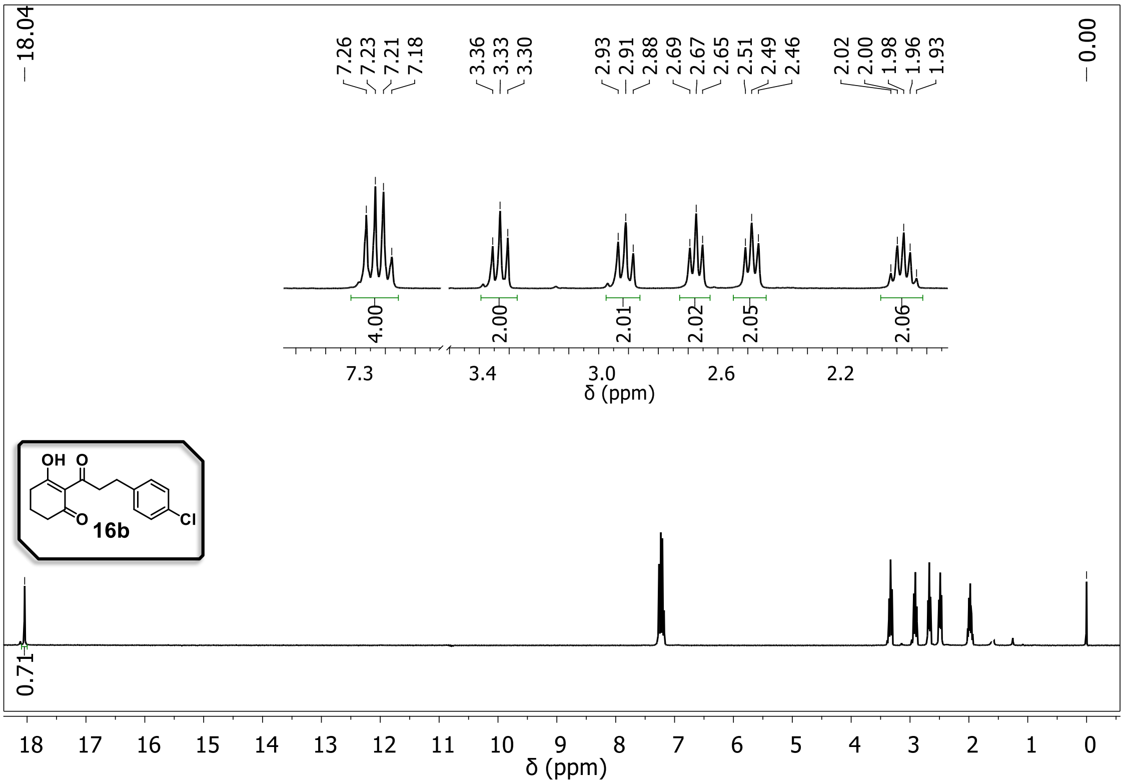

Figure S140. ^{1}H NMR (300 MHz, $CDCl_3$) spectrum of **16b**.

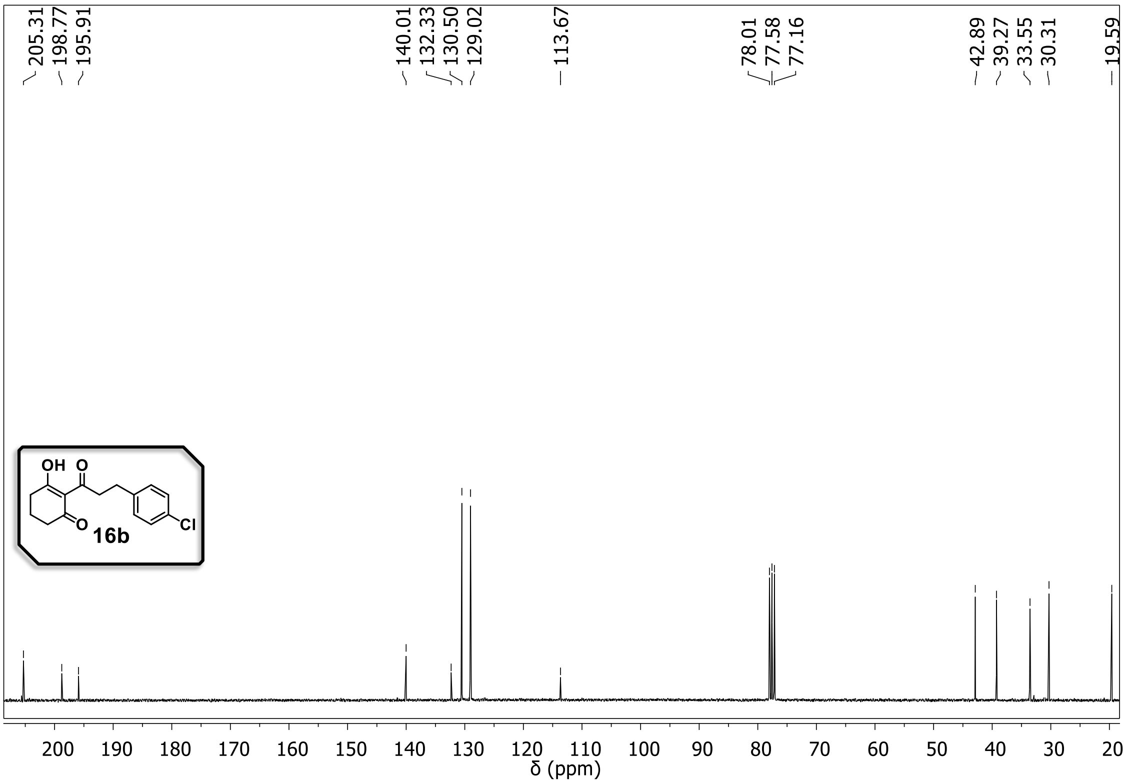

Figure S141. ^{13}C NMR (75 MHZ, $CDCl_3$) spectrum of **16b**.

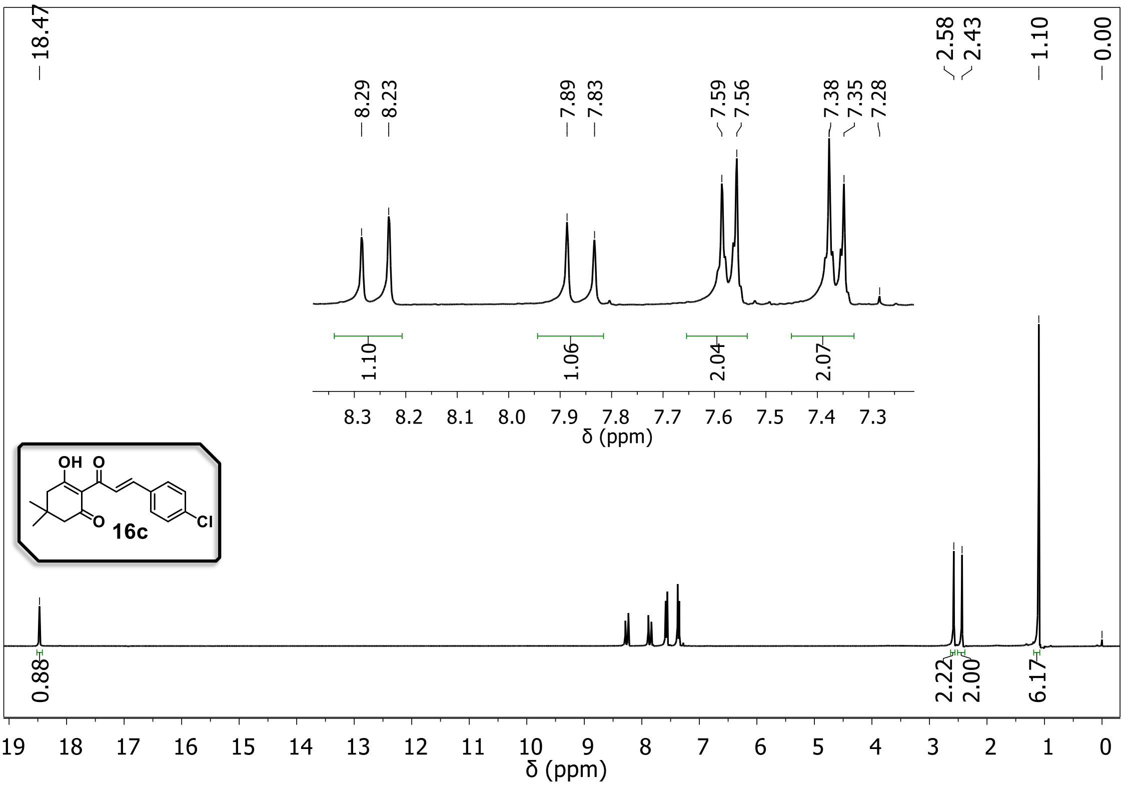

Figure S142. ^{1}H NMR (300 MHz, $CDCl_3$) spectrum of **16c**.

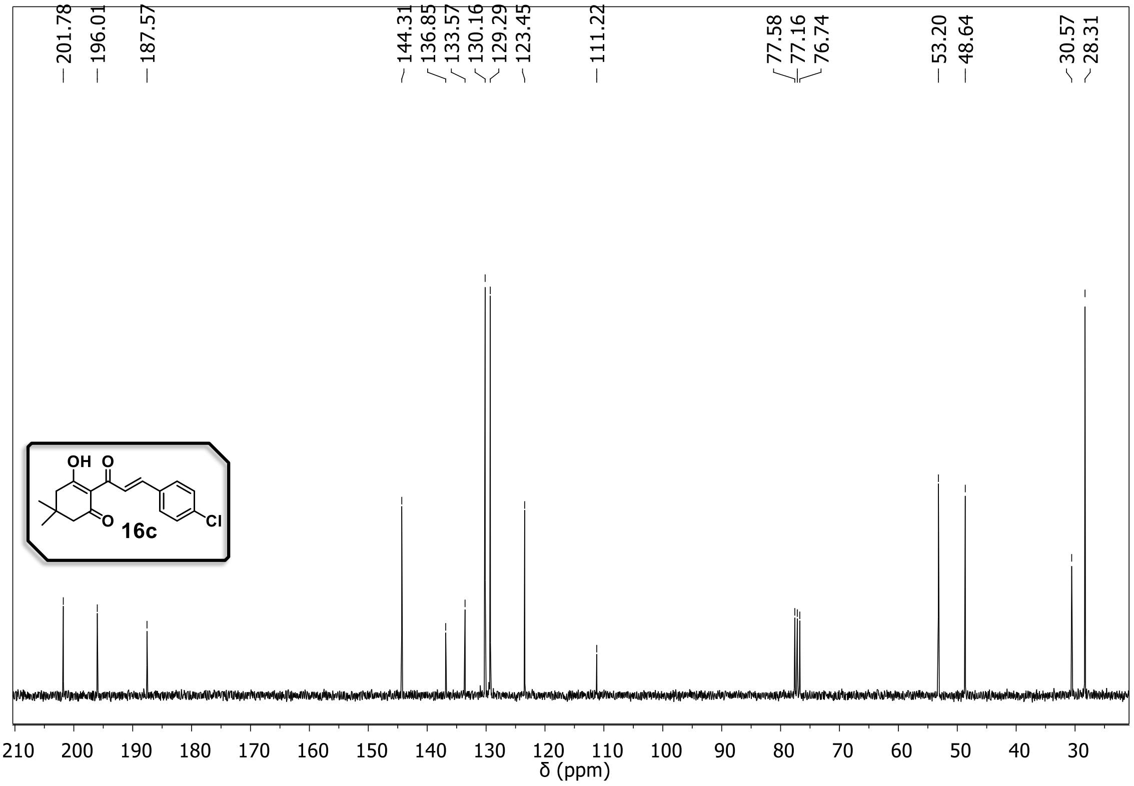

Figure S143. ^{13}C NMR (75 MHZ, $CDCl_3$) spectrum of **16c**.

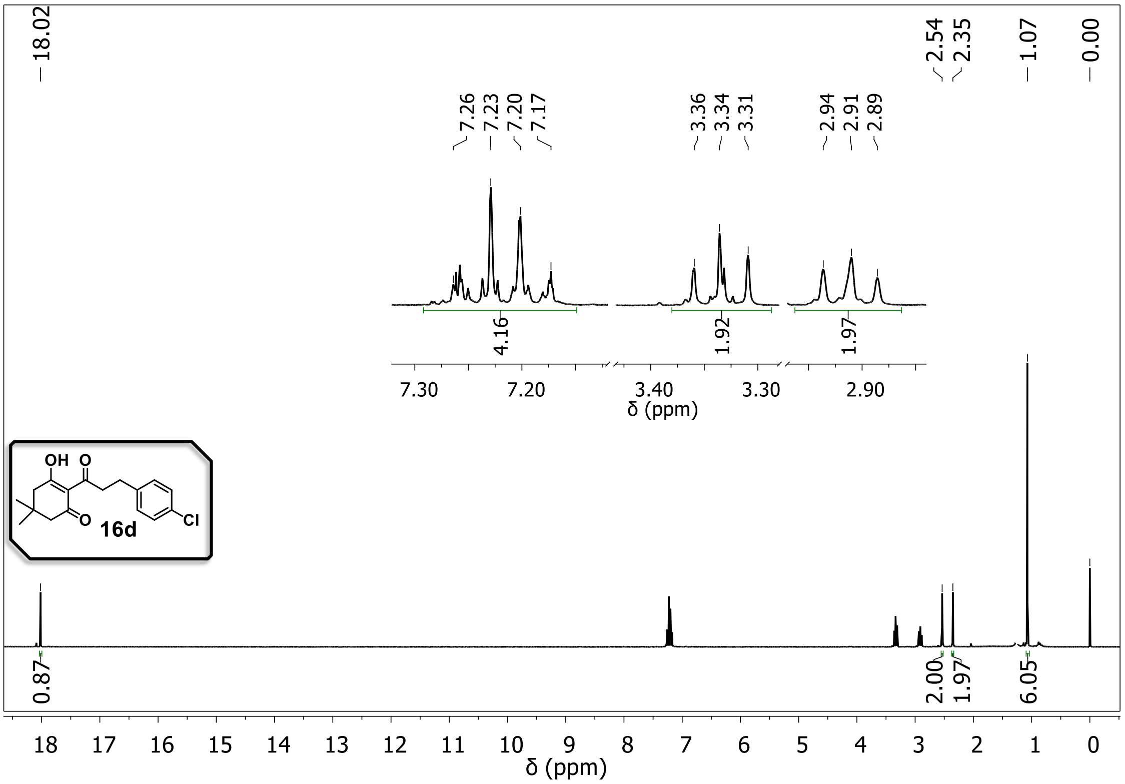

Figure S144. ^{1}H NMR (300 MHz, $CDCl_3$) spectrum of **16d**.

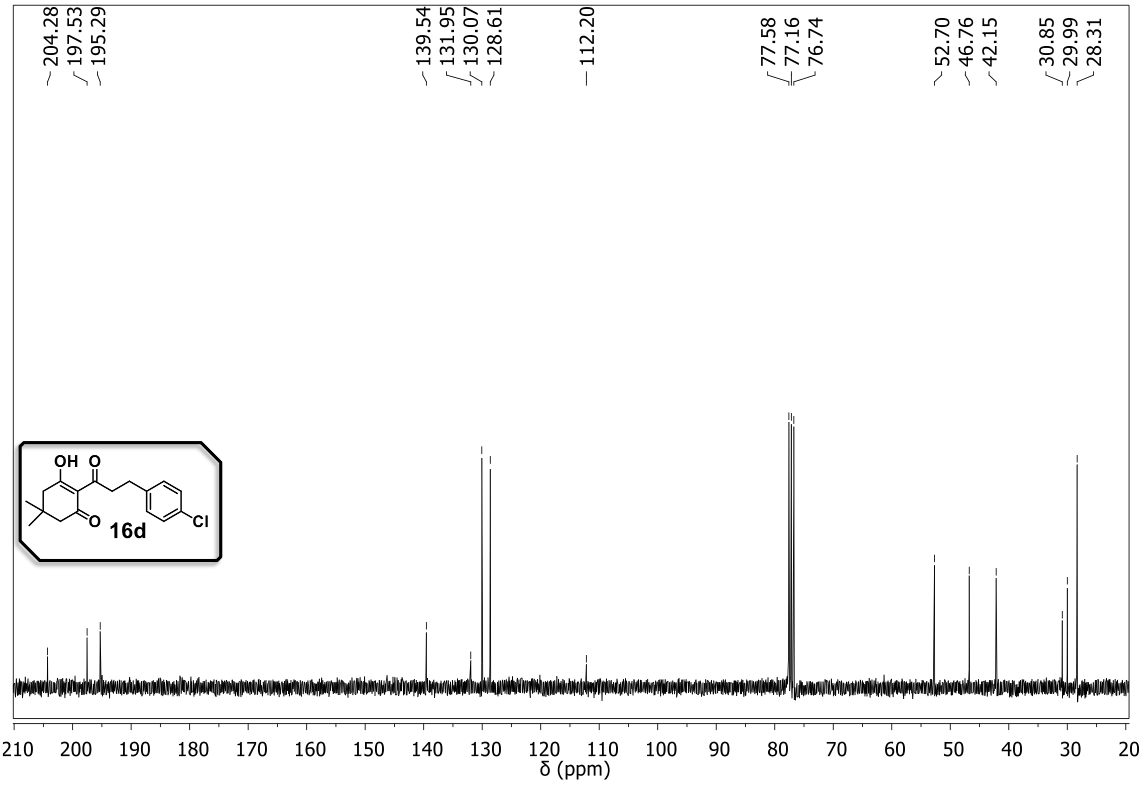

Figure S145. ^{13}C NMR (75 MHZ, $CDCl_3$) spectrum of **16d**.

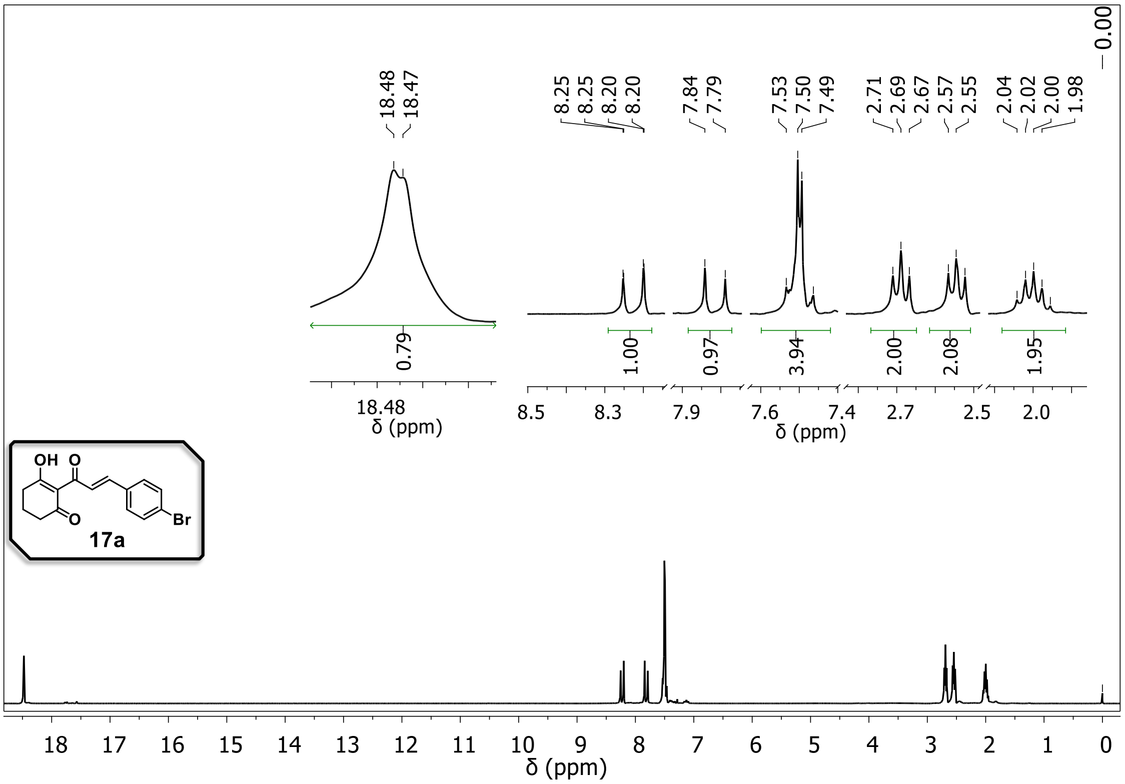

Figure S146. ^{1}H NMR (300 MHz, $CDCl_3$) spectrum of **17a**.

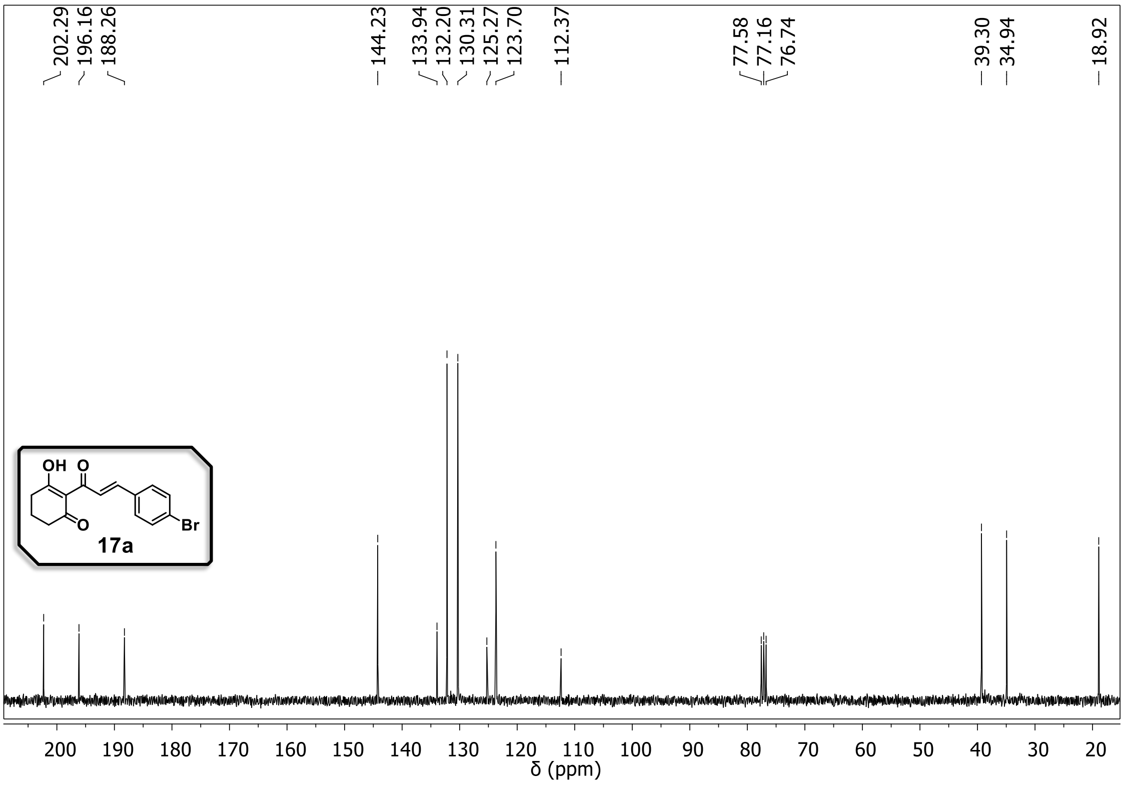

Figure S147. ^{13}C NMR (75 MHZ, $CDCl_3$) spectrum of **17a**.

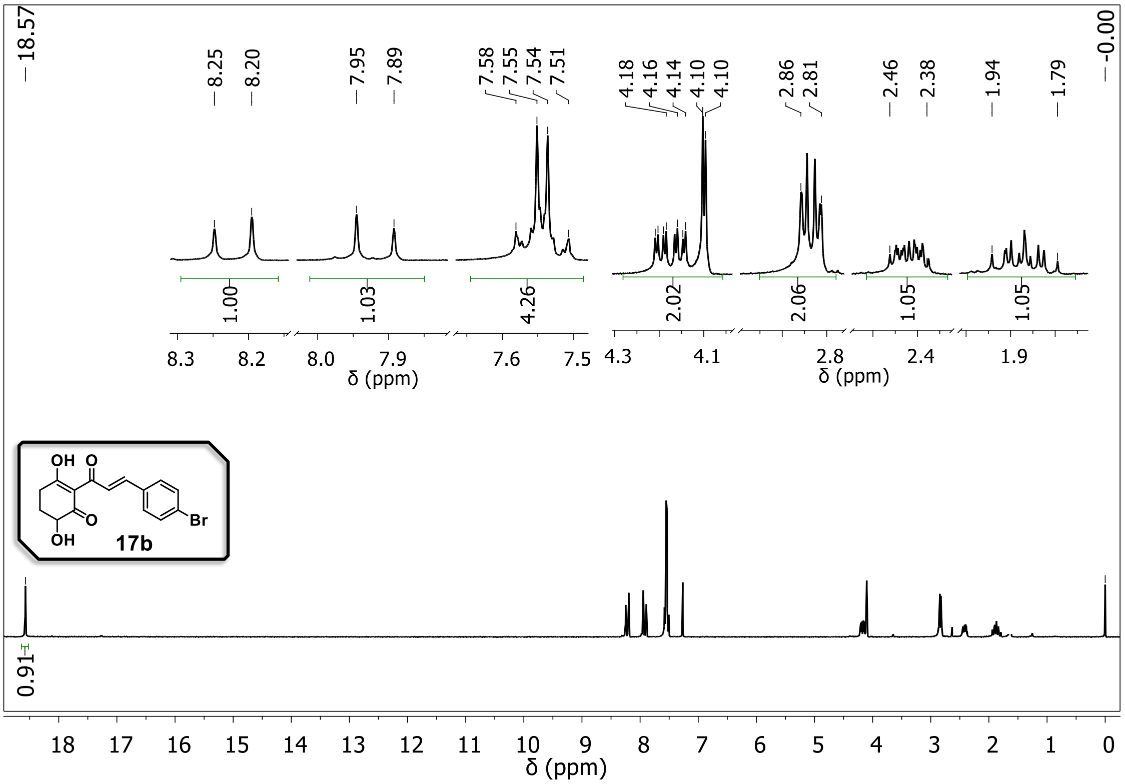

Figure S148. ^{1}H NMR (300 MHz, $CDCl_3$) spectrum of **17b**.

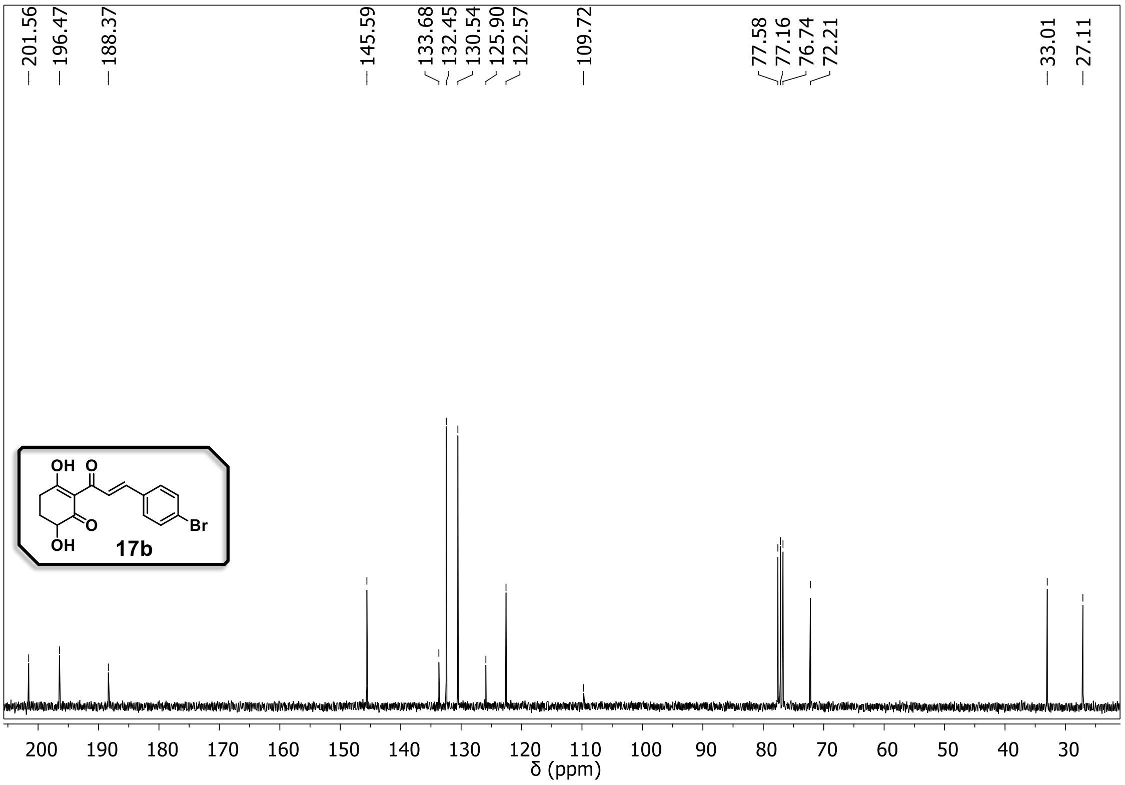

Figure S149. ^{13}C NMR (75 MHZ, $CDCl_3$) spectrum of **17b**.

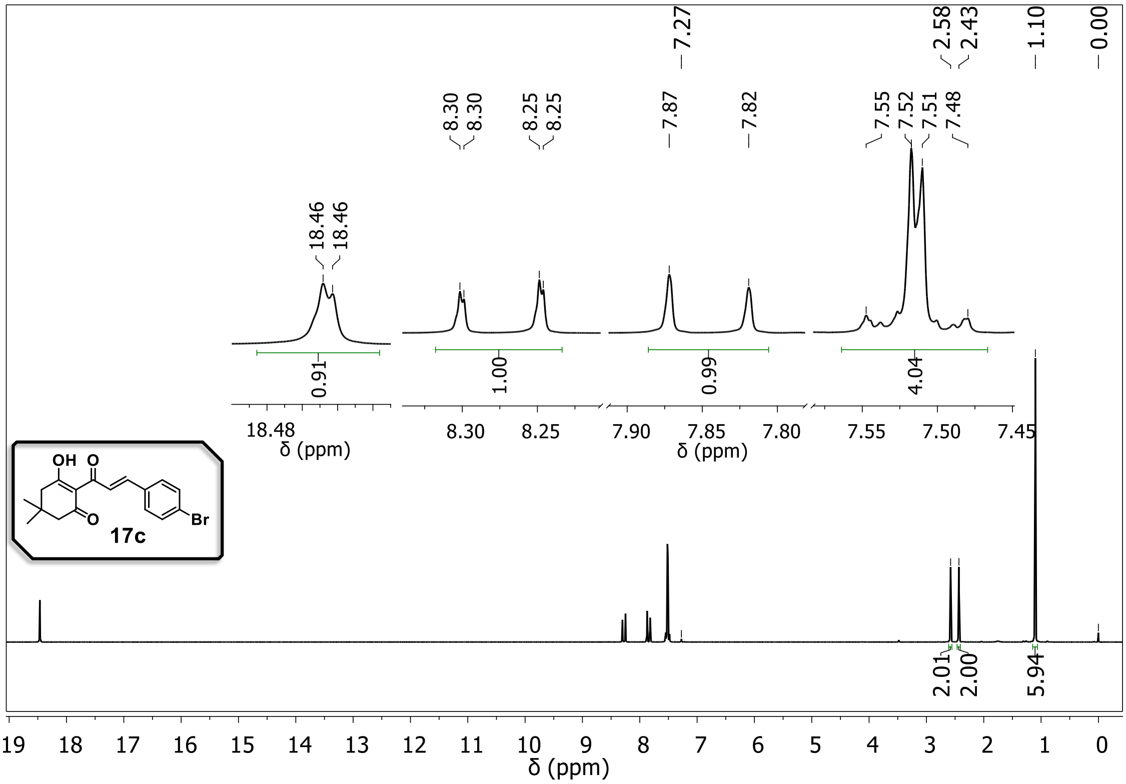

Figure S150. ^{1}H NMR (300 MHz, $CDCl_3$) spectrum of **17c**.

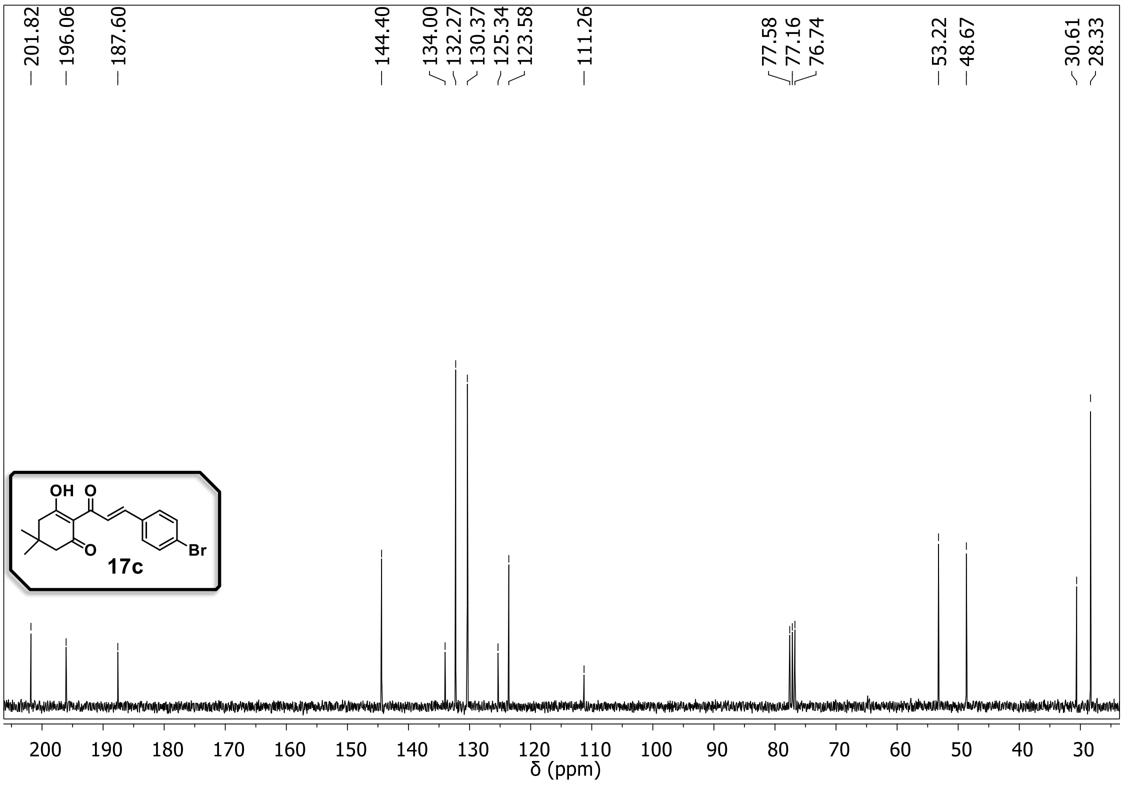

Figure S151. ^{13}C NMR (75 MHZ, $CDCl_3$) spectrum of **17c**.

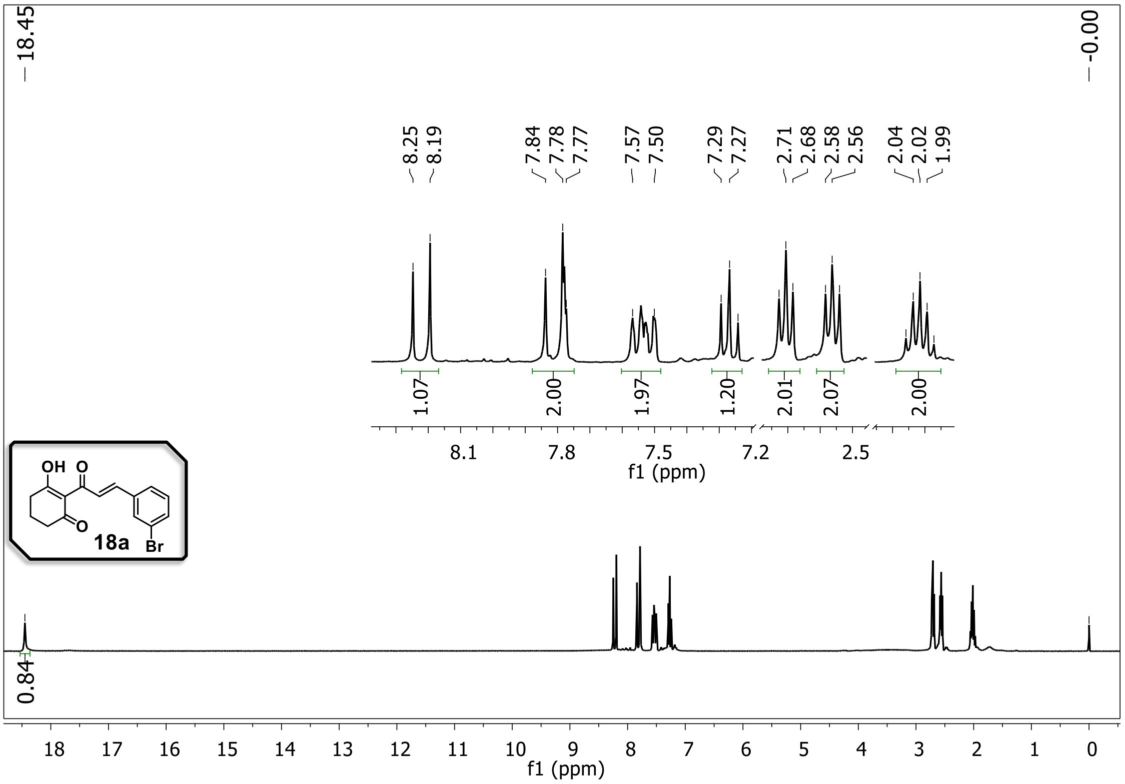

Figure S152. ^{1}H NMR (300 MHz, $CDCl_3$) spectrum of **18a**.

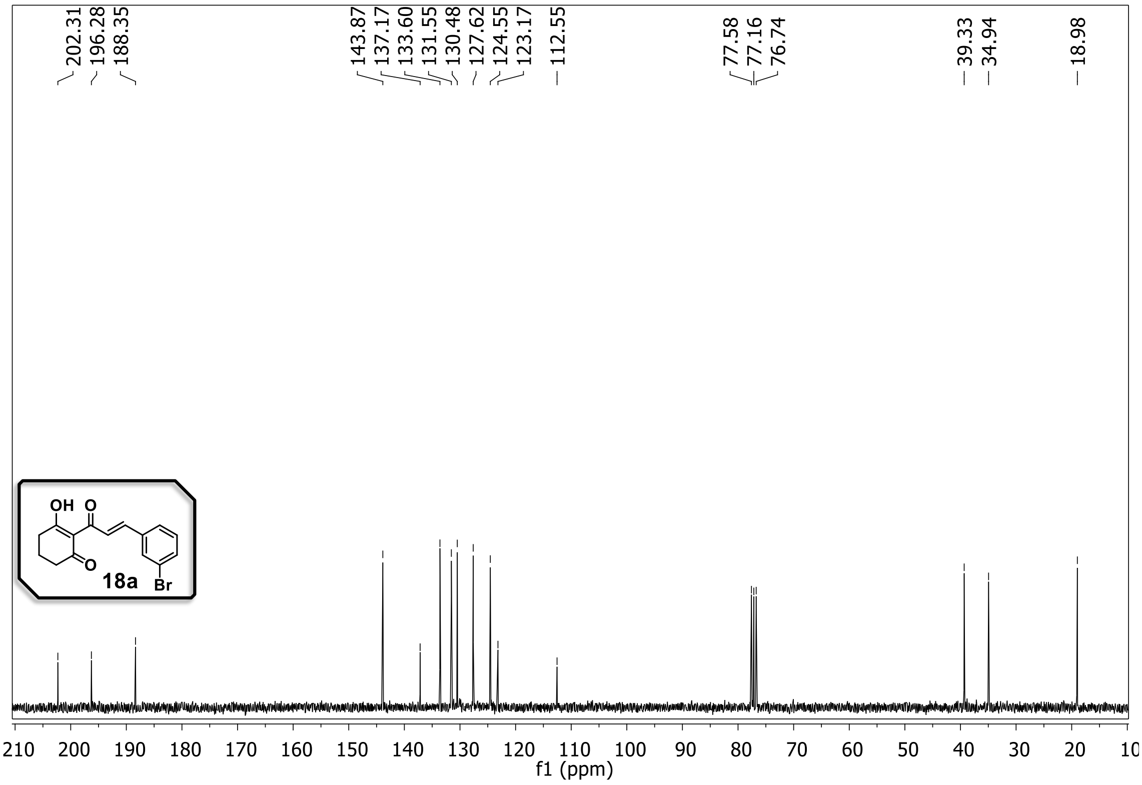

Figure S153. ^{13}C NMR (75 MHZ, $CDCl_3$) spectrum of **18a**.

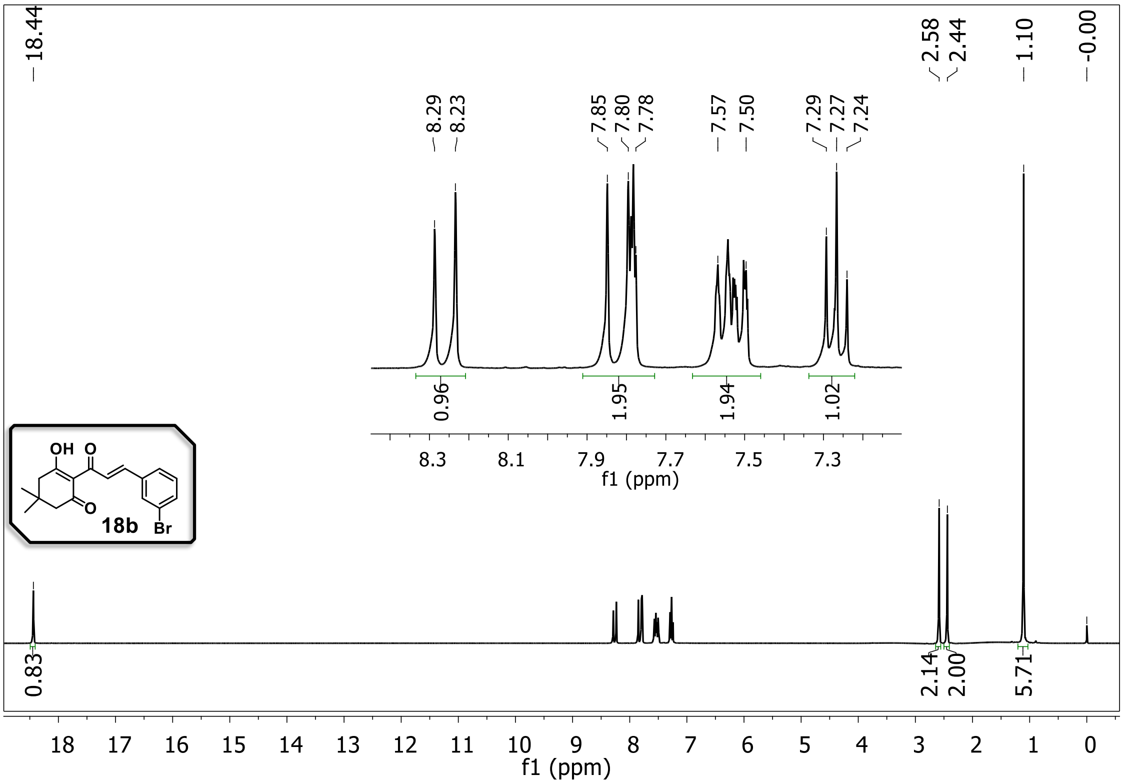

Figure S154. ^{1}H NMR (300 MHz, $CDCl_3$) spectrum of **18b**.

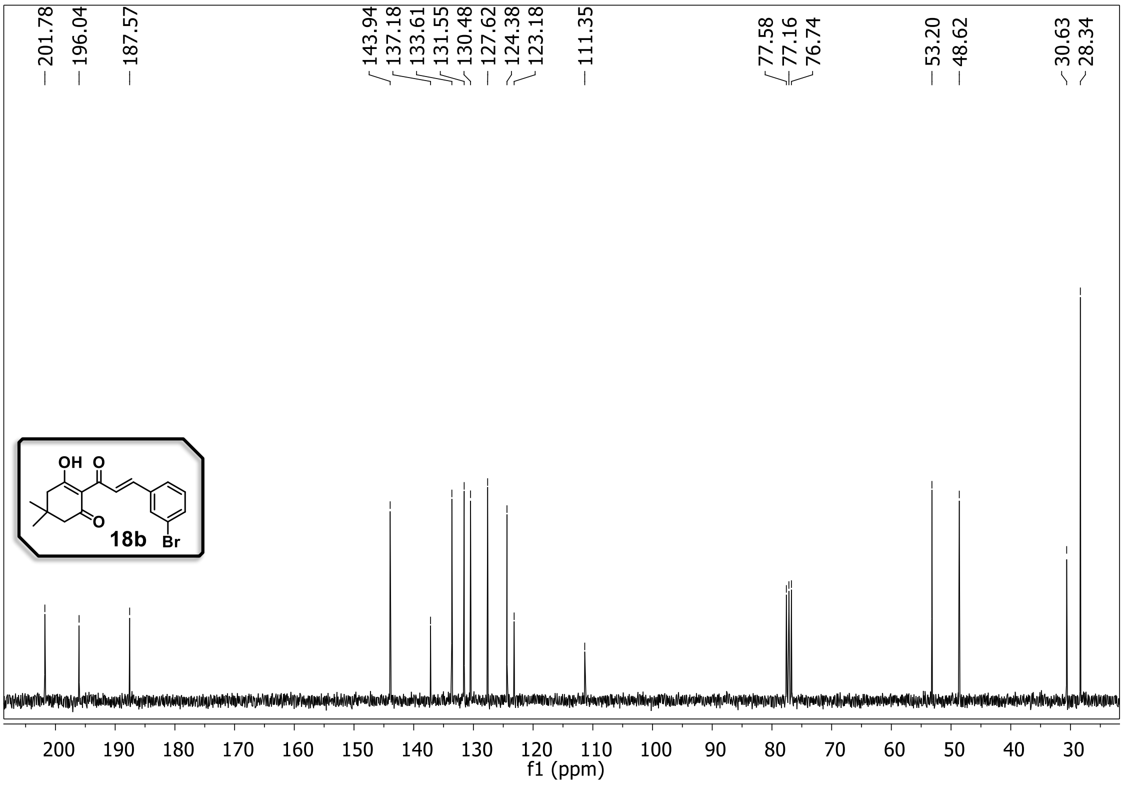

Figure S155. ^{13}C NMR (75 MHZ, $CDCl_3$) spectrum of **18b**.

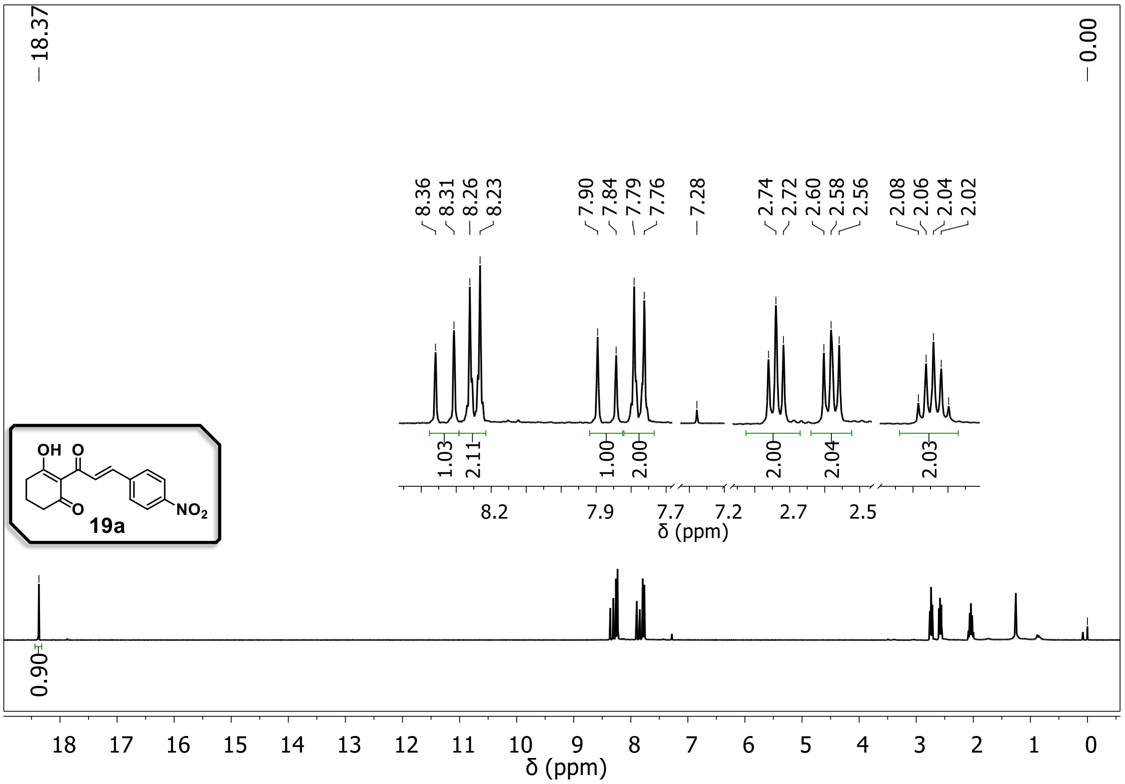

Figure S156. ^{1}H NMR (300 MHz, $CDCl_3$) spectrum of **19a**.

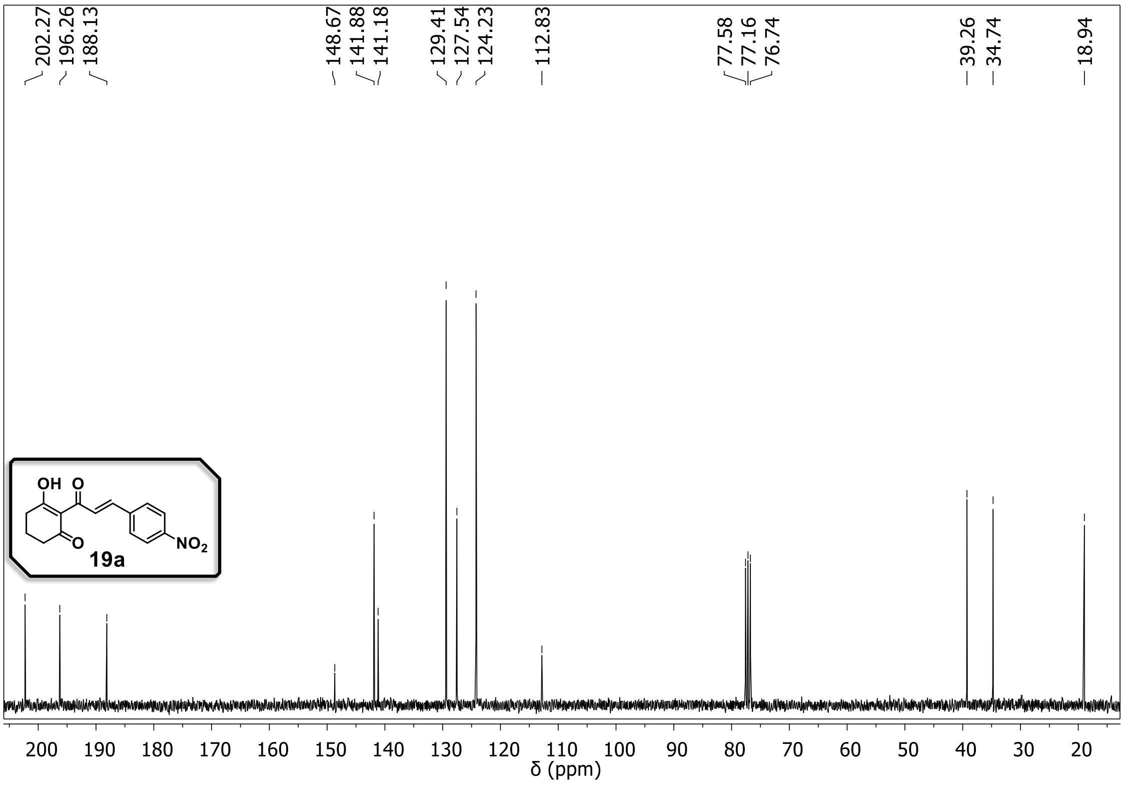

Figure S157. ^{13}C NMR (75 MHZ, $CDCl_3$) spectrum of **19a**.

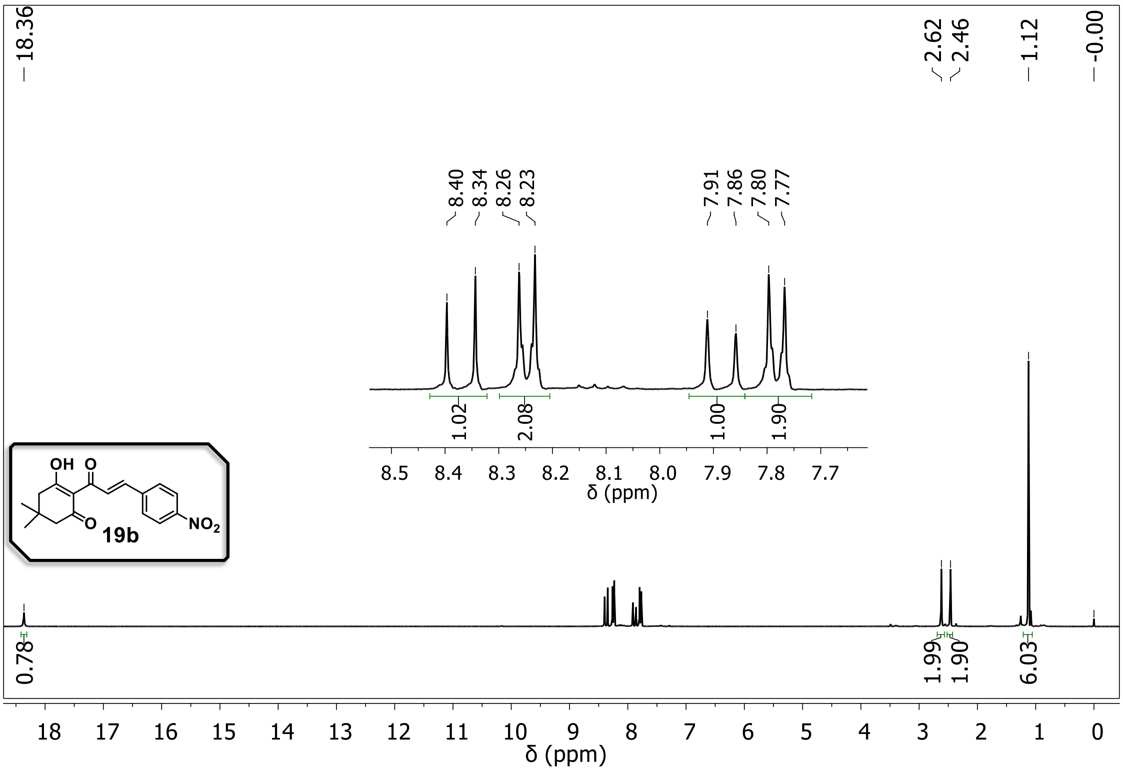

Figure S158. ^{1}H NMR (300 MHz, $CDCl_3$) spectrum of **19b**.

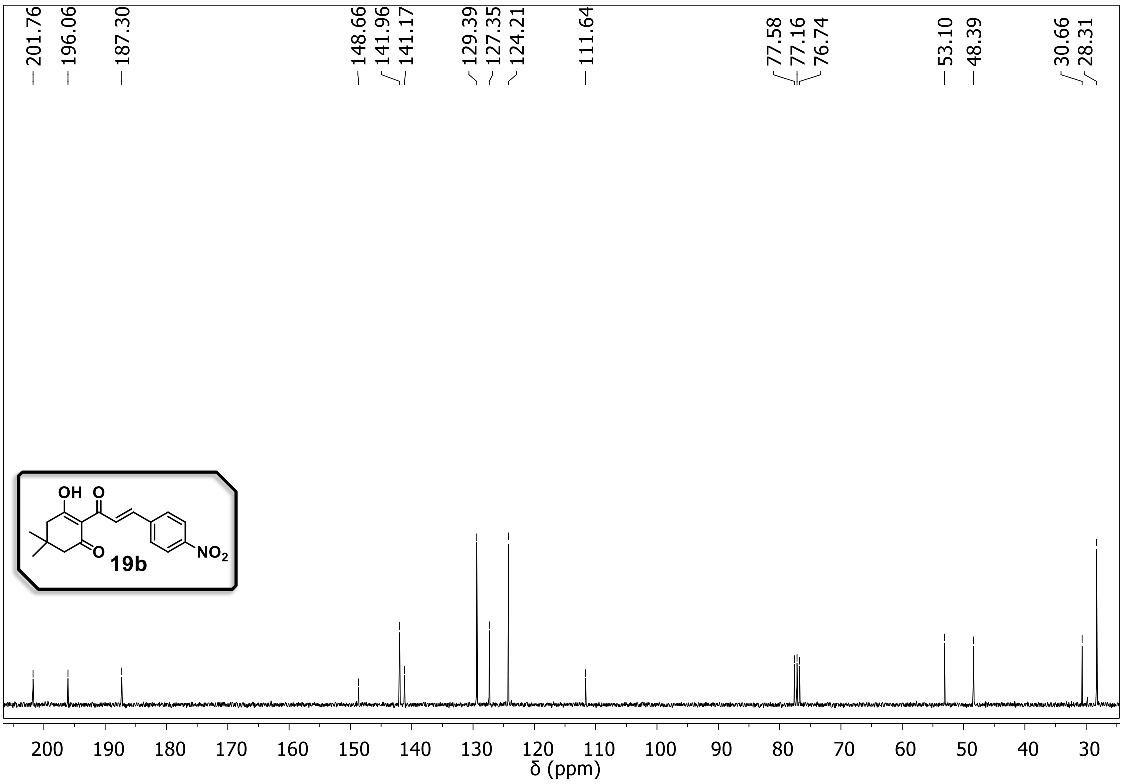

Figure S159. ^{13}C NMR (75 MHZ, $CDCl_3$) spectrum of **19b**.

Synthesis schemes for compounds

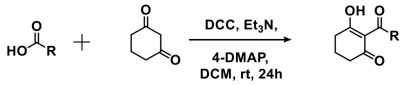

Entry	Carboxylic acid	Product
1	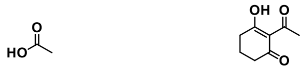	1a
2	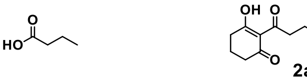	2a
3	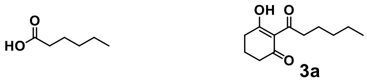	3a
4	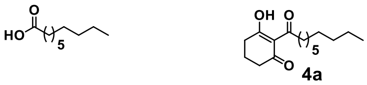	4a
5	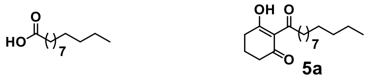	5a
6	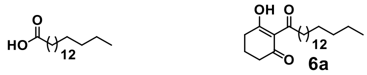	6a
7	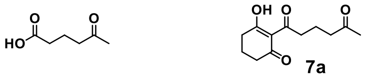	7a

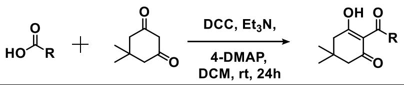

Entry	Carboxylic acid	Product
1'	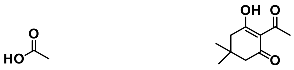	1e
2'	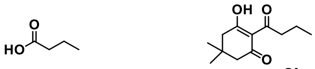	2b
3'	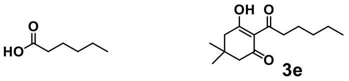	3e
4'	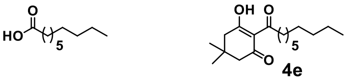	4e
5'	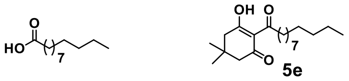	5e
6'	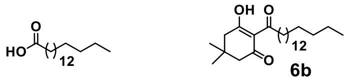	6b
7'	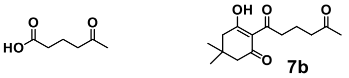	7b

Entry	Carboxylic acid	Product
8	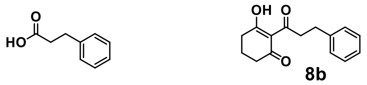	8b
9	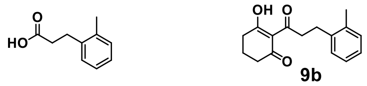	9b
10	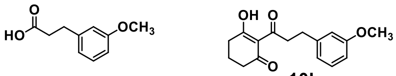	10b
11	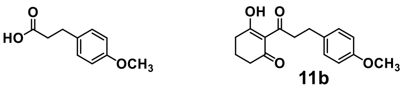	11b
12	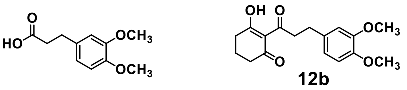	12b
13	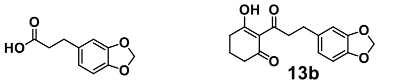	13b
14	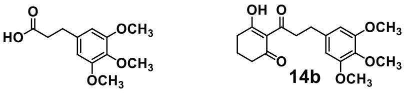	14b

Entry	Carboxylic acid	Product
8'	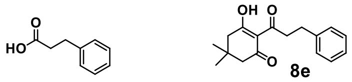	8e
9'	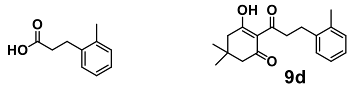	9d
10'	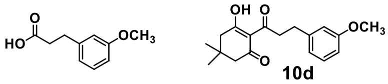	10d
11'	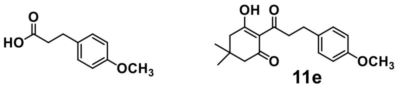	11e
12'	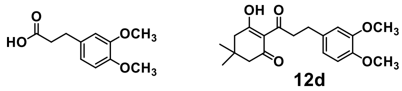	12d
13'	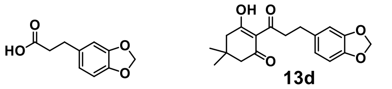	13d
14'	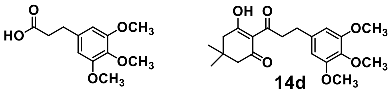	14d

Supplemental Scheme S1. Synthesis of 2-acyl-cyclohexane-1,3-dione

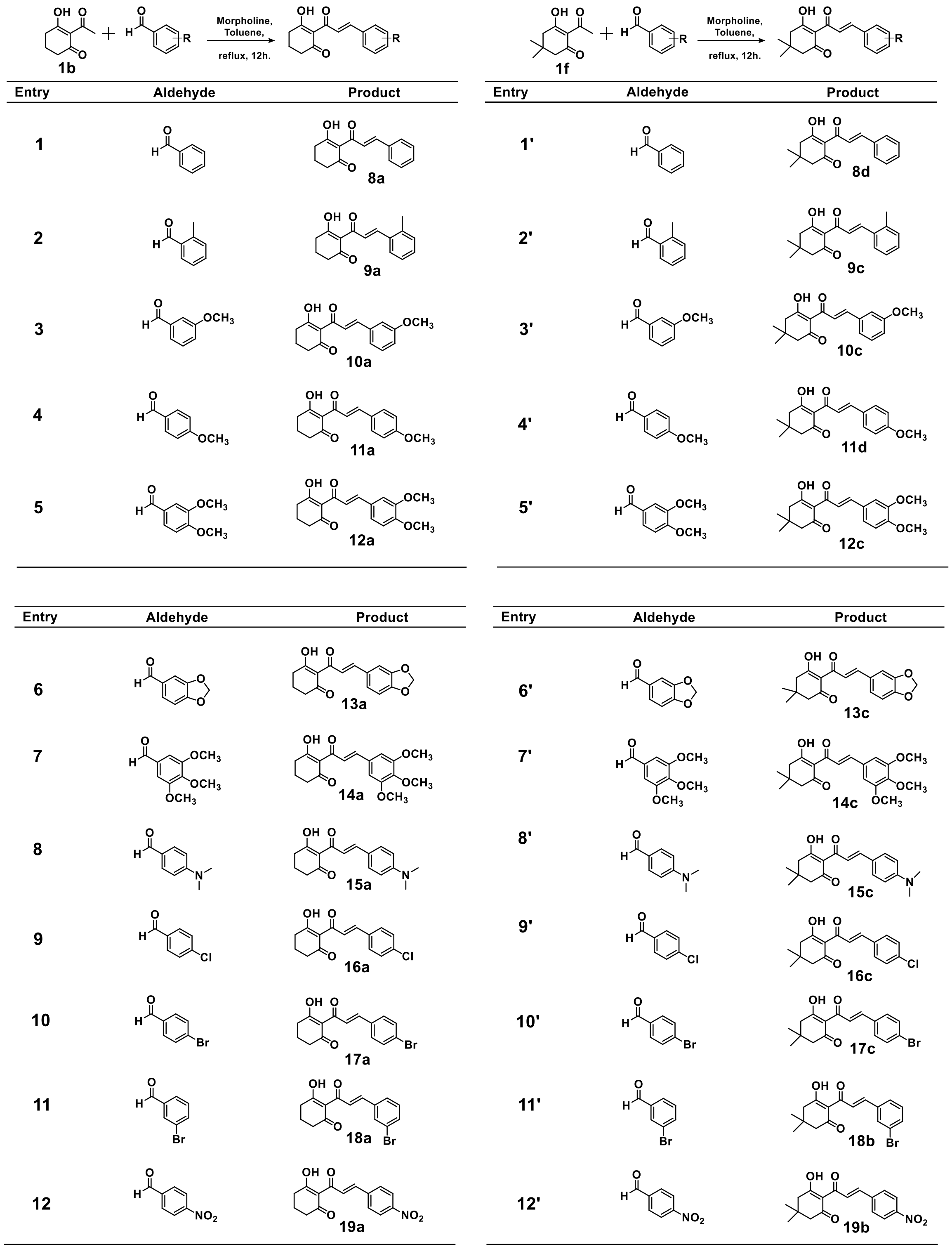

Supplemental Scheme S2. Unsaturated 2-acyl-cyclohexane-1,3-diones synthesized by aldol condensation

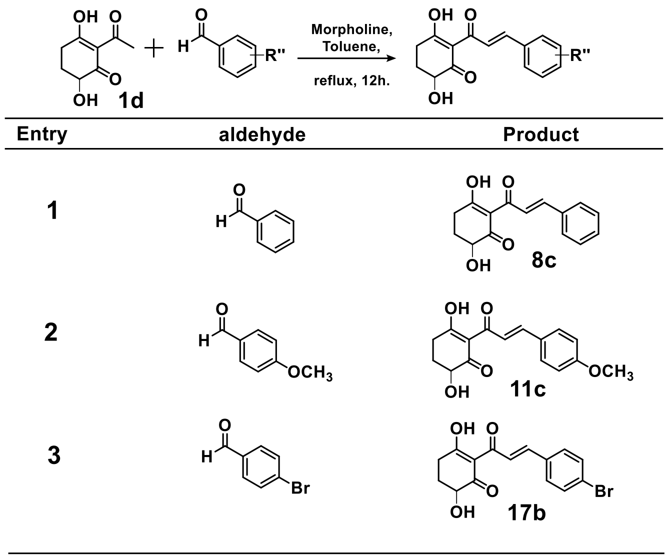

Supplemental Scheme S3. Hydroxylated 2-acyl-cyclohexane-1,3-diones synthesized by aldol condensation

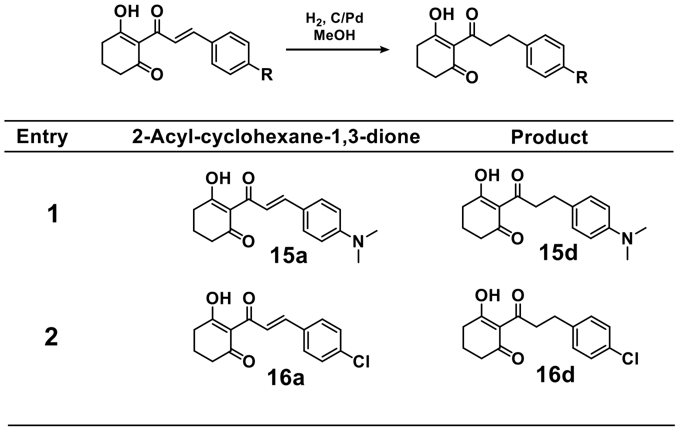

Supplemental Scheme S4. Hydrogenation of unsaturated 2-acyl-cyclohexane-1,3-diones

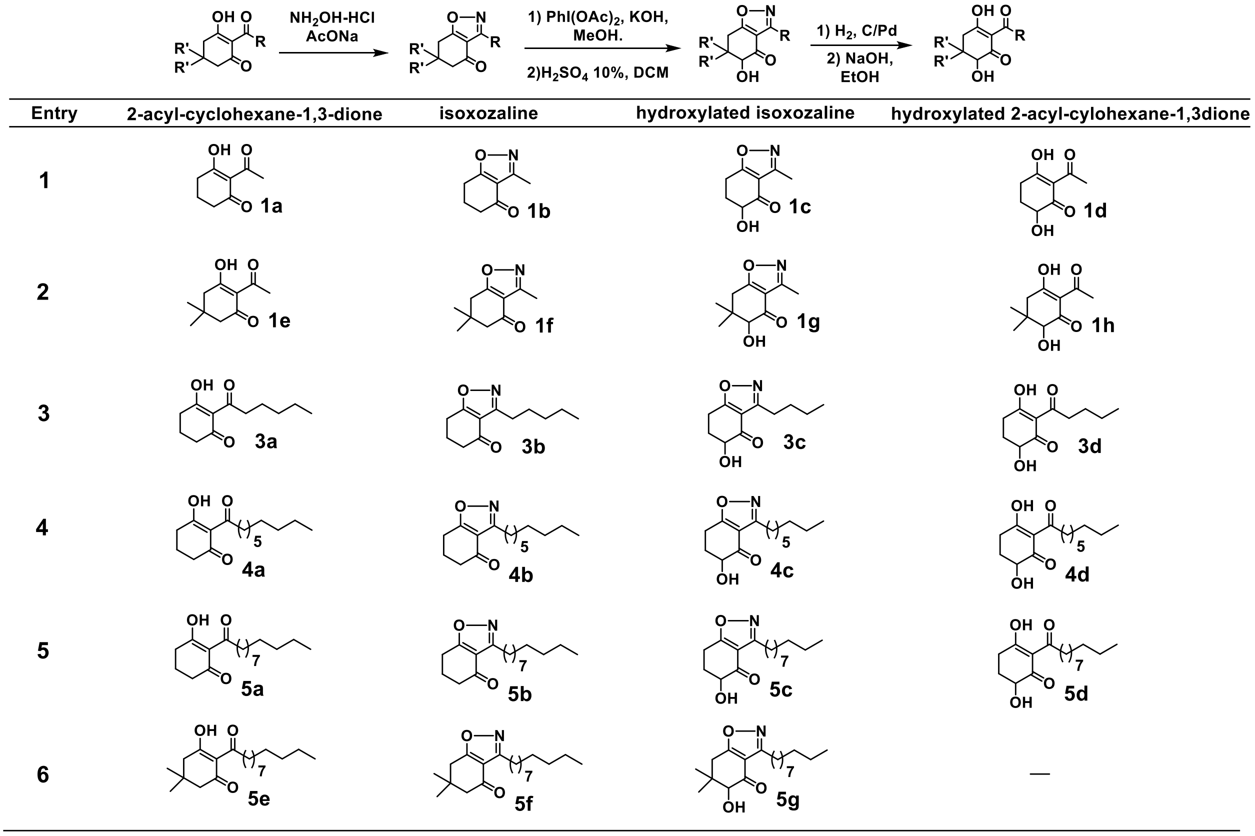

Supplemental Scheme S5. Hydroxylated 2-acyl-cyclohexane-1,3-diones and isoxazolines derivatives.

Spectroscopic data of compounds

Common moieties and numbering to all synthesized 2-acyl-cyclohexane-1,3-diones

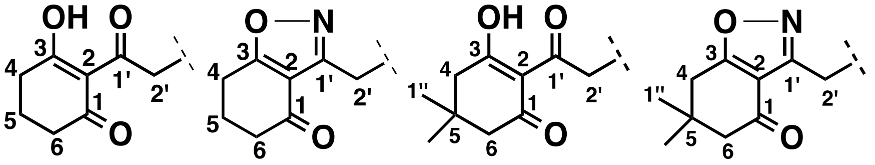

Compound 1a. Yellow liquid. IR (KBr, √max, cm^{-1}): 3315, 2957, 1666, 1566, 1556, 1445, 1420, 1190. 1H NMR (500 MHz, CDCl3): δ 18.12 (s, 1H, OH, enol), 2.67 (t, 6.4, 2H, H-4), 2.60 (s, 3H, H-2'), 2.50 (t, 6.5, 2H, H-6), 1.99 (quint, 6.5, 2H, H-5). ^{13}C NMR (75 MHZ, CDCl3) (125 MHz, $CDCl_3$): δ 203.07 (C-1'), 198.65 (C-3), 195.36 (C-1), 113.38 (C-2), 38.58 (C-4), 33.23 (C-6), 28.78 (C-2'), 18.98 (C-5). EIMS *m/z* (Rel. int. %): [M$^+$] 154 (55), 139 (25), 126 (25), 111 (15), 98 (100), 84 (30), 69 (28), 55 (20), 43 (52). HRESIMS (*m/z*): calcd. for $C_8H_{10}O_3$[M + H]+: 155.0708, found: 155.0703. Yield: 78%.

Compound 1b: Colorless liquid. IR (KBr, √max, cm^{-1}): 2966, 1672, 1599, 1469, 1425, 1073. ^{1}H NMR (500 MHz, $CDCl_3$): δ 2.98 (t, 6.3, 2H, H-4), 2.51 (t, 6.3, 2H, H-6), 2.48 (s, 3H, H-2'), 2.22 (quint, 6.3, 2H, H-5). ^{13}C NMR (75 MHZ, CDCl3) (125 MHz, $CDCl_3$): δ 193.43 (C-1), 181.02 (C-3), 157.58 (C-1'), 115.23 (C-2), 38.09 (C-6), 23.24 (C-4), 22.58 (C-5), 10.96 (C-2'). EIMS *m/z* (Rel. int. %): [M$^+$] 151 (50), 123 (100), 96 (9), 81 (71), 67 (25), 53 (6), 42 (16). HRESIMS (*m/z*): calcd. for $C_8H_9NO_2$[M + H]+: 152.0706, found: 152.0700. Yield: 82%.

Compound 1c: Pale gray solid. IR (KBr, √max, cm^{-1}): 3460, 2954, 1684, 1599, 1478, 1457, 1420, 1116. ^{1}H NMR (500 MHz, $CDCl_3$): δ 4.30 (dd, 12.6, 5.1, 1H, H-4), 3.74 (s, 1H, OH), 3.18-3.02 (m, 2H, H-6), 2.63-2.58 (m, 1H, H-5), 2.48 (s, 3H, H-2'), 2.16-2.07 (m, 1H, H-5). ^{13}C NMR (75 MHZ, CDCl3) (125 MHz, $CDCl_3$): δ 193.50 (C-1), 181.01 (C-3), 157.62 (C-1'),

113.68 (C-2), 73.05 (C-4), 30.54 (C-6), 21.86 (C-5), 10.68 (C-2'). EIMS *m/z* (Rel. int. %): [M^+] 167 (19), 138 (19), 123 (100), 81 (44), 67 (14), 55 (8), 57 (8), 42 (6). HRESIMS (*m/z*): calcd. for $C_8H_9NO_3$[M + H]+: 168.0661, found: 168.0649. Yield: 74%.

Compound 1d: Yellow solid. IR (KBr, ν_{max}, cm^{-1}): 3450, 2960, 1660, 1533, 1478, 1441, 1420, 1125. ^1H NMR (500 MHz, $CDCl_3$): δ 18.10 (s, 1H, OH, enol), 4.10 (ddd, 12.6, 5.6, 1.5, 1H, H-4), 4.00 (d, 1.5, 1H, OH), 2.82-2.78 (m, 2H, H-6), 2.63 (s, 3H, H-2'), 2.42-2.37 (m, 1H, H-5), 1.88-1.79 (m, 1H, H-5). ^{13}C NMR (75 MHZ, CDCl3) (125 MHz, $CDCl_3$): δ 203.10 (C-1'), 198.02 (C-3), 195.87 (C-1), 110.88 (C-2), 71.75 (C-4), 31.43 (C-6), 28.52 (C-5), 27.28 (C-2'). EIMS *m/z* (Rel. int. %): [M^+] 170 (20), 142 (25), 126 (40), 109 (6), 98 (100), 84 (30), 70 (28), 57 (7), 55 (12), 43 (77), 39 (9). HRESIMS (*m/z*): calcd. for $C_8H_{10}O_4$ [M + H]+: 171.0657, found: 171.0648. Yield: 94%.

Compound 1e: Yellow liquid. IR (KBr, ν_{max}, cm^{-1}): 3309, 2961, 1729, 1668, 1566, 1556, 1446, 1421, 1150. ^1H NMR (300 MHz, $CDCl_3$): δ 18.11 (s, 1H, OH, enol), 2.61 (s, 3H, H-2'), 2.54 (s, 2H, H-6), 2.37 (s, 2H, H-4), 1.09 (s, 6H, H-1''). ^{13}C NMR (75 MHZ, CDCl3) (75 MHz, $CDCl_3$): δ 202.45 (C-1'), 197.94 (C-3), 195.23 (C-1), 112.37 (C-2), 52.47 (C-5), 46.91 (C-6), 30.68 (C-4), 28.56 (C-2'), 28.22 (C-1''). EIMS *m/z* (Rel int. %): [M^+] 182 (35), 167 (15), 154 (5), 126 (50), 111 (6), 98 (100), 83 (23), 70 (19), 55 (15), 43 (45), 39 (10). HRESIMS (*m/z*): calcd. for $C_{10}H_{14}O_3$ [M + H]+: 183.1021, found: 183.1011. Yield: 88%.

Compound 1f: Pale gray solid. IR (film, ν_{max}, cm^{-1}): 2958, 1685, 1603, 1473, 1426, 1074. ^1H NMR (300 MHz, $CDCl_3$): δ 2.76 (s, 3H, H-4), 2.38 (s, 2H, H-2"), 2.31 (s, 2H, H-6), 1.08 (s, 6H, H-1''). ^{13}C NMR (75 MHZ, CDCl3) (75 MHz, $CDCl_3$): δ 192.53 (C-1), 180.34 (C-3), 157.08 (C-1'), 113.85 (C-2), 52.22 (C-5), 36.58 (C-4), 35.51 (C-6), 28.25 (C-2'), 13.71 (C-1''). EIMS *m/z* (Rel. int. %): [M^+] 179 (43), 164 (9), 136 (6), 123 (100), 95 (7), 81 (48), 67

(20), 55 (15), 41 (13), 39 (12). HRESIMS (*m/z*): calcd. for $C_{10}H_{13}NO_2$[M + H]+: 180.1025, found: 180.1017. Yield: 86%.

Compound 1g: Yellow solid. IR (KBr, ν_{max}, cm^{-1}): 3411, 2967, 1676, 1603, 1474, 1422, 1389, 1141. ^{1}H NMR (300 MHz, $CDCl_3$): δ 4.08 (d, 2.2, 1H, H-4), 3.68 (d, 2.2, 1H, OH), 2.93 (s, 2H, H-6), 2.49 (s, 3H, H-2'), 1.35 (s, 3H, H-1''), 0.90 (s, 3H, H-1''). ^{13}C NMR (75 MHZ, CDCl3) (75 MHz, $CDCl_3$): δ 192.96 (C-1), 179.98 (C-3), 157.49 (C-1'), 112.84 (C-2), 80.57 (C-4), 41.31 (C-6), 36.57 (C-5), 27.74 (C-2'), 18.98 (C-1''), 10.70 (C-1''). EIMS *m/z* (Rel. int.%): [M^+] 195 (10), 177 (5), 166 (5), 152 (8), 123 (100), 111 (4), 82 (20), 67 (15), 55 (17), 43 (16), 41 (17). HRESIMS (*m/z*): calcd. for $C_{10}H_{13}NO_3$[M +H]+: 196.0974, found: 196.0970. Yield: 75%.

Compound 1h: Yellow solid**. IR (**KBr, ν_{max}, cm^{-1}): 3424, 2965, 1671, 1559, 1449, 1415, 1137. ^{1}H NMR (300 MHz, $CDCl_3$): δ 17.96 (s, 1H, OH, enol), 3.97 (s, 1H, H-4), 3.88 (s, 1H, OH), 2.71-2.46 (m, 2H, H-6), 2.56 (s, 3H, H-2'), 1.18 (s, 3H, H-1''), 0.82 (s, 3H, H-1''. ^{13}C NMR (75 MHZ, CDCl3) (75 MHz, $CDCl_3$): δ 202.38 (C-1'), 196.65 (C-3), 195.44 (C-1), 110.56 (C-2), 79.29 (C-4), 46.07 (C-6), 36.44 (C-5), 28.00 (C-2'), 27.43 (C-1''), 18.83 (C-1''). EIMS *m/z* (Rel. int.%): [M^+] 198 (10), 180 (25), 169 (20), 165 (8), 127 (100), 98 (45), 85 (95), 72 (42), 69 (15), 57 (12), 43 (45), 41 (10). HRESIMS (*m/z*): calcd. for $C_{10}H_{14}O_4$[M +H]+: 199.0970, found: 199.0962. Yield: 90%.

Compound 2a. Pale yellow liquid. IR (KBr, ν_{max}, cm^{-1}): 3525, 2962, 1732, 1667, 1567, 1557, 1461, 1418, 1191. ^{1}H NMR (300 MHz, $CDCl_3$): δ 18,28 (s, 1H, OH, enol), 3.00 (t, 7.2, 2H, H-2'), 2.67 (t, 6.8, 2H, H-4), 2.49 (t, 6.8, 2H, H-6), 1.98 (quint: 6.8, 2H, H-5), 1.65 (sext, 7.4, 2H, H-3'), 0.98 (t, 7.4, 3H, H-4'). ^{13}C NMR (75 MHZ, CDCl3) (75 MHz, $CDCl_3$): δ

206.17 (C-1'), 198.66 (C-3), 195.37 (C-1), 113.05 (C-2), 42.50 (C-2'), 38.83 (C-4), 33.29 (C-6), 19.04 (C-5), 18.04 (C-3'), 13.93 (C-4'). EIMS *m/z* (Rel int. %): [M^+] 182 (42), 167 (27), 165 (6), 154 (25), 139 (100), 126 (10), 111 (30), 98 (15), 84 (20), 69 (46), 55 (43), 43 (35), 39 (13). HRESIMS (*m/z*): calcd. for $C_{10}H_{14}O_3$ [M + H]+: 183.1015, found: 183.1016. Yield: 87%.

Compound 2b. Pale yellow liquid. IR (KBr, ν_{max}, cm^{-1}): 2962, 1668, 1560, 1448, 1421, 1084. ^{1}H NMR (300 MHz, $CDCl_3$): δ 18.25 (s, 1H, OH, enol), 3.01 (t, 7.4, 2H, H-2'), 2.53 (s, 2H, H-6), 2.35 (s, 2H, H-4), 1.66 (sext, 7.4, 2H, H-3'), 1.08 (s, 6H, H-1''), 0.98 (t, 7.4, 3H, H-4'). ^{13}C NMR (75 MHZ, CDCl3) (75 MHz, $CDCl_3$): δ 205.67 (C-1'), 197.90 (C-3), 195.22 (C-1), 112.11 (C-2), 52.81 (C-5), 47.05 (C-6), 42.34 (C-2'), 30.79 (C-4), 28.32 (C-3'), 18.25 (C-1''), 14.04 (C-4'). EIMS *m/z* (Rel int. %): [M^+] 210 (60), 195 (50), 177 (20), 167 (100), 154 (25), 140 (35), 126 (20), 121 (10), 111 (30), 98 (22), 83 (82), 69 (40), 55 (35), 43 (50), 41 (31), 39 (12). HRESIMS (*m/z*): calcd. for $C_{12}H_{18}O_3$ [M + H]+: 211.1328, found: 211.1330. Yield: 85%.

Compound 3a: Yellow liquid. IR (KBr, ν_{max}, cm^{-1}): 2956, 1734, 1667, 1567, 1557, 1461, 1421, 1190. ^{1}H NMR (300 MHz, $CDCl_3$): δ 18.27 (s, 1H, OH, enol), 3.01 (t, 7.4, 2H, H-2'), 2.67 (t, 6.8, 2H, H-4), 2.49 (t, 6.8, 2H, H-6), 1.98 (quint, 6.8, 2H, H-5), 1.67-1.57 (m, 2H, H-3'), 1.40-1.28 (m, 4H, H-4', H-5'), 0.92-0.88 (m, 3H, H-6'). ^{13}C NMR (75 MHZ, CDCl3) (75 MHz, $CDCl_3$): δ 206.40 (C-1'), 198.71 (C-3), 195.33 (C-1), 113.08 (C-2), 40.60 (C-2’), 38.91 (C-4), 33.40 (C-6), 31.63 (C-3'), 24.45 (C-4'), 22.55 (C-5'), 19.15 (C-5), 14.02 (C-6'). EIMS *m/z* (Rel int. %): [M^+] 210 (16), 192 (9), 181 (6), 167 (100), 154 (42), 149 (14), 139 (75), 126 (25), 121 (7), 111 (29), 97 (15), 84 (12), 79 (10), 69 (47), 55 (50), 43 (35), 41

(17). HRESIMS (*m/z*): calcd. for $C_{12}H_{18}O_3$ [M + H]+: 211.1328, found: 211.1329. Yield: 62%.

Compound 3b: Colorless liquid. IR (film, ν_{max}, cm^{-1}): 2957, 1688, 1600, 1466, 1430, 1071. ^1H NMR (300 MHz, $CDCl_3$): δ 2.98 (t, 6.4, 2H, H-2'), 2.85 (t, 7.6, 2H, H-4), 2.52 (t, 6.5, 2H, H-6), 2.22 (quint, 6.5, 2H, H-5), 1.76-1.67 (m, 2H, H-3'), 1.38-1.34 (m, 4H, H-4', H-5'), 0.92 (m, 3H, H-6'). ^{13}C NMR (75 MHZ, CDCl3) (75 MHz, $CDCl_3$): δ 193.20 (C-1), 181.14 (C-3), 161.47 (C-1'), 114.79 (C-2), 38.17 (C-4), 31.52 (C-6), 27.06 (C-2'), 25.58 (C-3'), 23.26 (C-4'), 22.48 (C-5'), 22.41 (C-5), 14.07 (C-6'). EIMS *m/z* (Rel. int.%): [M^+] 207 (10), 192 (8), 178 (27), 164 (81), 151 (23), 138 (100), 123 (22), 106 (20), 94 (12), 82 (9), 68 (26), 55 (47), 43 (46), 41 (65). HRESIMS (*m/z*): calcd. for $C_{12}H_{17}NO_2$[M + H]+: 208.1338, found: 208.1337. Yield: 80%.

Compound 3c: Yellow solid. IR (KBr, ν_{max}, cm^{-1}): 3487, 2957, 1680, 1594, 1474, 1426, 1385, 1121. ^1H NMR (300 MHz, $CDCl_3$): δ 4.29 (ddd, 12.7, 5.3, 1.0, 1H, H-4), 3.69 (d, 1.0, 1H, OH), 3.20-2.99 (m, 2H, H-6), 2.88-2.83 (m, 2H, H-2'), 2.65-2.56 (m, 1H, H-5), 2.18-2.03 (m, 1H, H-5), 1.77-1.68 (m, 2H, H-3'), 1.38-1.33 (m, 4H, H-4', H-5'), 0.93-0.88 (m, 3H, H-6'). ^{13}C NMR (75 MHZ, CDCl3) (75 MHz, $CDCl_3$): δ 193.29 (C-1), 181.13 (C-3), 161.63 (C-1'), 113.28 (C-2), 73.13 (C-4), 31.49 (C-4'), 30.53 (C-2'), 27.08 (C-3'), 25.44 (C-5), 22.38 (C-5'), 21.98 (C-6), 14.05 (C-6'). EIMS *m/z* (Rel. int. %): [M^+] 223 (2), 194 (21), 179 (48), 166 (16), 151(22), 154 (46), 148 (13), 139 (10), 137 (35), 122 (23), 109 (15), 94 (21), 81 (17), 69 (27), 57 (13), 43 (40), 41 (39), 39 (15). HRESIMS (*m/z*): calcd. for $C_{12}H_{17}NO_3$[M + H]+: 224.1287, found: 224.1277. Yield: 70%.

Compound 3d: Yellow solid. IR (KBr, ν_{max}, cm^{-1}): 3455, 2956, 1663, 1554, 1465, 1448, 1413, 1120. ^1H NMR (300 MHz, $CDCl_3$): δ 18.28 (s, 1H, OH, enol), 4.10 (dd, 13.0, 5.5,

2H, H-4, OH), 3.13-2.91 (m, 2H, H-6), 2.82-2.77 (m, 2H, H-2'), 2.43-2.35 (m, 1H, H-5), 1.91-1.57 (m, 3H, H-5, H-3'), 1.38-1.33 (m, 4H, H-4', H-5'), 0.93-0.89 (m, 3H, H-6'). ^{13}C NMR (75 MHZ, CDCl3) (75 MHz, $CDCl_3$): δ 206.23 (C-1'), 198.04 (C-3), 195.70 (C-1), 110.40 (C-2), 71.72 (C-4), 40.34 (C-2'), 31.60 (C-3'), 31.42 (C-6), 27.26 (C-5), 24.35 (C-4'), 22.57 (C-5'), 14.06 (C-6'). EIMS *m/z* (Rel. int. %): [M^+] 226 (11), 208 (17), 183 (100), 165 (23), 154 (35), 140 (88), 137 (53), 126 (70), 111 (21), 98 (54), 84 (48), 81 (14), 71 (69), 69 (41), 55 (29), 43 (67), 41 (30), 39 (12). HRESIMS (*m/z*): calcd. for $C_{12}H_{18}O_4$ [M + H]+: 227.1283, found: 227.1271. Yield: 95%.

Compound 3e: Pale yellow liquid. IR (KBr, ν_{max}, cm^{-1}): 2958, 1668, 1558, 1467, 1449, 1421, 1148. ^{1}H NMR (300 MHz, $CDCl_3$): δ 18.25 (s, 1H, OH, enol), 3.02 (t, 7.4, 2H, H-2'), 2.53 (s, 2H, H-6), 2.35 (s, 2H, H-4), 1.67-1.57 (m, 2H, H-3'), 1.38-1.33 (m, 4H, H-4', H-5'), 1.08 (s, 6H, H-1''), 0.92-0.87 (m, 3H, H-6'). ^{13}C NMR (75 MHZ, CDCl3) (75 MHz, $CDCl_3$): δ 205.86 (C-1'), 197.94 (C-3), 195.20 (C-1), 112.07 (C-2), 52.80 (C-5), 47.07 (C-6), 40.41 (C-2'), 31.68 (C-3'), 30.78 (C-4), 28.31 (C-4'), 24.55 (C-5'), 22.60 (C-1''), 14.08 (C-6). EIMS *m/z* (Rel. int. %): [M^+] 238 (18), 220 (9), 209 (9), 195 (100), 182 (38) 177 (12), 167 (48), 154 (8), 140 (22), 126 (29), 111 (21), 97 (16), 85 (5), 83 (51), 69 (27): 55 (30), 43 (36), 41 (20), 39 (10). HRESIMS (*m/z*): calcd. for $C_{14}H_{22}O_3$ [M + H]+: 239.1641, found: 239.1649. Yield: 64%.

Compound 4a: Orange liquid. IR (KBr, ν_{max}, cm^{-1}): 2954, 2927, 1668, 1558, 1462, 1072. ^{1}H NMR (300 MHz, $CDCl_3$): δ 18.26 (s, 1H, OH, enol), 3.01 (t, 7.6, 2H, H-2'), 2.66 (t, 7.6, 2H, H-4), 2.48 (t, 7.6, 2H, H-4), 1.97 (quint, 6.2, 2H, H-6), 1.66-1.56 (m, 2H, H-3'), 1.37-1.26 (m, 12H, H-4'-H-9'), 0.90-0.85 (m, 3H, H-10'). ^{13}C NMR (75 MHZ, CDCl3) (75 MHz, $CDCl_3$): δ 206.49 (C-1'), 198.79 (C-3), 195.45 (C-1), 113.16 (C-2), 40.73 (C-2'), 38.97 (C-

4), 33.47 (C-6), 32.02 (C-3'), 29.62 (CH_2), 29.58 (CH_2), 29.54 (CH_2), 29.42 (CH_2), 24.83 (CH_2), 22.81 (CH_2), 19.20 (CH_2), 14.24 (C-10'). EIMS *m/z* (Rel int. %): [M^+] 266 (7), 248 (6), 167 (100), 154 (51), 139 (60), 126 (23), 111 (17), 97 (9), 84 (8), 69 (28), 55 (36), 43 (23). HRESIMS (*m/z*): calcd. for $C_{16}H_{25}O_3$ [M + H]+: 267.1948, found: 267.1953. Yield: 58%.

Compound 4b: Colorless liquid. IR (film, ν_{max}, cm^{-1}): 2926, 1689, 1600, 1466, 1430, 1072. ^1H NMR (300 MHz, $CDCl_3$): δ 2.98 (t, 6.3, 2H, H-2'), 2.67 (t, 7.7, 2H, H-4), 2.52 (t, 6.5, 2H, H-6), 1.98 (quint, 6.5, 2H, H-5), 1.91 (m, 2H, H-3'), 1.39-1.26 (m, 12H, H-4'–H-9'), 0.90-0.85 (m, 3H, H-10'). ^{13}C NMR (75 MHZ, CDCl3) (75 MHz, $CDCl_3$): δ 193.19 (C-1), 181.13 (C-3), 161.48 (C-1'), 114.81 (C-2), 38.18 (C-4), 31.99 (C-6), 29.58 (C-2') 29.40-29.38 (CH_2), 27.38 (CH_2), 25.64 (CH_2), 23.27 (CH_2), 22.80 (CH_2), 22.49 (CH_2), 14.25 (C-10'). EIMS *m/z* (Rel. int.%): [M^+] 263 (7), 248 (3), 234 (10), 220 (12), 206 (13), 192 (24), 178 (29), 164 (81), 151 (46), 138 (100), 123 (14), 55 (17), 43 (20). HRESIMS (*m/z*): calcd. for $C_{16}H_{25}NO_2$[M + H]+: 264.1964, found: 264.1958. Yield: 86%.

Compound 4c: Yellow solid. IR (KBr, ν_{max}, cm^{-1}): 3452, 2955, 1660, 1557, 1467, 1442, 1416, 1113. ^1H NMR (300 MHz, $CDCl_3$): δ 4.29 (dd, 12.7, 5.1, 1H, H-4), 3.70 (s, OH), 3.21-3.00 (m, 2H, H-6), 2.85 (t, 7.6, 2H, H-2'), 2.66-2.57 (m, 1H, H-5), 2.18-2.03 (m, 1H, H-5), 1.77-1.66 (m, 2H, H-3'), 1.38-1.33 (m, 12H, H-4'-H-9'), 0.93-0.88 (m, 3H, H-10'). ^{13}C NMR (75 MHZ, CDCl3) (75 MHz, $CDCl_3$): δ 193.29 (C-1), 181.15 (C-3), 161.63 (C-1'), 113.26 (C-2), 73.12 (C-4), 32.00 (CH_2), 30.51 (CH_2), 29.57 (CH_2), 29.41 (CH_2), 29.36 (CH_2), 29.35 (CH_2), 27.40 (CH_2), 25.49 (CH_2), 22.81 (CH_2), 21.98 (CH_2), 14.26 (C-10'). EIMS *m/z* (Rel. int.%): [M^+] 279 (6), 261 (6), 250 (11), 222 (13), 208 (11), 194 (18), 180 (61), 167 (49), 154 (58), 136 (100), 122 (16), 110 (13), 108 (17), 97 (17), 84 (15), 80

(16), 69 (32), 57 (16) 55 (25), 43 (33), 41 (42). HRESIMS (*m/z*): calcd. for $C_{16}H_{25}NO_3$[M + Na]+: 302.1732, found: 302.1726. Yield: 76%.

Compound 4d: Yellow solid. ^{1}H NMR (300 MHz, $CDCl_3$): δ 18.28 (s, 1H, OH, enol), 4.09 (dd, 13.0, 5.4, 2H, H-4, OH), 3.13-2.73 (m, 4H, H-6), 2.43-2.34 (m, 1H, H-5), 1.90-1.52 (m, 3H, H-5, H-3'), 1.38-1.27 (m, 12H, H-4', H-9'), 0.90-0.86 (m, 3H, H-10'). ^{13}C NMR (75 MHZ, CDCl3) (75 MHz, $CDCl_3$): δ 206.25 (C-1'), 198.05 (C-3), 195.70 (C-1), 110.41 (C-2), 71.73 (C-4), 40.41 (C-2'), 32.01 (C-3'), 31.43 (C-6), 29.58 (CH_2), 29.54 (CH_2), 29.47 (CH_2), 29.40 (CH_2), 27.28 (CH_2), 24.67 (CH_2), 22.80 (CH_2), 14.25 (C-10'). EIMS *m/z* (Rel. int.%): [M^+] 282 (3), 238 (2), 183 (15), 165 (5), 153 (5), 140 (11), 126 (8), 111 (4), 98 (7), 85 (12), 71 (13), 69 (24), 57 (29), 55 (45), 43 (100), 41 (100). HRESIMS (*m/z*): calcd. for $C_{16}H_{26}O_4$ [M + Na]+: 305.1729, found: 305.1725. Yield: 93%.

Compound 4e: Orange liquid. IR (KBr, ν_{max}, cm^{-1}): 2957, 2923, 1669, 1559, 1449, 1389, 1149. ^{1}H NMR (300 MHz, $CDCl_3$): δ 18.28 (s, 1H, OH, enol), 3.02 (t, 7.8, 2H, H-2'), 2.54 (s, 2H, H-4), 2.36 (s, 2H, H-6), 1.66-1.56 (m, 2H, H-3'), 1.38-1.27 (m, 12H, H-4'-H-9'), 1.08 (s, 6H, H-1''), 0.90-0.88 (m, 3H, H-10'). ^{13}C NMR (75 MHZ, CDCl3) (75 MHz, $CDCl_3$): δ 205.82 (C-1'), 197.89 (C-3), 195.15 (C-1), 112.00 (C-2), 52.74 (C-5), 46.99 (C-6), 40.44 (C-2'), 31.97 (C-3'), 30.73 (C-4), 29.57 (CH_2), 29.53 (CH_2), 29.49 (CH_2), 29.38 (CH_2), 28.27 (CH_2), 24.81 (CH_2), 22.77 (C-1''), 14.22 (C-10'). EIMS *m/z* (Rel int. %): [M^+] 294 (6), 279 (6), 209 (5), 195 (100), 182 (48), 167 (36), 154 (9), 140 (13), 126 (31), 111 (12), 97 (11), 83 (37), 69 (19), 55 (27), 43 (26). HRESIMS (*m/z*): calcd. for $C_{18}H_{30}O_3$[M + Na]+: 317.2087, found: 317.2086. Yield: 66%.

Compound 5a: Pale yellow liquid. IR (KBr, ν_{max}, cm^{-1}): 2925, 2854, 1668, 1564, 1558, 1462, 1073. ^{1}H NMR (300 MHz, $CDCl_3$): δ 18.27 (s, 1H, OH, enol), 3.01 (t, 7.6, 2H, H-2'),

2.66 (t, 6.6, 2H, H-4), 2.49 (t, 6.6, 2H, H-6), 1.97 (quint, 6.6, 2H, H-5), 1.61 (quint, 7.5, 2H, H-3'), 1.38-1.26 (m, 16H, H-4'-H-11'), 0.90-0.86 (m, 3H, H-12'). ^{13}C NMR (75 MHZ, CDCl3) (75 MHz, $CDCl_3$): δ 206.31 (C-1'), 198.60 (C-3), 195.25 (C-1), 112.97 (C-2), 40.56 (C-2'), 38.80 (C-4), 33.29 (C-6), 31.88 (C-3'), 29.59 (CH_2), 29.49 (CH_2), 29.42 (CH_2), 29.37 (CH_2), 29.31 (CH_2), 24.65 (CH_2), 22.66 (CH_2), 19.04 (CH_2), 14.08 (C-12'). EIMS *m/z* (Rel int. %): [M^+] 294 (6), 276 (7), 205 (4), 191 (4), 167 (100), 154 (56), 139 (54), 111 (15), 97 (8), 69 (24), 55 (30), 43 (20), 41(16). HRESIMS (*m/z*): calcd. for $C_{18}H_{30}O_3$ [M + H]+: 295.2267, found: 295.2269. Yield: 67%.

Compound 5b: Colorless liquid. IR (film, ν_{max}, cm^{-1}): 2926, 1689, 1601, 1466, 1430, 1072. ^1H NMR (300 MHz, $CDCl_3$): δ 2.97 (t, 6.3, 2H, H-2'), 2.85 (t, 7.7, 2H, H-4), 2.51 (t, 6.5, 2H, H-6), 2.21 (quint, 6.5, 2H, H-5), 1.75-1.65 (m, 2H, H-3'), 1.39-1.25 (m, 16H, H-4'–H-11'), 0.90-0.85 (m, 3H, H-10'). ^{13}C NMR (75 MHZ, CDCl3) (75 MHz, $CDCl_3$): δ 193.18 (C-1), 181.13 (C-3), 161.52 (C-1'), 114.84 (C-2), 38.06 (C-4), 32.06 (C-6), 29.76 (CH_2), 29.65 (CH_2), 29.49 (CH_2), 29.42 (CH_2), 29.40 (CH_2), 27.42 (CH_2), 25.67 (CH_2), 23.30 (CH_2), 22.83 (CH_2), 22.52 (CH_2), 14.26 (C-12'). EIMS *m/z* (Rel. int.%): [M^+] 291 (2), 220 (3), 178 (6), 164 (44), 138 (23), 122(4), 80 (6), 55 (34), 43 (83), 41 (100). HRESIMS (*m/z*): calcd. for $C_{18}H_{29}NO_2$[M + H]+: 292.2277, found: 292.2277. Yield: 80%.

Compound 5c: Yellow solid. IR (KBr, ν_{max}, cm^{-1}): 3435, 3382, 2919, 1676, 1594, 1470, 1413, 1364, 1121. ^1H NMR (300 MHz, $CDCl_3$): δ 4.29 (dd, 12.7, 5.1, 1H, H-4), 3.76 (s, OH), 3.21-2.99 (m, 2H, H-6), 2.85 (t, 7.6, 2H, H-2'), 2.66-2.56 (m, 1H, H-5), 2.18-2.04 (m, 1H, H-5), 1.77-1.66 (m, 2H, H-3'), 1.33-1.26 (m, 16H, H-4'-H-11'), 0.90-0.85 (m, 3H, H-10'). ^{13}C NMR (75 MHZ, CDCl3) (75 MHz, $CDCl_3$): δ 193.28 (C-1), 181.12 (C-3), 161.60 (C-1'), 113.25 (C-2), 73.10 (C-4), 32.02 (CH_2), 30.49 (CH_2), 29.73 (CH_2), 29.60 (CH_2), 29.46

(CH_2), 29.33 (CH_2), 27.37 (CH_2), 25.47 (CH_2), 22.81 (CH_2), 21.96 (CH_2), 14.25 (C-12'). EIMS *m/z* (Rel. int.%): [M^+] 307 (1), 292 (1), 261 (1), 190 (2), 180 (9), 167 (4), 162 (5), 154 (6), 136 (9), 124 (4), 110 (3), 97 (5), 80 (5), 69 (14), 67 (8), 57 (29), 55 (27), 43 (100), 41 (94). HRESIMS (*m/z*): calcd. for $C_{18}H_{29}NO_3$[M + H]+: 308.2226, found: 308.2219. Yield: 76%.

Compound 5d: Yellow solid. IR (KBr, ν_{max}, cm^{-1}): 3443, 2955, 2920, 1648, 1536, 1471, 1454, 1413, 1112. ^{1}H NMR (300 MHz, $CDCl_3$): δ 18.28 (s, 1H, OH, enol), 4.09 (dd, 13.0, 5.4, 2H, H-4), 3.13-2.73 (m, 4H, H-4), 2.43-2.34 (m, 1H, H-5), 1.90-1.52 (m, 3H, H-5, H-3'), 1.38-1.27 (m, 12H, H-4' - H-9'), 0.90-0.86 (m, 3H, H-12'). ^{13}C NMR (75 MHZ, CDCl3) (75 MHz, $CDCl_3$): δ 206.27 (C-1'), 198.07 (C-3), 195.71 (C-1), 110.41 (C-2), 71.74 (C-4), 40.42 (C-2'), 32.05 (C-3'), 31.45 (C-6), 29.76 (CH_2), 29.63 (CH_2), 29.55 (CH_2), 29.48 (CH_2), 27.29 (CH_2), 24.69 (CH_2), 22.83 (CH_2), 14.28 (C-12'). EIMS *m/z* (Rel. int.%): [M^+] 310 (3), 266 (1), 183 (12), 165 (4), 140 (8), 126 (7), 109 (6), 98 (6), 85 (12), 69 (24), 57 (30), 55 (40), 43 (100), 41 (84). HRESIMS (*m/z*): calcd. for $C_{18}H_{30}O_4$ [M + Na]+: 333.2042, found: 333.2036. Yield: 90%.

Compound 5e: Pale yellow liquid. IR (KBr, ν_{max}, cm^{-1}): 2957, 2927, 1669, 1558, 1467, 1450, 1042. ^{1}H NMR (300 MHz, $CDCl_3$): δ 18.26 (s, 1H, OH, enol), 3.02 (t, 7.4, 2H, H-2'), 2.53 (s, 2H, H-4), 2.35 (s, 2H, H-6), 1.69-1.52 (m, 2H, H-3'), 1.41-1.19 (m, 16H, H-4'-H-11'), 1.08 (s, 6H, H-1''), 0.92-0.82 (m, 3H, H-12'). ^{13}C NMR (75 MHZ, CDCl3) (75 MHz, $CDCl_3$): δ 205.89 (C-1'), 197.97 (C-3), 195.26 (C-1), 112.05 (C-2), 52.79 (C-5), 47.05 (C-4), 40.49 (C-2'), 32.05 (C-3'), 30.79 (C-6), 29.76 (CH_2), 29.66 (CH_2), 29.58 (CH_2), 28.54 (CH_2), 28.48 (CH_2), 28.32 (CH_2), 24.85 (CH_2), 22.83 (C-1''), 14.27 (C-12'). EIMS *m/z* (Rel int. %): [M^+] 322 (9), 307 (15), 289 (9), 195 (100), 182 (66), 167 (38), 154 (11), 140 (17),

126 (32), 111 (11), 97 (11), 83 (35), 69 (17), 55 (12), 43 (15), 41 (11. HRESIMS (*m/z*): calcd. for $C_{20}H_{34}O_3$[M + Na]+: 345.2406, found: 345.2401. Yield: 80%.

Compound 5f: Colorless liquid. IR (film, ν_{max}, cm^{-1}): 2958, 2926, 1690, 1602, 1466, 1411, 1159. ^1H NMR (300 MHz, $CDCl_3$): δ 2.85 (t, 7.6, H-2'), 2.83 (s, 3H, H-6), 2.39 (s, 2H, H-4), 1.75-1.66 (m, 2H, H-3'), 1.25-1.15 (m, 16H, H-4'-H-11'), 1.15 (s, 6H, H-1''), 0.90-0.85 (m, 3H, H-12''). ^{13}C NMR (75 MHZ, CDCl3) (75 MHz, $CDCl_3$): δ 192.56 (C-1), 180.66 (C-3), 161.41 (C-1'), 113.74 (C-2), 52.62 (C-5), 36.96 (C-6), 35.69 (C-4), 32.04 (CH_2), 29.75 (CH_2), 29.63 (CH_2), 29.48 (CH_2), 29.38 (CH_2), 28.52 (CH_2), 27.40 (CH_2), 25.63 (CH_2), 22.83 (C-1''), 14.27 (C-12'). EIMS *m/z* (Rel. int.%): [M^+] 319 (2), 248 (2), 220 (5), 192 (45), 136 (14), 110 (4), 83 (9), 55 (38), 43 (93), 41 (100). HRESIMS (*m/z*): calcd. for $C_{20}H_{33}NO_2$[M + H]+: 320.2590, found: 320.2585. Yield: 76%.

Compound 5g: Yellow solid. IR (KBr, ν_{max}, cm^{-1}): 3470, 2952, 2921, 1674, 1598, 1479, 1470, 1391, 1364, 1138. ^1H NMR (300 MHz, $CDCl_3$): δ 4.08 (d, 2.1, 1H, H-4), 3.70 (d, 2.1, 1H, OH), 2.93 (s, 2H, H-6), 2.85 (dd, 7.7, 2.2, H-2'), 2.53 (s, 2H, H-4), 1.77-1.67 (m, 2H, H-3'), 1.35 (s, 2H, H-1''), 1.41-1.19 (m, 16H, H-4'-H-11'), 0.90 (s, 3H, H-1''), 0.90-0.86 (m, 3H, H-12'). ^{13}C NMR (75 MHZ, CDCl3) (75 MHz, $CDCl_3$): δ 192.74 (C-1), 180.06 (C-3), 161.52 (C-1'), 112.41 (C-2), 80.62 (C-4), 41.24 (C-5), 36.67 (C-6), 32.05 (CH_2), 29.75 (CH_2), 29.62 (CH_2), 29.48 (CH_2), 29.36 (CH_2), 29.34 (CH_2), 27.75 (CH_2), 27.42 (CH_2), 25.49 (CH_2), 22.83 (CH_2), 18.97 (C-1''), 14.26 (C-12'). EIMS *m/z* (Rel. int.%): [M^+] 335 (2), 302 (4), 208 (14), 190 (6), 164 (6), 72 (20), 57 (34), 55 (30), 43 (100), 41 (88). HRESIMS (*m/z*): calcd. for $C_{20}H_{33}NO_3$[M + H]+: 336.2539, found: 336.2534. Yield: 82%.

Compound 6a: White solid. IR (KBr, ν_{max}, cm^{-1}): 3432, 2954, 2920, 1652, 1560, 1462, 1441, 1077. ^1H NMR (300 MHz, $CDCl_3$): δ 18.27 (s, 1H, OH, enol), 3.01 (t, 7.6, 2H, H-2'),

2.66 (t, 6.6, 2H, H-4), 2.48 (t, 6.6, 2H, H-6), 1.97 (quint, 6.6, 2H, H-5), 1.66-1.56 (2H, H-3'), 1.38-1.26 (m, 26H, H-4'-H-16'), 0.90-0.86 (m, 3H, H-17'). ^{13}C NMR (75 MHZ, CDCl3) (75 MHz, $CDCl_3$): δ 206.31 (C-1'), 198.60 (C-3), 195.24 (C-1), 112.98 (C-2), 40.56 (C-2'), 38.80 (C-4), 33.30 (C-6), 31.91 (C-6), 29.67 (CH_2), 29.65 (CH_2), 29.61 (CH_2), 29.51 (CH_2), 29.38 (CH_2), 29.34 (CH_2), 24.66 (CH_2), 22.67 (CH_2), 19.04 (CH_2), 14.09 (C-17'). EIMS *m/z* (Rel int. %): [M^+] 364 (4), 346 (8), 205 (4), 191 (5), 167 (100), 154 (72), 139 (45), 126 (19), 111 (13), 97 (9), 69 (21), 57 (12), 55 (29), 43 (27), 41 (16). HRESIMS (*m/z*): calcd. for $C_{23}H_{40}O_3$ [M + H]+: 365.3050, found: 365.3045. Yield: 69%.

Compound 6b: White solid. IR (KBr, ν_{max}, cm^{-1}): 3435, 2919, 2852, 1661, 1567, 1471, 1433, 1148. ^{1}H NMR (300 MHz, $CDCl_3$): δ 18.26 (s, 1H, OH, enol), 3.02 (t, 7.8, 2H, H-2'), 2.53 (s, 2H, H-6), 2.35 (s, 2H, H-4), 1.66-1.56 (m, 2H, H-3'), 1.44-1.25 (m, 26H, H-4'-H-16'), 1.07 (s, 6H, H-1''), 0.93-0.83 (m, 3H, H-17'). ^{13}C NMR (75 MHZ, CDCl3) (75 MHz, $CDCl_3$): δ 205.85 (C-1'), 197.91 (C-3), 195.15 (C-1), 112.06 (C-2), 52.81 (C-5), 47.07 (C-6), 40.46 (C-2'), 32.06 (C-3'), 30.77 (C-4), 29.83 (CH_2), 29.80 (CH_2), 29.76 (CH_2), 29.66 (CH_2), 29.57 (CH_2), 29.54 (CH_2), 29.50 (CH_2), 28.31 (CH_2), 24.87 (CH_2), 22.83 (C-1''), 14.26 (C-17'). EIMS *m/z* (Rel int. %): [M^+] 392 (5), 359 (10), 209 (5), 195 (100), 182 (66), 167 (31), 154 (11), 140 (10), 126 (25), 111 (10), 97 (13), 83 (30), 69 (16), 55 (22), 43 (28), 41 (14). HRESIMS (*m/z*): calcd. for $C_{25}H_{44}O_3$ [M + Na]+: 415.3182, found: 415.3185. Yield: 71%.

Compound 7a: Yellow liquid. IR (KBr, ν_{max}, cm^{-1}): 3613, 1715, 1665, 1561, 1501, 1459, 1190. ^{1}H NMR (300 MHz, $CDCl_3$): δ 18.08 (s, 1H, OH, enol), 3.04 (t, 7.5, 2H, H-4), 2.67 (t, 6.5, 2H, H-2'), 2.52(t, 7.3, 2H, H-4'), 2.48 (t, 6.5, 2H, H-6), 2.15 (s, 3H, H-6'), 1.98 (quint, 6.5, 2H, H-3'), 1.91 (quint, 7.5, 2H, H-5). ^{13}C NMR (75 MHZ, CDCl3) (75 MHz, $CDCl_3$): δ

208.36 (C-5'), 205.62 (C-1'), 198.28 (C-3), 195.41 (C-1), 113.21 (C-2), 42.98 (C-4'), 40.00 (C-2’), 38.86 (C-4), 33.16 (C-6), 30.02 (C-3), 19.19 (C-6'), 18.59 (C-5). EIMS *m/z* (Rel int. %): [M^+] 224 (0), 206 (8), 167 (10), 139 (19), 126 (5), 111(14), 97 (5), 85 (7), 69 (32), 55 (36), 43 (100), 41 (24). HRESIMS (*m/z*): calcd. for $C_{12}H_{16}O_4$ [M + Na]+: 247.0940, found: 247.0949. Yield: 66%.

Compound 7b: Pale yellow liquid. IR (KBr, ν_{max}, cm^{-1}): 3648, 2959, 1715, 1666, 1566, 1556, 1442, 1151. ^1H NMR (300 MHz, $CDCl_3$): δ 18.09 (s, 1H, OH, enol), 3.04 (t, 7.3, 2H, H-2'), 2.56-2.51 (m, 4H, H-6, H-4'), 2.35(s, 2H, H-4), 2.15 (s, 3H, H-6'), 1.95-1.85 (m, 2H, H-3'), 1.08 (s, 6H, H-1''). ^{13}C NMR (75 MHZ, CDCl3) (75 MHz, $CDCl_3$): δ 208.26 (C-5'), 204.87 (C-2'), 197.31 (C-3), 195.11 (C-1), 111.94 (C-2), 52.50 (C-5), 46.54 (C-6), 42.79 (C-4'), 39.63 (C-2'), 30.68 (C-4), 29.90 (C-3'), 28.14 (C-6'), 18.40 (C-1''. EIMS *m/z* (Rel int. %): [M^+] 252 (0), 234 (7), 195 (9), 167 (10), 126 (6), 111 (8), 83 (19), 69 (21), 55 (28), 43 (100), 41 (27). HRESIMS (*m/z*): calcd. for $C_{14}H_{20}O_4$ [M + Na]+: 275.1253, found: 275.1259. Yield: 54%.

Compound 8a: Yellow solid. IR (KBr, ν_{max}, cm^{-1}): 3436, 2944, 1658, 1625, 1575, 1524, 1494, 1448, 1189, 979. ^1H NMR (300 MHz, $CDCl_3$): δ 18.53 (s, 1H), 8.27 (d, 15.9, H-3'), 7.94 (d, 15.9, H-2'), 7.67-7.64 (m, 2H, H-5', H-9'), 7.41-7.39 (m, 3H, H-6'-H-8'), 2.70 (t, 6.6, 2H, H-3'), 2.56 (t, 6.0, 2H, H-6), 2.01 (quint, 6.0, 2H, H-5). ^{13}C NMR (75 MHZ, CDCl3) (75 MHz, $CDCl_3$): δ 202.39 (C-1), 196.27 (C-3), 188.61 (C-1'), 146.04 (C-3'), 135.11 (C-4'), 131.01 (C-6', C-8'), 129.15 (C-5', H-9'), 129.04 (C-7'), 123.11 (C-2'), 112.41 (C-2), 39.44 (C-4), 35.12 (C-6), 19.04 (C-5). EIMS *m/z* (Rel int. %): [M^+] 242 (52), 241 (54), 214 (12), 186 (20), 171 (38), 165 (100), 131 (69), 115 (32), 103 (71), 91 (10), 77 (47), 69 (19), 55

(17), 51 (17), 42 (11), 39 (8). HRESIMS (*m/z*): calcd. for $C_{15}H_{14}O_3$ [M + H]+: 243.1015, found: 243.1015. Yield: 66%.

Compound 8b: Colorless liquid. IR (KBr, $\sqrt{}_{max}$, cm^{-1}): 3062, 2952, 1665, 1560, 1497, 1439, 1190, 964. ^{1}H NMR (300 MHz, $CDCl_3$): δ 18.09 (s, 1H: OH, enol), 7.31-7.18 (m, 5H, H-5'- H-9'), 3.36 (t, 7.8, 2H, H-2'), 2.94 (t, 7.8, 2H, H-4), 2.66 (t, 6.6, 2H, H-3'), 2.48 (t, 6.0, 2H, H-6), 1.96 (quint, 6.0, 2H, H-5). ^{13}C NMR (75 MHZ, CDCl3) (75 MHz, $CDCl_3$): δ 205.17 (C-1'), 198.36 (C-3), 195.39 (C-1), 141.10 (C-4'), 128.67 (C-6', C-8'), 128.50 (C-5', C-9'), 126.17 (C-7'), 113.26 (C-2), 42.52 (C-2'), 38.85 (C-4), 33.19 (C-6), 30.60 (C-3'), 19.17 (C-5). EIMS *m/z* (Rel int. %): [M^+] 244 (52), 226 (15), 216 (15), 198 (5), 170 (10), 153 (15), 139 (78), 112 (100), 104 (20), 91 (78), 84 (17), 77 (16), 69 (36), 55 (30), 39 (10). HRESIMS (*m/z*): calcd. for $C_{15}H_{16}O_3$ [M + H]+: 245.1166, found: 245.1170. Yield: 43%.

Compound 8c: Yellow solid. IR (KBr, $\sqrt{}_{max}$, cm^{-1}): 3433, 2947, 1659, 1623, 1576, 1517, 1497, 1460, 1118, 964. ^{1}H NMR (300 MHz, $CDCl_3$): δ 18.60 (s, 1H, OH, enol), 8.26 (d, 15.9, 1H, H-3'), 8.02 (d, 15.9, 1H, H-2'), 7.70-7.67 (m, 2H, H-5', H-9'), 7.46-7.42 (m, 3H, H-6'-H-8'), 4.18 (dd, 13.0, 1.7, 1H, H-4), 4.12 (d, 1.7 Hz, OH), 2.86-2.81 (m, 2H, H-6), 2.46-2.38 (m, 2H, H-4), 1.94-1.80 (m, 1H, H-4). ^{13}C NMR (75 MHZ, CDCl3) (75 MHz, $CDCl_3$): δ 201.57 (C-1), 196.40 (C-3), 188.51 (C-1'), 147.24 (C-3'), 134.76 (C-4'), 131.45 (C-5', C-9'), 129.29 (C-6', C-8'), 129.16 (C-7'), 121.89 (C-2'), 109.60 (C-2), 72.19 (C-4), 33.08 (C-5), 27.13 (C-6). EIMS *m/z* (Rel int. %): [M^+] 258 (14), 213 (8), 186 (18), 158 (7), 131 (64), 115 (72), 103 (89), 91 (21), 77 (100), 69 (40), 63 (20), 57 (52), 55 (38), 51 (57), 43 (53), 39 (47). HRESIMS (*m/z*): calcd. for $C_{15}H_{14}O_4$ [M + Na]+: 281.0790, found: 281.0789. Yield: 60%.

Compound 8d: Yellow solid. IR (KBr, ν_{max}, cm^{-1}): 3435, 2956, 1644, 1620, 1575, 1528, 1470, 1450, 1176, 960. ^1H NMR (300 MHz, $CDCl_3$): δ 18.51 (d, 0.9 Hz, 1H, OH, enol), 8.31 (dd, 15.9, 0.9, 1H, H-3'), 7.95 (d, 15.9, 1H, H-2'), 7.67-7.64 (m, 2H, H-5', H-9'), 7.57-7.38 (m, 2H, H-6'-H-8'), 2.57 (s, 2H, H-6), 2.43 (s, 2H, H-4), 1.09 (s, 6H, H-1''). ^{13}C NMR (75 MHZ, CDCl3) (75 MHz, $CDCl_3$): δ 201.81 (C-1), 195.98 (C-3), 187.79 (C-1'), 146.05 (C-2'), 135.07 (C-4'), 130.97 (C-6', C-8'), 129.10 (C-5', C-9'), 129.00 (C-7'), 122.89 (C-2'), 111.17 (C-2), 53.24 (C-5), 48.73 (C-6), 30.56 (C-4), 28.32 (C-1''). EIMS *m/z* (Rel int. %): [M^+] 270 (41), 269 (50), 252 (6), 237 (7), 213 (5), 193 (100), 186 (18), 171 (24), 131 (61), 115 (25), 103 (59), 91 (9), 77 (37), 69 (12), 55 (14), 43 (8), 41 (10). HRESIMS (*m/z*): calcd. for $C_{17}H_{18}O_3$ [M + H]+: 271.1328, found: 271.1331. Yield: 70%.

Compound 8e: Yellow solid. IR (KBr, ν_{max}, cm^{-1}): 3312, 3025, 2955, 1667, 1561, 1490, 1434, 1144, 946. ^1H NMR (300 MHz, $CDCl_3$): δ 18.09 (s, 1H: OH, enol), 7.28-7.18 (m, 5H, H-5'-H9'), 3.37 (t, 7.8, 2H, H-2'), 2.94 (t, 7.8, 2H, H-3'), 2.53 (s, 2H, H-6), 2.35 (s, 2H, H-4), 1.07(s, 6H, H-1''). ^{13}C NMR (75 MHZ, CDCl3) (75 MHz, $CDCl_3$): δ 204.60 (C-1'), 197.55 (C-3), 195.20 (C-1), 141.07 (C-4'), 128.68 (C-6', C-8'), 128.52 (C-5', C-9'), 126.19 (C-7'), 112.20 (C-2), 52.72 (C-4), 46.83 (C-6), 42.27 (C-2'), 30.83 (C-4), 30.67 (C-3'), 28.32 (C-1'). EIMS *m/z* (Rel int. %): [M^+] 272 (55), 254 (14), 216 (7), 198 (12), 181 (14), 167 (85), 140 (100), 125 (11), 111 (16), 105 (26) 91 (83), 83 (55), 77 (14), 69 (26), 55 (22), 43 (18), 41 (13). HRESIMS (*m/z*): calcd. for $C_{17}H_{20}O_3$ [M + Na]+: 295.1304, found: 295.1306. Yield: 60%.

Compound 9a: Yellow solid. IR (KBr, ν_{max}, cm^{-1}): 3435, 2944, 1658, 1623, 1599, 1582, 1525, 1439, 1416, 1170, 982. ^1H NMR (300 MHz, $CDCl_3$): δ 18.53 (d, 0.9, 1H: OH, enol),

8.27 (dd, 15.8, 0.9, 1H, H-3'), 7.95 (d, 15.8, 1H, H-2'), 7.49-7.44 (m, 2H, H-8', H-9'), 7.32-7.21 (m, 2H, H-6', H-7'), 2.70 (t, 6.5, 2H, H-4), 2.57 (t, 6.5, 2H, H-6), 2.39 (s, 3H, CH_3), 2.06-2.00 (m, 2H, H-5). ^{13}C NMR (75 MHZ, CDCl3) (75 MHz, $CDCl_3$): δ 202.29 (C-1), 196.16 (C-3), 185.56 (C-1'), 143.30 (C-3'), 138.54 (C-4'), 133.77 (C-5'), 130.88 (C-7'), 130.67 (C-6'), 127.39 (C-9'), 126.44 (C-8'), 123.77 (C-2'), 112.29 (C-2), 39.29 (C-4), 34.98 (C-6), 19.89 (C-5), 18.92 (CH_3). EIMS *m/z* (Rel int. %): [M^+] 256 (11), 255 (10), 238 (100), 210 (12), 182 (26), 165 (39), 145 (26), 139 (33), 128 (15), 115 (78), 105 (14), 91 (29), 69 (30), 55 (25), 43 (7), 39 (11). HRESIMS (*m/z*): calcd. for $C_{16}H_{16}O_3$[M + H]+: 257.1172, found: 257.1176. Yield: 56%.

Compound 9b: White solid. IR (KBr, ν_{max}, cm^{-1}): 3017, 1722, 1687, 1666, 1492, 1459, 1436, 1050, 959. ^1H NMR (500 MHz, $CDCl_3$): δ 18.13 (s, 1H, OH, enol), 7.21-7.19 (m, 2H, H-8', H-9'), 7.15-7.09 (m, 2H, H-6', H-7'), 3.31 (t, 7.5, 2H, H-2'), 2.93 (t, 7.5, 2H, H-3'), 2.67 (t, 6.5, 2H, H-4), 2.48 (t, 7.0, 2H, H-6), 2.35 (s, 3H), 1.98 (quint, 6.5, 2H, H-5). ^{13}C NMR (75 MHZ, CDCl3) (75 MHz, $CDCl_3$): δ 205.13 (C-1'), 198.35 (C-3), 195.26 (C-1), 139.07 (C-4'), 136.16 (C-5'), 130.22 (C-6'), 128.92 (C-9'), 126.27 (C-7'), 126.07 (C-8'), 113.21 (C-2), 41.06 (C-2'), 38.76 (C-4), 33.14 (C-6), 27.95 (C-3'), 19.33 (C-5), 19.08 (CH_3). EIMS *m/z* (Rel int. %): [M^+] 258 (1), 240 (86), 239 (56), 221 (6), 194 (4), 165 (4), 153 (9), 139 (37), 112 (57), 105 (100), 91 (15), 84 (11), 79 (20), 77 (21), 69 (25), 55 (24), 43 (6), 39 (8). HRESIMS (*m/z*): calcd. for $C_{16}H_{18}O_3$[M + Na]+: 281.1148, found: 281.1151. Yield: 57%.

Compound 9c: Yellow solid. IR (KBr, ν_{max}, cm^{-1}): 3435, 3102, 2949, 1560, 1572, 1512, 1460, 1419, 1037, 983. ^1H NMR (300 MHz, $CDCl_3$): δ 18.52 (s, 0.9 Hz, 1H: OH, enol), 8.28 (d, 15.8, 1H, H-3'), 8.21 (d, 15.8, 1H, H-2'), 7.78-7.75 (m, 1H, H-9'), 7.32-7.20 (m,

2H, H-6'-H-8'), 2.58 (s, 2H, H-4): 2.48 (s, 3H, CH_3), 2.39 (s, 2H, H-6), 1.11 (s, 6H, H-1'). ^{13}C NMR (75 MHZ, CDCl3) (75 MHz, $CDCl_3$): δ 201.85 (C-1), 196.08 (C-3), 187.99 (C-1'), 143.57 (C-3'), 138.67 (C-4'), 133.96 (C-5'), 131.01 (C-7'), 130.81 (C-6'), 127.57 (C-9'), 126.59 (C-8'), 123.77 (C-2'), 111.27 (C-2), 53.32 (C-5), 48.82 (C-6), 30.67 (C-4), 28.40 (C-1''), 20.02 (CH_3). EIMS *m/z* (Rel int. %): [M^+] 284 (49), 283 (46), 269 (14), 251 (8), 200 (23), 193 (100), 185 (31), 145 (38), 129 (12), 115 (53), 105 (9), 91 (24), 83 (17), 77 (7), 69 (12), 55 (14), 43 (10), 39 (8). HRESIMS (*m/z*): calcd. for $C_{18}H_{20}O_3$[M + H]+: 285.1491, found: 285.1485. Yield: 66%.

Compound 9d: Yellow liquid. IR (KBr, ν_{max}, cm^{-1}): 3018, 2959, 1667, 1558, 1493, 1448, 1049, 950. ^1H NMR (300 MHz, $CDCl_3$): δ 18.12 (s, 0.9 Hz, 1H: OH, enol), 7.25-7.09 (m, 4H, H-6'-H-9'), 3.32 (t, 7.5, 2H, H-2'), 2.93 (t, 7.5, 2H, H-3'), 2.54 (s, 2H, H-4): 2.48 (s, 5H, CH_3, H-6), 1.07 (s, 6H, H-1'). ^{13}C NMR (75 MHZ, CDCl3) (75 MHz, $CDCl_3$): δ 204.64 (C-1'), 197.63 (C-3), 195.19 (C-1), 139.10 (C-4'), 136.26 (C-5'), 130.31 (C-6'), 129.01 (C-9'), 126.35 (C-7'), 126.15 (C-8'), 112.21 (C-2), 52.70 (C-5), 46.86 (C-6), 40.88 (C-2), 30.81 (C-4), 28.31 (C-1''), 28.06 (C-3'), 19.41 (CH_3). EIMS *m/z* (Rel int. %): [M^+] 286 (1), 268 (76), 267 (75), 249 (4), 212 (4), 184 (5), 167 (32), 153 (5), 140 (46), 111 (11), 105 (100), 91 (15), 83 (36), 79 (20), 77 (18), 69 (18), 55 (16), 43 (13), 41 (10). HRESIMS (*m/z*): calcd. for $C_{18}H_{22}O_3$ [M + Na]+: 309.1461, found: 309.1461. Yield: 61%.

Compound 10a: Yellow solid. IR (KBr, ν_{max}, cm^{-1}): 3436, 3108, 2946, 1667, 1649, 1621, 1578, 1528, 1494, 1462, 1263, 1043, 985. ^1H NMR (300 MHz, $CDCl_3$): δ 18.52 (d, 0.8, 1H, OH, enol), 8.24 (dd, 15.9, 0.8, 1H, H-3'), 7.89 (d, 15.9, 1H, H-2'), 7.14-7.33 (m, 3H, H-5'. H-8', H-9'), 7.57-7.50 (m, 1H, H-7'), 2.71 (t, 6.4, 2H, H-4), 2.56 (t, 6.4, 2H, H-6), 1.10 (t, 6.4, 2H, H-5). ^{13}C NMR (75 MHZ, CDCl3) (75 MHz, $CDCl_3$): δ 202.31(C-1), 196.13

(C-3), 188.41 (C-1'), 159.94 (C-6'), 145.83 (C-3'), 136.37 (C-4'), 129.90 (C-8'), 123.25 (C-2'), 121.86 (C-9'), 117.11 (C-7'), 113.41 (C-5'), 112.29 (C-2), 55.35 (OCH_3), 39.31 (C-4), 34.99 (C-6), 18.92 (C-5). EIMS *m/z* (Rel int. %): [M^+] 272 (76), 271 (34), 254 (25), 244 (19), 227 (17) 216 (44), 201 (100), 165 (85), 161 (46), 133 (30), 118 (36), 103 (22), 90 (23), 77 (29), 69 (22), 55 (25), 42 (13), 39 (10). HRESIMS (*m/z*): calcd. for $C_{16}H_{16}O_4$ [M + H]+: 273.1121, found: 273.1128. Yield: 60%.

Compound 10b: White solid. IR (KBr, $\sqrt{}_{max}$, cm^{-1}): 3448, 2943, 1659, 1598, 1543, 1489, 1472, 1449, 1252, 1039, 849. ^1H NMR (300 MHz, $CDCl_3$): δ 18.03 (s, 1H, OH, enol), 7.20 (t, 7.8, 1H, H-8'), 6.81 (m, 3H, H-5', H-7', H-9'), 3.80 (s, 3H, OCH_3), 3.36 (t, 8.0, 2H, H-2'), 2.92 (t, 8.0, 2H, H-3'), 2.67 (t, 6.6, 2H, H-4'), 2.49 (t, 7.2, H-6'), 1.97 (quint, 6.4, 2H, H-5'). ^{13}C NMR (75 MHZ, CDCl3) (75 MHz, $CDCl_3$): δ 204.99 (C-1'), 198.16 (C-1), 195.22 (C-3), 159.63 (C-6'), 142.61 (C-4'), 129.33 (C-8'), 120.90 (C-9'), 114.19 (C-5'), 113.13 (C-2), 111.49 (C-7'), 55.14 (OCH_3), 42.28 (C-2'), 38.71 (C-4), 33.03 (C-6), 30.44 (C-3'), 19.04 (C-5). EIMS *m/z* (Rel int. %): [M^+] 274 (54), 256 (49), 200 (10), 172 (7), 153 (10), 139 (36), 135 (100), 121 (66), 112 (82), 105 (24), 91 (41), 84 (14), 77 (24), 69 (36), 55 (34), 41 (8), 39 (9). HRESIMS (*m/z*): calcd. for $C_{16}H_{18}O_4$[M + H]+: 275.1278, found: 275.1274. Yield: 44%.

Compound 10c: Yellow solid. IR (KBr, $\sqrt{}_{max}$, cm^{-1}): 3097, 2963, 1648, 1601, 1576, 1525, 1436, 1415, 1259, 1048, 961. ^1H NMR (300 MHz, $CDCl_3$): δ 18.50 (s, 1H, OH, enol), 8.29 (d, 15.9, 1H, H-3'), 7.92 (d, 15.9, 1H, H-2'), 7.34-7.23 (m, 2H, H-8', H-9'), 7.17-7.16 (m, 1H, H-5'), 6.98-6.94 (m, 1H, H-7'), 3.85 (s, 2H, OCH_3), 2.56 (s, 2H, H-6), 2.43 (s, 2H, H-4), 1.10 (s, 6H, H-1''). ^{13}C NMR (75 MHZ, CDCl3) (75 MHz, $CDCl_3$): δ 201.87 (C-1), 196.06 (C-3), 187.78 (C-1'), 160.03 (C-6'), 146.06 (C-3'), 136.45 (C-4'), 129.99 (C-8'), 123.14 (C-2'),

122.00 (C-9'), 117.28 (C-7'), 113.43 (C-5'), 111.20 (C-2), 55.44 (OCH_3), 53.27 (C-5), 48.77 (C-6), 30.60 (C-4), 28.35 (C-1''). EIMS *m/z* (Rel int. %): [M^+] 300 (79), 299 (38), 282 (33), 267 (14), 255 (7), 216 (43), 201 (72), 193 (100), 161 (48), 133 (26), 118 (33), 115 (13), 103 (21), 90 (19), 83 (26), 77 (24), 69 (16), 55 (21), 43 (11), 41 (13). HRESIMS (*m/z*): calcd. for $C_{18}H_{20}O_4$ [M + H]+: 301.1434, found: 301.1433. Yield: 68%.

Compound 10d: Yellow liquid. IR (KBr, ν_{max}, cm^{-1}): 3315, 2958, 1667, 1584, 1574, 1557, 1489, 1436, 1261, 1045, 949. ^{1}H NMR (300 MHz, $CDCl_3$): δ 18.07 (s, 1H, OH, enol), 7.20 (t, 7.8, 1H), 6.81 (m, 3H), 3.79 (s, 3H), 3.36 (t, 7.2, 2H), 2.92 (t, 7.2, 2H), 2.53 (s, 2H), 2.35s, 2H), 1.07 (s, 6H). ^{13}C NMR (75 MHZ, CDCl3) (75 MHz, $CDCl_3$): δ 204.56 (C-1), 197.49 (C-3), 195.18 (C-1'), 159.78 (C-6'), 142.70 (C-4'), 129.48 (C-8'), 121.05 (C-9'), 114.30 (C-5'), 112.22 (C-2), 111.69 (C-7'), 55.29 (OCH_3), 52.72 (C-5), 46.80 (C-6), 42.16 (C-2'), 30.83 (C-4), 30.66 (C-3'), 28.32 (C-1''). EIMS *m/z* (Rel int. %): [M^+] 302 (58), 284 (58), 253 (7), 228 (7), 200 (12), 181 (10), 167 (39), 140 (88), 135 (100), 121 (72), 111 (16), 105 (23), 91 (42), 83 (57), 77 (23), 69 (25), 55 (23), 43 (17), 41 (13). HRESIMS (*m/z*): calcd. for $C_{18}H_{22}O_4$ [M + Na]+: 325.1410, found: 325.1413. Yield: 50%.

Compound 11a: Yellow solid. IR (KBr, ν_{max}, cm^{-1}): 3434, 3100, 2956, 1650, 1617, 1594, 1530, 1457, 1265, 1171, 1024, 982. ^{1}H NMR (300 MHz, $CDCl_3$): δ 18.57 (d, 0.9, 1H, OH, enol), 8.17 (dd, 15.9, 0.9, 1H, H-3'), 7.92 (d, 15.9, 1H, H-2'), 7.61 (d, 8.9, 2H, H-5', H-9'), 6.91 (d, 8.9, 2H, H-6', H-8'), 2.67 (t, 6.5, 2H, H-4), 2.54 (t, 6.4, 2H, H-6), 1.99 (quint, 6.5, 2H, H-5). ^{13}C NMR (75 MHZ, CDCl3) (75 MHz, $CDCl_3$): δ 202.42 (C-1), 196.28 (C-3), 188.24 (C-1'), 162.17 (C-7'), 146.16 (C-3'), 131.05 (C-5', C-9'), 127.88 (C-4'), 120.35 (C-2'), 114.50 (C-6', C-8'), 112.01 (C-2), 55.49 (OCH_3), 39.44 (C-4), 35.25 (C-6), 19.01 (C-5). EIMS *m/z* (Rel int. %): [M^+] 272 (100), 271 (75), 254 (10), 241 (9), 216 (20), 201 (53),

165 (37), 161 (57), 134 (44), 133 (47), 121 (52), 115 (13), 111 (6), 108 (25), 103 (21), 89 (18), 79 (12), 77 (30), 69 (18), 55 (21), 42 (10), 39 (8). HRESIMS (*m/z*): calcd. for $C_{16}H_{16}O_4$ [M + H]+: 273.1121, found: 273.1123. Yield: 66%.

Compound 11b: White solid. IR (KBr, ν_{max}, cm^{-1}): 3011, 2950, 1655, 1614, 1562, 1514, 1461, 1242, 1177, 1028, 958. ^1H NMR (300 MHz, $CDCl_3$): δ 18.13 (s, 1H, OH, enol), 7.18 (d, 8.6, H-5', H-9'), 6.82 (d, 8.6, H-6', H-8'), 3.78 (s, 3H, OCH_3), 3.32 (t, 8.0, 2H, H-2'), 2.88 (t, 8.0, 2H, H-3'), 2.66 (t, 6.8, 2H, H-4), 2.49 (t, 6.6, 2H, H-6), 1.97 (quint, 6.6, 2H, H-5). ^{13}C NMR (75 MHZ, CDCl3) (75 MHz, $CDCl_3$): δ 205.24 (C-1'), 198.45 (C-1), 195.36 (C-3), 158.05 (OCH_3), 133.16 (C-4'), 129.59 (C-5', C-9'), 113.91 (C-2, C-6', C-8'), 55.37 (OCH_3), 42.76 (C-2'), 38.87 (C-4), 33.24 (C-6), 29.80 (C-3'), 19.17 (C-5). EIMS *m/z* (Rel int. %): [M^+] 274 (10), 134 (3), 121 (100), 91 (5), 77 (6), 55 (5), 41 (1). HRESIMS (*m/z*): calcd. for $C_{16}H_{18}O_4$ [M + Na]+: 297.1097, found: 297.1103. Yield: 37%.

Compound 11c: Yellow solid. IR (KBr, ν_{max}, cm^{-1}): 3425, 3102, 2947, 1663, 1601, 1573, 1512, 1427, 1247, 1174, 1012, 963. ^1H NMR (300 MHz, $CDCl_3$): δ 18.54 (s, 1H, OH, enol), 8.13 (d, 15.9, 1H, H-3'), 7.98 (d, 15.9, 1H, H-2'), 7.63 (d, 8.9, 2H, H-5', H-9'), 6.93 (d, 8.9, 2H, H-6', H-8'), 4.17-4.05 (m, 2H, H-4, OH), 3.78 (s, 3H, OCH_3), 2.80-2.76 (m, 2H, H-6), 2.40-2.35 (m, 2H, H-5), 1.91-1.74 (m, 2H, H-5). ^{13}C NMR (75 MHZ, CDCl3) (75 MHz, $CDCl_3$): δ 201.63 (C-1), 196.36 (C-3), 188.06 (C-1'), 162.52 (C-7), 147.37 (C-3'), 131.30 (C-5′, C-9'), 127.54 (C-2', C-4'), 119.05 (C-2), 114.61 (C-6', C-8'), 72.13 (C-4), 55.54 (OCH_3), 33.22 (C-5), 27.10 (C-6). EIMS *m/z* (Rel int. %): [M^+] 288 (100), 270 (14), 244 (13), 216 (4), 201 (20), 161 (96), 147 (16), 133 (46), 121 (45), 115 (10), 89 (11), 77 (23), 69 (8), 43 (3). HRESIMS (*m/z*): calcd. for $C_{16}H_{16}O_5$[M + Na]+: 311.0895, found: 311.0889. Yield: 56%.

Compound 11d: Yellow solid. IR (KBr, ν_{max}, cm^{-1}): 3436, 3095, 2950, 1660, 1619, 1571, 1512, 1458, 1256, 1174, 1022, 985. ^1H NMR (300 MHz, $CDCl_3$): δ 18.53 (d, 0.9, 1H, OH, enol), 8.21 (dd, 15.9, 0.9, 1H, H-3'), 7.95 (d, 15.9, 1H, H-2'), 7.63 (d, 8.9, 2H, H-5', H-9'), 6.93 (d, 8.9, 2H, H-6', H-8'), 3.85 (s, 3H, OCH_3), 2.56 (s, 2H, H-6), 2.07 (s, 2H, H-4), 1.10 (s, 6H, H-1''). ^{13}C NMR (75 MHZ, CDCl3) (75 MHz, $CDCl_3$): δ 201.93 (C-1), 196.16 (C-3), 187.62 (C-1'), 162.26 (C-7'), 146.33 (C-3'), 131.13 (C-5', C-9'), 127.97 (C-4'), 120.24 (C-3'), 114.57 (C-2), 110.90 (C-6', C-8'), 55.57 (OCH_3), 55.38 (C-5), 49.00 (C-6), 30.60 (C-4), 28.39 (C-1''). EIMS *m/z* (Rel int. %): [M^+] 300 (100), 299 (92), 269 (9), 216 (33), 201 (49), 193 (49), 161 (72), 134 (49), 133 (48), 121 (49), 108 (28), 105 (5), 90 (15), 77 (29), 69 (16), 55 (21), 41 (13). HRESIMS (*m/z*): calcd. for $C_{18}H_{20}O_4$ [M + H]+: 301.1434, found: 301.1436. Yield: 62%

Compound 11e: Yellow solid. IR (KBr, ν_{max}, cm^{-1}): 3039, 1689, 1665, 1611, 1466, 1441, 1421, 1246, 1178, 1037, 923. ^1H NMR (300 MHz, $CDCl_3$): δ 18.11 (s, 1H, OH, enol), 7.18 (d, 8.5, 2H, H-5', H-9'), 6.82 (d, 8.5, 2H, H-6', H-8'), 3.78 (s, 3H, OCH_3), 3.33 (t, 7.5, 2H, H-2'), 2.88 (t, 7.5, 2H, H-3'), 2.53 (s, 2H, H-6), 2.35 (s, 2H, H-4), 1.07(s, 6H, H-1''). ^{13}C NMR (75 MHZ, CDCl3) (75 MHz, $CDCl_3$): δ 204.52 (C-1'), 197.51 (C-3), 195.04 (C-1), 157.92 (C-7'), 132.98 (C-4'), 129.45 (C-5', C-9'), 113.79 (C-6', C-8'), 112.05 (C-2), 55.23 (OCH_3), 52.58 (C-5), 46.72 (C-6), 42.35 (C-2'), 30.66 (C-3'), 29.74 (C-4), 28.16 (C-1''). EIMS *m/z* (Rel int. %): [M^+] 302(11), 123(9), 121(100), 108(6), 83(5), 77(5), 55(5), 43(5), 41(6). HRESIMS (*m/z*): calcd. for $C_{18}H_{22}O_4$ [M + Na]+: 325.1410, found: 325.1413. Yield: 39%.

Compound 12a: Yellow solid. IR (KBr, ν_{max}, cm^{-1}): 3560, 3400, 3006, 2954, 1657, 1637, 1618, 1599, 1467, 1426, 1263, 1147, 1022, 980. ^1H NMR (300 MHz, $CDCl_3$): δ 18.57 (s,

1H, OH, enol), 8.16 (d, 15.9, 2H, H-3'), 7.91 (d, 15.9, 2H, H-3'), 7.23 (dd, 8.3, 1.9, 1H, H-9'), 7.18 (d, 1.9, 2H, H-5'), 6.88 (d, 8.3, 2H, H-8'), 3.94 (s, 3H, m-OCH_3), 3.92 (s, 3H, p-OCH_3), 2.68 (t, 6.5, 2H, H-4), 2.56 (t, 6.6, 2H, H-6), 2.00 (quint, 6.6, 2H, H-5). ^{13}C NMR (75 MHZ, CDCl3) (75 MHz, $CDCl_3$): δ 202.46 (C-1), 196.36 (C-3), 188.11 (C-1'), 151.95 (C-6'), 149.31 (C-7'), 146.43 (C-3'), 128.13 (C-4'), 124.38 (C-3'), 120.44 (C-9), 111.94 (C-2), 111.03 (C-5'), 110.21 (C-8'), 56.04 (p-OCH_3), 55.96 (m-OCH_3), 39.41 (C-4), 35.22 (C-6), 18.97 (C-5). EIMS *m/z* (Rel int. %): [M^+] 302 (100), 301 (35), 271 (30), 246 (45), 231 (92), 215 (27), 191 (39), 165 (39), 151 (80), 133 (14), 118 (17), 91 (22), 77 (28), 69 (26), 55 (40), 42 (12). HRESIMS (*m/z*): calcd. for $C_{17}H_{18}O_5$ [M + H]+: 303.1227, found: 303.1229. Yield: 54%.

Compound 12b: Pale solid. IR (KBr, ν_{max}, cm^{-1}): 3436, 3014, 2952, 1657, 1607, 1589, 1519, 1475, 1442, 1240, 1135, 1026, 962. ^1H NMR (300 MHz, $CDCl_3$): δ 18.12 (s, 1H, OH, enol), 6.80-6.70 (m, 3H, H-5', H-8', H-9'), 3.88 (s, 3H, m-OCH_3), 3.85 (s, 3H, p-OCH_3), 3.34 (t, 7.5, 2H, H-2'), 2.89 (t, 7.6, 2H, H-3'), 2.66 (t, 6.4, 2H, H-4), 2.48 (t, 6.4, 2H, H-6), 1.97 (quint, 6.4, 2H, H-5). ^{13}C NMR (75 MHZ, CDCl3) (75 MHz, $CDCl_3$): δ 205.17 (C-1'), 198.42 (C-1), 195.36 (C-3), 148.89 (C-6'), 147.43 (C-7'), 133.71 (C-4'), 120.47 (C-9'), 113.26 (C-5'), 111.97 (C-8'), 111.31 (C-2), 56.01 (p-OCH_3), 55.92 (m-OCH_3), 42.66 (C-2'), 38.84 (C-4), 33.20 (C-6), 30.30 (C-3'), 19.14 (C-5). EIMS *m/z* (Rel int. %): [M^+] 304 (28), 165 (4), 151 (100), 138 (5), 107 (7), 91 (6), 69 (7), 55 (7). HRESIMS (*m/z*): calcd. for $C_{17}H_{20}O_5$ [M + Na]+: 327.1202, found: 327.1200. Yield: 74%.

Compound 12c: Yellow solid. IR (KBr, ν_{max}, cm^{-1}): 3437, 2956, 1660, 1619, 1582, 1516, 1422, 1264, 1141, 1027, 979. ^1H NMR (300 MHz, $CDCl_3$): δ 18.55 (d, 0.9, 1H, OH, enol), 8.21 (dd, 15.9, 0.9, 2H, H-3'), 7.93 (d, 15.9, 2H, H-3'), 7.23 (dd, 8.3, 1.9, 1H, H-9'), 7.18

(d, 1.9, 2H, H-5'), 6.88 (d, 8.3, 2H, H-8'), 3.94 (s, 3H, m-OCH_3), 3.92 (s, 3H, p-OCH_3), 2.56 (s, 2H, H-6), 2.43 (s, 2H, H-4), 1.10 (s, 6H, H-1''). ^{13}C NMR (75 MHZ, CDCl3) (75 MHz, $CDCl_3$): δ 201.91 (C-1), 196.17 (C-3), 187.43 (C-1'), 151.99 (C-6'), 149.35 (C-7'), 146.53 (C-3'), 128.16 (C-4'), 124.44 (C-2'), 120.28 (C-9'), 111.05 (C-8'), 110.77 (C-5'), 110.17 (C-2), 56.06 (p-OCH_3), 55.98 (m-OCH_3), 53.29 (C-5), 48.89 (C-6), 30.51 (C-4), 28.30 (C-1''). EIMS *m/z* (rel. int. %): [M^+] 330 (100), 329 (44), 312 (39), 299 (27), 269 (7), 246 (44), 231 (74), 215 (24), 193 (40), 191 (45), 164 (21), 151 (55), 138 (15), 118 (15), 83 (41), 77 (24): 69 (22), 55 (27), 43 (15). HRESIMS (*m/z*): calcd. for $C_{19}H_{22}O_5$ [M + H]+: 331.1540, found: 331.1547. Yield: 58%.

Compound 12d: Pale yellow solid. IR (KBr, ν_{max}, cm^{-1}): 3434, 3012, 2944, 1656, 1607, 1591, 1515, 1250, 1134, 1026, 956. ^1H NMR (300 MHz, $CDCl_3$): δ 18.12 (s, 1H, OH, enol), 6.81-6.79 (m, 3H, H-5', H-8', H-9'), 3.87 (s, 3H, m-OCH_3), 3.85 (s, 3H, p-OCH_3), 3.34 (t, 7.5, 2H, H-2'), 2.89 (t, 7.6, 2H, H-3'), 2.53 (s, 2H, H-6), 2.35 (s, 2H, H-4), 1.07 (s, 6H, H-1''). ^{13}C NMR (75 MHZ, CDCl3) (75 MHz, $CDCl_3$): δ 204.46 (C-1'), 197.52 (C-3), 195.06 (C-1), 148.79 (C-6'), 147.34 (C-7'), 133.53 (C-4'), 120.35 (C-9'), 112.08 (C-5'), 111.84 (C-8'), 111.20 (C-2), 55.90 (p-OCH_3), 55.81 (m-OCH_3), 52.58 (C-5), 46.71 (C-6), 42.25 (C-2'), 30.66 (C-4), 30.27 (C-3'), 28.15 (C-1''). EIMS *m/z* (Rel int. %): [M^+] 332 (24), 165 (3), 151 (100), 138 (5), 107 (7), 83 (8), 69 (5), 55 (5). HRESIMS (*m/z*): calcd. for $C_{19}H_{24}O_5$ [M + H]+: 333.1696, found: 333.1702. Yield: 78%.

Compound 13a: Pale yellow solid. IR (KBr, ν_{max}, cm^{-1}): 3446, 3105, 2954, 1659, 1618, 1597, 1567, 1500, 1442 1252, 1188, 1036, 930. ^1H NMR (300 MHz, $CDCl_3$): δ 18.53 (d, 0.8, 1H, OH, enol), 8.11 (d, 15.9, 2H, H-3'), 7.85 (d, 15.9, 2H, H-3'), 7.18 (d, 1.9, 2H, H-5'), 7.12 (dd, 8.1, 1.9, 1H, H-9), 6.88 (d, 8.1, 2H, H-8'), 6.01 (s, 2H, OCH_2O), 2.67 (t, 6.5,

2H, H-4), 2.54 (t, 6.5, 2H, H-6), 1.99 (quint, 6.5, H-5). ^{13}C NMR (75 MHZ, CDCl3) (75 MHz, $CDCl_3$): δ 202.36 (C-1), 196.21 (C-3), 188.15 (C-1'), 150.41 (C-6'), 148.52 (C-7'), 146.00 (C-3'), 129.65 (C-9'), 126.10 (C-4'), 120.83 (C-9'), 112.05 (C-2), 108.63 (C-8'), 107.27 (C-5'), 101.78 (CH_2), 39.40 (C-4), 35.17 (C-6), 18.99 (C-1''). EIMS *m/z* (Rel. int.%): [M^+] 286 (100), 285 (45), 268 (14), 258 (15), 230 (44), 175 (30), 165 (32), 145 (47), 135 (53), 117 (35), 89 (57), 77 (13), 63 (25), 55 (23), 44 (31). HRESIMS (*m/z*): calcd. for $C_{16}H_{14}O_5$ [M + Na]+: 309.0733, found: 309.0736. Yield: 60%.

Compound 13b: Yellow solid. IR (KBr, $ν_{max}$, cm^{-1}): 3071, 2952, 2893, 1682, 1664, 1490, 1459, 1444, 1245, 1190, 1039, 928. ^1H NMR (300 MHz, $CDCl_3$): δ 18.08 (s, 1H, OH, enol), 6.74 (m, 3H, H-5', H-8', H-9'), 5.91 (s, 2H, OCH_2O), 3.30 (t, 7.5, 2H, H-2'), 2.86 (t, 7.5, 2H, H-3'), 2.67 (t, 6.5, 2H, H-4), 2.48 (t, 6.8, 2H, H-6), 1.98 (quint, 6.5, H-5). ^{13}C NMR (75 MHZ, CDCl3) (75 MHz, $CDCl_3$): δ 205.02 (C-1'), 198.36 (C-1), 195.33 (C-3), 147.61 (C-6'), 145.85 (C-7'), 134.89 (C-4'), 121.36 (C-9'), 113.19 (C-5'), 109.15 (C-2), 108.22 (C-8'), 100.85 (CH_2), 42.81 (C-2'), 38.80 (C-4), 33.14 (C-6), 30.31 (C-3'), 19.12 (C-5). EIMS *m/z* (Rel int. %): [M^+] 288 (26), 270 (3), 135 (100), 112 (9), 91 (6), 77 (13), 69 (8), 55 (8). HRESIMS (*m/z*): calcd. for $C_{16}H_{16}O_5$ [M + Na]+: 311.0889, found: 311.0896. Yield: 68%.

Compound 13c: Yellow solid. IR (KBr, $ν_{max}$, cm^{-1}): 3432, 3103, 2957, 1658, 1616, 1596, 1502, 1448, 1417, 1263, 1104, 1038, 929. ^1H NMR (300 MHz, $CDCl_3$): δ 18.51 (s, 1H, OH, enol), 8.15 (d, 15.8, 2H, H-3'), 7.86 (d, 15.8, 2H, H-3'), 7.19 (d, 1.3, 2H, H-5'), 7.12 (dd, 8.1, 1.3, 1H, H-9'), 6.82 (d, 8.1, 2H, H-8'), 6.01 (s, 2H, OCH_2O), 2.56 (s, 2H, H-6), 2.42 (s, 2H, H-4), 1.07 (s, 6H, H-1''). ^{13}C NMR (75 MHZ, CDCl3) (75 MHz, $CDCl_3$): δ 201.80 (C-1), 195.99 (C-3), 187.43 (C-1'), 150.42 (C-6'), 148.53 (C-7'), 146.07 (C-3'),

129.66 (C-4'), 126.09 (C-2'), 120.65 (C-9'), 110.87 (C-2), 108.64 (C-8'), 107.28 (C-5'), 101.78 (CH_2), 53.24 (C-5), 48.83 (C-6), 30.51 (C-4), 28.30 (C-1''). EIMS *m/z* (Rel. int.%): [M^+] 314 (100), 313 (58), 296 (35), 281 (17), 253 (9), 230 (57), 215 (87), 193 (47), 175 (48), 145 (61), 135 (45), 117 (43), 89 (67), 77 (12), 69 (23), 55 (26), 41 (16). HRESIMS (*m/z*): calcd. for $C_{18}H_{18}O_5$ [M + Na]+: 337.1046, found: 337.1050. Yield: 66%.

Compound 13d: Yellow liquid. IR (KBr, ν_{max}, cm^{-1}): 2958, 2890, 1667, 1558, 1504, 1490, 1445, 1246, 1148, 1040, 929. ^1H NMR (300 MHz, $CDCl_3$): δ 18.08 (s, 1H, OH, enol), 6.74 (m, 3H, H-5', H-8', H-9'), 5.91 (s, 2H, OCH_2O), 3.30 (t, 7.5, 2H, H-2'), 2.85 (t, 7.5, 2H, H-3'), 2.53 (s, 2H, H-6), 2.35 (s, 2H, H-4), 1.08 (s, 6H, H-1''). ^{13}C NMR (75 MHZ, CDCl3) (75 MHz, $CDCl_3$): δ 204.37 (C-1'), 197.49 (C-3), 195.08 (C-1), 147.58 (C-6'), 145.81 (C-7'), 134.77 (C-4'), 121.29 (C-9'), 112.06 (C-2), 109.09 (C-9'), 108.17 (C-8'), 100.79 (CH_2), 52.55 (C-5), 46.66 (C-6), 42.48 (C-2'), 30.69 (C-4), 30.33 (C-3'), 28.18 (C-1''). EIMS *m/z* (Rel int. %): [M^+] 316 (26), 298 (3), 148 (6), 135 (100), 122 (5), 83 (8), 77 (10), 55 (4). HRESIMS (*m/z*): calcd. for $C_{18}H_{20}O_5$[M + H]+: 317.1383, found: 317.1389. Yield: 62%.

Compound 14a: Yellow solid. IR (KBr, ν_{max}, cm^{-1}): 3436, 3006, 2941, 2844, 1662, 1614, 1580, 1503, 1456, 1414, 1245, 1127, 1006, 979. ^1H NMR (300 MHz, $CDCl_3$): δ 18.56 (s, 1H, OH, enol), 8.19 (d, 15.8, 2H, H-3'), 7.86 (d, 15.8, 2H, H-3'), 3.91 (s, 6H, m-OCH3), 3.91 (s, 6H, p-OCH_3), 2.69 (t, 6.5, 2H, H-4), 2.56 (t, 6.5, 2H, H-6), 2.01 (quint, 6.5, 2H, H-4). ^{13}C NMR (75 MHZ, CDCl3) (75 MHz, $CDCl_3$): δ 202.45 (C-1), 196.35 (C-3), 188.16 (C-1'), 153.43 (m-OCH_3), 146.15 (C-3'), 140.87 (C-6', C-8'), 130.51 (C-4'), 122.06 (C-2'), 112.11 (C-2), 106.21 (C-5', C-9'), 61.03 (p-OCH_3), 56.22 (m-OCH_3), 39.36 (C-4), 35.09 (C-6), 18.94 (C-5). EIMS *m/z* (Rel. int.%): [M^+] 332(100), 317 (8), 301 (22), 287 (26), 276

(25), 261 (84), 246 (11), 181 (38), 105 (12), 91 (13), 69 (24), 55 (35), 42 (8). HRESIMS (*m/z*): calcd. for $C_{18}H_{20}O_6$ [M + H]+: 333.1332, found: 333.1340. Yield: 60%.

Compound 14b: White solid. IR (KBr, ν_{max}, cm^{-1}): 2956, 2937, 1656, 1590, 1459, 1421, 1241, 1126, 1038, 1011, 978, 921. ^1H NMR (500 MHz, $CDCl_3$): δ 18.09 (s, 1H, OH, enol), 6.49 (s, 2H, C-5'), 3.85 (s, 6H, m-OCH_3), 3.82 (s, 3H, p-OCH_3), 3.35 (t, 7.2, 2H, H-2'), 2.89 (t, 7.5, 2H, H-3'), 2.68 (t, 6.3, 2H, H-4), 2.49 (t, 6.0, 2H, H-6), 1.98 (quint, 6.3, 2H, H-4). ^{13}C NMR (75 MHZ, CDCl3) (75 MHz, $CDCl_3$): δ 205.06 (C-1'), 198.40 (C-1), 195.45 (C-3), 153.19 (C-6', C-8'), 136.89 (C-5', C-9'), 136.35 (C-4'), 113.28 (C-2), 105.56 (C-5'), 60.93 (p-OCH_3), 56.17 (m-OCH_3), 42.57 (C-2'), 38.84 (C-4), 33.16 (C-6), 31.05 (C-3'), 19.14 (C-5). EIMS *m/z* (Rel int. %): [M^+] 334(28), 195(18), 182(11), 181(100), 139(9), 69(13), 55(15). HRESIMS (*m/z*): calcd. for $C_{18}H_{22}O_6$ [M + Na]+: 357.1308, found: 357.1308. Yield: 38%.

Compound 14c: Yellow solid. IR (KBr, ν_{max}, cm^{-1}): 3437, 3093, 3003, 2978, 2948, 1662, 1620, 1579, 1503, 1461, 1413, 1242, 1124, 977. ^1H NMR (300 MHz, CDCl3): δ 18.54 (d, 0.9, 1H, OH, enol), 8.23 (dd, 15.9, 0.9, 2H, H-3'), 7.88 (d, 15.9, 2H, H-3'), 3.91 (s, 3H, m-OCH_3), 3.90 (s, 3H, p-OCH_3), 2.58 (s, 2H, H-6), 2.44 (s, 2H, H-4), 1.10 (s, 6H, H-1''). ^{13}C NMR (75 MHZ, CDCl3) (75 MHz, $CDCl_3$): δ 201.92 (C-1), 196.19 (C-3), 187.47 (C-1'), 153.47 (C-6', C-8'), 146.28 (C-3'), 140.92 (C-7'), 130.54 (C-4'), 121.90 (C-2'), 110.95 (C-2), 106.24 (C-5', C-9'), 61.06 (p-OCH_3), 56.25 (m-OCH_3), 53.26 (C-5), 48.78 (C-6), 30.54 (C-4), 28.30 (C-1''). EIMS *m/z* (Rel. int.%): [M^+] 360 (100), 359 (16), 345 (18), 329 (22), 276 (33), 261 (65), 245 (21), 193 (29), 181 (33), 91 (13), 83 (62), 69 (18), 55 (22), 44 (13). HRESIMS (*m/z*): calcd. for $C_{20}H_{24}O_6$ [M + H]+: 361.1645, found: 361.1650. Yield: 61%.

Compound 14d: Pale yellow solid. IR (KBr, ν_{max}, cm^{-1}): 3435, 3003, 2937, 1669, 1590, 1552, 1505, 1453, 1420, 1232, 1123, 1005, 973. ^{1}H NMR (500 MHz, $CDCl_3$): δ 18.11 (s, 1H, OH, enol), 6.49 (s, 2H, H-5'), 3.85 (s, 6H, m-OCH_3), 3.82 (s, 3H, p-OCH_3), 3.36 (t, 8.2, 2H, H-2'), 2.89 (t, 8.0, 2H, H-3'), 2.54 (s, 2H, H-4'), 2.36 (s, 2H, H-6), 1.08 (s, 6H, H-1''). ^{13}C NMR (75 MHZ, CDCl3) (75 MHz, CDCl3): δ 204.45 (C-1'), 197.59 (C-3), 195.22 (C-1), 153.20 (C-6', C-8'), 136.81 (C-4'), 136.36 (C-7'), 112.22 (C-2), 105.53 (C-5', C-9'), 60.93 (p-OCH_3), 56.16 (m-OCH_3), 52.69 (C-5), 46.77 (C-6), 42.24 (C-2'), 31.12 (C-3), 30.79 (C-4), 28.26 (C-1'). EIMS *m/z* (Rel int. %): [M^+] 362 (36), 195 (19), 181 (100), 148 (4), 83 (13), 69 (6), 55 (6). HRESIMS (*m/z*): calcd. for $C_{20}H_{26}O_6$ [M + Na]+: 385.1621, found: 385.1626. Yield: 30%.

Compound 15a: Red solid. IR (KBr, ν_{max}, cm^{-1}): 3436, 3099, 2939, 1643, 1589, 1526, 1464, 1402, 1256, 1056, 988. ^{1}H NMR (300 MHz, $CDCl_3$): δ 18.54 (d, 1H, 1.0, OH, enol), 8.13 (dd, 15.6, 1.0, 1H, H-3'), 7.98 (d, 15.6, 1H, H-2'), 7.57 (d, 8.9, 2H, H-6', H-8'), 6.87 (d, 8.9, 2H, H-5', H-9'), 3.05 (s, 6H, $N(CH_3)_2$) 2.65 (s, 6.5 Hz, 2H, H-6), 2.54 (s, 2H, 6.5, H-4), 1.98 (s, 2H, 6.5, H-5). ^{13}C NMR (75 MHZ, CDCl3) (75 MHz, $CDCl_3$): δ 202.56 (C-1), 196.43 (C-3), 187.50 (C-1'), 152.55 (C-7'), 147.96 (C-3'), 131.60 (C-5', C-9'), 123.07 (C-2'), 116.64 (C-4'), 111.90 (C-2), 111.52 (C-6', C-8'), 40.23 ($N(CH_3)_2$), 39.62 (C-4), 35.71 (C-6), 19.16 (C-5). EIMS *m/z* (Rel. int.%): [M^+] 285(100), 286 (19), 229(5), 214(10), 200(3), 174(20), 158(15), 147 (42), 134(63), 121(12), 103(6), 77(7), 73(5), 42(4). HRESIMS (*m/z*): calcd. for $C_{17}H_{19}NO_3$ [M + H]+: 286.1443, found: 286.1443. Yield: 55%.

Compound 15b: Orange oil. IR (KBr, ν_{max}, cm^{-1}): 3432, 3305, 3009, 2953, 2888, 1662, 1617, 1563, 1524, 1443, 1228, 1068, 947. ^{1}H NMR (300 MHz, $CDCl_3$): δ 18.17 (s, 1H, OH, enol), 7.15 (d, 8.9, 2H, H-6', H-8'), 6.66 (d, 8.9, 2H, H-5', H-9'), 3.31 (t, 7.6, 2H, H-

2'), 2.91 (s, 6H, $N(CH_3)_2$), 2.85 (t, 7.6, 2H, H-3'), 2.66 (t, 6.5, 2H, H-4), 2.48 (t, 6.5, 2H, H-6), 1.97 (quint, 6.5, 2H, H-5). ^{13}C NMR (75 MHZ, CDCl3) (75 MHz, $CDCl_3$): δ 205.32 (C-1'), 198.41 (C-3), 195.20 (C-1'), 149.19 (C-7'), 129.15 (C-4'), 129.07 (C-5', C-9'), 113.15 (C-6', C-8'), 112.97 (C-2), 42.72 (C-2'), 40.88 ($N(CH_3)_2$), 38.76 (C-4), 33.20 (C-6), 29.66 (C-3'), 19.05 (C-5). EIMS *m/z* (Rel. int.%): [M^+] 287 (11), 146 (2), 134 (100), 118 (7), 91 (5), 55 (2). HRESIMS (*m/z*): calcd. for $C_{17}H_{21}NO_3$ [M + H]+: 288.1594, found: 288.1605. Yield: 96%.

Compound 15c: Violet solid. IR (KBr, $ν_{max}$, cm^{-1}): 3436, 3092, 2956, 2866, 1650, 1584, 1533, 1498, 1403, 1282, 1162, 1042, 989. ^1H NMR (300 MHz, $CDCl_3$): δ 18.47 (d, 1.0, 1H, OH, enol), 8.16 (dd, 15.6, 1.0, 1H, H-3'), 7.98 (d, 15.6, 1H, H-2'), 7.57 (d, 8.9 Hz, 2H, H-6'**,** H-8'), 6.66 (d, 8.9, 2H, H-5', H-9'), 3.04 (s, 6H, $N(CH_3)_2$), 2.53 (s, 2H, H-6), 2.41 (s, 2H, H-4), 1.08 (s, 6H, H-1''). ^{13}C NMR (75 MHZ, CDCl3) (75 MHz, $CDCl_3$): δ 201.92 (C-1), 196.12 (C-3), 186.78 (C-1'), 152.55 (C-7'), 147.98 (C-3'), 131.57 (C-5', C-9'), 122.98 (C-2'), 116.37 (C-6', C-8'), 111.83 (C-2), 110.28 (C-6'), 53.43 (C-5), 49.32 (C-6), 40.17 ($N(CH_3)_2$), 30.49 (C-4), 28.35 (C-1'). EIMS *m/z* (Rel. int.%): [M^+] 313 (82), 312 (27), 207 (14), 174 (25), 147 (47), 146 (38), 134 (100) 121 (20), 105 (4), 91 (7), 77 (9), 55 (8), 44 (23). HRESIMS (*m/z*): calcd. for $C_{19}H_{23}NO_3$ [M + H]+: 314.1750, found: 314.1753. Yield: 61%.

Compound 15d: Orange oil. IR (KBr, $ν_{max}$, cm^{-1}): 2957, 2871, 1714, 1666, 1615, 1564, 1522, 1446, 1407, 1227, 1060, 948. ^1H NMR (300 MHz, $CDCl_3$): δ 18.16 (s, 1H, OH, enol), 7.14 (d, 8.6, 1H, H-6', H-8'), 6.69 (d, 8.6, 1H, H-5', H-9'), 3.31 (t, 7.6, 2H, H-2'), 2.90 (s, 6H, $N(CH_3)_2$), 2.85 (t, 7.6, 2H, H-3'), 2.51 (s, 2H, H-4), 2.34 (s, 2H, H-6), 1.06 (s, 6H, H-1''). ^{13}C NMR (75 MHZ, CDCl3) (75 MHz, $CDCl_3$): δ 204.80 (C-1), 197.67 (C-3),

195.07 (C-1'), 149.25 (C-7'), 129.22 (C-4'), 129.13 (C-5', C-9'), 113.07 (C-6', C-8'), 112.15 (C-2), 52.69 (C-5), 46.88 (C-6), 42.51 (C-2'), 40.97 ($N(CH_3)_2$), 30.73 (C-4), 29.81 (C-3'), 28.27 (C-1''). EIMS *m/z* (Rel. int.%): [M^+] 315 (12), 134 (100), 118 (6), 91 (4). HRESIMS (*m/z*): calcd. for $C_{19}H_{25}NO_3$ [M + H]+: 316.1907, found: 316.1906. Yield: 95%.

Compound 16a: Orange solid. IR (KBr, $\sqrt{}_{max}$, cm^{-1}): 3432, 3100, 2951, 1660, 1620, 1588, 1520, 1490, 1431, 1274, 1188, 1010, 982. ^1H NMR (300 MHz, $CDCl_3$): δ 18.49 (s, 1H, OH, enol), 8.22 (d, 15.8, 1H, H-3'), 7.84 (d, 15.8, 1H, H-2'), 7.57 (d, 8.8, 2H, H-6', H-8'), 7.36 (d, 8.8, 2H, H-5', H-9'), 2.69 (s, 6.5, 2H, H-6), 2.55 (s, 2H, 6.5, H-4), 2.00 (s, 2H, 6.5, H-5). ^{13}C NMR (75 MHZ, CDCl3) (75 MHz, $CDCl_3$): δ 202.31 (C-1), 196.20 (C-3), 188.30 (C-1'), 144.20 (C-3'), 136.82 (C-7'), 133.54 (C-4'), 130.14 (C-5', C-9'), 129.25 (C-6', C-8'), 123.60 (C-2'), 112.37 (C-2), 39.32 (C-4), 34.95 (C-6), 18.94 (C-5). EIMS *m/z* (Rel. int.%): [M^++2] 277 (16), [M^+] 275 (38), 258 (7), 241 (8), 205 (26), 185 (7), 165 (100), 139 (19), 137 (22), 115 (18), 102 (33), 101 (25), 75 (14), 69 (17), 55 (16), 42 (11). HRESIMS (*m/z*): calcd. for $C_{15}H_{13}ClO_3$ [M + Na]+: 299.0445, found: 299.0947. Yield: 70%.

Compound 16b: Orange liquid. IR (KBr, $\sqrt{}_{max}$, cm^{-1}): 3310, 3028, 2954, 1666, 1558, 1493, 1437, 1285, 1092, 962. ^1H NMR (300 MHz, $CDCl_3$): δ 18.02 (s, 1H, OH, enol), 7.28-7.17 (m, 4H, H-5', H-6'- H-9'), 3.33 (t, 7.6, 2H, H-2'), 2.91 (t, 7.6, 2H, H-3'), 2.67 (t, 6.6, 2H, H-4), 2.48 (t, 6.2, 2H, H-6), 1.97 (quint, 6.2, 2H, H-5). ^{13}C NMR (75 MHZ, CDCl3) (75 MHz, $CDCl_3$): δ 205.31 (C-1'), 198.77 (C-1), 195.91 (C-3), 140.01 (C-4'), 132.33 (C-7'), 130.50 (C-5', C-9'), 129.02 (C-6', C-8'), 113.67 (C-2), 42.89 (C-2'), 39.27 (C-4), 33.55 (C-3'), 30.31 (C-6), 19.59 (C-5). EIMS *m/z* (Rel. int.%): [M^++2] 280 (10), [M^+] 278 (31), 260 (9), 225 (9), 153 (18), 140 (21), 139 (83), 127 (20), 125 (66), 112 (100), 69 (34), 55 (28). HRESIMS (*m/z*): calcd. for $C_{15}H_{15}ClO_3$ [M + H]+: 279.0782, found: 279.0775. Yield: 77%.

Compound 16c: Orange solid. IR (KBr, $ν_{max}$, cm^{-1}): 3435, 3094,3056, 2953, 2925, 1650, 1625, 1591, 1569, 1494, 1426, 1270, 1041, 981. ^1H NMR (500 MHz, $CDCl_3$): δ 18.47 (s, 1H, OH, enol), 8.26 (d, 15.8, 1H, H-3'), 7.86 (d, 15.8, 1H, H-2'), 8.25 (d, 8.9, 2H, H-6', H-8'), 7.79 (d, 8.9, 2H, H-5', H-9'), 2.62 (s, 2H, H-6), 2.46 (s, 2H, H-4), 1.12 (s, 6H, H-1''). ^{13}C NMR (75 MHZ, CDCl3) (75 MHz, $CDCl_3$): δ 201.78 (C-1), 196.01 (C-3), 187.57 (C-1'), 144.31 (C-3'), 136.85 (C-7'), 133.57 (C-4'), 130.16 (C-5', C-9'), 129.29 (C-6', C-8'), 123.45 (C-2'), 111.22 (C-2), 53.20 (C-5), 48.64 (C-6), 30.57 (C-4), 28.31 (C-1''). EIMS *m/z* (Rel. int.%): [M^++2] 306 (15), 305 (24) [M^+] 304 (42), 303 (48), 289 (7), 204 (22), 206 (7), 193 (100), 167 (20), 165 (43), 157 (10), 137 (29), 115 (22), 102 (35), 101 (27), 75 (11), 69 (17), 55 (20), 41 (13). HRESIMS (*m/z*): calcd. for $C_{17}H_{17}ClO_3$ [M + H]+: 305.0938, found: 305.0948. Yield: 72%.

Compound 16d: Orange liquid. IR (KBr, $ν_{max}$, cm^{-1}): 3028, 2959, 2930, 1666, 1562, 1494, 1440, 1268, 1093, 950. ^1H NMR (300 MHz, $CDCl_3$): δ 18.03 (s, 1H, OH, enol), 7.26-7.17 (m, 4H, H-5' - H-9'), 3.34 (t, 8.0, 2H, H-2'), 2.91 (t, 8.0, 2H, H-3'), 2.54 (s, 2H, H-4), 2.35 (s, 2H, H-6), 1.07(s, 6H, H-1''). ^{13}C NMR (75 MHZ, CDCl3) (75 MHz, $CDCl_3$): δ 204.28 (C-1'), 197.53 (C-1), 195.29 (C-3), 139.54 (C-4'), 131.95 (C-7'), 130.07 (C-5', C-9'), 128.61 (C-6', C-8'), 112.20 (C-2), 52.70 (C-5), 46.76 (C-6), 42.15 (C-2'), 30.85 (C-3'), 29.99 (C-1''), 28.31 (C-1'). EIMS *m/z* (Rel. int.%): [M^++2] 308 (11), [M^+] 306 (32), 288 (8), 181 (16), 167 (77), 153 (11), 140 (100), 127 (19), 125 (67), 111 (14), 103 (14), 89 (12), 83 (46), 69 (22), 55(18), 43 (16). HRESIMS (*m/z*): calcd. for $C_{17}H_{19}ClO_3$ [M + H]+: 307.1095, found: 307.1092. Yield: 83%.

Compound 17a: Orange solid. IR (KBr, $ν_{max}$, cm^{-1}): 3437, 3100, 2950, 1658, 1620, 1583, 1562, 148, 1432, 1181, 1071, 982. ^1H NMR (300 MHz, $CDCl_3$): δ 18.48 (d, 0.8 Hz, 1H,

OH, enol), 8.23 (dd, 15.9, 0.8, 1H, H-3'), 7.82 (d, 15.9, 1H, H-2'), 7.53-7.46 (m, 4H, H-5'-H-6'), 2.69 (t, 6.5, 2H, H-4), 2.55 (t, 6.4, 2H, H-6), 2.00 (quint, 6.5, 2H, H-5). ^{13}C NMR (75 MHZ, CDCl3) (75 MHz, $CDCl_3$): δ 202.29 (C-1), 196.16 (C-3), 188.26 (C-1'), 144.23 (C-3'), 133.94 (C-4'), 132.20 (C-6', C-8'), 130.31 (C-5', C-9'), 125.27 (C-2'), 123.70 (C-7'), 111.37 (C-2), 39.30 (C-4), 34.94 (C-6), 18.92 (C-5). EIMS *m/z* (Rel. int.%): [M^++2] 322 (25), 321 (34), [M^+] 320 (28), 319 (31), 304 (6), 302 (6), 266 (11), 264 (11), 251 (20), 249 (23), 210 (22), 208 (22), 165 (100), 129 (17), 115 (26), 102 (74), 69 (22), 55 (21), 42 (14). HRESIMS (*m/z*): calcd. for $C_{15}H_{13}BrO_3$ [M + H]+: 321.0120, found: 321.0121. Yield: 68%.

Compound 17b: Yellow solid. IR (KBr, $\sqrt{}_{max}$, cm^{-1}): 3431, 3099, 2941, 1661, 1624, 1584, 1563, 1486, 1439, 1070, 980. ^{1}H NMR (300 MHz, $CDCl_3$): δ 18.57 (s, 1H, OH, enol), 8.23 (d, 15.9, 1H, H-3'), 7.92 (d, 15.9, 1H, H-2'), 7.58-7.51 (m, 4H, H-5'-H-9'), 4.17 (ddd, 13.3, 5.5, 1.9, 1H, H-4), 4.10 (d, 1.9, 1H, OH), 2.86-2.81 (m, 2H, H-6), 2.46-2.38 (m, 1H, H-5), 1.94-1.79 (m, 1H, H-5). ^{13}C NMR (75 MHZ, CDCl3) (75 MHz, $CDCl_3$): δ 201.56 (C-1), 196.47 (C-3), 188.37 (C-1'), 145.59 (C-3'), 133.68 (C-4'), 132.45 (C-6', C-8'), 130.54 (C-5', C-9'), 125.90 (C-2'), 122.57 (C-7'), 109.72 (C-2), 72.21 (C-4), 33.01 (C-5), 27.11 (C-6). EIMS *m/z* (Rel. int.%): [M^++2] 338 (9), 337 (4), [M^+] 336 (10), 335 (2), 266 (11), 264 (12), 211 (21), 209 (18), 181 (19), 129 (26), 115 (40), 102 (100), 89 (12), 76 (25), 69 (22), 51 (18), 36 (31). HRESIMS (*m/z*): calcd. for $C_{15}H_{13}BrO_4$ [M + Na]+: 358.9889, found: 358.9892. Yield: 64%.

Compound 17c: Orange solid. IR (KBr, $\sqrt{}_{max}$, cm^{-1}): 3436, 3095, 3053, 2952, 1651, 1626, 1587, 1566, 1518, 1491, 1406, 1286, 1073, 981. ^{1}H NMR (300 MHz, $CDCl_3$): δ 18.46 (d, 1.0, 1H, OH, enol), 8.28 (dd, 15.9, 1.0, 1H, H-3'), 7.85 (d, 15.9, 1H, H-2'), 7.55-7.48 (m, 4H, H-5'-H-9'), 2.58 (s, 2H, H-6), 2.43 (s, 2H, H-4), 1.10 (s, 6H, H-1''). ^{13}C NMR (75 MHZ,

CDCl3) (75 MHz, $CDCl_3$): δ 201.82 (C-1), 196.06 (C-3), 187.60 (C-1'), 144.40 (C-3'), 134.00 (C-4'), 132.27 (C-6', C-8'), 130.37 (C-5', C-9'), 125.34 (C-2'), 123.58 (C-7'), 111.26 (C-3), 53.22 (C-6), 48.67 (C-4), 30.61 (C-5), 28.33 (C-1''). EIMS *m/z* (Rel. int.%): [M^++2] 350 (23), 349 (31), [M^+] 348 (26), 347 (29), 266 (10), 264 (12), 251 (13), 249 (12), 211 (20), 209 (21), 193 (100), 157 (9), 129 (17), 115 (21), 102 (60), 83 (21), 69 (16), 55 (18), 41 (12). HRESIMS (*m/z*): calcd. for $C_{17}H_{17}BrO_3$ [M + Na]+: 371.0253, found: 371.0260. Yield: 71%.

Compound 18a: Dark yellow solid. IR (KBr, ν_{max}, cm^{-1}): 3444, 3048, 2945, 1656, 1622, 1558, 1526, 1477, 1435, 1289, 1071, 984. ^{1}H NMR (300 MHz, $CDCl_3$): δ 18.45 (s, 1H, OH, enol), 8.22 (d, 15.9, 1H, H-3'), 7.84-7.77 (m, 2H, H-2', H-5'), 7.57-7.50 (m, 2H, H-7', H-8'), 7.27 (t, 7.8, 2H, H-8'), 2.71 (t, 6.5, 2H, H-4), 2.56 (t, 6.5, 2H, H-6), 1.10 (s, 6.5 Hz, 6H, H-5). ^{13}C NMR (75 MHZ, CDCl3) (75 MHz, $CDCl_3$): δ 202.31 (C-1), 196.28 (C-3), 188.35 (C-1'), 143.87 (C-3'), 137.17 (C-4'), 133.60 (C-5'), 131.55 (C-7'), 130.48 (C-8'), 127.62 (C-2'), 124.55 (C-9'), 123.17 (C-6'), 112.55 (C-2), 39.33 (C-4), 34.94 (C-6), 18.98 (C-5). EIMS *m/z* (Rel. int.%): [M^++2] 322 (14), 321 (14), [M^+] 320 (15), 319 (14), 304 (3), 302 (4), 266 (6), 264 (6), 251 (10), 249 (10), 241 (12), 211 (12), 209 (12), 185 (12), 165 (100), 157 (5), 129 (11), 115 (17): 102 (52), 69 (16), 55 (15), 42 (11). HRESIMS (*m/z*): calcd. for $C_{15}H_{13}BrO_3$ [M + H]+: 321.0120, found: 321.0120. Yield: 65%.

Compound 18b: Dark yellow solid. IR (KBr, ν_{max}, cm^{-1}): 3436, 3098, 2955, 1667, 1623, 1553, 1520, 1476, 1434, 1273, 1040, 980. ^{1}H NMR (300 MHz, $CDCl_3$): δ 18.44 (s, 1H, OH, enol), 8.26 (d, 15.9, 1H, H-3'), 7.85-7.78 (m, 2H, H-2', H-5'), 7.57-7.50 (m, 2H, H-7', H-8'), 7.27 (t, 7.8, 2H, H-8'), 2.84 (s, 2H, H-4), 2.44 (s, 2H, H-6), 1.10 (s, 6H, H-1''). ^{13}C NMR (75 MHZ, CDCl3) (75 MHz, $CDCl_3$): δ 201.78 (C-1), 196.04 (C-3), 187.57 (C-1'),

143.94 (C-3'), 137.18 (C-4'), 133.61 (C-5'), 131.55 (C-7'), 130.48 (C-8'), 127.62 (C-9'), 124.38 (C-2'), 123.18 (C-6'), 111.35 (C-2), 53.20 (C-5), 48.62 (C-6), 30.63 (C-4), 28.34 (C-1''). EIMS *m/z* (Rel. int.%): [M^++2] 350 (15), 349 (16), [M^+] 348 (15), 347 (13), 266 (5), 264 (5), 251 (6), 249 (6), 211 (13), 209 (12), 193 (100), 181 (5), 157 (5), 129 (11), 115 (14), 102 (43), 83 (13), 69 (11), 55 (12), 41 (8). HRESIMS (*m/z*): calcd. for $C_{17}H_{17}BrO_3$ [M + H]+: 349.0433, found: 349.0436. Yield: 69%.

Compound 19a: Yellow solid. IR (KBr, ν_{max}, cm^{-1}): 3438, 3096, 2958, 2897, 1655, 1629, 1597, 1509, 1440, 1407, 1348, 1278, 1113, 979. ^1H NMR (300 MHz, $CDCl_3$): δ 18.37 (s, 1H, OH, enol), 8.34 (d, 15.8, 1H, H-3'), 8.25 (d, 8.9, 2H, H-6', H-8'), 7.87 (d, 15.8, 1H, H-2'), 7.78 (d, 8.9, 2H, H-5', H-9'), 2.74 (t, 6.4, 2H, H-6), 2.58 (t, 6.5, 2H, H-4), 2.04 (t, 6.4, 2H, H-5), 1.98 (quint, 6.5, 2H), 1.91 (quint, 7.5, 2H). ^{13}C NMR (75 MHZ, CDCl3) (75 MHz, $CDCl_3$): δ 202.27 (C-1), 196.26 (C-3), 188.13 (C-1'), 148.67 (C-7'), 141.88 (C-3'), 141.18 (C-4'), 129.41 (C-5', C-9'), 127.54 (C-2'), 124.23 (C-6', C-8'), 112.83 (C-2), 39.26 (C-6), 34.74 (C-4), 18.94 (C-5). EIMS *m/z* (Rel. int.%): [M^+] (28), 269 (5), 257 (25), 239 (9), 231 (5), 216 (10), 214 (11), 186 (7), 176 (20), 166 (11), 165 (100), 146 (15), 134 (18), 130 (34), 119 (21), 118 (24), 115 (15), 106 (15), 102 (27), 91 (14), 76 (12), 69 (21), 55 (22), 42 (16), 39 (10). HRESIMS (*m/z*): calcd. for $C_{15}H_{13}NO_5$ [M + H]+: 288.0866, found: 288.0870. Yield: 60%.

Compound 19b: Orange solid. IR (KBr, ν_{max}, cm^{-1}): 3434, 3099, 2956, 2930, 1659, 1629, 1597, 1519, 1432, 1405, 1344, 1278, 1108, 962. ^1H NMR (300 MHz, $CDCl_3$): δ 18.36 (s, 1H, OH, enol), 8.37 (d, 15.8, 1H, H-3'), 8.25 (d, 8.9, 2H, H-6', H-8'), 7.89 (d, 15.8, 1H, H-2'), 7.79 (d, 8.9, 2H, H-5', H-9'), 2.62 (s, 2H, H-6), 2.46 (s, 2H, H-4), 1.12 (s, 6H, H-1''). ^{13}C NMR (75 MHZ, CDCl3) (75 MHz, $CDCl_3$): δ 207.76 (C-1), 196.06 (C-3), 187.30 (C-1'),

148.66 (C-7'), 141.96 (C-3'), 141.17 (C-4'), 129.39 (C-5', C-9'), 127.35 (C-6', C-8'), 124.21 (C-2'), 111.64 (C-3), 53.10 (C-6), 48.39 (C-4), 30.66 (C-5), 28.31 (C-1''). EIMS *m/z* (Rel. int.%): [M^+] (26), 285 (23), 268 (6), 259 (2), 231 (4), 214 (11), 193 (100), 176 (20), 146 (14), 130 (28), 119 (20) 106 (16), 83 (22), 69 (17), 55 (21), 43 (9), 41 (13), 39 (8). HRESIMS (*m/z*): calcd. for $C_{17}H_{17}NO_5$ [M + H]+: 316.1179, found: 316.1186. Yield: 65%.
